# Isomerization of 5-(2*H*-Azirin-2-yl)oxazoles: An Atom-Economic Approach to 4*H*-Pyrrolo[2,3-*d*]oxazoles

**DOI:** 10.3390/molecules26071881

**Published:** 2021-03-26

**Authors:** Timur O. Zanakhov, Ekaterina E. Galenko, Mariya A. Kryukova, Mikhail S. Novikov, Alexander A. Khlebnikov

**Affiliations:** Institute of Chemistry, St. Petersburg State University, 7/9 Universitetskaya nab., 199034 St. Petersburg, Russia; zanahovtim@rambler.ru (T.O.Z.); ekaterina.e.galenko@gmail.com (E.E.G.); mary_kryukova@mail.ru (M.A.K.); m.novikov@spbu.ru (M.S.N.)

**Keywords:** isomerization, azirine, oxazole, diazo compounds, pyrrolo[2,3-*d*]oxazole

## Abstract

An atom economical method for the preparation of variously substituted 4*H*-pyrrolo[2,3-*d*]oxazoles was developed on the basis of thermal isomerization of 5-(2*H*-azirin-2-yl)oxazoles. The latter were prepared by Rh_2_(oct)_4_ catalyzed reaction of 2-(3-aryl/heteroaryl)-2-diazoacetyl-2*H*-azirines with a set of substituted acetonitriles, benzonitriles, acrylonitrile and fumaronitrile. According to DFT calculations the transformation of 5-(2*H*-azirin-2-yl)oxazole to 4*H*-pyrrolo[2,3-*d*]oxazole occurs through the nitrenoid-like transition state to give a 3a*H*-pyrrolo[2,3-*d*]oxazole intermediate, followed by 1,5-H-shift.

## 1. Introduction

**Scheme 1:**
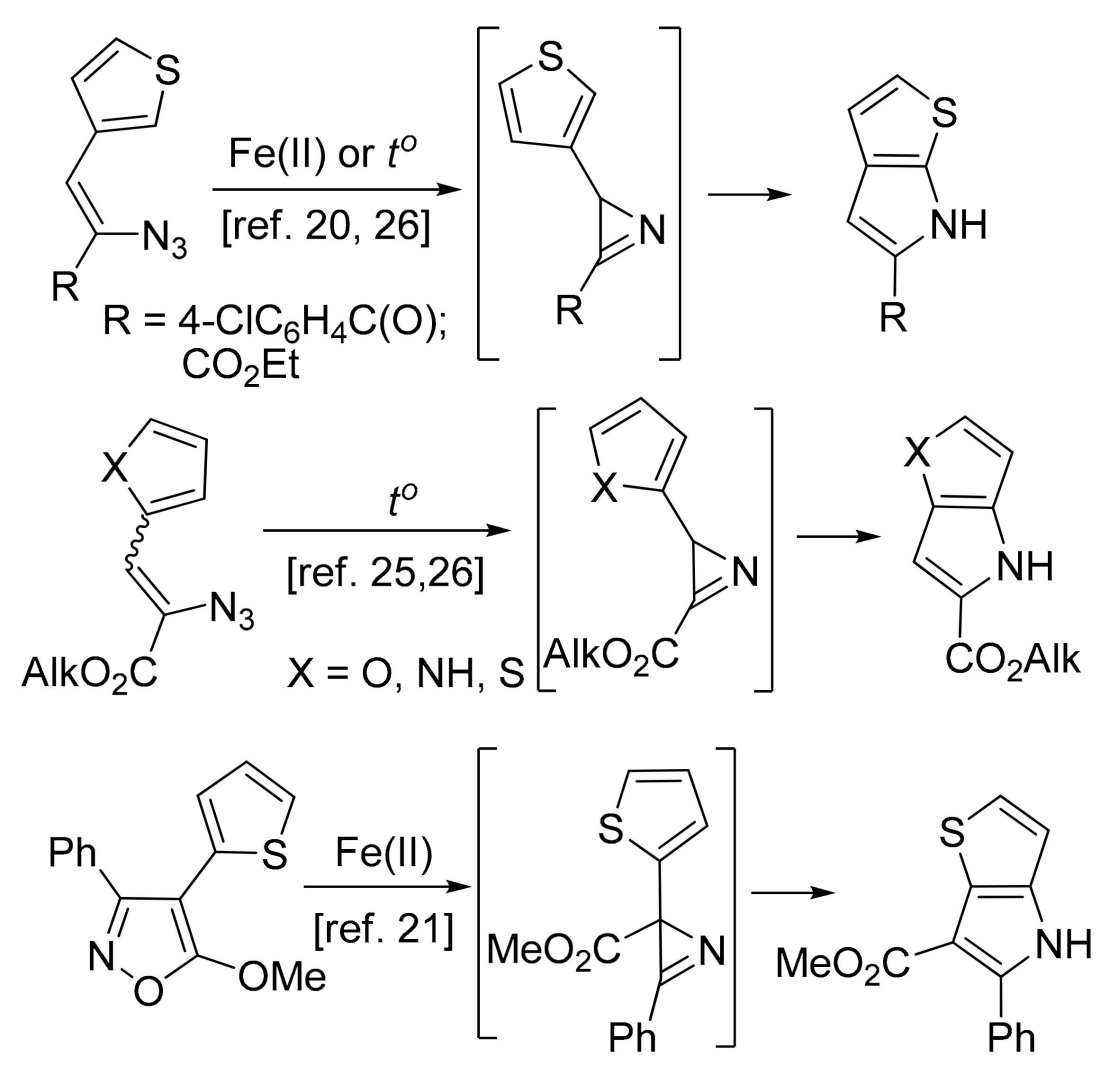
Synthesis of 5,5-heterobicyclic systems via isomerization of 2*H*-azirines.

Intramolecular ring-to-ring isomerization, while having 100% atom economy [1], appears to be a very attractive synthetic approach. Such isomerization of substituted azirines, which occurs easily due to the high strain of the three-membered ring under thermolysis, photolysis or catalysis, is widely used for the synthesis of various heterocycles [2,3,4]. 2-Carbonyl-substituted 2*H*-azirines can be isomerized to isoxazoles [2,3,4,5,6,7,8] or oxazoles [2,3,4,5,8], 2-vinyl-2*H*-azirines to pyrroles [9,10,11,12], and 2-(hydrazonomethyl) and 2-(iminomethyl)-2*H*-azirines to pyrazoles [13,14]. The isomerization can lead also to six-membered rings, thus, variously substituted pyridines were prepared from 2-propargyl [15,16,17,18], 2-allyl [19] or 2-vinyl-substituted 2*H*-azirines [10]. 5,6-Bicyclic heterocycles are also available via isomerization of azirines. Various indoles [2,3,4,20,21] and pyrazolo[1,5-*a*]pyridines [22,23,24,25] were synthesized by thermal or catalytic isomerization of 2-aryl- and 2-(pyrid-2-yl)-2*H*-azirines, respectively. Meanwhile, this isomerization has been used less for the preparation of 5,5-heterobicyclic systems. Single representatives of 6*H*-thieno[2,3-*b*]pyrrole [20,26], 4*H*-thieno[3,2-*b*]pyrrole [21,25,26], 4*H*-furo[3,2-*b*]pyrrole [26], 1,4-dihydropyrrolo[3,2-*b*]pyrrole [26] have been synthesized by rearrangement of vinyl azides or isoxazole, which presumably proceeds via the intermediate formation of the corresponding 2*H*-azirines followed by their isomerization (Scheme 1).

**Scheme 2:**
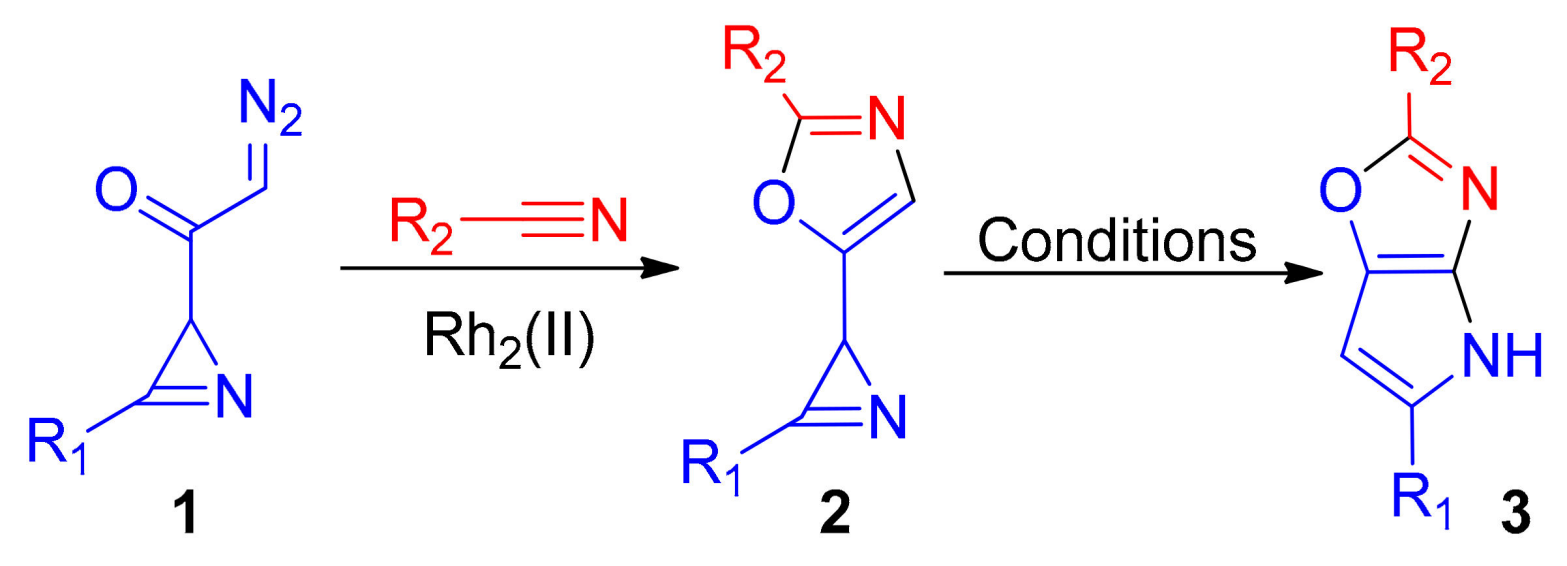
Approach for the synthesis of 4*H*-pyrrolo[2,3-*d*]oxazoles **3**.

5,5-Heterobicyclic systems with three and more heteroatoms in the bicyclic framework, as far as we know, have not been prepared by using the azirine methodology. This is not least due to the difficulties in the synthesis of polyheteroatomic azoles bearing an azirin-2-yl substituent. One of the possible solutions to this problem could be the use of 2-diazoacetylazirine building blocks **1**, the convenient synthesis of which we have recently reported [27,28]. Preliminary results on the Rh(II)-catalyzed reaction of 2-diazoacetylazirines with acetonitrile demonstrated the principal utility of this protocol for the preparation of 5-(2*H*-azirin-2-yl)oxazoles **2** [27]. We hypothesized that oxazoles **2** can be isomerized to 4*H*-pyrrolo[2,3-*d*]oxazoles **3** (Scheme 2), which have not yet been characterized in the literature. Only one compound of this class, 4*H*-pyrrolo[2,3-*d*]oxazole-5-carboxylic acid, was mentioned in connection with the study of *D*-amino acid oxidase inhibition, however, its synthesis, physical and spectral data were not published [29]. Here, we report the synthesis of a series of variously substituted 5-(2*H*-azirin-2-yl)oxazoles **2**, their conversion to 4*H*-pyrrolo[2,3-*d*]oxazoles **3** and full characterization of the latter, including X-ray structural study.

## 2. Results and Discussion

**Figure 1:**
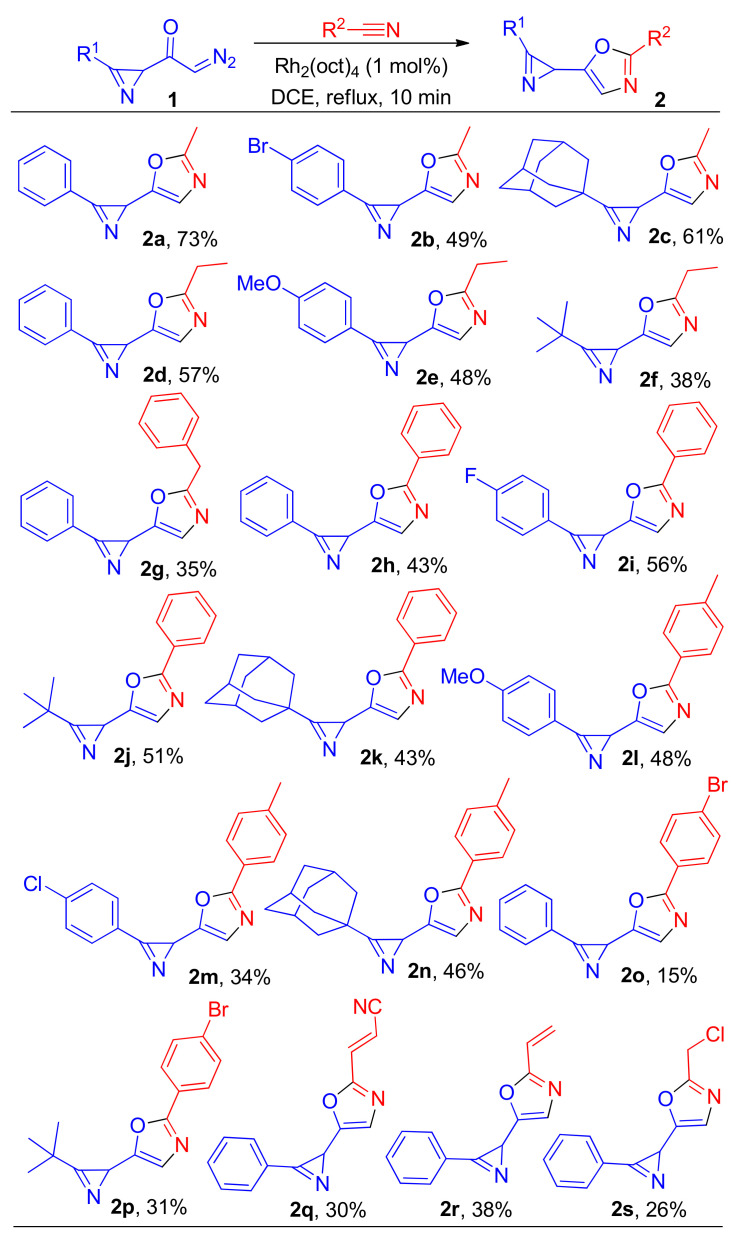
Synthesis of 5-(2*H*-azirin-2-yl)oxazoles **2**.

A series of 5-(2*H*-azirin-2-yl)oxazoles **2** was synthesized from 2-(3-aryl/heteroaryl)-2-diazoacetyl-2*H*-azirines **1** [28] by Rh_2_(oct)_4_ catalyzed reaction [27] with a set of substituted acetonitriles, benzonitriles, acrylonitrile and fumaronitrile in a 15–73% yield (Figure 1).

**Figure 2:**
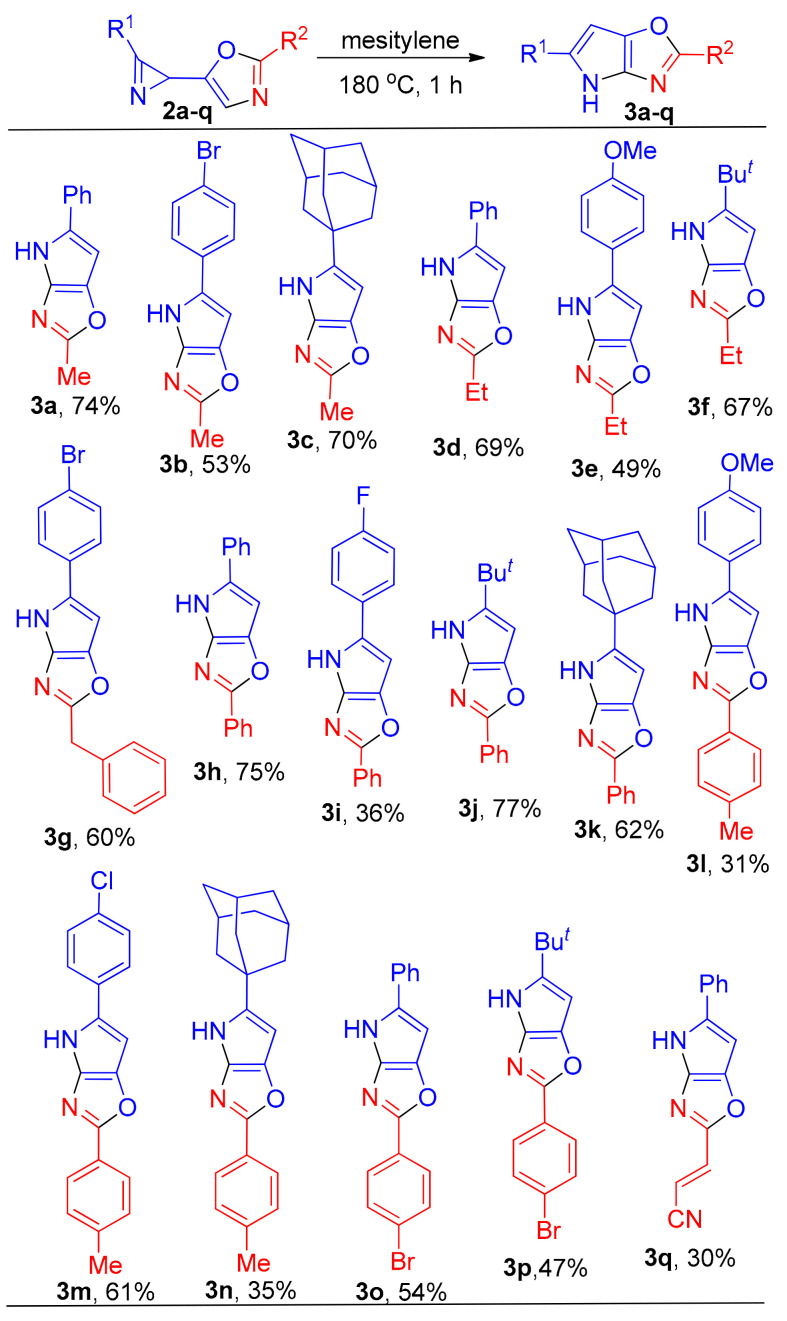
Synthesis of 4*H*-pyrrolo[2,3-*d*]oxazoles **3**.

Intramolecular ring-to-ring isomerization of azirines with the formation of five-membered nitrogen heterocyles most often occurs under conditions of metal catalysis or thermolysis at high temperatures [2,3,4,5,6,7,8,9,10,11,12,13,14,15,16,17,18,19,20,21,22,23,24,25,26]. Using azirine **2a** as a test compound, various isomerization conditions (catalysts, solvents, temperatures) were tested to achieve maximum yield of 4*H*-pyrrolo[2,3-*d*]oxazole **3a**. First of all, iron-containing catalysts, which successfully have been used for isomerization of different azirines, were tested. However, the use of FeCl_2_·4H_2_O (20 mol%) at various temperatures in acetonitrile (85 °C), 1,4-dioxane (110 °C), mesitylene (170 °C), DMSO (170 °C), anhydrous FeCl_2_ (20 mol%) in acetonitrile (85 °C), 1,4-dioxane (110 °C), mesitylene (150 °C), DMSO (150 °C) and anhydrous FeCl_3_ (20 mol%) in acetonitrile (85 °C), 1,4-dioxane (110 °C), toluene (110 °C), THF (66 °C) resulted in complete resinification of the reaction mixtures. Similar results were obtained when Co(acac)_2_·(20 mol%), Co(acac)_3_·(20 mol%) were tried as catalysts. The use of ZnBr_2_ (20 mol%), CuOAc (20 mol%), [(MeCN)_4_Cu]BF_4_ (20 mol%), Cu(tfacac)_2_ (20 mol%), Yb(Tf)_3_ (20 mol%) in acetonitrile (85 °C), 1,4-dioxane (110 °C), toluene (110 °C), DMF (110 °C) also resulted in complete resinification of the reaction mixtures. Heating of azirine **2a** in the presence of NiCl_2_·6H_2_O (20 mol%) in acetonitrile (85 °C), 1,4-dioxane (110 °C), toluene (110 °C) gave only traces of pyrrolooxazole **3a**. It was suggested that the failure of these experiments is due to the non-selective interaction of the catalysts with the polyheteroatomic substrate. Therefore, in the further experiments, it was decided to carry out the thermolysis in the absence of catalysts in an inert solvent. Heating azirine **2a** in mesitylene at 170 °C for 3 h afforded pyrrolooxazole **3a** in 70% yield. An increase in temperature to 180 °C reduced the reaction time to 1 h. These reaction conditions were used for the isomerization of 5-(2*H*-azirin-2-yl)oxazoles **2b-q** to 4*H*-pyrrolo[2,3-*d*]oxazoles **2b-q** (Figure 2).

**Figure 3:**
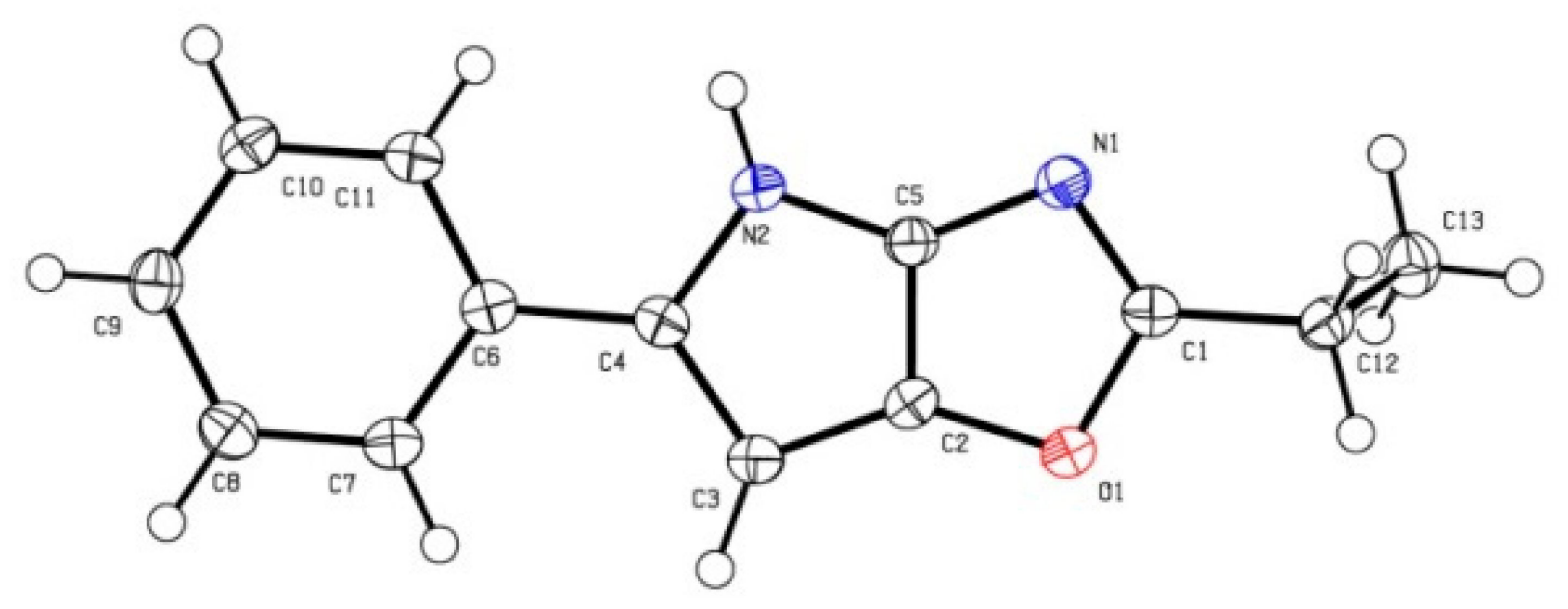
Perspective view of compounds **3d** showing thermal ellipsoids at 50% probability level.

The reaction tolerates a variety of substituted aryl and alkyl groups at the 3 position (R^1^) in azirine fragment and at the 2 position (R^2^) in oxazole fragment of starting compounds **2** and affords the desired products **3** in generally good yields (30–77%). Whereas heating compounds **2r** and **2s** with vinyl and chloromethyl substituents resulted in complete resinification of the reaction mixtures. All new compounds were characterized by ^1^H, ^13^C-NMR and HRMS methods. Moreover, the structure of **3d** was also confirmed by single-crystal X-ray diffraction analysis (Figure 3). Pyrrolooxazoles **3a-q** are non-hygroscopic crystalline solids, which are stable under an air atmosphere for at least 3 months at rt.

**Scheme 3:**
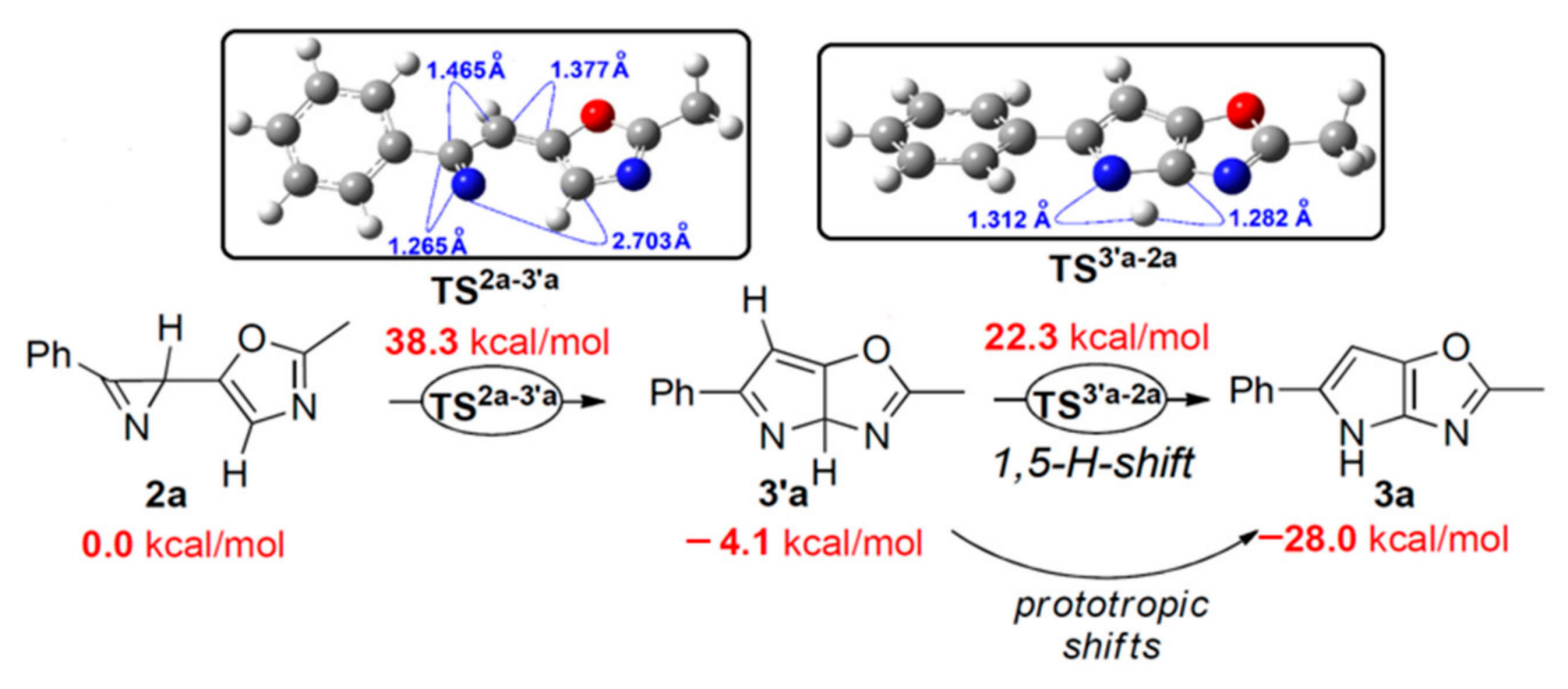
Relative Gibbs free energies for the energy profile of compound **2a** thermolysis in mesitylene (in kcal/mol, 398 K, DFT B3LYP-D3/6-311+G(d,p) level with SMD model for mesitylene).

To shed some light on the mechanism for the isomerization of azirines **2** into pyrrolooxazoles **3,** the DFT calculations of the transformation **2a**→**3a** was performed at the DFT B3LYP-D3/6-311+G(d,p) level of theory with SMD model for mesitylene (Scheme 3, for details of the calculations see the Supplementary Materials).

According to the calculations, the transformation of the azirine ring of **2a** occurs through the nitrenoid-like transition state **TS^2a-3′a^** with high relative Gibbs free energy. However, this activation barrier (38.3 kcal/mol) can be overcome under the harsh experimental conditions to give 2-methyl-5-phenyl-3a*H*-pyrrolo[2,3-*d*]oxazole **3′a**. The intermediate **3′a** can be further aromatized to final product **3′a** by 1,5-H-shift (**TS^3′a-3a^**) through the surmountable activation barrier under the experimental conditions. An intermolecular 1,2-prototropic shift at the last stage also cannot be excluded if it has a lower energy barrier.

**Scheme 4:**
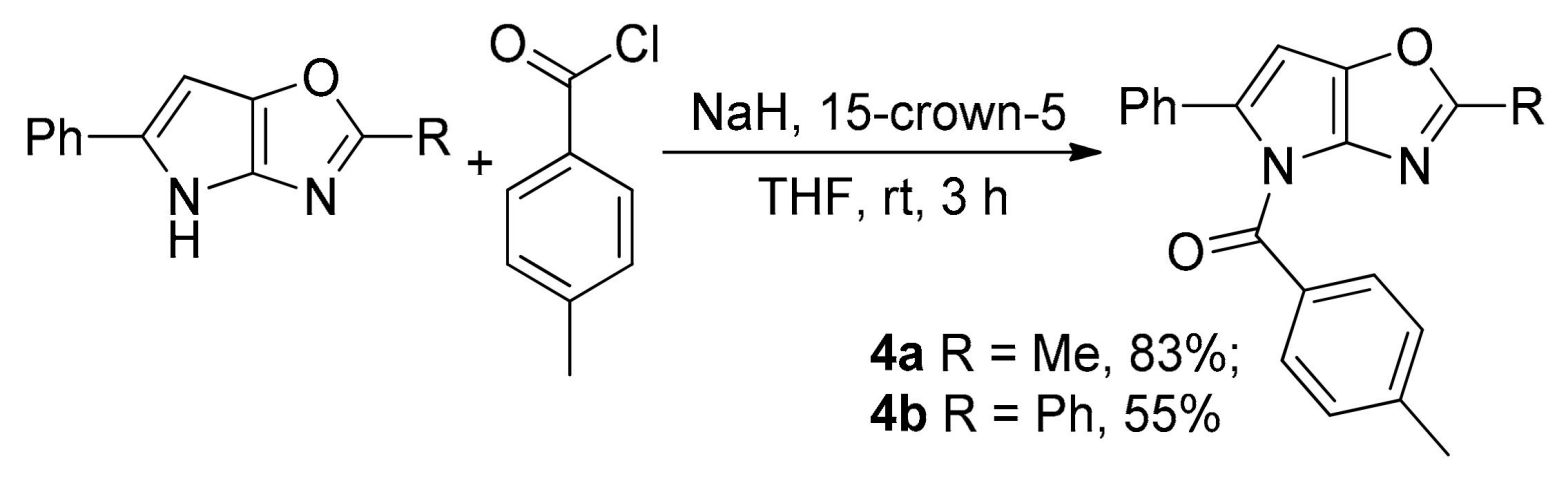
Acylation of compounds **3a,h**.

The pyrrole nitrogen of 4*H*-pyrrolo[2,3-*d*]oxazoles **3** can be protected by acylation. Thus, the reaction of compound **3a,h** with *p-*toluoyl chloride mediated with NaH/15-crown-5 afforded compounds **4a,b** in moderate-to-good yield (Scheme 4).

## 3. Materials and Methods

### 3.1. General Instrumentation

Melting points were determined on a melting point apparatus SMP30 (Research Park, Saint Petersburg State University, Saint Petersburg, Russia). ^1^H-NMR (400 MHz) and ^13^C-NMR (100 MHz) spectra were recorded on a Bruker AVANCE 400 spectrometer (Research Park, Saint Petersburg State University, Saint Petersburg, Russia) in CDCl_3_ or DMSO-*d*_6_. Chemical shifts (δ) are reported in parts per million downfield from tetramethylsilane (TMS, δ = 0.00). ^1^H-NMR spectra were calibrated according to the residual peak of CDCl_3_ (7.26 ppm) and DMSO-*d*_6_ (2.50 ppm). For all new compounds, ^13^C{^1^H} and ^13^C-DEPT-135 spectra were recorded and calibrated according to the peak of CDCl_3_ (77.00 ppm) and DMSO-*d*_6_ (39.51 ppm). Electrospray ionization (ESI), positive mode, mass spectra were measured on a Bruker MaXis mass spectrometer, HRMS-ESI-QTOF (Research Park, Saint Petersburg State University, Saint Petersburg, Russia). Single-crystal X-ray data were collected by means of a HyPix diffractometer (Research Park, Saint Petersburg State University, Saint Petersburg, Russia). The crystal of **3d** was measured at a temperature of 100(2) K, using monochromated CuKα radiation. Crystallographic data for the structure **3d** (CCDC 2064882) have been deposited with the Cambridge Crystallographic Data Centre. Thin-layer chromatography (TLC) was conducted on aluminum sheets precoated with SiO_2_ ALUGRAM SIL G/UV254. Column chromatography was performed on Macherey-Nagel silica gel 60M (Research Park, Saint Petersburg State University, Saint Petersburg, Russia) (0.04–0.063 mm). 1,2-Dichloroethane was washed with concentrated H_2_SO_4_ and water, then with saturated aq. NaHCO_3_ and dried over CaCl_2_, then distilled from P_2_O_5_, then from CaH_2_, and stored over anhydrous K_2_CO_3_. Acetonitrile and propionitrile were distilled from P_2_O_5_, then distilled from anhydrous K_2_CO_3_, and stored over 3Å sieves. Other nitriles were recrystallized or distilled prior to use. 3-Aryl/heteryl-5-chloroisoxazoles **5** and diazoacetylazirines **1** were prepared according to the published procedures [28,30].

### 3.2. General Experimental Procedures

#### 3.2.1. General Procedure A (GP-A) for the Synthesis of *5-(2H-azirin-2-yl)oxazoles* **2**

Compound **2** was prepared following the slightly modified published procedure [27]. A portion of Rh_2_(oct)_4_ (1 mol%) was added to a mixture of azirine **1** (1 mmol) and appropriate nitrile (50–200 mmol) in DCE (500 mL per 1 mmol. of azirine). The resulting mixture was refluxed under argon for 10 min. The solvent was removed in vacuo, and the residue was purified by column chromatography on silica gel.

#### 3.2.2. General Procedure B (GP-B) for the Synthesis of *4H-pyrrolo[2,3-d]oxazoles* **3**

A solution of azirine **2** (0.1 mmol) in mesitylene (0.5 mL per 0.1 mmol of **2**) in a thick-wall tube with screw cap at 180 °C (bath temperature) for 1 h (TLC control).

#### 3.2.3. General Procedure C (GP-C) for the Synthesis of *4H-pyrrolo[2,3-d]oxazol-4-yl)(p-tolyl)methanones* **4**

A mixture of 4*H*-pyrrolo[2,3-*d*]oxazole **3** (0.1 mmol) and 15-crown-5 (0.1 mmol) in THF was added to a suspension of NaH (60% in mineral oil, 0.15 mmol, 1.5 eq.) in THF. After stirring for 0.5 h, a solution of 4-methylbenzoyl chloride (0.2 mmol) in THF was added dropwise to the resulting mixture. The reaction mixture was stirred for 3 h at room temperature, then poured into cold water and extracted with ethyl acetate. The combined organic phases were dried over Na_2_SO_4_, the solvent was removed in vacuo and the residue was purified by column chromatography on silica gel.

#### 3.2.4. Specific Procedures and Characterization

*2-Methyl-5-(3-phenyl-2H-azirin-2-yl)oxazole* (**2a**)**.** Compound **2a** [27] was prepared following the general procedure GP-A from azirine **1a** (463 mg, 2.5 mmol), acetonitrile (26 mL, 500 mmol) and Rh_2_(oct)_4_ (19.5 mg, 0.025 mmol) in DCE (250 mL) in 365 mg (73% yield, after column chromatography on silica (light petroleum/ethyl acetate, 4:1 (*v/v*)) as a light brown oil. ^1^H-NMR (CDCl_3_, 400 MHz): *δ* 2.36 (s, 3H), 3.25 (s, 1H), 6.84 (s, 1H), 7.56–7.62 (m, 2H), 7.63–7.67 (m, 1H), 7.90–7.93 (m, 2H).

*2-Methyl-5-(3-(4-bromophenyl)-2H-azirin-2-yl)oxazole* (**2b**). Compound **2b** was prepared following the general procedure GP-A from azirine **1b** (264 mg, 1 mmol), acetonitrile (10.4 g, 200 mmol) and Rh_2_(oct)_4_ (8 mg, 0.01 mmol) in DCE (150 mL) in 135 mg (49% yield, after column chromatography on silica (light petroleum/ethyl acetate, 7:1–4:1 (*v/v*)) as a brown oil. ^1^H-NMR (CDCl_3_, 400 MHz): *δ* 2.36 (s, 3H), 3.27 (s, 1H), 6.85 (s, 1H), 7.73–7.79 (m, 4H). ^13^C{^1^H}-NMR (CDCl_3_, 100 MHz): *δ* 13.9 (CH_3_), 25.3 (CH), 122.6 (C), 124.5 (CH), 128.6 (C), 131.1 (CH), 132.8 (CH), 150.7 (C), 160.9 (C), 162.1 (C). HRMS (ESI) *m/z*: [M + H]^+^ calcd. for C_12_H_10_BrN_2_O^+^ 276.9971; found 276.9974. IR (KBr, cm^−1^): ν 1571, 1743, 2925.

*5-(3-(Adamantan-1-yl)-2H-azirin-2-yl)-2-methyloxazole* (**2c**). Compound **2c** was prepared following the general procedure GP-A from azirine **1c** (100 mg, 0.41 mmol), acetonitrile (4.3 mL, 82 mmol) and Rh_2_(oct)_4_ (3 mg, 0.0041 mmol) in DCE (100 mL) in 63 mg (61% yield, after column chromatography on silica (light petroleum/ethyl acetate, 10:1 (*v/v*)) as a yellow oil. ^1^H-NMR (CDCl_3_, 400 MHz): *δ* 1.77–1.85 (m, 6H), 1.95–1.98 (m, 6H), 2.10–2.13 (m, 3H), 2.38 (s, 3H), 2.81 (s, 1H), 6.75 (s, 1H). ^13^C{^1^H}-NMR (CDCl_3_, 100 MHz): *δ* 13.9 (CH_3_), 24.0 (CH), 27.5 (CH), 35.6 (C), 36.4 (CH_2_), 38.2 (CH_2_), 123.6 (CH), 152.0 (C), 160.2 (C), 170.7 (C). HRMS (ESI) *m/z*: [M + Na]^+^ calcd. for C_16_H_20_N_2_NaO^+^ 279.1468; found 279.1458. IR (KBr, cm^−1^): ν 1574, 1697, 2851, 2907.

*2-Ethyl-5-(3-phenyl-2H-azirin-2-yl)oxazole* (**2d**). Compound **2d** was prepared following the general procedure GP-A from azirine **1a** (185 mg, 1 mmol), propionitrile (14.3 mL, 200 mmol) and Rh_2_(oct)_4_ (8 mg, 0.01 mmol) in DCE (200 mL) in 121 mg (57% yield, after column chromatography on silica (light petroleum/ethyl acetate, 11:1–7:1 (*v/v*)) as a light brown oil. ^1^H-NMR (CDCl_3_, 400 MHz): *δ* 1.26 (t, 3H, *J* = 7.6 Hz), 2.69 (q, 2H, *J* = 7.6 Hz), 3.26 (s, 1H), 6.83 (s, 1H), 7.56–7.66 (m, 3H), 7.90–7.93 (m, 2H). ^13^C{^1^H}-NMR (CDCl_3_, 100 MHz): *δ* 11.1 (CH_3_), 21.7 (CH_2_), 25.1 (CH), 123.8 (C), 124.1 (CH), 129.3 (C), 129.9 (CH), 133.6 (CH), 151.0 (C), 162.5 (C), 165.2 (C). HRMS (ESI) *m/z*: [M + H]^+^ calcd. for C_13_H_12_N_2_O^+^ 213.1022; found 213.1027. IR (KBr, cm^−1^): ν 1566, 1599, 1691, 1745, 2981.

*2-Ethyl-5-(3-(4-methoxyphenyl)-2H-azirin-2-yl)oxazole* (**2e**). Compound **2e** was prepared following the general procedure GP-A from azirine **1d** (215 mg, 1 mmol), propionitrile (14.3 mL, 200 mmol) and Rh_2_(oct)_4_ (8 mg, 0.01 mmol) in DCE (200 mL) in 117 mg (48% yield, after column chromatography on silica (light petroleum/ethyl acetate, 4:1 (*v/v*)) as a brown oil. ^1^H-NMR (CDCl_3_, 400 MHz): *δ* 1.26 (t, 3H, *J* = 7.6 Hz), 2.69 (q, 2H, *J* = 7.6 Hz), 3.19 (s, 1H), 3.91 (s, 3H), 6.80 (s, 1H), 7.06–7.09 (m, 2H), 7.84–7.87 (m, 2H). ^13^C{^1^H}-NMR (CDCl_3_, 100 MHz): *δ* 11.0 (CH_3_), 21.7 (CH_2_), 24.7 (CH), 55.6 (CH_3_), 114.9 (CH), 116.1 (C), 123.8 (CH), 131.9 (CH), 151.3 (C), 161.1 (C), 163.8 (C), 165.02 (C). HRMS (ESI) *m/z*: [M + H]^+^ calcd. for C_14_H_15_N_2_O_2_^+^ 243.1128; found 243.1131. IR (KBr, cm^−1^): ν 1509, 1567, 1605, 1679, 1745, 2939, 2980.

*5-(3-(tert-Butyl)-2H-azirin-2-yl)-2-ethyloxazole* (**2f**). Compound **2f** was prepared following the general procedure GP-A from azirine **1e** (150 mg, 0.91 mmol), propionitrile (9.7 mL, 136 mmol) and Rh_2_(oct)_4_ (7 mg, 0.0091 mmol) in DCE (150 mL) in 67 mg (38% yield, after column chromatography on silica (light petroleum/ethyl acetate, 6:1 (*v/v*)) as an orange oil. ^1^H-NMR (CDCl_3_, 400 MHz): *δ* 1.27 (t, 3H, *J* = 7.6 Hz), 1.34 (s, 9H), 2.70 (q, 2H, *J* = 7.6 Hz), 2.90 (s, 1H), 6.80 (s, 1H). ^13^C{^1^H}-NMR (CDCl_3_, 100 MHz): *δ* 11.0 (CH3), 21.6 (CH2), 24.5 (CH), 26.0 (CH3), 33.3 (C), 123.6 (CH), 151.4 (C), 164.7 (C), 171.8 (C). HRMS (ESI) *m/z*: [M + H]^+^ calcd. for C_11_H_17_N_2_O^+^ 193.1335; found 193.1328. IR (KBr, cm^−1^): ν 1568, 1700, 2971.

*2-Benzyl-5-(3-(4-bromophenyl)-2H-azirin-2-yl)oxazole* (**2g**). Compound **2g** was prepared following the general procedure GP-A from azirine **1d** (200 mg, 0.76 mmol), benzyl cyanide (12.9 g, 110 mmol) and Rh_2_(oct)_4_ (6 mg, 0.0076 mmol) in DCE (200 mL) in 94 mg (35% yield, after column chromatography on silica (toluene/light petroleum/ethyl acetate, 20:1 + 0.5% triethylamine (*v/v*)) as a brown oil. ^1^H-NMR (CDCl_3_, 400 MHz): *δ* 3.30 (s, 1H), 4.06 (s, 2H), 6.89 (s, 1H), 7.26–7.35 (m, 5H), 7.75–7.81 (m, 4H). ^13^C{^1^H}-NMR (CDCl_3_, 100 MHz): *δ* 25.3 (CH), 34.6 (CH_2_), 122.6 (C), 124.5 (CH), 127.1 (CH), 128.7 (CH), 128.8 (C), 131.1 (CH), 132.8 (CH), 135.3 (C), 151.3 (C), 161.9 (C), 161.9 (C), 162.5 (C). HRMS (ESI) *m/z*: [M + H]^+^ calcd. for C_18_H_14_BrN_2_O^+^ 353.0284; found 353.0288. IR (KBr, cm^−1^): ν 1670, 2924.

*2-Phenyl-5-(3-phenyl-2H-azirin-2-yl)oxazole* (**2h**). Compound **2h** [27] was prepared following the general procedure GP-A from azirine **1a** (500 mg, 2.7 mmol), benzonitrile (29 mL, 284 mmol) and Rh_2_(oct)_4_ (2.1 mg, 0.027 mmol) in DCE (250 mL) in 302 mg (43% yield, after column chromatography on silica (light petroleum/ethyl acetate, 7:1 (*v/v*)) as a brown oil. ^1^H-NMR (CDCl_3_, 400 MHz): *δ* 3.37 (s, 1H), 7.06 (s, 1H), 7.38–7.42 (m, 3H), 7.59–7.67 (m, 3H), 7.92–7.98 (m, 4H).

*5-(3-(4-Fluorophenyl)-2H-azirin-2-yl)-2-phenyloxazole* (**2i**). Compound **2i** was prepared following the general procedure GP-A from azirine **1f** (102 mg, 0.5 mmol), benzonitrile (10.4 mL, 100 mmol) and Rh_2_(oct)_4_ (4 mg, 0.005 mmol) in DCE (200 mL) in 78 mg (56% yield, after column chromatography on silica (light petroleum/ethyl acetate, 1:0–11:1–6:1 (*v/v*)) as a brown oil. ^1^H-NMR (CDCl_3_, 400 MHz): *δ* 3.38 (s, 1H), 7.07 (s, 1H), 7.28–7.33 (m, 2H), 7.38–7.42 (m, 3H), 7.91–7.94 (m, 2H), 7.96–8.00 (m, 2H). ^13^C{^1^H}-NMR (CDCl_3_, 100 MHz): *δ* 25.4 (CH), 117.0 (d, CH, *J* = 22.4 Hz,), 119.9 (d, C, *J* = 3.2 Hz,), 125.8 (CH), 126.2 (CH), 127.3 (C), 128.7 (CH), 130.2 (CH), 132.3 (d, CH, *J* = 9.3 Hz), 136.0 (CH), 151.4 (C), 161.3 (C), 165.9 (d, C, *J* = 256.6 Hz). HRMS (ESI) *m/z*: [M + H]^+^ calcd. for C_17_H_12_FN_2_O^+^ 279.0928; found 279.0925. IR (KBr, cm^−1^): ν 1505, 1543, 1600, 1745, 3059.

*5-(3-(tert-Butyl)-2H-azirin-2-yl)-2-phenyloxazole* (**2j**). Compound **2j** was prepared following the general procedure GP-A from azirine **1e** (150 mg, 0.91 mmol), benzonitrile (14 mL, 137 mmol) and Rh_2_(oct)_4_ (7 mg, 0.0091 mmol) in DCE (150 mL) in 111 mg (51% yield, after column chromatography on silica (light petroleum/ethyl acetate, 9:1 (*v/v*)) as an orange oil. ^1^H-NMR (CDCl_3_, 400 MHz): *δ* 1.40 (s, 9H), 3.02 (s, 1H), 7.06 (s, 1H), 7.41–7.43 (m, 3H), 7.93–7.96 (m, 2H). ^13^C{^1^H}-NMR (CDCl_3_, 100 MHz): *δ* 25.6 (CH), 26.1 (CH_3_), 33.6 (C), 125.5 (CH), 126.1 (CH), 127.4 (C), 128.7 (CH), 130.1 (CH), 152.1 (C), 160.6 (C), 171.6 (C). HRMS (ESI) *m/z*: [M + Na]^+^ calcd. for C_15_H_16_N_2_NaO^+^ 263.1155; found 263.1144. IR (KBr, cm^−1^): ν 1547, 1590, 2933, 2970.

*5-(3-(Adamantan-1-yl)-2H-azirin-2-yl)-2-phenyloxazole* (**2k**). Compound **2k** was prepared following the general procedure GP-A from azirine **1c** (150 mg, 0.62 mmol), benzonitrile (9.5 mL, 93 mmol) and Rh_2_(oct)_4_ (5 mg, 0.0062 mmol) in DCE (130 mL) in 84 mg (43% yield, after column chromatography on silica (light petroleum/ethyl acetate, 7:1 (*v/v*)) as a yellow oil. ^1^H-NMR (CDCl_3_, 400 MHz): *δ* 1.79–1.87 (m, 6H), 1.99–2.07 (m, 6H), 2.12–2.16 (m, 3H), 2.93 (s, 1H), 7.01 (s, 1H), 7.41–7.45 (m, 3H), 7.93–7.96 (m, 2H). ^13^C{^1^H}-NMR (CDCl_3_, 100 MHz): *δ* 24.1 (CH), 27.5 (CH), 35.7 (C), 36.4 (CH_2_), 38.3 (CH_2_), 125.2 (CH), 126.1 (CH), 127.5 (C), 128.8 (CH), 130.1 (CH), 152.4 (C), 160.5 (C), 170.5 (C). HRMS (ESI) *m/z*: [M + Na]^+^ calcd. for C_21_H_22_N_2_NaO^+^ 341.1624; found 341.1621. IR (KBr, cm^−1^): ν 1580, 1756, 2855, 2911.

*5-(3-(4-Methoxyphenyl)-2H-azirin-2-yl)-2-(p-tolyl)oxazole* (**2l**). Compound **2l** was prepared following the general procedure GP-A from azirine **1d** (200 mg, 0.93 mmol), *p*-toluonitrile (7 g, 60 mmol) and Rh_2_(oct)_4_ (7 mg, 0.0093 mmol) in DCE (200 mL) in 58 mg (27% yield, after column chromatography on silica (light petroleum/ethyl acetate, 1:0–8:1–4:1 (*v/v*)) as a brown oil. ^1^H-NMR (CDCl_3_, 400 MHz): *δ* 2.37 (s, 3H), 3.31 (s, 1H), 3.92 (s, 3H), 7.02 (s, 1H), 7.08–7.10 (m, 2H), 7.19–7.21 (m, 2H), 7.82–7.84 (m, 2H), 7.88–7.92 (m, 2H). ^13^C{^1^H}-NMR (CDCl_3_, 100 MHz): *δ* 21.4 (CH_3_), 24.9 (CH), 55.6 (CH_3_), 114.9 (CH), 115.9 (C), 124.7 (C), 125.4 (CH), 126.2 (CH), 129.3 (CH), 132.0 (CH), 140.4 (C), 151.7 (C), 160.9 (C), 161.2 (C), 163.9 (C). HRMS (ESI) *m/z*: [M + H]^+^ calcd. for C_19_H_17_N_2_O_2_^+^ 305.1285; found 305.1296. IR (KBr, cm^−1^): ν 1509, 1605, 1676, 1724, 2853, 2924.

*5-(3-(4-Chlorophenyl)-2H-azirin-2-yl)-2-(p-tolyl)oxazole* (**2m**). Compound **2m** was prepared following the general procedure GP-A from azirine **1g** (200 mg, 0.91 mmol), *p*-toluonitrile (10.6 g, 91 mmol) and Rh_2_(oct)_4_ (7 mg, 0.0091 mmol) in DCE (150 mL) in 95 mg (34% yield, after column chromatography on silica (light petroleum/ethyl acetate, 1:0–7:1 (*v/v*)) as a brown oil. ^1^H-NMR (CDCl_3_, 400 MHz): *δ* 2.37 (s, 3H), 3.37 (s, 1H), 7.04 (s, 1H), 7.19–7.21 (m, 2H), 7.58–7.60 (m, 2H), 7.79–7.81 (m, 2H), 7.89–7.91 (m, 2H). ^13^C{^1^H}-NMR (CDCl_3_, 100 MHz): *δ* 21.5 (CH_3_), 25.5 (CH), 122.1 (C), 124.6 (C), 125.8 (CH), 126.2 (CH), 129.4 (CH), 129.9 (CH), 131.1 (CH), 140.1 (C), 140.6 (C), 150.9 (C), 161.3 (C), 161.8 (C). HRMS (ESI) *m/z*: [M + H]^+^ calcd. for C_18_H_14_ClN_2_O_2_^+^ 309.0789; found 309.0792. IR (KBr, cm^−1^): ν 1590, 1675, 1741, 2924.

*5-(3-(Adamantan-1-yl)-2H-azirin-2-yl)-2-(p-tolyl)oxazole* (**2n**). Compound **2n** was prepared following the general procedure GP-A from azirine **1c** (100 mg, 0.41 mmol), *p*-toluonitrile (5.2 g, 41 mmol) and Rh_2_(oct)_4_ (3 mg, 0.0041 mmol) in DCE (100 mL) in 63 mg (46% yield, after column chromatography on silica (light petroleum/ethyl acetate, 1:0–10:1 (*v/v*)) as a yellow oil. ^1^H-NMR (CDCl_3_, 400 MHz): *δ* 1.79–1.87 (m, 6H), 1.98–2.07 (m, 6H), 2.12–2.15 (m, 3H), 2.39 (s, 3H), 2.92 (s, 1H), 6.99 (s, 1H), 7.23–7.25 (m, 2H), 7.82–7.84 (m, 2H). ^13^C{^1^H}-NMR (CDCl_3_, 100 MHz): *δ* 21.5 (CH_3_), 24.2 (CH), 27.5 (CH), 35.7 (C), 36.4 (CH_2_), 38.3 (CH_2_), 124.8 (C), 125.1 (CH), 126.0 (CH), 129.5 (CH), 140.4 (C), 152.1 (C), 160.7 (C), 170.6 (C). HRMS (ESI) *m/z*: [M + H]^+^ calcd. for C_22_H_25_N_2_O^+^ 333.1961; found 333.1964. IR (KBr, cm^−1^): ν 1590, 1758, 2856, 2903.

*2-(4-Bromophenyl)-5-(3-phenyl-2H-azirin-2-yl)oxazole* (**2o**). Compound **2o** was prepared following the general procedure GP-A from azirine **1a** (185 mg, 1 mmol), 4-bromobenzonitrile (25 g, 200 mmol) and Rh_2_(oct)_4_ (8 mg, 0.01 mmol) in DCE (200 mL) in 51 mg (15% yield, after column chromatographies on silica (toluene/light petroleum/ethyl acetate, 100:0:0–100:1:1–0:12:1 (*v/v*); hexanes/methyl acetate, 20:1 (*v/v*) + 0.5% NEt_3_) as a brown oil. The low yield of compound **2o** is associated with its significant losses during chromatographic isolation due to the low solubility of the starting 4-bromobenzonitrile, which is used in a large excess in the reaction. ^1^H-NMR (CDCl_3_, 400 MHz): *δ* 3.36 (s, 1H), 7.05 (s, 1H), 7.52–7.55 (m, 2H), 7.59–7.63 (m, 2H), 7.65–7.69 (m, 1H), 7.77–7.81 (m, 2H), 7.94–7.971 (m, 2H). ^13^C{^1^H}-NMR (CDCl_3_, 100 MHz): *δ* 25.2 (CH), 123.4 (C), 124.6 (C), 125.8 (CH), 126.3 (C), 127.7 (CH), 129.4 (CH), 130.0 (CH), 132.0 (CH), 133.8 (CH), 152.02 (C), 160.1 (C), 162.2 (C). HRMS (ESI) *m/z*: [M + H]^+^ calcd. for C_17_H_12_N_2_O^+^ 339.0128; found 339.0128. IR (KBr, cm^−1^): ν 1599, 1675, 1728, 1741.

*2-(4-Bromophenyl)-5-(3-(tert-butyl)-2H-azirin-2-yl)oxazole* (**2p**). Compound **2p** was prepared following the general procedure GP-A from azirine **1e** (150 mg, 0.91 mmol), 4-bromobenzonitrile (14 g, 77 mmol) and Rh_2_(oct)_4_ (7 mg, 0.0091 mmol) in DCE (150 mL) in 89 mg (31% yield, after column chromatography on silica (light petroleum/ethyl acetate, 9:1–8:1 (*v/v*)) as a red oil. ^1^H-NMR (CDCl_3_, 400 MHz): *δ* 1.39 (s, 9H), 3.00 (s, 1H), 7.04 (s, 1H), 7.55–7.57 (m, 2H), 7.78-7.80 (m, 2H). ^13^C{^1^H}-NMR (CDCl_3_, 100 MHz): *δ* 25.5 (CH), 26.1 (CH_3_), 33.6 (C), 124.6 (C), 125.5 (CH), 126.3 (C), 127.5 (CH), 132.0 (CH), 152.5 (C), 159.7 (C), 171.4 (C). HRMS (ESI) *m/z*: [M + Na]^+^ calcd. for C_15_H_15_BrN_2_NaO^+^ 341.0260; found 341.0251. IR (KBr, cm^−1^): ν 1573, 1754, 2971.

*(E)-3-(5-(3-phenyl-2H-azirin-2-yl)oxazol-2-yl)acrylonitrile* (**2q**). Compound **2q** was prepared following the general procedure GP-A from azirine **1a** (100 mg, 0.54 mmol), fumaronitrile (4.2 g, 54 mmol) and Rh_2_(oct)_4_ (4 mg, 0.0054 mmol) in DCE (150 mL) in 38 mg (30% yield, after column chromatography on silica (light petroleum/ethyl acetate, 1:0–30:1 (*v/v*)) as a yellow oil. ^1^H-NMR (CDCl_3_, 400 MHz): *δ* 3.30 (s, 1H), 6.10 (d, 1H, *J* = 16.5 Hz), 7.08 (d, 1H, *J* = 16.5 Hz), 7.13 (s, 1H), 7.59–7.63 (m, 2H), 7.66–7.70 (m, 1H), 7.90–7.93 (m, 2H). ^13^C{^1^H}-NMR (CDCl_3_, 100 MHz): *δ* 24.9 (CH), 102.4 (CH), 116.5 (C), 122.8 (C), 127. (CH), 129.5 (CH), 130.0 (CH), 133.8 (CH), 134.0 (CH), 154.2 (C), 157.0 (C), 161.3 (C). HRMS (ESI) *m/z*: [M + Na]^+^ calcd. for C_14_H_9_N_3_NaO^+^ 258.0638; found 258.0638. IR (KBr, cm^−1^): ν 1516, 1746, 2217, 2854, 2924, 3062.

*5-(3-Phenyl-2H-azirin-2-yl)-2-vinyloxazole* (**2r**). Compound **2r** was prepared following the general procedure GP-A from azirine **1a** (222 mg, 1.2 mmol), acrylonitrile (15.7 mL, 240 mmol) and Rh_2_(oct)_4_ (10 mg, 0.012 mmol) in DCE (200 mL) in 106 mg (38% yield, after column chromatography on silica (light petroleum/ethyl acetate, 5:1 (*v/v*)) as a yellow oil. ^1^H-NMR (CDCl_3_, 400 MHz): *δ* 3.30 (s, 1H), 5.53 (dd, 1H, *J* = 11.3, 1.1 Hz), 6.04 (dd, 1H, *J* = 17.7, 1.1 Hz), 6.51 (dd, 1H, *J* = 17.7, 11.3 Hz), 6.97 (s, 1H), 7.58–7.67 (m, 3H), 7.92–7.95 (m, 2H). ^13^C{^1^H}-NMR (CDCl_3_, 100 MHz): *δ* 25.1 (CH), 121.4 (CH_2_), 123.2 (CH), 123.5 (C), 125.4 (CH), 129.4 (CH), 129.9 (CH), 133.7 (CH), 151.4 (C), 160.4 (C), 162.2 (C). HRMS (ESI) *m/z*: [M + Na]^+^ calcd. for C_13_H_10_N_2_NaO^+^ 233.0685; found 233.0677. IR (KBr, cm^−1^): ν 1519, 1597, 1696, 1745, 3061.

*2-(Chloromethyl)-5-(3-phenyl-2H-azirin-2-yl)oxazole* (**2s**). Compound **2s** was prepared following the general procedure GP-A from azirine **1a** (200 mg, 1.1 mmol), chloroacetonitrile (13.8 mL, 220 mmol) and Rh_2_(oct)_4_ (8 mg, 0.01 mmol) in DCE (150 mL) in 67 mg (26% yield, after column chromatography on silica (light petroleum/ethyl acetate, 5:1 (*v/v*)) as a yellow oil. ^1^H-NMR (CDCl_3_, 400 MHz): *δ* 3.29 (s, 1H), 4.53 (d, 2H, *J* = 0.8 Hz), 6.93 (s, 1H), 7.58–7.63 (m, 2H), 7.65–7.69 (m, 1H), 7.91–7.94 (m, 2H). ^13^C{^1^H}-NMR (CDCl_3_, 100 MHz): *δ* 24.9 (CH), 35.8 (CH_2_), 123.3 (C), 124.8 (CH), 129.4 (CH), 130.0 (CH), 133.8 (CH), 153.3 (C), 158.2 (C), 161.8 (C). HRMS (ESI) *m/z*: [M + H]^+^ calcd. for C_12_H_10_ClN_2_O^+^ 213.0476; found 233.0476. IR (KBr, cm^−1^): ν 1526, 1598, 1689, 2854, 2926, 3035, 3143.

*2-Methyl-5-phenyl-4H-pyrrolo[2,3-d]oxazole* (**3a**). Compound **3a** was prepared following the general procedure GP-B from azirine **2a** (100 mg, 0.5 mmol) in mesitylene (1.0 mL) in 74 mg (74% yield, after column chromatography on silica (chloroform/methanol, 0:1–100:1 (*v/v*)) as a light brown solid: mp 194–195 °C (chloroform). ^1^H-NMR (DMSO-*d*_6_, 400 MHz): *δ* 2.52 (s, 3H), 6.60 (d, 1H, *J* = 1.7 Hz), 7.15–7.19 (m, 1H), 7.33–7.37 (m, 2H), 7.64–7.67 (m, 2H), 11.60 (s, 1H). ^13^C{^1^H}-NMR (DMSO-*d*_6_, 100 MHz): *δ* 14.8 (CH_3_), 87.7 (CH), 123.4 (CH), 125.9 (CH), 128.7 (CH), 131.1 (C), 133.4 (C), 139.0 (C), 140.9 (C), 162.6 (C). HRMS (ESI) *m/z*: [M + H]^+^ calcd. for C_12_H_11_N_2_O^+^ 199.0866; found 199.0873. IR (KBr, cm^−1^): ν 1509, 1556, 1606, 3167, 3205.

*2-Methyl-5-(4-bromophenyl)-4H-pyrrolo[2,3-d]oxazole* (**3b**). Compound **3b** was prepared following the general procedure GP-B from azirine **2b** (98 mg, 0.35 mmol) in mesitylene (1.5 mL) in 52 mg (53% yield, after evaporation of solvent and washing with cold ether) as a brown solid: mp 274–276 °C (mesitylene). ^1^H-NMR (DMSO-*d*_6_, 400 MHz): *δ* 2.52 (s, 3H), 6.66 (d, *J* = 1.6 Hz, 1H), 7.52–7.55 (m, 2H), 7.60–7.63 (m, 2H), 11.68 (s, 1H). ^13^C{^1^H}-NMR (DMSO-*d*_6_, 100 MHz): *δ* 14.8 (CH_3_), 88.3 (CH), 118.4 (C), 125.3 (CH), 129.8 (C), 131.6 (CH), 132.7 (C), 139.4 (C), 140.9 (C), 162.0 (C). HRMS (ESI) *m/z*: [M + H]^+^ calcd. for C_12_H_10_BrN_2_O^+^ 276.9971; found 276.9965. IR (KBr, cm^−1^): ν 1558, 3131, 3214.

*5-(Adamantan-1-yl)-2-methyl-4H-pyrrolo[2,3-d]oxazole* (**3c**). Compound **3c** was prepared following the general procedure GP-B from azirine **2c** (56 mg, 0.22 mmol) in mesitylene (1.0 mL) in 35 mg (70% yield, after column chromatography on silica (light petroleum/ethyl acetate, 7:1 (*v/v*)) as a light brown solid: mp 204–206 °C (light petroleum/ethyl acetate). ^1^H-NMR (CDCl_3_, 400 MHz): *δ* 1.73–1.86 (m, 6H), 1.94–1.95 (m, 6H), 2.07–2.09 (m, 3H), 2.55 (s, 3H), 5.83 (d, 1H, *J* = 1.8 Hz), 8.96 (s, 1H). ^13^C{^1^H}-NMR (CDCl_3_, 100 MHz): *δ* 15.0 (CH_3_), 27.5 (CH), 28.5 (CH), 34.2 (C), 36.7 (CH_2_), 43.0 (CH_2_), 85.6 (CH), 136.1 (C), 140.7 (C), 143.0 (C), 160.5 (C). HRMS (ESI) *m/z*: [M + H]^+^ calcd. for C_16_H_21_N_2_O^+^ 257.1648; found 257.1647. IR (KBr, cm^−1^): ν 1552, 1663, 1714, 2847, 2905, 3211.

*2-Ethyl-5-phenyl-4H-pyrrolo[2,3-d]oxazole* (**3d**). Compound **3d** was prepared following the general procedure GP-B from azirine **2d** (75 mg, 0.35 mmol) in mesitylene (1.0 mL) in 52 mg (69% yield, after column chromatography on silica (toluene/chloroform, 100:1 (*v/v*)) as a light brown solid: mp 155–157 °C (toluene). ^1^H-NMR (CDCl_3_, 400 MHz): *δ* 1.43 (t, 3H, *J* = 7.6 Hz), 2.97 (q, 2H, *J* = 7.6 Hz), 6.45 (d, 1H, *J* = 1.6 Hz), 7.21-7.26 (s, 1H), 7.37–7.40 (m, 2H), 7.52–7.55 (m, 2H), 9.26 (s, 1H). ^13^C{^1^H}-NMR (CDCl_3_, 100 MHz): *δ* 11.8 (CH_3_), 23.1 (CH_2_), 88.4 (CH), 124.0 (CH), 126.5 (CH), 128.9 (CH), 132.2 (C), 133.6 (C), 138.7 (C), 141.9 (C), 166.74 (C). HRMS (ESI) *m/z*: [M + H]^+^ calcd. for C_13_H_13_N_2_O^+^ 213.1022; found 213.1015. IR (KBr, cm^−1^): ν 1508, 1551, 1604, 3204.

*2-Ethyl-5-(4-methoxyphenyl)-4H-pyrrolo[2,3-d]oxazole* (**3e**). Compound **3e** was prepared following the general procedure GP-B from azirine **2e** (82 mg, 0.34 mmol) in mesitylene (1.5 mL) in 40 mg (49% yield, after column chromatography on silica (light petroleum/ethyl acetate, 5:1 + 5% chloroform (*v/v*)) as a brown solid: mp 214–216 °C (light petroleum/ethyl acetate). ^1^H-NMR (DMSO-*d*_6_, 400 MHz): *δ* 1.40 (t, 3H, *J* = 7.6 Hz), 2.92 (q, 2H, *J* = 7.6 Hz), 3.84 (s, 3H), 6.30 (d, 1H, *J* = 1.6 Hz), 6.92–6.95 (m, 2H), 7.44–7.47 (m, 2H), 9.03 (s, 1H). ^13^C{^1^H}-NMR (DMSO-*d*_6_, 100 MHz): *δ* 11.9 (CH_3_), 22.6 (CH_2_), 55.6 (CH_3_), 87.1 (CH), 114.7 (CH), 125.4 (CH), 126.7 (C), 131.8 (C), 138.7 (C), 141.3 (C), 158.2 (C), 165.8 (C). HRMS (ESI) *m/z*: [M + H]^+^ calcd. for C_14_H_15_N_2_O_2_^+^ 243.1128; found 243.1122. IR (KBr, cm^−1^): ν 1517, 1551, 1613, 2959, 3233.

*5-(tert-Butyl)-2-ethyl-4H-pyrrolo[2,3-d]oxazole* (**3f**). Compound **3f** was prepared following the general procedure GP-B from azirine **2f** (59 mg, 0.31 mmol) in mesitylene (1.5 mL) in 39 mg (67% yield, after column chromatography on silica (light petroleum/ethyl acetate, 7:1 (*v/v*)) as a light brown solid: mp 151–154 °C (light petroleum/ethyl acetate). ^1^H-NMR (CDCl_3_, 400 MHz): *δ* 1.35 (s, 9H), 1.37 (t, 3H, *J* = 7.5 Hz), 2.87 (q, 2H, *J* = 7.5 Hz,), 5.86 (d, 1H, *J* = 1.7 Hz), 8.82 (s, 1H). ^13^C{^1^H}-NMR (CDCl_3_, 100 MHz): *δ* 11.8 (CH_3_), 22.8 (CH_2_), 30.6 (CH_3_), 32.4 (C), 86.0 (CH), 136.4 (C), 140.4 (C), 142.4 (C), 165.3 (C). HRMS (ESI) *m/z*: [M + H]^+^ calcd. for C_11_H_17_N_2_O^+^ 193.1335; found 193.1333. IR (KBr, cm^−1^): ν 1525, 1550, 1584, 2867, 2963, 3236.

*2-Benzyl-5-(4-bromophenyl)-4H-pyrrolo[2,3-d]oxazole* (**3g**). Compound **3g** was prepared following the general procedure GP-B from azirine **2g** (94 mg, 0.27 mmol) in mesitylene (1.5 mL) in 56 mg (60% yield, after column chromatography on silica (light petroleum/ethyl acetate, 4:1 + 5% chloroform (*v/v*)) as a brown solid: mp 227–230 °C (light petroleum/ethyl acetate). ^1^H-NMR (DMSO-*d*_6_, 400 MHz): *δ* 4.22 (s, 2H), 6.66 (d, 1H, *J* = 1.7 Hz), 7.26–7.29 (m, 1H), 7.32–7.37 (m, 4H), 7.53–7.56 (m, 2H), 7.60–7.63 (m, 2H), 11.72 (s, 1H). ^13^C{^1^H}-NMR (DMSO-*d*_6_, 100 MHz): *δ* 34.9 (CH_2_), 88.4 (CH), 118.6 (C), 125.4 (CH), 126.8 (CH), 128.6 (CH), 128.7 (CH), 130.3 (C), 131.6 (CH), 132.5 (C), 136.1 (C), 139.3 (C), 141.2 (C), 163.5 (C). HRMS (ESI) *m/z*: [M + H]^+^ calcd. for C_18_H_14_BrN_2_O^+^ 353.0284; found 353.0284. IR (KBr, cm^−1^): ν 1506, 3210.

*2,5-Diphenyl-4H-pyrrolo[2,3-d]oxazole* (**3h**). Compound **3h** was prepared following the general procedure GP-B from azirine **2h** (85 mg, 0.33 mmol) in mesitylene (1.0 mL) in 64 mg (75% yield, after column chromatography on silica (toluene/chloroform, 100:1 (*v/v*)) as a light brown solid: mp 246–247 °C (toluene). ^1^H-NMR (DMSO-*d*_6_, 400 MHz): *δ* 6.75 (d, 1H, *J* = 1.6 Hz), 7.21–7.25 (m, 1H), 7.38–7.42 (m, 2H), 7.48–7.50 (m, 1H), 7.51–7.56 (m, 2H), 7.72–7.74 (m, 2H), 8.01–8.04 (m, 2H), 11.86 (s, 1H). ^13^C{^1^H}-NMR (DMSO-*d*_6_, 100 MHz): *δ* 87.9 (CH), 123.8 (CH), 125.3 (CH), 126.4 (CH), 128.1 (C), 128.8 (CH), 129.1 (CH), 129.8 (CH), 133.0 (C), 133.4 (C), 140.3 (C), 141.7 (C), 160.8 (C). HRMS (ESI) *m/z*: [M + H]^+^ calcd. for C_17_H_13_N_2_O^+^ 261.1022; found 261.1015. IR (KBr, cm^−1^): ν 1467, 1600, 3256.

*5-(4-Fluorophenyl)-2-phenyl-4H-pyrrolo[2,3-d]oxazole* (**3i**). Compound **3i** was prepared following the general procedure GP-B from azirine **2i** (120 mg, 0.43 mmol) in mesitylene (1.5 mL) in 43 mg (36% yield, after evaporation of solvent and washing with acetonitrile) as a brown solid: mp 248–251 °C (mesitylene). ^1^H-NMR (DMSO-*d*_6_, 400 MHz): *δ* 6.72 (d, 1H, *J* = 1.6 Hz), 7.23–7.28 (m, 2H), 7.46–7.50 (m, 1H), 7.51–7.56 (m, 2H), 7.74–7.77 (m, 2H), 8.00–8.03 (m, 2H), 11.86 (s, 1H). ^13^C{^1^H}-NMR (DMSO-*d*_6_, 100 MHz): *δ* 88.5 (CH), 116.2 (d, CH, *J* = 21.6 Hz), 125.8 (CH), 126.2 (d, CH, *J* = 7.8 Hz), 128.5 (C), 129.6 (CH), 130.2 (d, C, *J* = 3.2 Hz), 130.3 (CH), 132.9 (C), 140.7 (C), 142.1 (C), 161.2 (C), 161.4 (d, C, *J* = 243.7 Hz). HRMS (ESI) *m/z*: [M + H]^+^ calcd. for C_17_H_12_FN_2_O^+^ 279.0928; found 279.0932. IR (KBr, cm^−1^): ν 1513, 1563, 3252.

*5-(tert-Butyl)-2-phenyl-4H-pyrrolo[2,3-d]oxazole* (**3j**). Compound **3j** was prepared following the general procedure GP-B from azirine **2j** (87 mg, 0.36 mmol) in mesitylene (1.5 mL) in 67 mg (77% yield, after column chromatography on silica (light petroleum/ethyl acetate, 9:1 (*v/v*)) as a light brown solid: mp 119–121 °C (light petroleum/ethyl acetate). ^1^H-NMR (CDCl_3_, 400 MHz): *δ* 1.35 (s, 9H), 5.96 (d, 1H, *J* = 1.7 Hz), 7.35–7.39 (m, 1H), 7.41–7.46 (m, 2H), 8.03–8.05 (m, 2H), 8.19 (s, 1H). ^13^C{^1^H}-NMR (CDCl_3_, 100 MHz): *δ* 30.5 (CH_3_), 32.6 (C), 86.5 (CH), 125.7 (CH), 128.7 (CH), 129.0 (C), 129.2 (CH), 138.0 (C), 141.2 (C), 144.2 (C), 160.8 (C). HRMS (ESI) *m/z*: [M + H]^+^ calcd. for C_15_H_17_N_2_O^+^ 241.1335; found 241.1337. IR (KBr, cm^−1^): ν 1541, 1571, 1605, 2963, 3134, 3215.

*5-(Adamantan-1-yl)-2-phenyl-4H-pyrrolo[2,3-d]oxazole* (**3k**). Compound **3k** was prepared following the general procedure GP-B from azirine **2k** (50 mg, 0.15 mmol) in mesitylene (1.0 mL) in 31 mg (62% yield, after column chromatography on silica (light petroleum/ethyl acetate, 10:1 (*v/v*)) as a light grey solid: mp 205–207 °C (light petroleum/ethyl acetate). ^1^H-NMR (CDCl_3_, 400 MHz): *δ* 1.69–1.79 (m, 6H), 1.92–1.93 (m, 6H), 2.04–2.06 (m, 3H), 5.93 (d, 1H, *J* = 1.7 Hz), 7.35–7.39 (m, 1H), 7.41–7.45 (m, 2H), 8.03–8.06 (m, 2H), 8.34 (s, 1H). ^13^C{^1^H}-NMR (CDCl_3_, 100 MHz): *δ* 28.5 (CH), 34.4 (C), 36.6 (CH_2_), 42.8 (CH_2_), 85.9 (CH), 125.7 (CH), 128.7 (CH), 129.0 (C), 129.2 (CH), 137.7 (C), 141.3 (C), 144.9 (C), 160.7 (C). HRMS (ESI) *m/z*: [M + Na]^+^ calcd. for C_21_H_22_N_2_NaO^+^ 341.1624; found 341.1627. IR (KBr, cm^−1^): ν 1569, 2848, 2900, 3264.

*5-(4-Methoxyphenyl)-2-(p-tolyl)-4H-pyrrolo[2,3-d]oxazole* (**3l**). Compound **3l** was prepared following the general procedure GP-B from azirine **2l** (58 mg, 0.19 mmol) in mesitylene (1.0 mL) in 18 mg (31% yield, after column chromatography on silica (light petroleum/ethyl acetate, 1:0–4:1 (*v/v*)) as a light brown solid: mp 217–219 °C (light petroleum/ethyl acetate). ^1^H-NMR (DMSO-*d*_6_, 400 MHz): *δ* 2.37 (s, 3H), 3.78 (s, 3H), 6.58 (d, 1H, *J* = 1.6 Hz), 6.96–6.98 (m, 2H), 7.32–7.34 (m, 2H), 7.63–6.65 (m, 2H), 7.87–7.89 (m, 2H), 11.65 (s, 1H). ^13^C{^1^H}-NMR (DMSO-*d*_6_, 400 MHz): *δ* 21.5 (CH_3_), 55.6 (CH_3_), 87.3 (CH), 114.8 (CH), 125.6 (CH), 125.7 (CH), 126.0 (C), 126.3 (C), 130.2 (CH), 133.7 (C), 139.9 (C), 140.1 (C), 142.0 (C), 158.5 (C), 161.0 (C). HRMS (ESI) *m/z*: [M + H]^+^ calcd. for C_19_H_17_N_2_O_2_^+^ 305.1285; found 305.1296. IR (KBr, cm^−1^): ν 1604, 1661, 3252.

*5-(4-Chlorophenyl)-2-(p-tolyl)-4H-pyrrolo[2,3-d]oxazole* (**3m**). Compound **3m** was prepared following the general procedure GP-B from azirine **2m** (76 mg, 0.25 mmol) in mesitylene (1.5 mL) in 47 mg (61% yield, after evaporation of solvent and washing with cold ether) as a light brown solid: mp 237–239 °C (mesitylene). ^1^H-NMR (DMSO-*d*_6_, 400 MHz): *δ* 2.38 (s, 3H), 6.78 (d, 1H, *J* = 1.5 Hz), 7.34–7.36 (m, 2H), 7.44–7.46 (m, 2H), 7.72–7.74 (m, 2H), 7.90–7.92 (m, 2H), 11.89 (s, 1H). ^13^C{^1^H}-NMR (DMSO-*d*_6_, 400 MHz): *δ* 21.5 (CH_3_), 89.0 (CH), 125.7 (CH), 125.8 (C), 125.9 (CH), 129.3 (CH), 130.2 (CH), 131.0 (C), 132.2 (C), 132.5 (C), 140.3 (C), 141.0 (C), 141.9 (C), 161.9 (C). HRMS (ESI) *m/z*: [M + H]^+^ calcd. for C_18_H_14_ClN_2_O^+^ 309.0789; found 309.0777. IR (KBr, cm^−1^): ν 1551, 3247.

*5-(Adamantan-1-yl)-2-(p-tolyl)-4H-pyrrolo[2,3-d]oxazole* (**3n**). Compound **3n** was prepared following the general procedure GP-B from azirine **2n** (60 mg, 0.18 mmol) in mesitylene (1.0 mL) in 21 mg (35% yield, after column chromatography on silica (light petroleum/ethyl acetate, 7:1 (*v/v*)) as a light brown solid: mp 198–201 °C (light petroleum/ethyl acetate). ^1^H-NMR (CDCl_3_, 400 MHz): *δ* 1.70–1.79 (m, 6H), 1.92–1.93 (m, 6H), 2.06 (s, 3H), 2.39 (s, 3H), 5.92 (s, 1H), 7.23–7.25 (m, 2H), 7.92–7.94 (m, 2H), 8.31 (s, 1H). ^13^C{^1^H}-NMR (CDCl_3_, 100 MHz): *δ* 21.4 (CH), 28.5 (CH), 34.4 (C), 36.7 (CH_2_), 42.9 (CH_2_), 85.8 (CH), 125.7 (CH), 126.3 (C), 129.4 (CH), 137.6 (C), 139.3 (C), 141.0 (C), 144.6 (C), 161.0 (C). HRMS (ESI) *m/z*: [M + Na]^+^ calcd. for C_22_H_24_N_2_NaO^+^ 355.1781; found 355.1781. IR (KBr, cm^−1^): ν 1551, 1570, 2847, 2908, 3261.

*2-(4-Bromophenyl)-5-phenyl-4H-pyrrolo[2,3-d]oxazole* (**3o**). Compound **3o** was prepared following the general procedure GP-B from azirine **2o** (48 mg, 0.14 mmol) in mesitylene (0.5 mL) in 26 mg (54% yield, after evaporation of solvent and washing with cold ether) as a light brown solid: mp 244–245 °C (mesitylene). ^1^H-NMR (DMSO-*d*_6_, 400 MHz): *δ* 6.74 (d, 1H, *J* = 1.5 Hz), 7.21–7.24 (m, 1H), 7.37–7.41 (m, 2H), 7.70–7.73 (m, 4H), 7.92–7.94 (m, 2H), 11.89 (s, 1H). ^13^C{^1^H}-NMR (DMSO-*d*_6_, 100 MHz): *δ* 87.9 (CH), 123.0 (C), 123.8 (CH), 126.5 (CH), 127.1 (CH), 127.2 (C), 128.8 (CH), 132.2 (CH), 132.9 (C), 133.8 (C), 140.3 (C), 141.9 (C), 159.8 (C). HRMS (ESI) *m/z*: [M + H]^+^ calcd. for C_17_H_12_N_2_O^+^ 339.0128; found 339.0131. IR (KBr, cm^−1^): ν 1597, 3262.

*2-(4-Bromophenyl)-5-(tert-butyl)-4H-pyrrolo[2,3-d]oxazole* (**3p**). Compound **3p** was prepared following the general procedure GP-B from azirine **2p** (91 mg, 0.29 mmol) in mesitylene (1.5 mL) in 44 mg (47% yield, after column chromatography on silica (light petroleum/ethyl acetate, 10:1 (*v/v*)) as a light brown solid: mp 181–183 °C (light petroleum/ethyl acetate). ^1^H-NMR (CDCl_3_, 400 MHz): *δ* 1.35 (s, 9H), 5.95 (d, 1H, *J* = 1.7 Hz), 7.54–7.56 (m, 2H), 7.87-7.89 (m, 2H), 8.07 (s, 1H). ^13^C{^1^H}-NMR (CDCl_3_, 100 MHz): *δ* 30.5 (CH_3_), 32.6 (C), 86.5 (CH), 123.3 (C), 127.0 (CH), 127.9 (C), 131.9 (CH), 138.0 (C), 141.4 (C), 144.7 (C), 159.7 (C). HRMS (ESI) *m/z*: [M + H]^+^ calcd. for C_15_H_16_BrN_2_O^+^ 319.0441; found 319.0434. IR (KBr, cm^−1^): ν 1534, 1566, 2955, 3225.

*(E)-3-(5-phenyl-4H-pyrrolo[2,3-d]oxazol-2-yl)acrylonitrile* (**3q**). Compound **3q** was prepared following the general procedure GP-B from azirine **2q** (84 mg, 0.36 mmol) in mesitylene (1.0 mL) in 25 mg (30% yield, after evaporation of solvent and washing with cold ether) as a yellow solid: mp 251–253 °C (mesitylene). ^1^H-NMR (DMSO-*d*_6_, 400 MHz): *δ* 6.46 (d, 1H, *J* = 16.3 Hz), 6.78 (d, 1H, *J* = 1.5 Hz), 7.27–7.31 (m, 1H), 7.41–7.45 (m, 2H), 7.55 (d, 1H, *J* = 16.3 Hz), 7.76–7.78 (m, 2H), 12.12 (s, 1H). ^13^C{^1^H}-NMR (DMSO-*d*_6_, 100 MHz): *δ* 88.2 (CH), 99.3 (CH), 118.7 (C), 124.8 (CH), 127.8 (CH), 129.4 (CH), 132.7 (C), 135.1 (CH), 138.0 (C), 141.8 (C), 143.7 (C), 158.2 (C). HRMS (ESI) *m/z*: [M + Na]^+^ calcd. for C_14_H_9_N_3_NaO^+^ 258.0638; found 258.0643. IR (KBr, cm^−1^): ν 1555, 1603, 1628, 1744, 2218, 2924, 3212.

*(2-Methyl-5-phenyl-4H-pyrrolo[2,3-d]oxazol-4-yl)(p-tolyl)methanone* (**4a**). Compound **4a** was prepared following the general procedure GP-C from 4*H*-pyrrolo[2,3-*d*]oxazole **3a** (100 mg, 0.51 mmol), NaH (30 mg, 0.76 mmol), 15-crown-5 (112 mg, 0.51 mmol), 4-methylbenzoyl chloride (154 mg, 1.0 mmol) in tetrahydrofuran (5.0 mL) in 132 mg (83% yield, after column chromatography on silica (light petroleum/ethyl acetate, 10:1 (*v/v*)) as a yellow solid: mp 121–124 °C (light petroleum/ethyl acetate). ^1^H-NMR (CDCl_3_, 400 MHz): *δ* 2.44 (s, 3H), 2.52 (s, 3H), 6.43 (s, 1H), 7.21–7.25 (m, 2H), 7.27–7.234 (m, 5H), 7.85–7.87 (m, 2H). ^13^C{^1^H}-NMR (CDCl_3_, 100 MHz): *δ* 15.1 (CH_3_), 21.8 (CH_3_), 98.2 (CH), 127.2 (CH), 127.8 (CH), 128.3 (CH), 129.1 (CH), 130.0 (C), 131.2 (CH), 133.3 (C), 136.5 (C), 140.9 (C), 141.0 (C), 144.7 (C), 162.4 (C), 166.7 (C). HRMS (ESI) *m/z*: [M + Na]^+^ calcd. for C_20_H_16_N_2_NaO^+^ 339.1104; found 339.1104. IR (KBr, cm^−1^): ν 1611, 1705, 2919.

*(2,5-Diphenyl-4H-pyrrolo[2,3-d]oxazol-4-yl)(p-tolyl)methanone* (**4b**). Compound **4b** was prepared following the general procedure GP-C from 4*H*-pyrrolo[2,3-*d*]oxazole **3h** (58 mg, 0.22 mmol), NaH (14 mg, 0.33 mmol), 15-crown-5 (49 mg, 0.55 mmol), 4-methylbenzoyl chloride (68 mg, 0.44 mmol) in tetrahydrofuran (3.0 mL) in 46 mg (55% yield, after column chromatography on silica (light petroleum/ethyl acetate, 20:1 (*v/v*)) as a yellow solid: mp 156–158 °C (light petroleum/ethyl acetate). ^1^H-NMR (CDCl_3_, 400 MHz): *δ* 2.47 (s, 3H), 6.53 (s, 1H), 7.25–7.34 (m, 5H), 7.36–7.43 (m, 5H), 7.90–7.92 (m, 2H), 7.97–8.00 (m, 2H). ^13^C{^1^H}-NMR (CDCl_3_, 400 MHz): *δ* 21.8 (CH_3_), 98.1 (CH), 126.2 (CH), 127.4 (CH), 127.8 (CH), 128.1 (C), 128.3 (CH), 128.7 (CH), 129.0 (CH), 129.95 (C), 129.99 (CH), 131.4 (CH), 133.2 (C), 137.8 (C), 141.4 (C), 142.3 (C), 144.7 (C), 162.2 (C), 166.8 (C). HRMS (ESI) *m/z*: [M + Na]^+^ calcd. for C_25_H_18_N_2_NaO^+^ 401.1260; found 401.1262. IR (KBr, cm^−1^): ν 1609, 1699, 2854, 2924.

## 4. Conclusions

A series of variously substituted 4*H*-pyrrolo[2,3-*d*]oxazoles was synthesized by thermally induced isomerization of 5-(2*H*-azirin-2-yl)oxazoles in mesitylene at 180 °C. The reaction tolerates a variety of substituted aryl and alkyl groups at the 3 position in the azirine fragment and at the 2 position in the oxazole fragment of starting compounds. Whereas heating 5-(3-phenyl-2*H*-azirin-2-yl)-2-vinyl/(chloromethyl)oxazoles resulted in complete resinification of the reaction mixtures. Starting 5-(2*H*-azirin-2-yl)oxazoles were prepared by Rh_2_(oct)_4_ catalyzed reaction of 2-(3-aryl/heteroaryl)-2-diazoacetyl-2*H*-azirines with a set of substituted acetonitriles, benzonitriles, acrylonitrile and fumaronitrile. According to DFT calculations at the DFT B3LYP-D3/6-311+G(d,p) level of theory with SMD model for mesitylene, the transformation of 5-(2*H*-azirin-2-yl)oxazole to 4*H*-pyrrolo[2,3-*d*]oxazole occurs through the nitrenoid-like transition state to give 3a*H*-pyrrolo[2,3-*d*]oxazole intermediate, followed by either by 1,5-H-shift or a pathway involving intermolecular 1,2-prototropic shift.

## Data Availability

Data are contained within the article.

## Supplementary Materials

The following are available online, X-Ray diffraction experiments; NMR spectra of compounds **2–4**; Computational Details.

## Sample Availability

Samples of the compounds **2–4** are available from the authors.
