# Supplementary material for: Isomerization of 5-(2H-Azirin-2-yl)oxazoles: An Atom-Economic Approach to 4H-Pyrrolo[2,3-d]oxazoles
Source: Molecules. 2021 Mar 26;26(7):1881. doi: 10.3390/molecules26071881 (PMC8036974; doi:10.3390/molecules26071881)
Supplement: Supplementary file 1 [file molecules-26-01881-s001.pdf]

Supporting Information

for

Isomerization of 5-(2*H*-Azirin-2-yl)oxazoles: an Atom-Economic Approach to 4*H*-Pyrrolo[2,3-*d*]oxazoles

Timur O. Zanakhov, Ekaterina E. Galenko, Mariya A. Kryukova, Mikhail S. Novikov, Alexander A. Khlebnikov*

St. Petersburg State University, Institute of Chemistry, 7/9 Universitetskaya nab., St. Petersburg, 199034, Russia

E-mail: a.khlebnikov@spbu.ru

Table of Contents:

X-Ray Diffraction Experiments S2
NMR Spectra of Compounds **2** S6
NMR Spectra of Compounds **3** S59
NMR Spectra of Compounds **4** S110
Computational Details S116

X-RAY DIFFRACTION EXPERIMENTS

Crystal structure of **3d** was determined by single crystal X-ray diffraction analysis. Suitable crystals were selected and fixed on micro-mounts and the diffraction data were collected on a HyPix diffractometer. The crystal of **3d** was measured at a temperature of 100(2) K, using monochromated CuKα radiation. The unit cell parameters and refinement characteristics of the crystal structure of **3d** is given below. Using Olex2 [1], the structures were solved with the ShelXT [2] structure solution program using Intrinsic Phasing and refined with the ShelXL [3] refinement package using Least Squares minimization.

References

1. Dolomanov, O.V.; Bourhis, L.J.; Gildea, R.J; Howard, J.A.K.; Puschmann, H. J. Appl. Cryst. 2009, 42, 339.

2. Sheldrick, G.M. Acta Cryst. 2015, A71, 3.

3. Sheldrick, G.M.Acta Cryst. 2015, C71, 3.

2-Ethyl-5-phenyl-4*H*-pyrrolo[2,3-*d*]oxazole

Single crystal of **3d** was obtained by slow evaporation of toluene solution at room temperature. (CCDC 2064882).

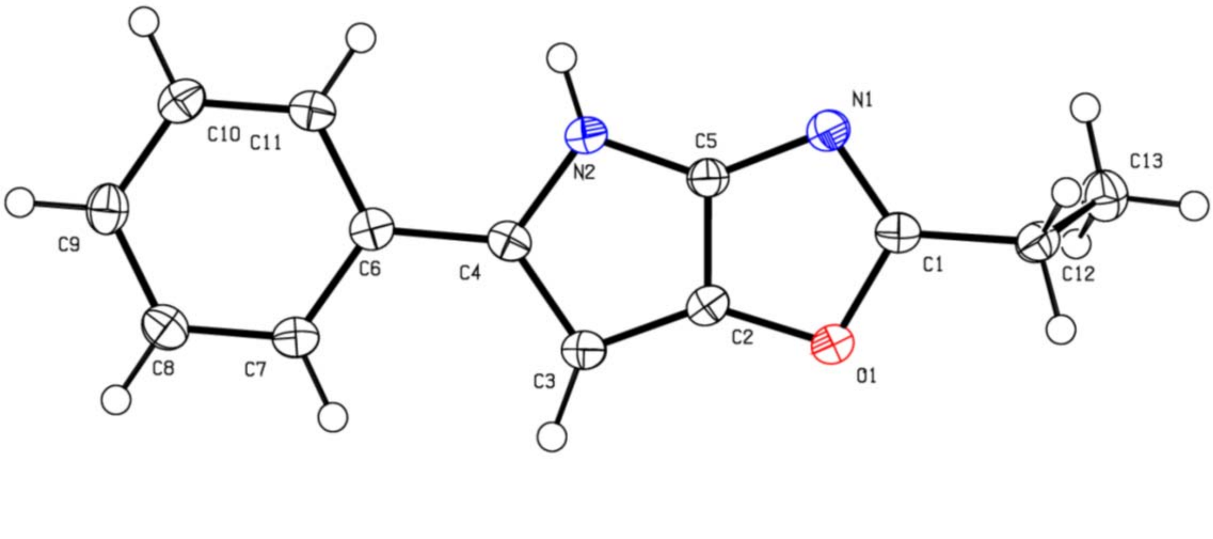

Molecular structure of compound **3d**, displacement parameters are drawn at 50% probability level.

Table S1. Crystal data and structure refinement for 3d.

Identification code	**3d** (17505_TZ-19)
Empirical formula	$C_{13}H_{12}N_2O$
Formula weight	212.25
Temperature/K	100.00(10)
Crystal system	monoclinic
Space group	$P2_1/c$
a/Å	13.1494(3)
b/Å	14.4153(3)
c/Å	5.49770(10)
α/°	90
β/°	90.628(2)
γ/°	90
Volume/Å^3	1042.04(4)
Z	4
ρ_{calc}g/cm^3	1.353
μ/mm^{-1}	0.701
F(000)	448.0
Crystal size/mm^3	0.15 × 0.13 × 0.1
Radiation	CuKα (λ = 1.54184)
2Θ range for data collection/°	6.722 to 139.978
Index ranges	-16 ≤ h ≤ 16, -17 ≤ k ≤ 17, -6 ≤ l ≤ 6
Reflections collected	10049
Independent reflections	1920 [R_{int} = 0.0454, R_{sigma} = 0.0364]
Data/restraints/parameters	1920/0/150
Goodness-of-fit on F^2	1.059
Final R indexes [I>=2σ (I)]	R_1 = 0.0392, wR_2 = 0.1004
Final R indexes [all data]	R_1 = 0.0478, wR_2 = 0.1053
Largest diff. peak/hole / e Å^{-3}	0.14/-0.22

Table S2. Fractional Atomic Coordinates (×10^4) and Equivalent Isotropic Displacement Parameters (Å^2×10^3) for 3d. U_{eq} is defined as 1/3 of of the trace of the orthogonalised U_{IJ} tensor.

Atom	*x*	*y*	*z*	U(eq)
O_1	3380.4(7)	3510.6(6)	5323.8(16)	21.7(2)
N_2	5667.9(8)	4266.0(7)	7570.2(19)	20.5(3)
N_1	3772.9(8)	4400.5(7)	8615(2)	21.5(3)
C_4	6091.1(10)	3807.2(8)	5595(2)	18.8(3)
C_5	4641.1(10)	4137.9(9)	7412(2)	19.5(3)
C_2	4427.9(10)	3607.2(9)	5434(2)	20.1(3)
C_1	3053.3(10)	4011.7(8)	7310(2)	20.2(3)
C_{11}	7865.4(10)	4218.7(9)	6870(2)	21.6(3)
C_8	8636.4(10)	3378.9(9)	2752(2)	23.5(3)
C_6	7188.6(10)	3810.0(8)	5205(2)	19.1(3)
C_{12}	1941.3(10)	4029.3(9)	7739(2)	21.5(3)
C_3	5323.5(10)	3381.4(9)	4217(2)	20.8(3)
C_9	9298.6(10)	3787.0(9)	4427(3)	23.7(3)
C_7	7594.8(10)	3393.7(8)	3121(2)	20.9(3)
C_{10}	8904.9(10)	4207.6(9)	6486(2)	24.0(3)
C_{13}	1629.4(11)	3416.9(10)	9864(3)	27.8(3)

Table S3. Anisotropic Displacement Parameters ($Å^2 \times 10^3$) for 3d. The Anisotropic displacement factor exponent takes the form: $-2\pi^2[h^2a^{*2}U_{11}+2hka^*b^*U_{12}+\ldots]$.

Atom	U_{11}	U_{22}	U_{33}	U_{23}	U_{13}	U_{12}
O_1	20.2(5)	24.0(5)	20.7(5)	-3.1(4)	-1.3(4)	-1.9(3)
N_2	19.9(5)	22.2(5)	19.4(5)	-4.5(4)	-1.3(4)	-1.3(4)
N_1	20.8(6)	22.2(5)	21.6(5)	-1.8(4)	-1.3(4)	-0.3(4)
C_4	23.0(7)	15.4(6)	18.0(6)	0.7(5)	1.2(5)	0.6(5)
C_5	20.3(6)	19.6(6)	18.5(6)	-1.4(5)	-0.7(5)	-0.1(5)
C_2	19.8(6)	20.2(6)	20.2(6)	0.3(5)	-2.5(5)	-1.9(5)
C_1	23.7(7)	18.3(6)	18.7(6)	0.6(5)	-1.3(5)	0.6(5)
C_{11}	25.9(7)	20.4(6)	18.6(6)	-0.7(5)	0.0(5)	-0.2(5)
C_8	26.8(7)	21.1(6)	22.6(6)	0.5(5)	1.9(5)	2.5(5)
C_6	22.7(7)	15.3(6)	19.3(6)	3.2(5)	-0.9(5)	-0.4(5)
C_{12}	20.8(6)	21.1(6)	22.4(6)	-1.5(5)	-3.1(5)	0.2(5)
C_3	23.2(6)	20.9(6)	18.3(6)	-1.5(5)	-0.7(5)	-0.9(5)
C_9	20.3(6)	23.6(6)	27.1(7)	4.7(5)	0.5(5)	1.5(5)
C_7	24.9(7)	18.1(6)	19.8(6)	0.2(5)	-1.7(5)	0.0(5)
C_{10}	24.1(7)	24.5(6)	23.3(7)	1.4(5)	-4.0(5)	-2.4(5)
C_{13}	26.5(7)	28.9(7)	27.9(7)	2.4(6)	1.5(6)	1.0(6)

Table S4. Bond Lengths for 3d.

Atom	Atom	Length/Å	Atom	Atom	Length/Å
O_1	C_2	1.3852(15)	C_2	C_3	1.3990(19)
O_1	C_1	1.3816(16)	C1	C_{12}	1.4839(19)
N_2	C_4	1.3926(17)	C_{11}	C_6	1.3995(17)
N_2	C_5	1.3647(16)	C_{11}	C_{10}	1.3855(19)
N_1	C_5	1.3787(18)	C_8	C_9	1.3910(18)
N_1	C_1	1.3074(16)	C_8	C_7	1.3870(19)
C_4	C_6	1.4614(18)	C_6	C_7	1.4041(19)
C_4	C_3	1.3973(17)	C_{12}	C_{13}	1.5243(19)
C_5	C_2	1.3562(18)	C_9	C_{10}	1.389(2)

Table S5. Bond Angles for 3d.

Atom	Atom	Atom	Angle/°
C_1	O_1	C_2	103.35(9)
C_5	N_2	C_4	106.82(10)
C_1	N_1	C_5	102.53(11)
N_2	C_4	C_6	121.11(11)
N_2	C_4	C_3	109.81(11)
C_3	C_4	C_6	129.07(12)
N_2	C_5	N_1	139.12(11)
C_2	C_5	N_2	108.85(12)
C_2	C_5	N_1	112.00(11)
O_1	C_2	C_3	142.90(11)
C_5	C_2	O_1	106.76(12)
C_5	C_2	C_3	110.30(11)
O_1	C_1	C_{12}	116.76(10)

Atom	Atom	Atom	Angle/°
N_1	C_1	O_1	115.35(12)
N_1	C_1	C_{12}	127.87(12)
C_{10}	C_{11}	C_6	121.13(13)
C_7	C_8	C_9	120.58(13)
C_{11}	C_6	C_4	121.81(12)
C_{11}	C_6	C_7	117.95(12)
C_7	C_6	C_4	120.24(11)
C_1	C_{12}	C_{13}	112.70(10)
C_4	C_3	C_2	104.20(11)
C_{10}	C_9	C_8	119.20(12)
C_8	C_7	C_6	120.73(12)
C_{11}	C_{10}	C_9	120.41(12)

Table S6. Hydrogen Atom Coordinates (Å×10^4) and Isotropic Displacement Parameters (Å^2×10^3) for 3d.

Atom	*x*	*y*	*z*	U(eq)
H_2	5992	4571	8677	25
H_{11}	7613	4503	8257	26
H_8	8894	3094	1373	28
H_{12A}	1588	3822	6278	26
H_{12B}	1732	4663	8060	26
H_9	9997	3778	4172	28
H_7	7161	3125	1975	25
H_{10}	9342	4484	7614	29
H_{13A}	1838	2790	9563	42
H_{13B}	904	3438	10036	42
H_{13C}	1949	3638	11332	42
H_3	5427(12)	3017(12)	2730(30)	31(4)

2-Methyl-5-(3-phenyl-2*H*-azirin-2-yl)oxazole 2a, ^{1}H NMR, 400 MHz, $CDCl_3$

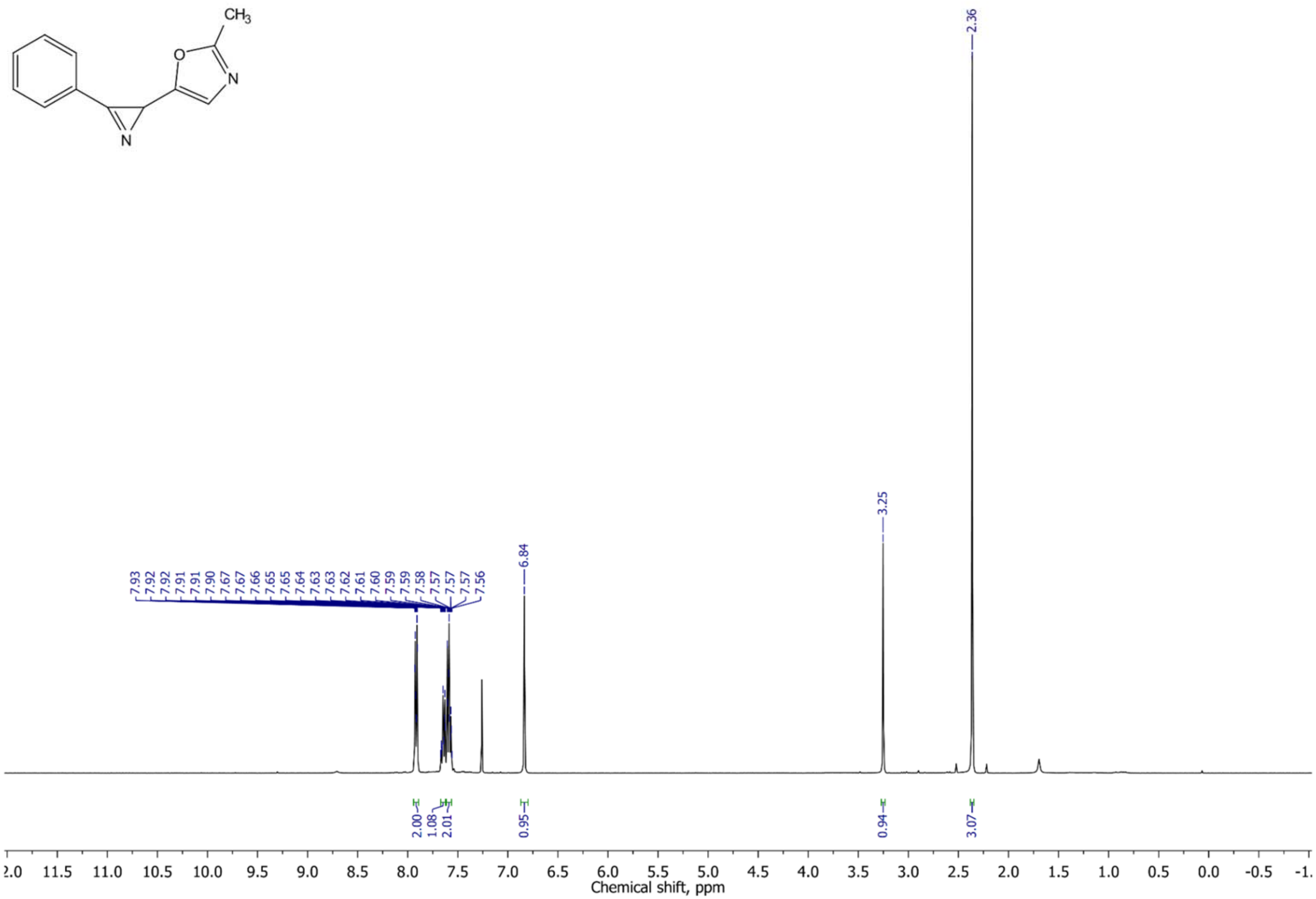

2-Methyl-5-(3-(4-bromophenyl)-2*H*-azirin-2-yl)oxazole 2b, **^{1}H NMR, 400 MHz, $CDCl_3$**

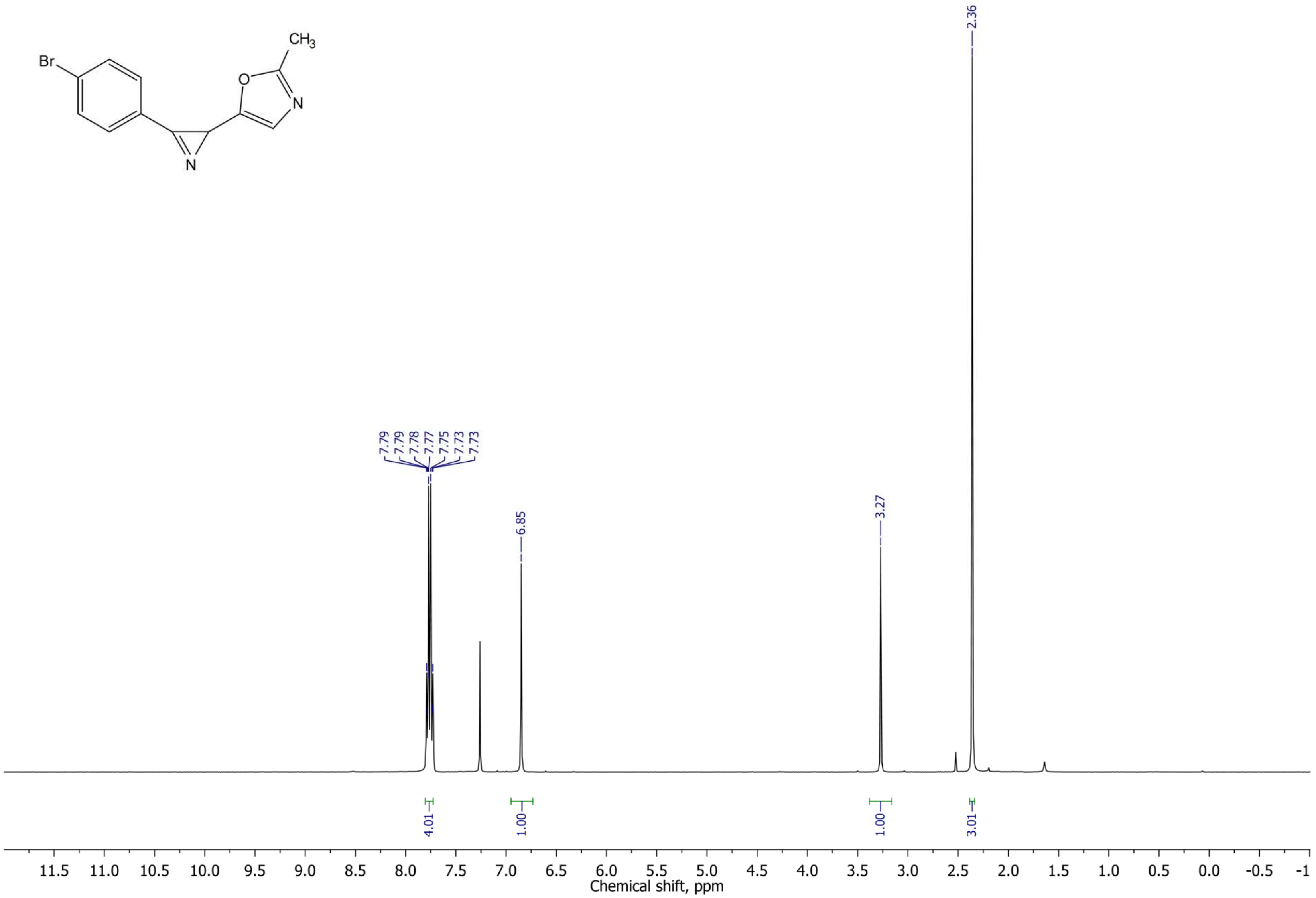

2-Methyl-5-(3-(4-bromophenyl)-2*H*-azirin-2-yl)oxazole 2b, $^{13}C\{^1H\}$ NMR, 100 MHz, $CDCl_3$

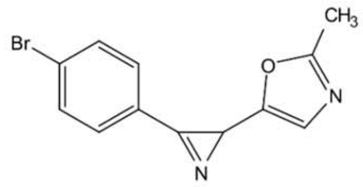

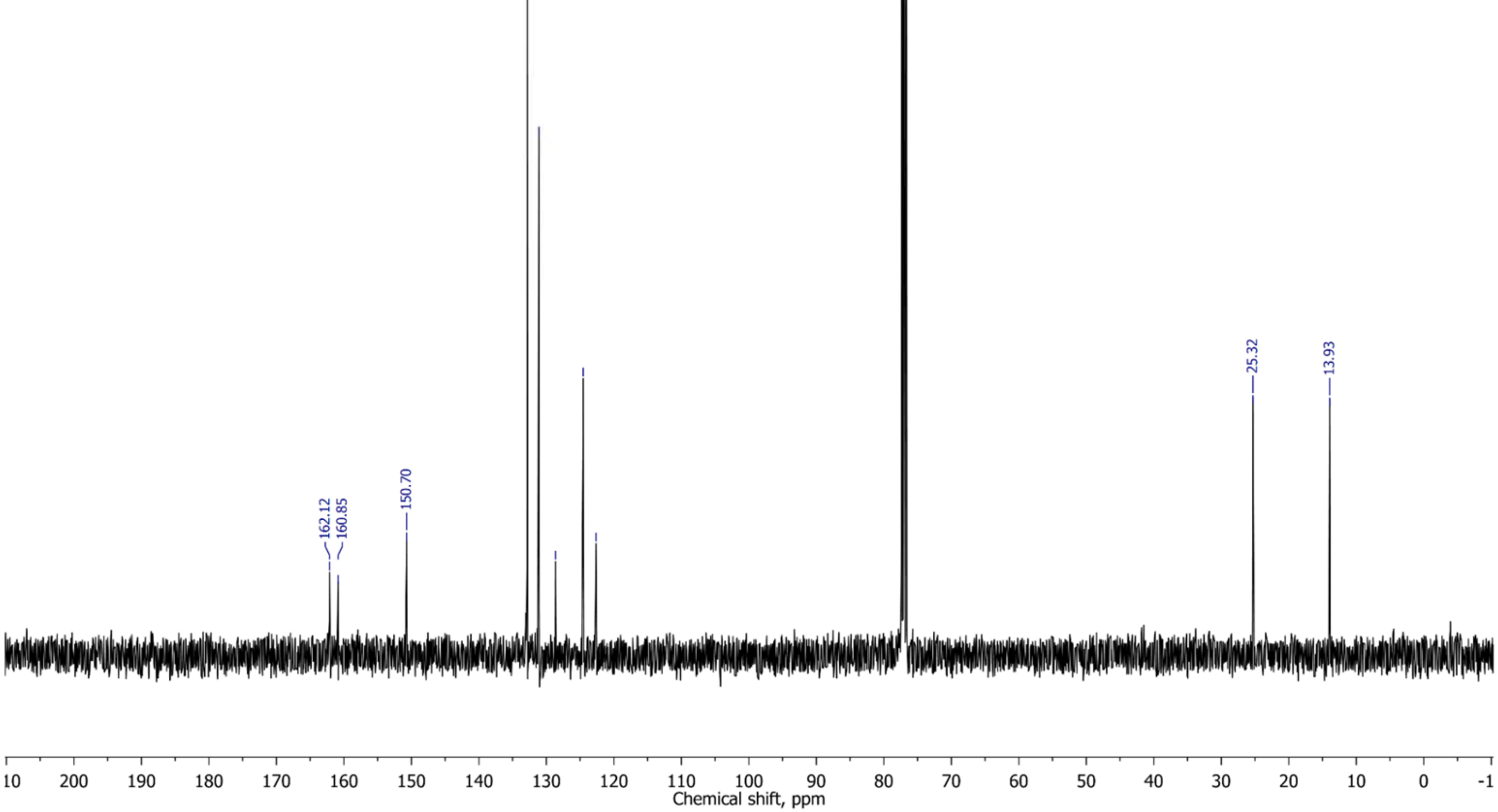

2-Methyl-5-(3-(4-bromophenyl)-2*H*-azirin-2-yl)oxazole 2b, DEPT, 100 MHz, $CDCl_3$

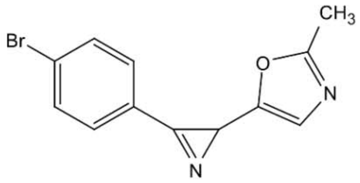

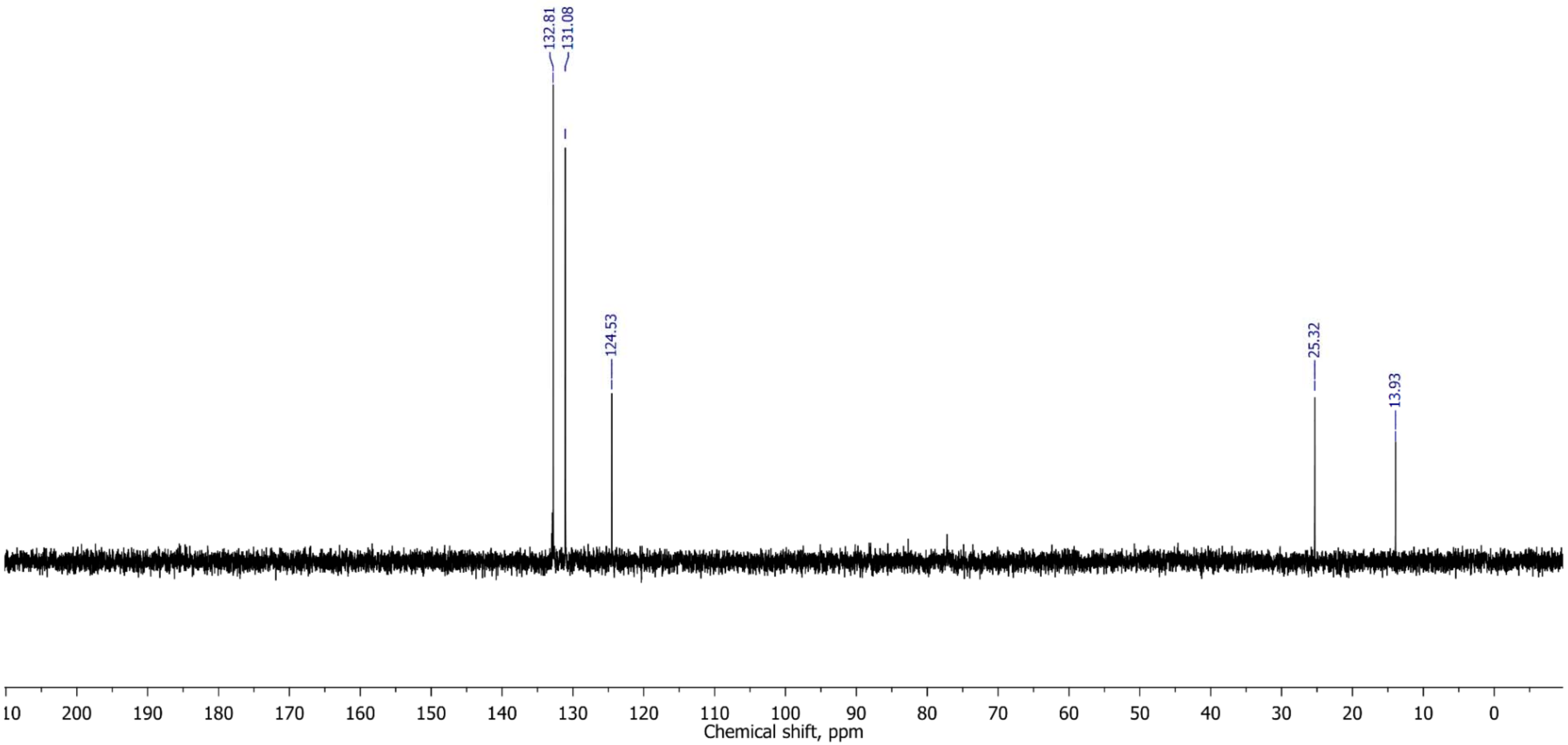

5-(3-(Adamantan-1-yl)-2*H*-azirin-2-yl)-2-methyloxazole 2c, ^{1}H NMR, 400 MHz, $CDCl_3$

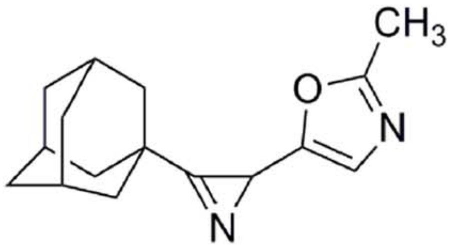

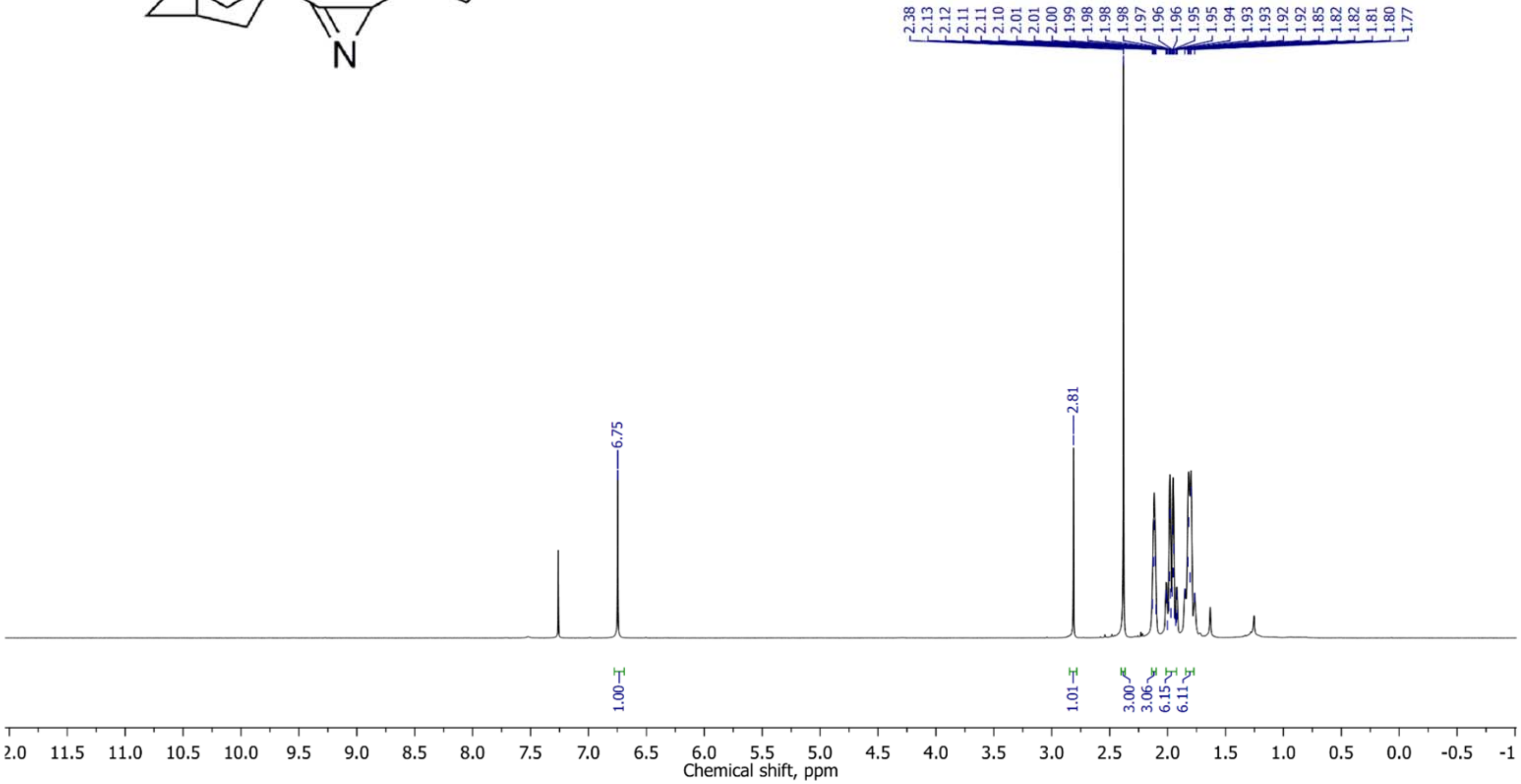

5-(3-(Adamantan-1-yl)-2*H*-azirin-2-yl)-2-methyloxazole 2c, $^{13}C\{^1H\}$ NMR, 100 MHz, $CDCl_3$

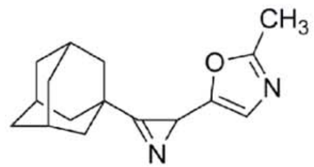

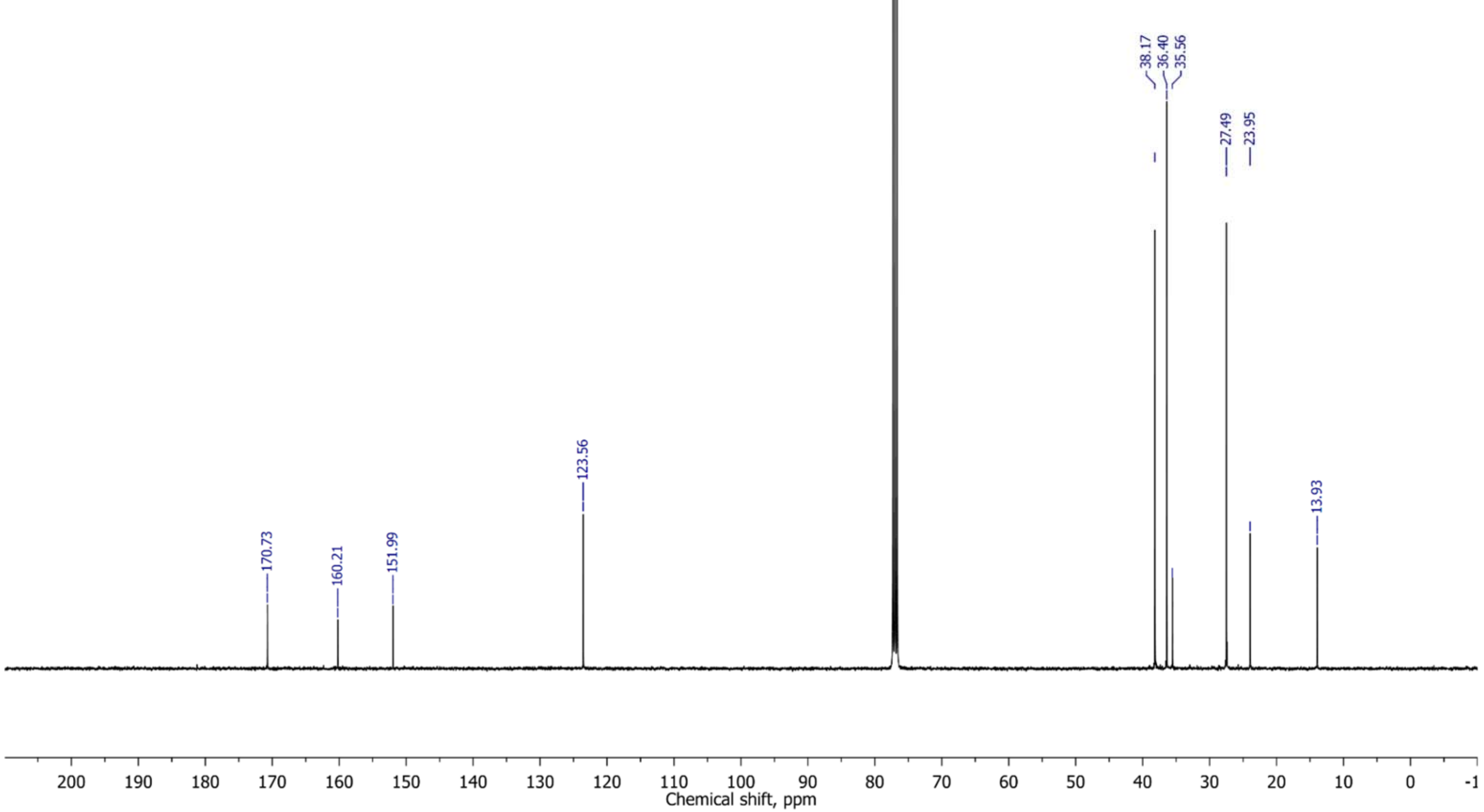

5-(3-(Adamantan-1-yl)-2*H*-azirin-2-yl)-2-methyloxazole 2c, DEPT, 100 MHz, $CDCl_3$

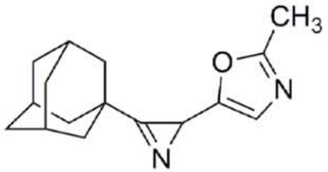

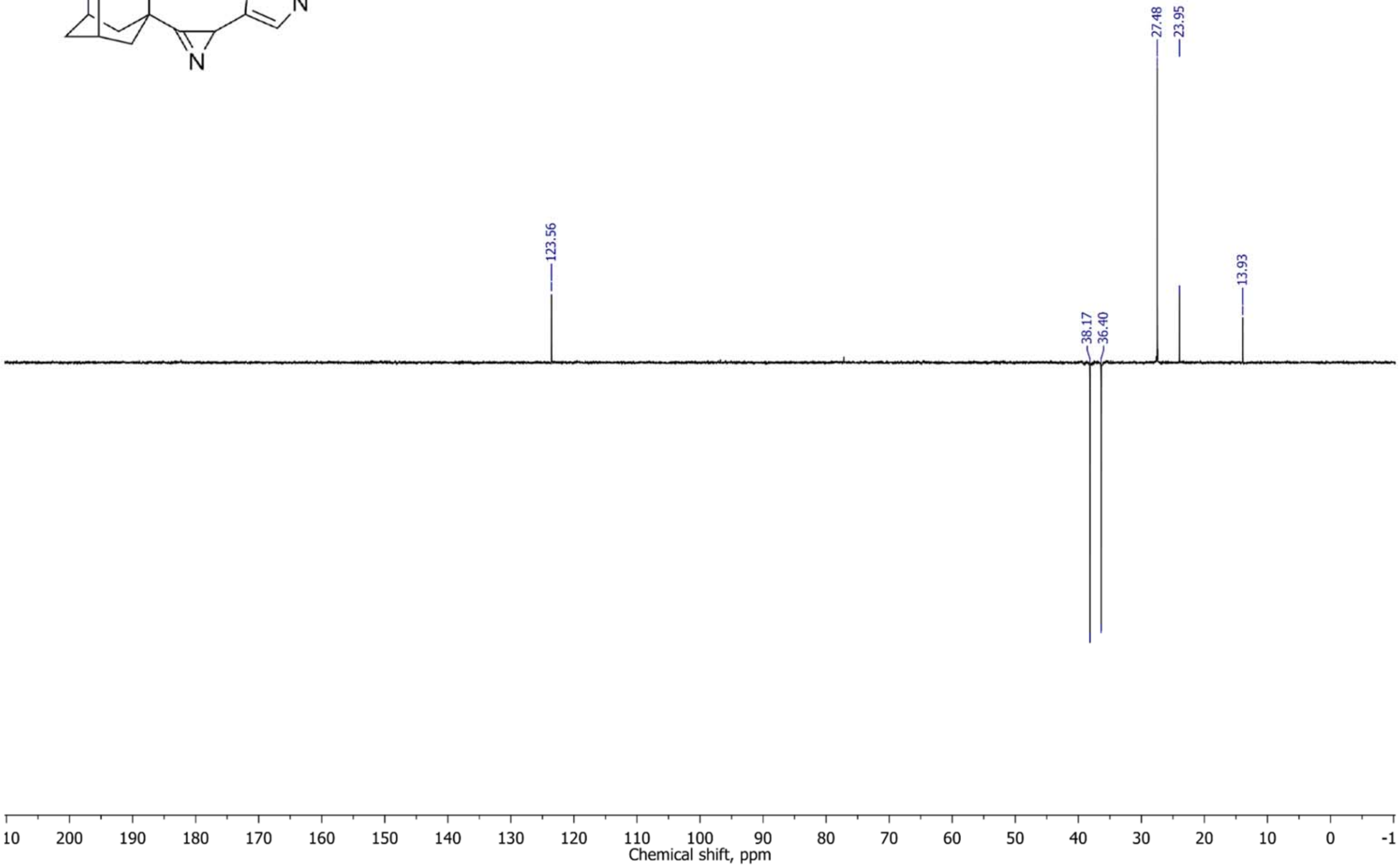

2-Ethyl-5-(3-phenyl-2*H*-azirin-2-yl)oxazole 2d, ^{1}H NMR, 400 MHz, $CDCl_3$

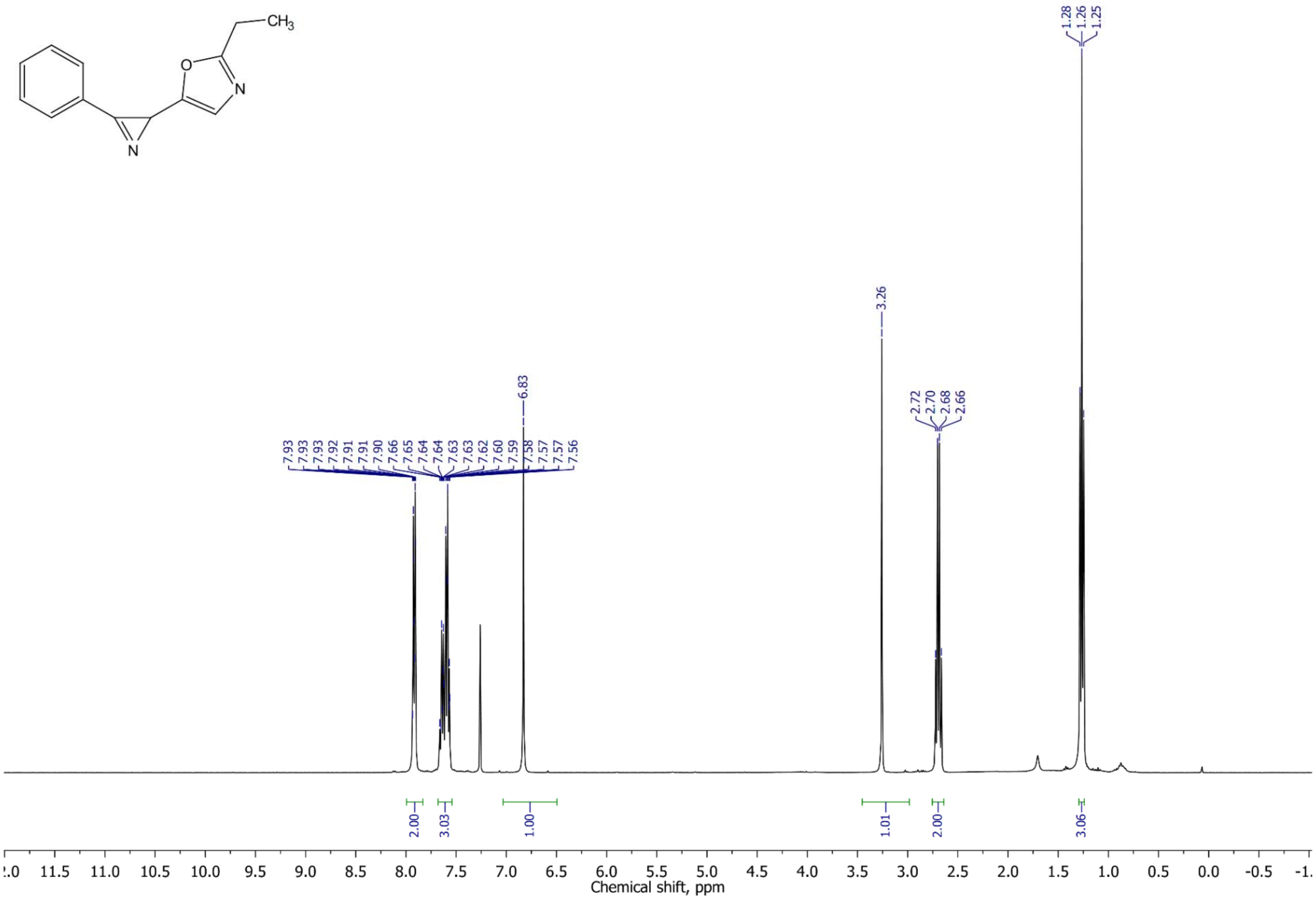

2-Ethyl-5-(3-phenyl-2*H*-azirin-2-yl)oxazole 2d, $^{13}C\{^{1}H\}$ NMR, 100 MHz, $CDCl_3$

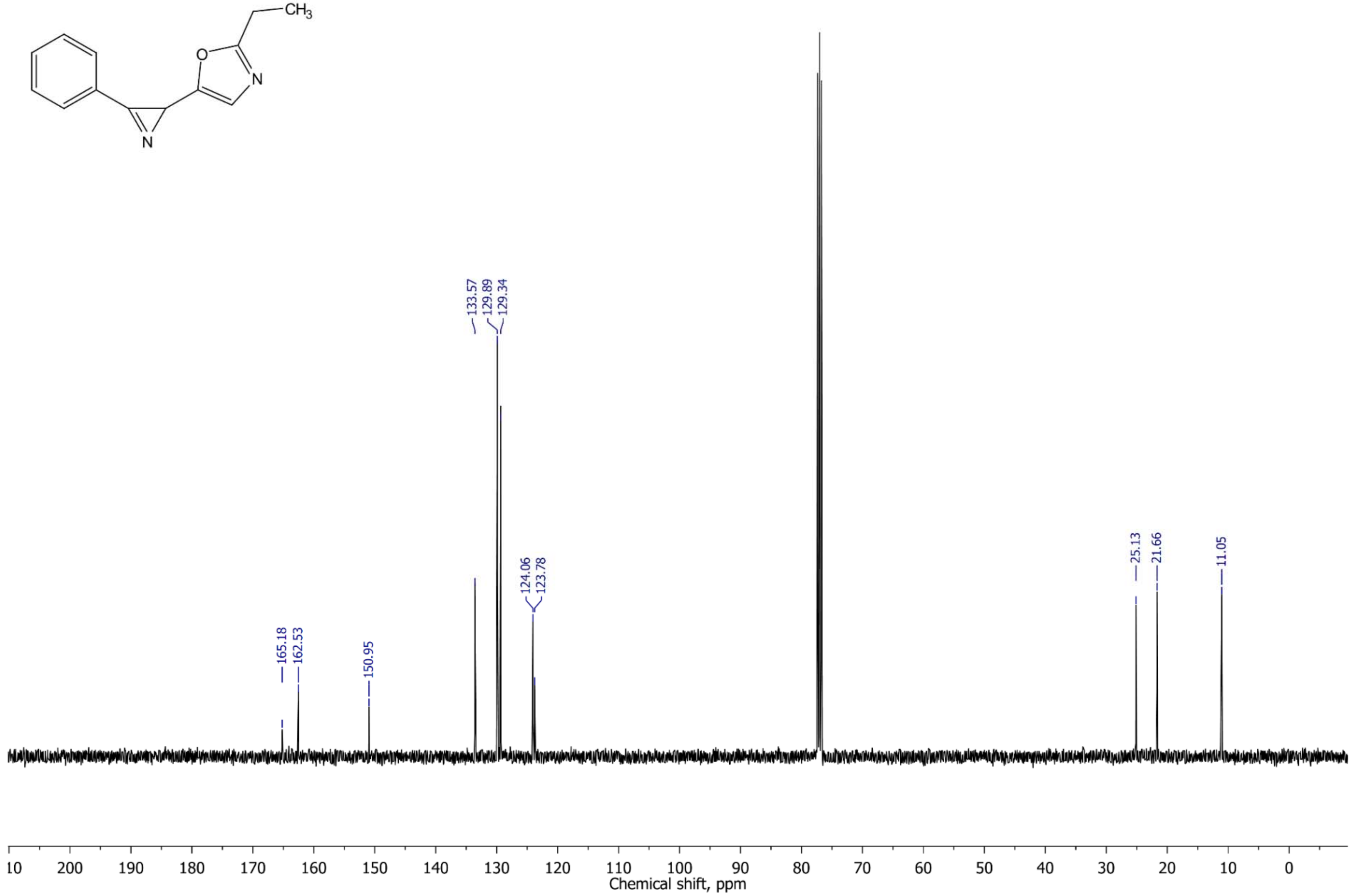

2-Ethyl-5-(3-phenyl-2*H*-azirin-2-yl)oxazole 2d, DEPT, 100 MHz, $CDCl_3$

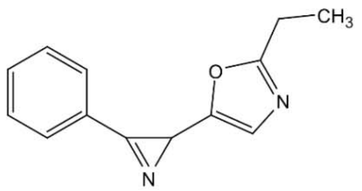

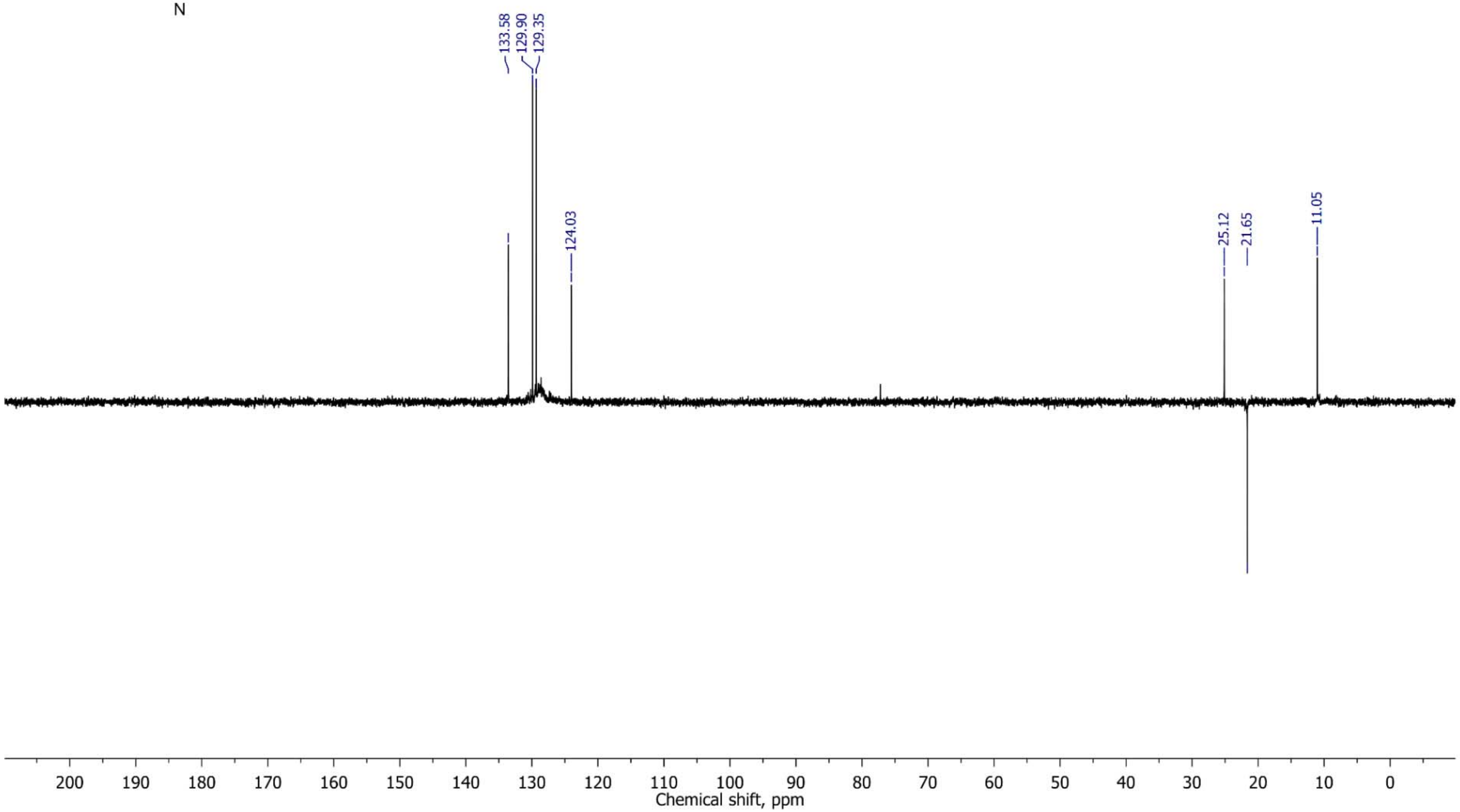

2-Ethyl-5-(3-(4-methoxyphenyl)-2*H*-azirin-2-yl)oxazole 2e, ^{1}H NMR, 400 MHz, $CDCl_3$

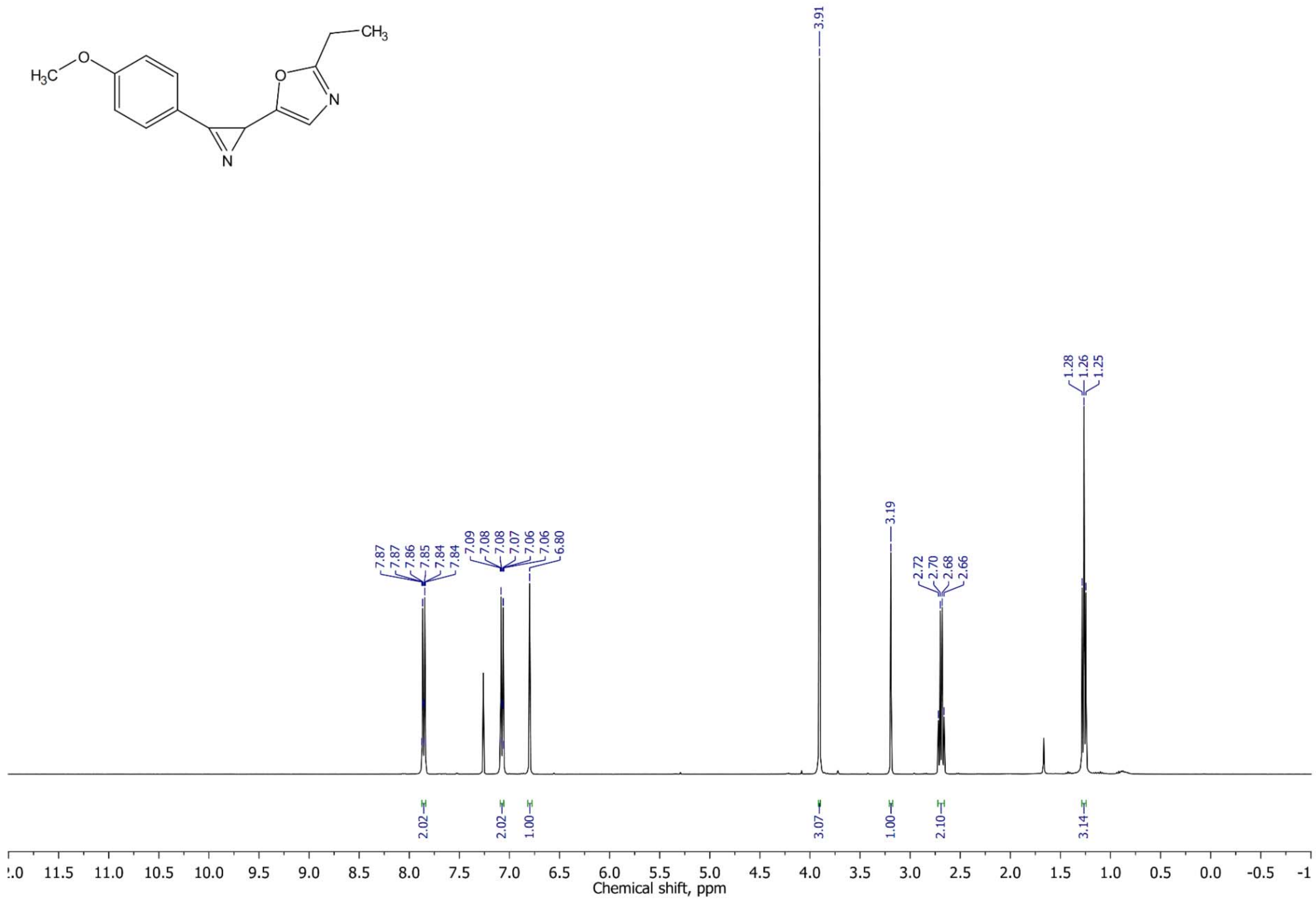

2-Ethyl-5-(3-(4-methoxyphenyl)-2*H*-azirin-2-yl)oxazole 2e, $^{13}C\{^{1}H\}$ NMR, 100 MHz, $CDCl_3$

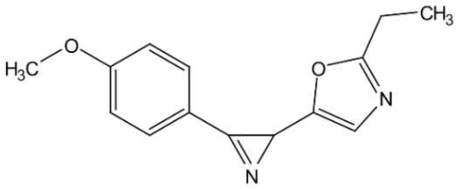

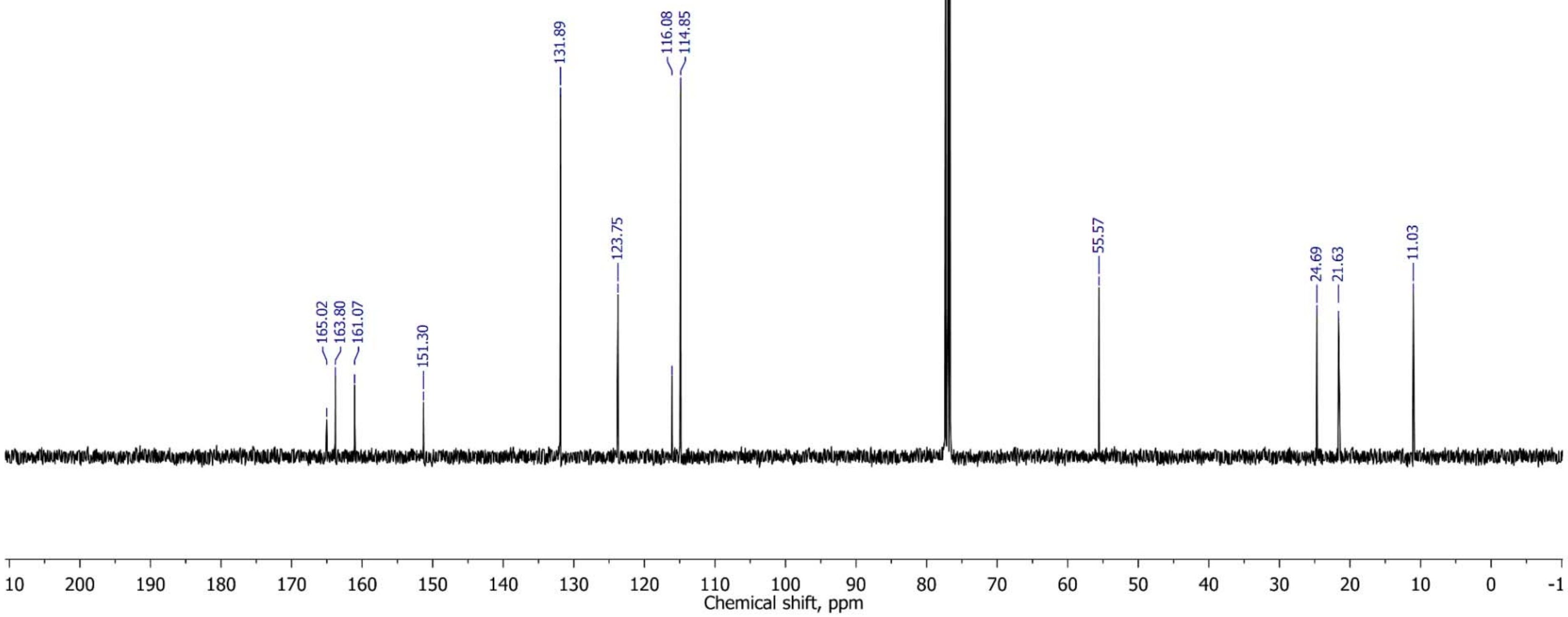

2-Ethyl-5-(3-(4-methoxyphenyl)-2*H*-azirin-2-yl)oxazole 2e, DEPT, 100 MHz, $CDCl_3$

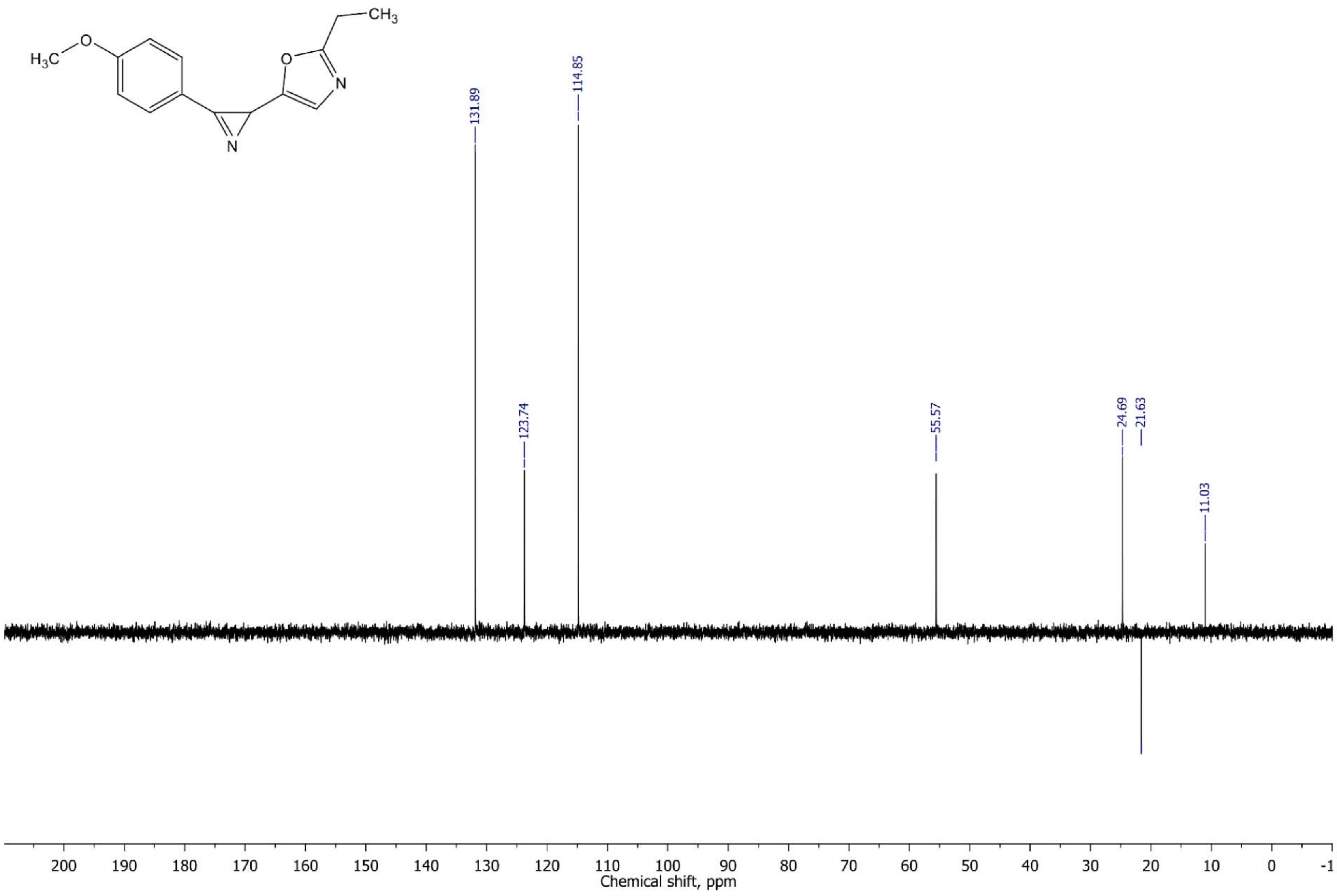

5-(3-(*tert*-Butyl)-2*H*-azirin-2-yl)-2-ethyloxazole 2f, ^{1}H NMR, 400 MHz, $CDCl_3$

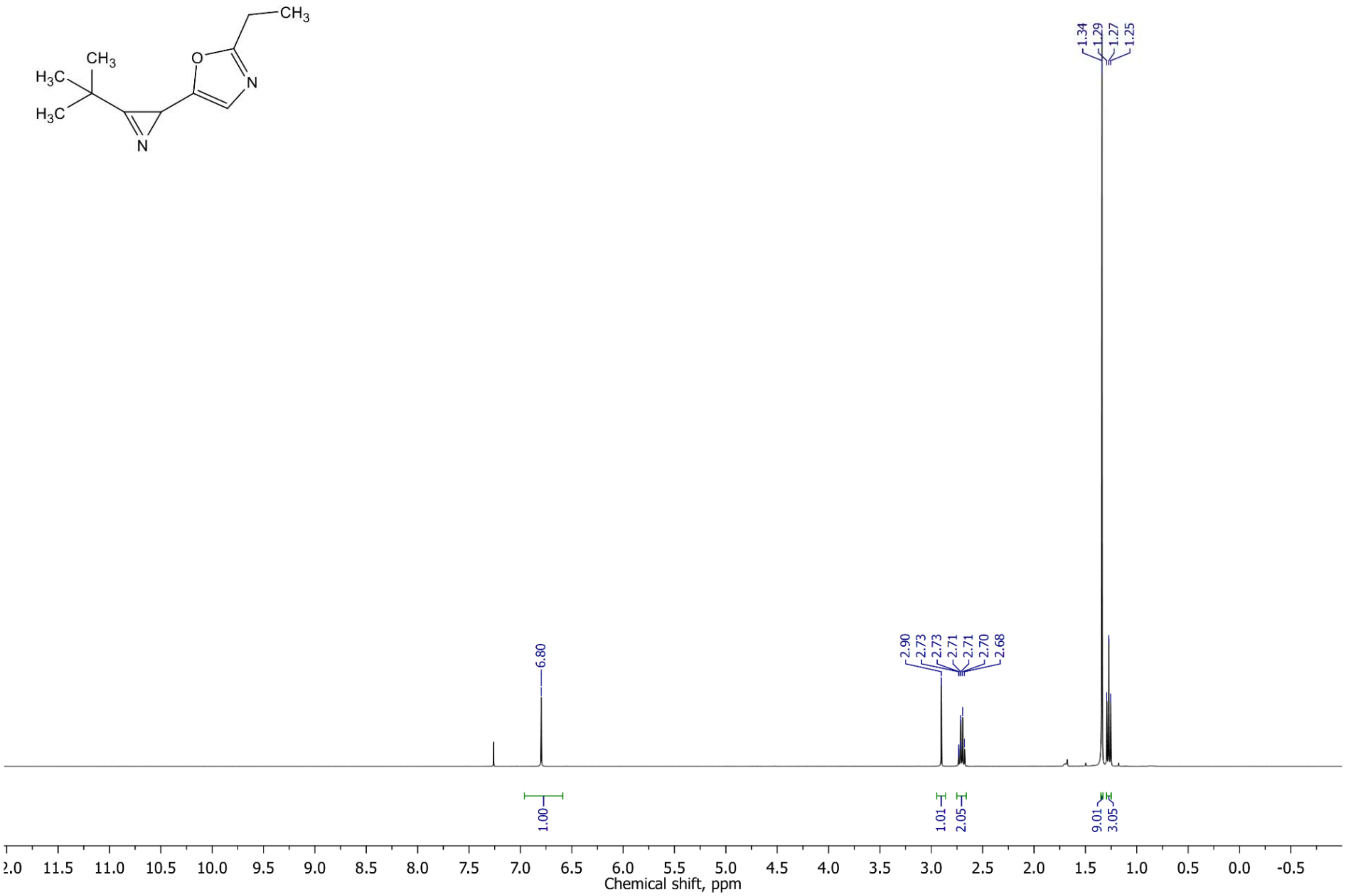

5-(3-(*tert*-Butyl)-2*H*-azirin-2-yl)-2-ethyloxazole 2f, $^{13}C\{^{1}H\}$ NMR, 100 MHz, $CDCl_3$

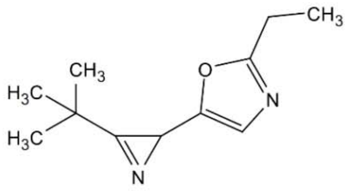

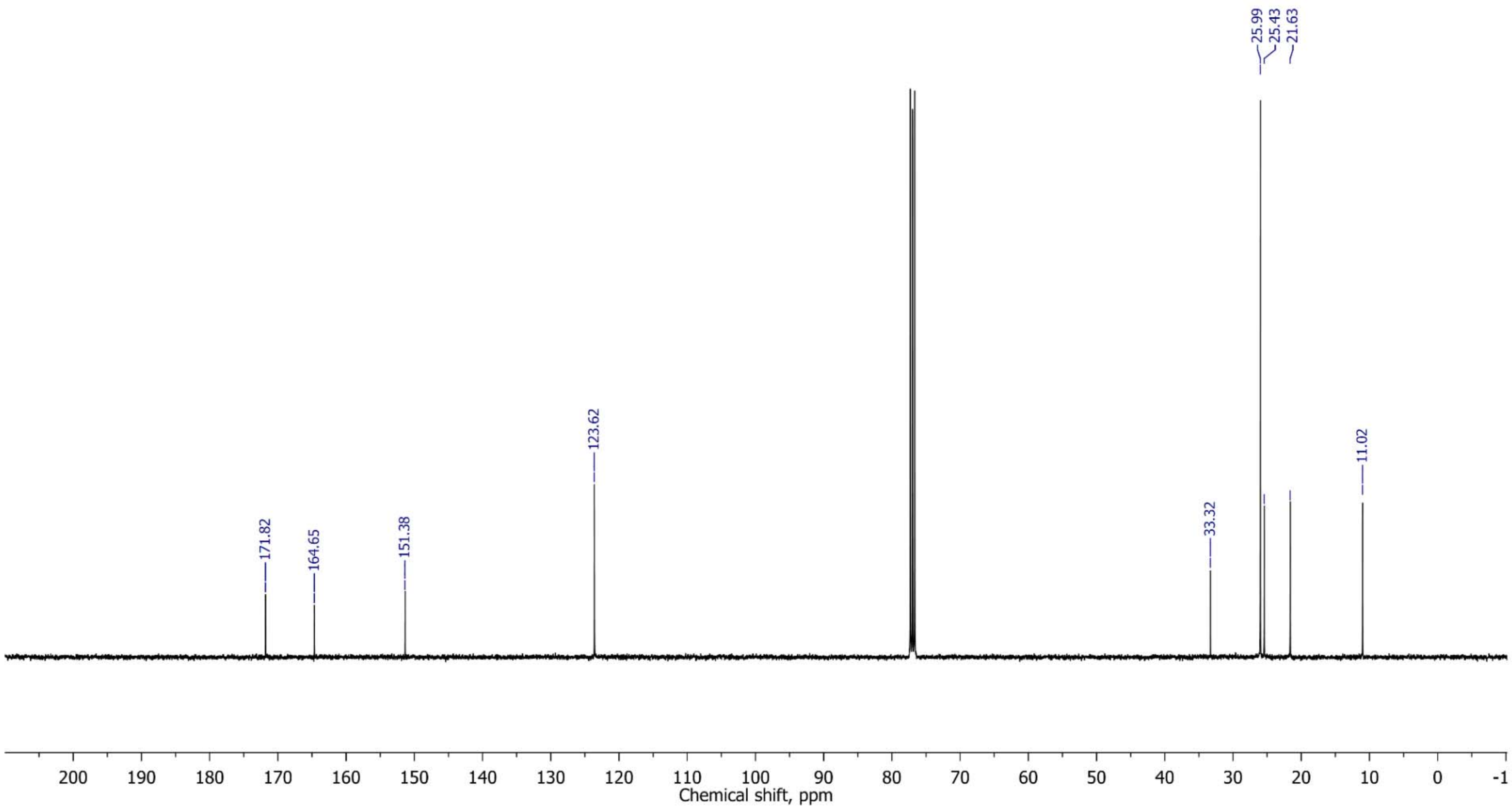

5-(3-(*tert*-Butyl)-2*H*-azirin-2-yl)-2-ethyloxazole 2f, DEPT, 100 MHz, $CDCl_3$

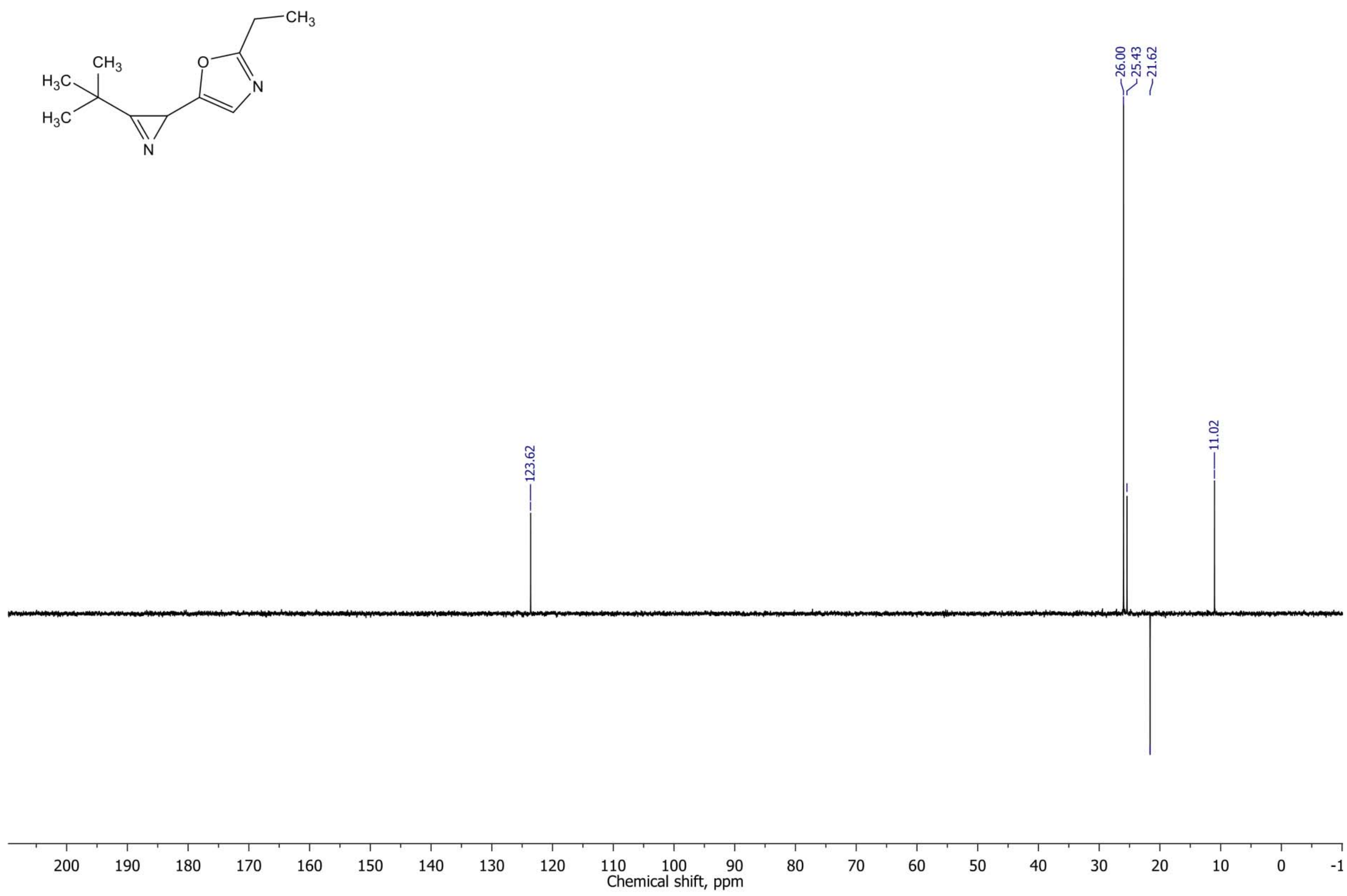

2-Benzyl-5-(3-(4-bromophenyl)-2*H*-azirin-2-yl)oxazole 2g, ^{1}H NMR, 400 MHz, $CDCl_3$

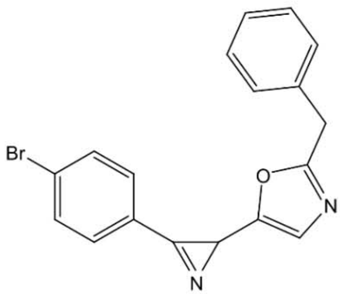

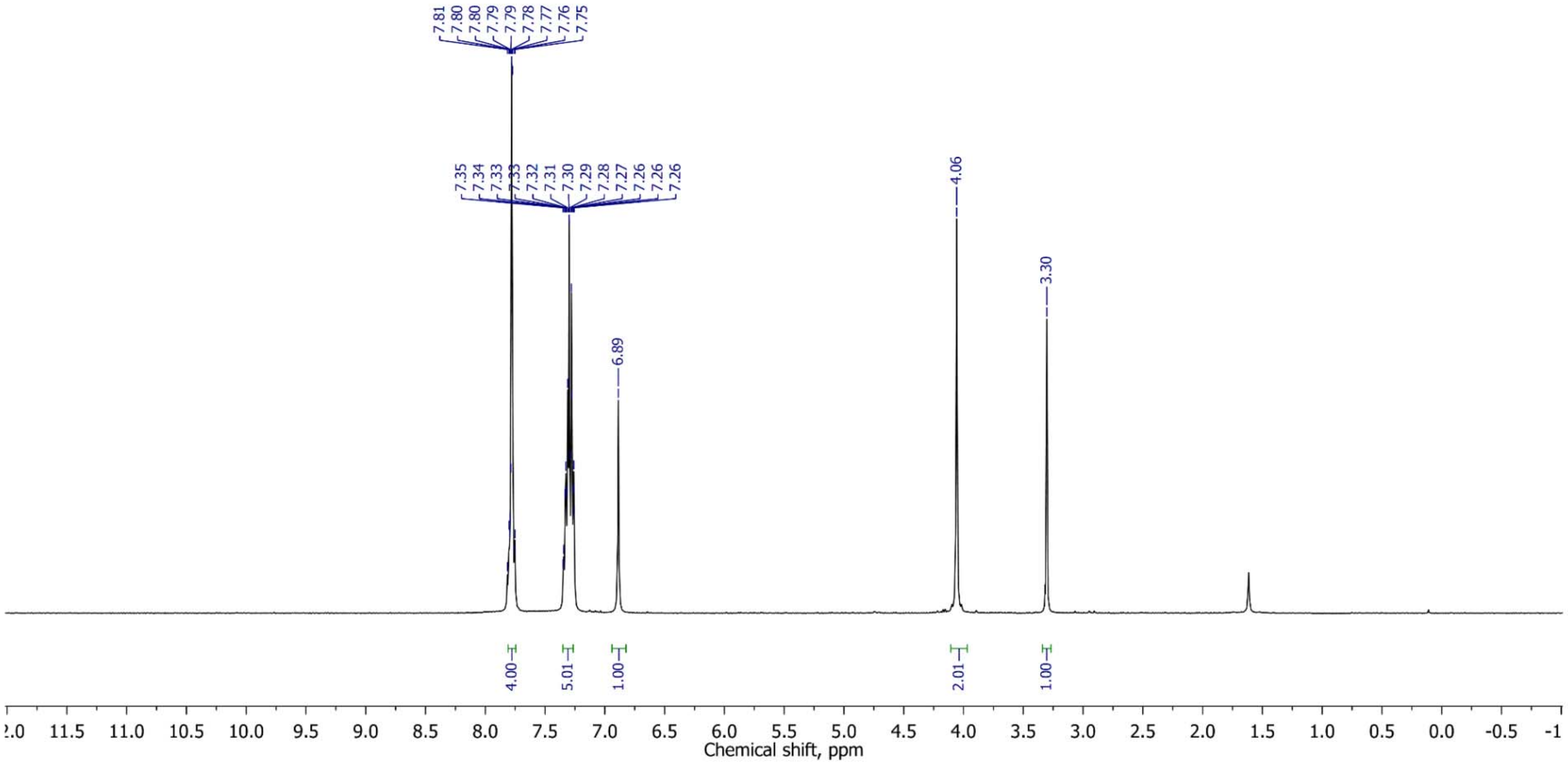

2-Benzyl-5-(3-(4-bromophenyl)-2*H*-azirin-2-yl)oxazole 2g, $^{13}C\{^{1}H\}$ NMR, 100 MHz, $CDCl_3$

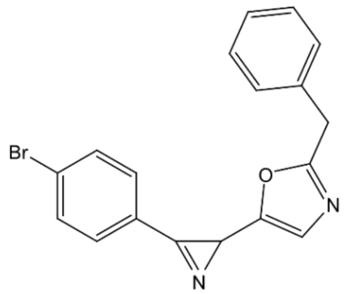

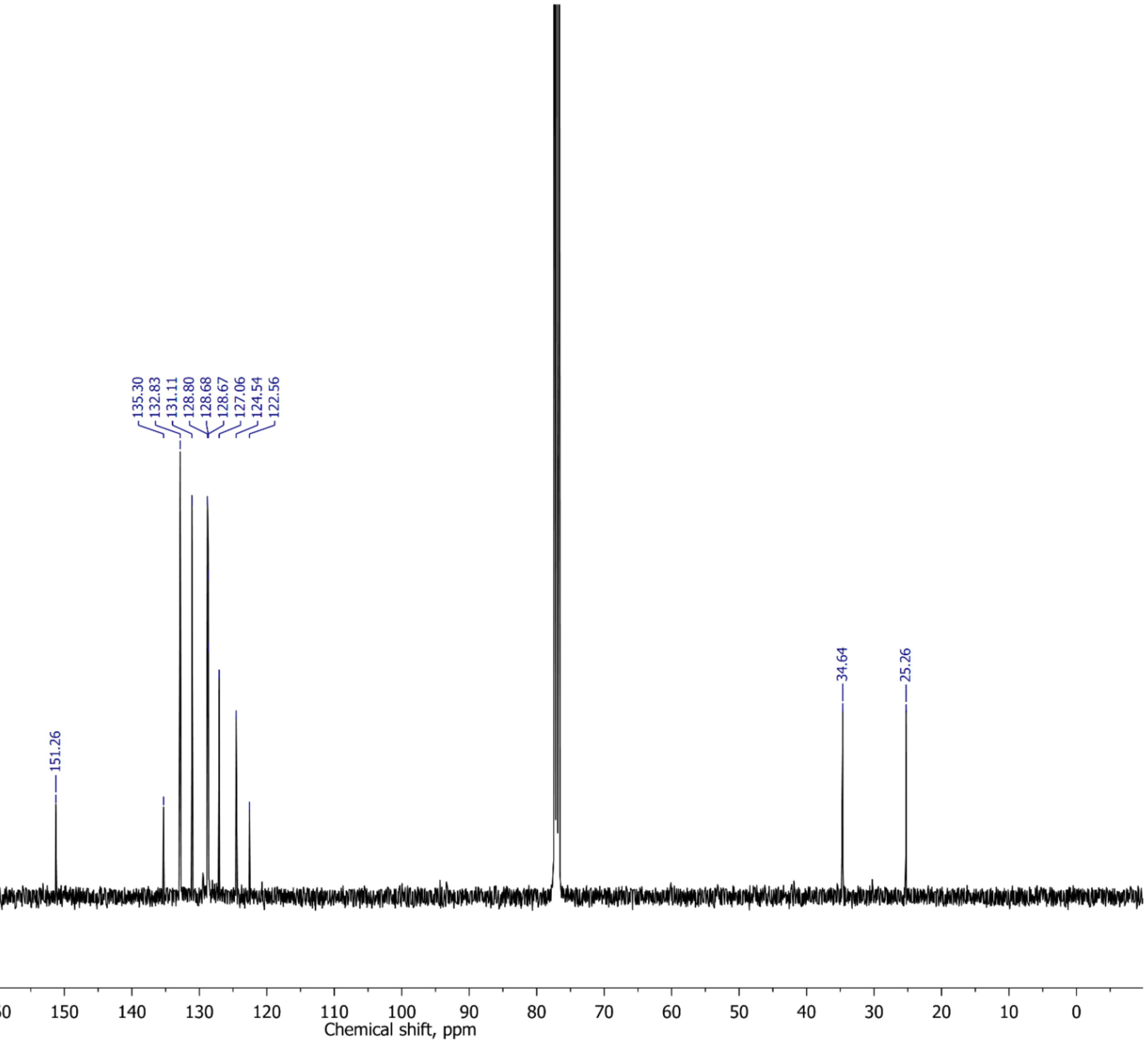

2-Benzyl-5-(3-(4-bromophenyl)-2*H*-azirin-2-yl)oxazole 2g, DEPT, 100 MHz, $CDCl_3$

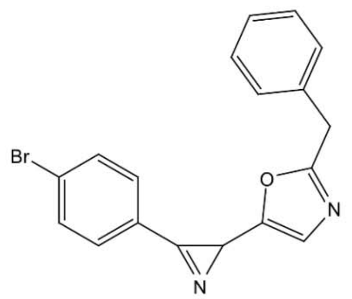

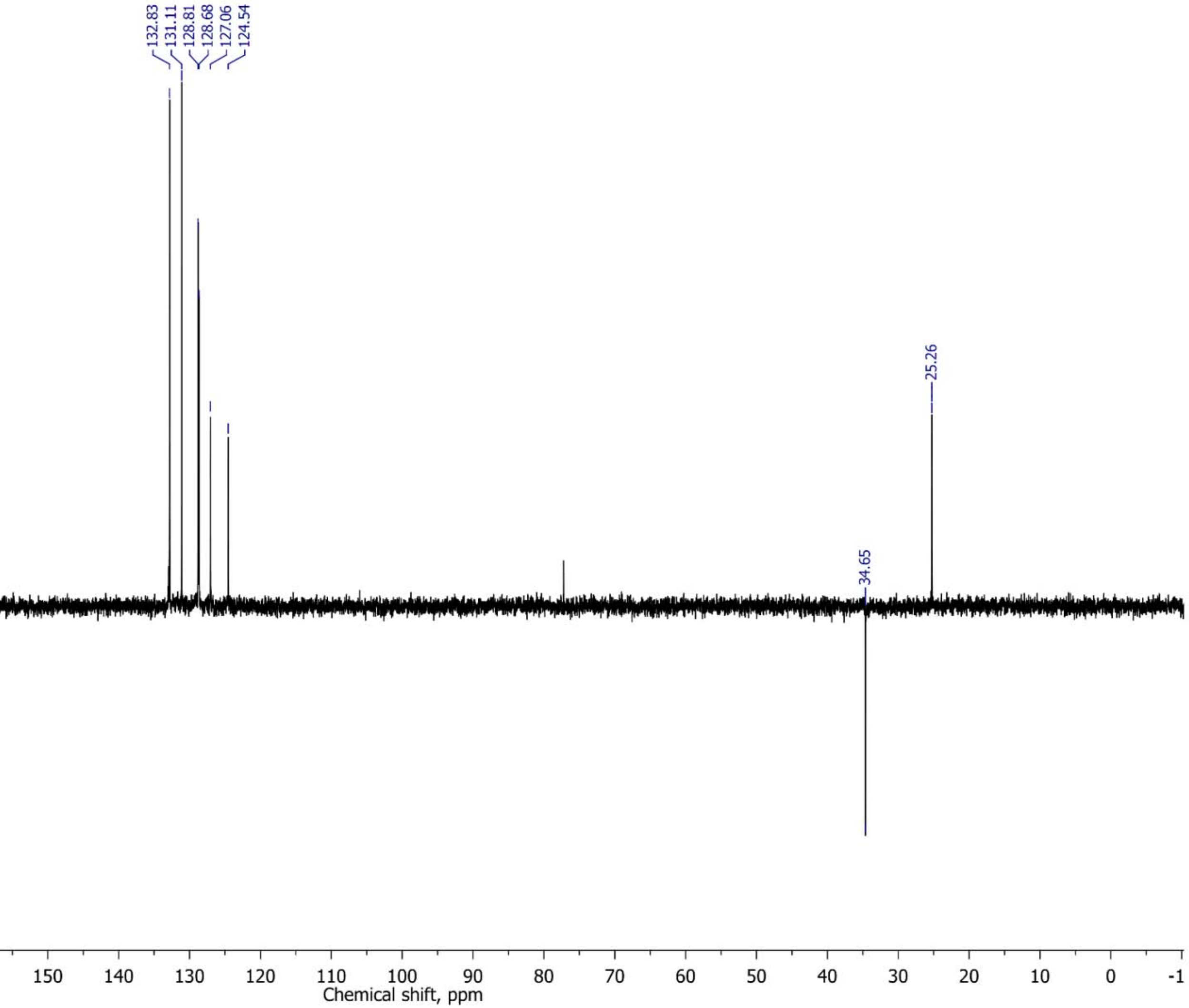

2-Phenyl-5-(3-phenyl-2*H*-azirin-2-yl)oxazole 2h, ^{1}H NMR, 400 MHz, $CDCl_3$

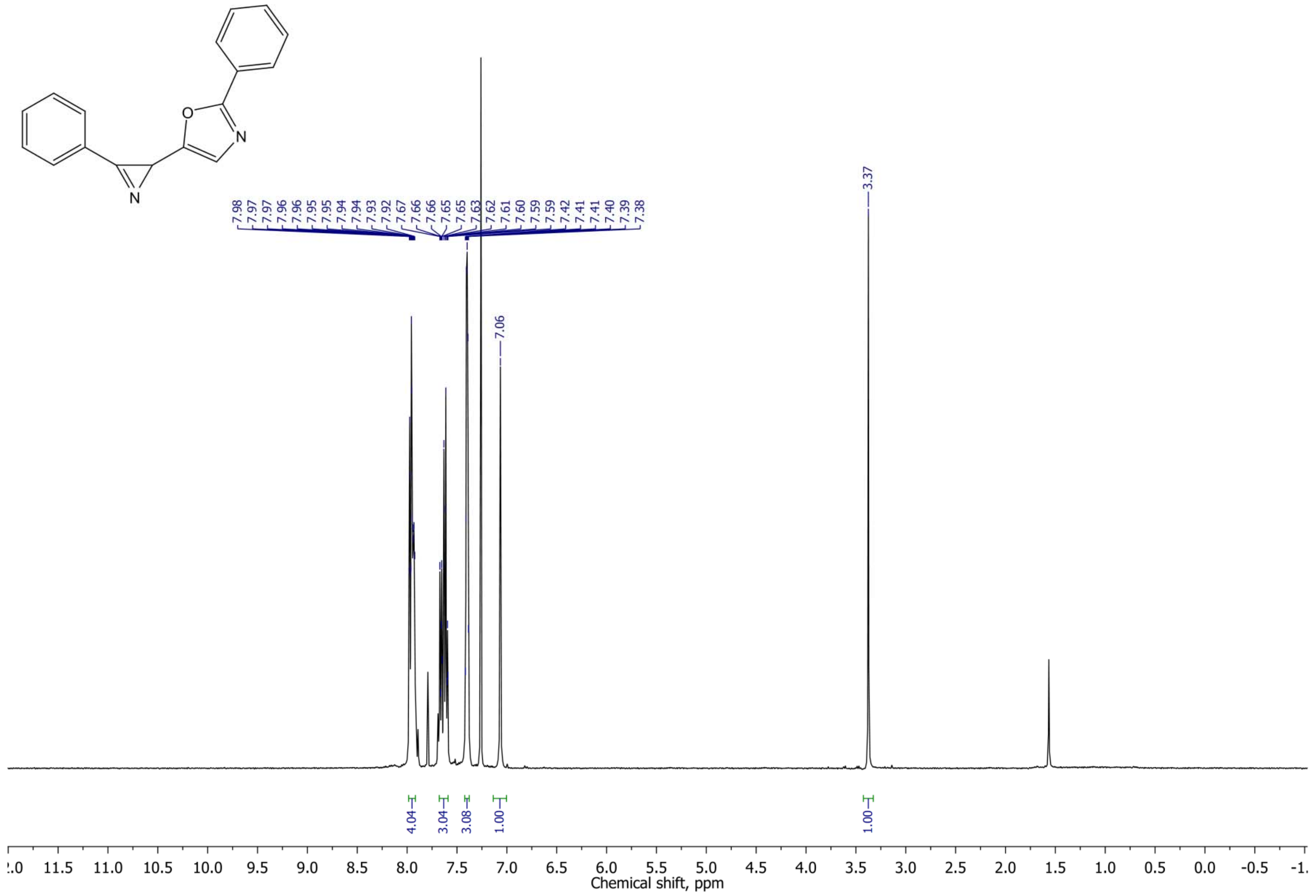

5-(3-(4-Fluorophenyl)-2*H*-azirin-2-yl)-2-phenyloxazole 2i, ^{1}H NMR, 400 MHz, $CDCl_3$

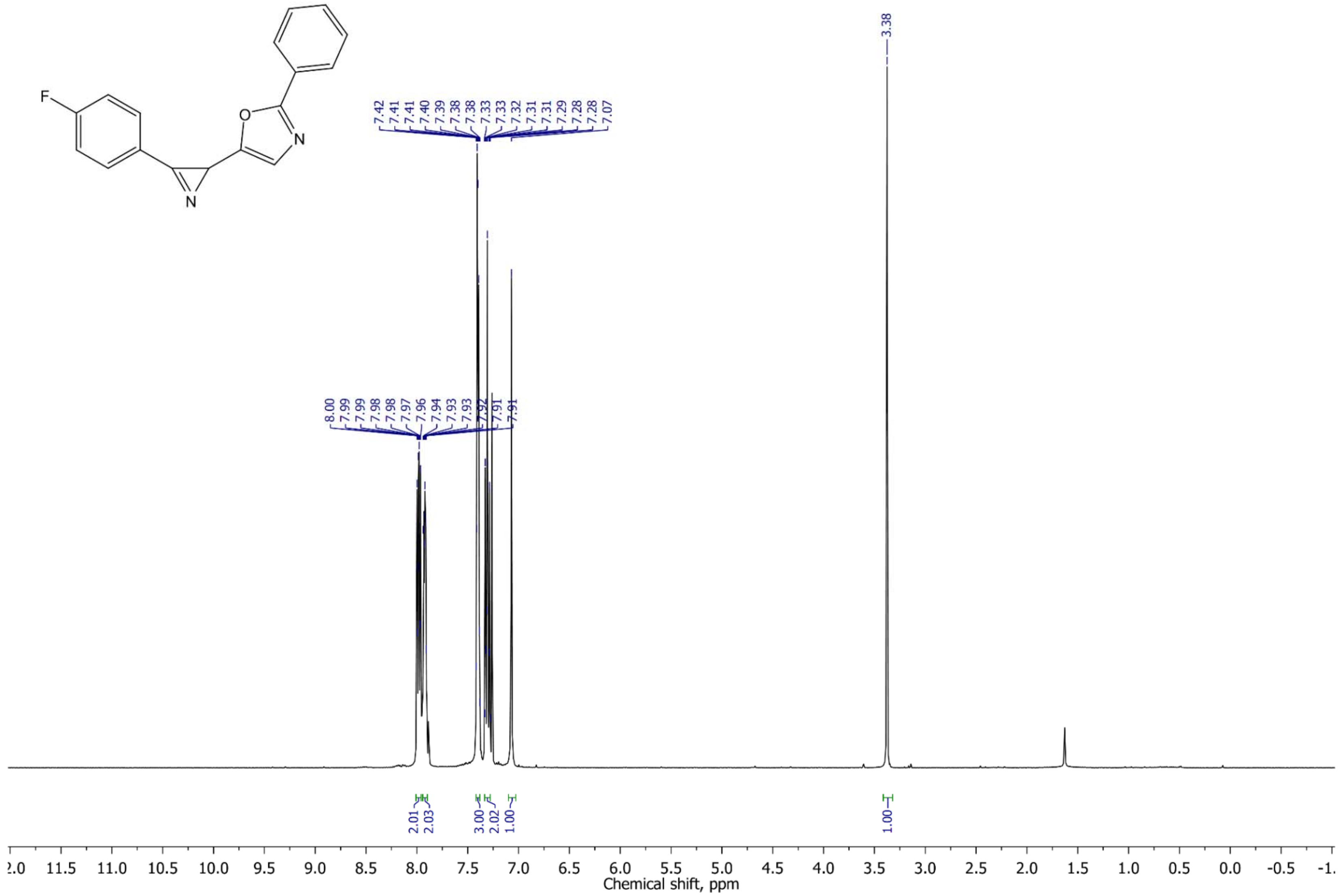

5-(3-(4-Fluorophenyl)-2*H*-azirin-2-yl)-2-phenyloxazole 2i, $^{13}C\{^{1}H\}$ NMR, 100 MHz, $CDCl_3$

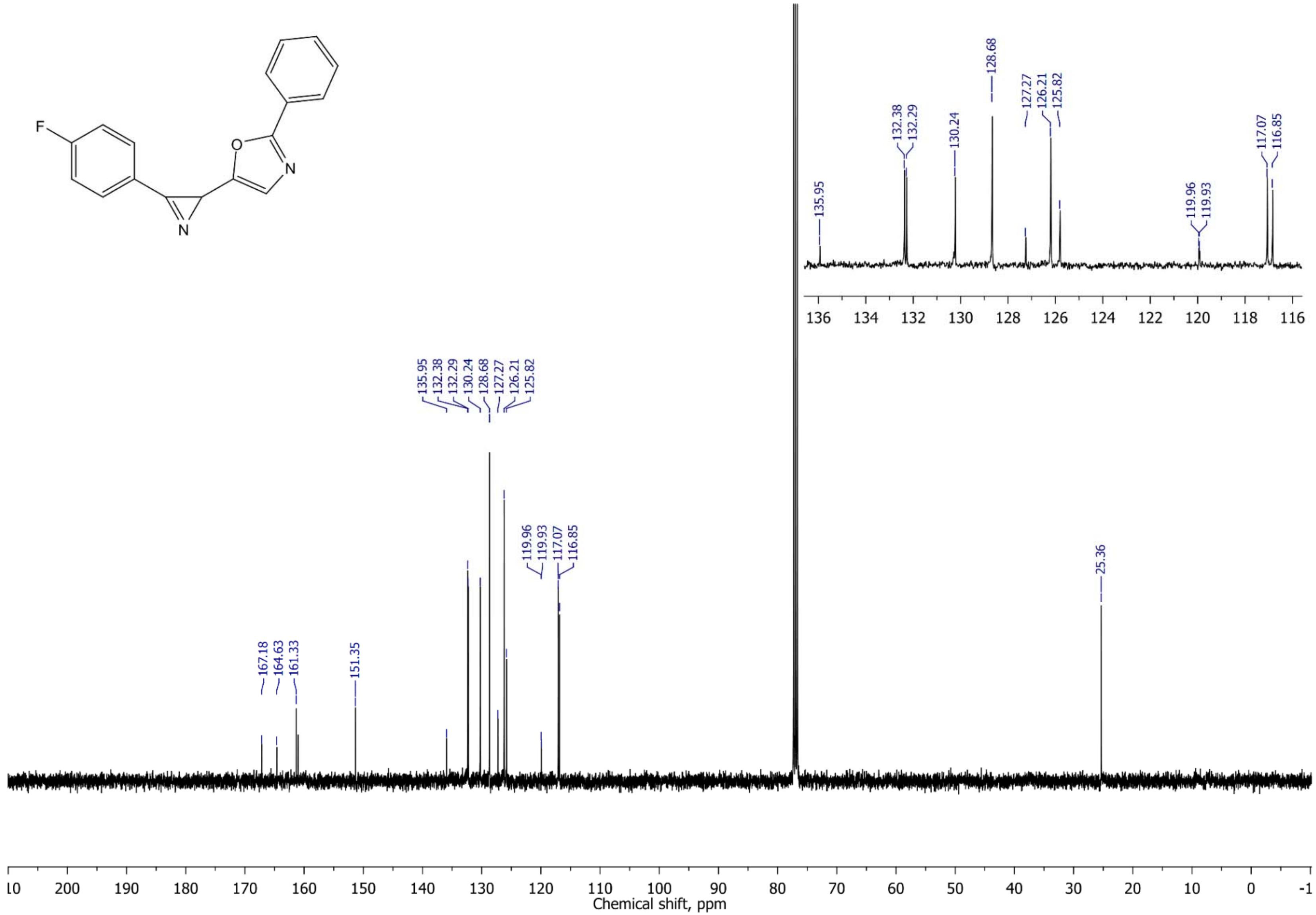

5-(3-(4-Fluorophenyl)-2*H*-azirin-2-yl)-2-phenyloxazole 2i, DEPT, 100 MHz, CDCl$_3$

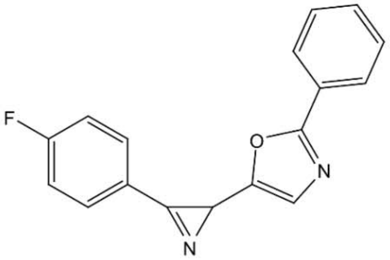

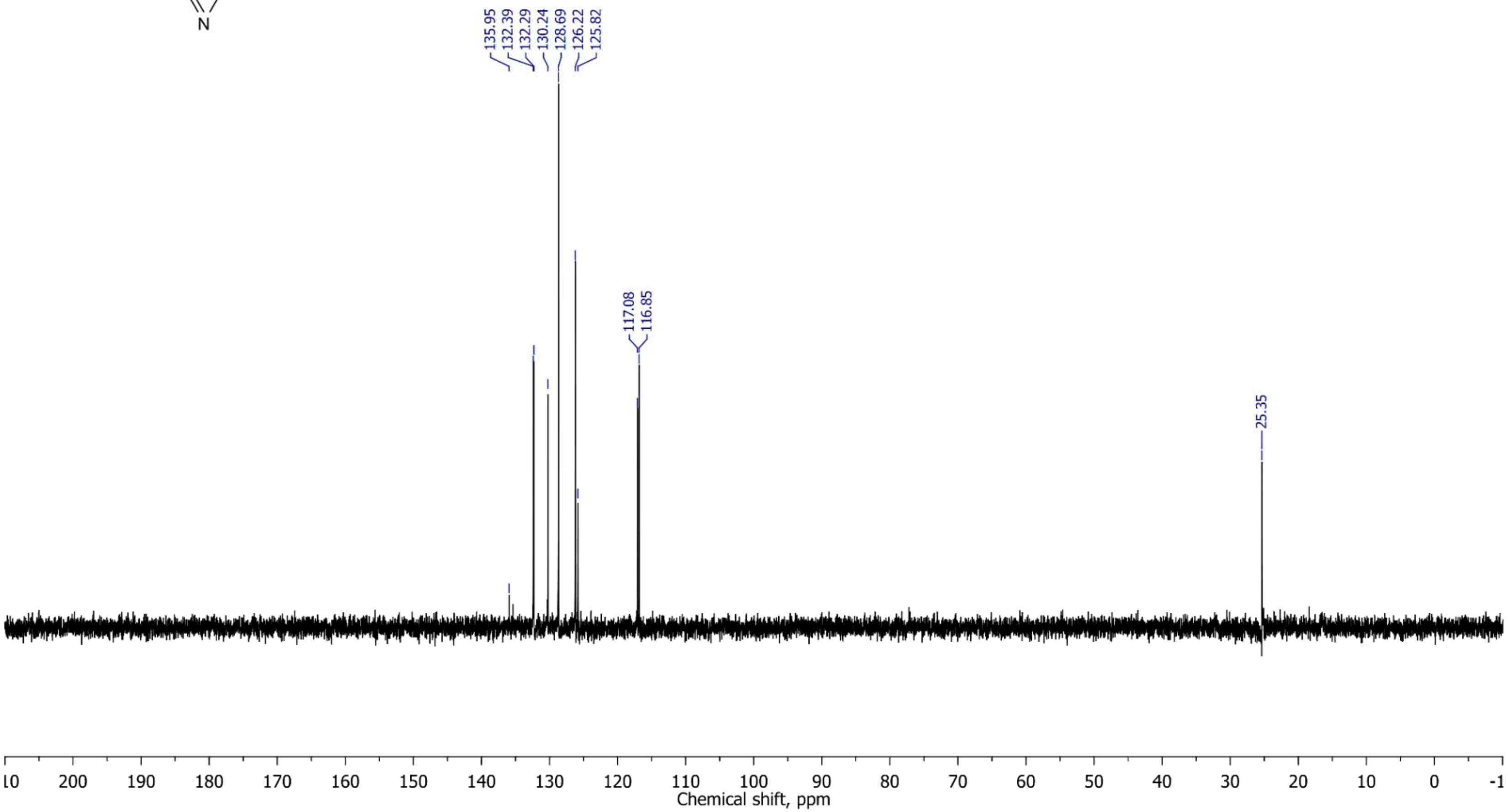

5-(3-(*tert*-Butyl)-2*H*-azirin-2-yl)-2-phenyloxazole 2j, ^{1}H NMR, 400 MHz, $CDCl_3$

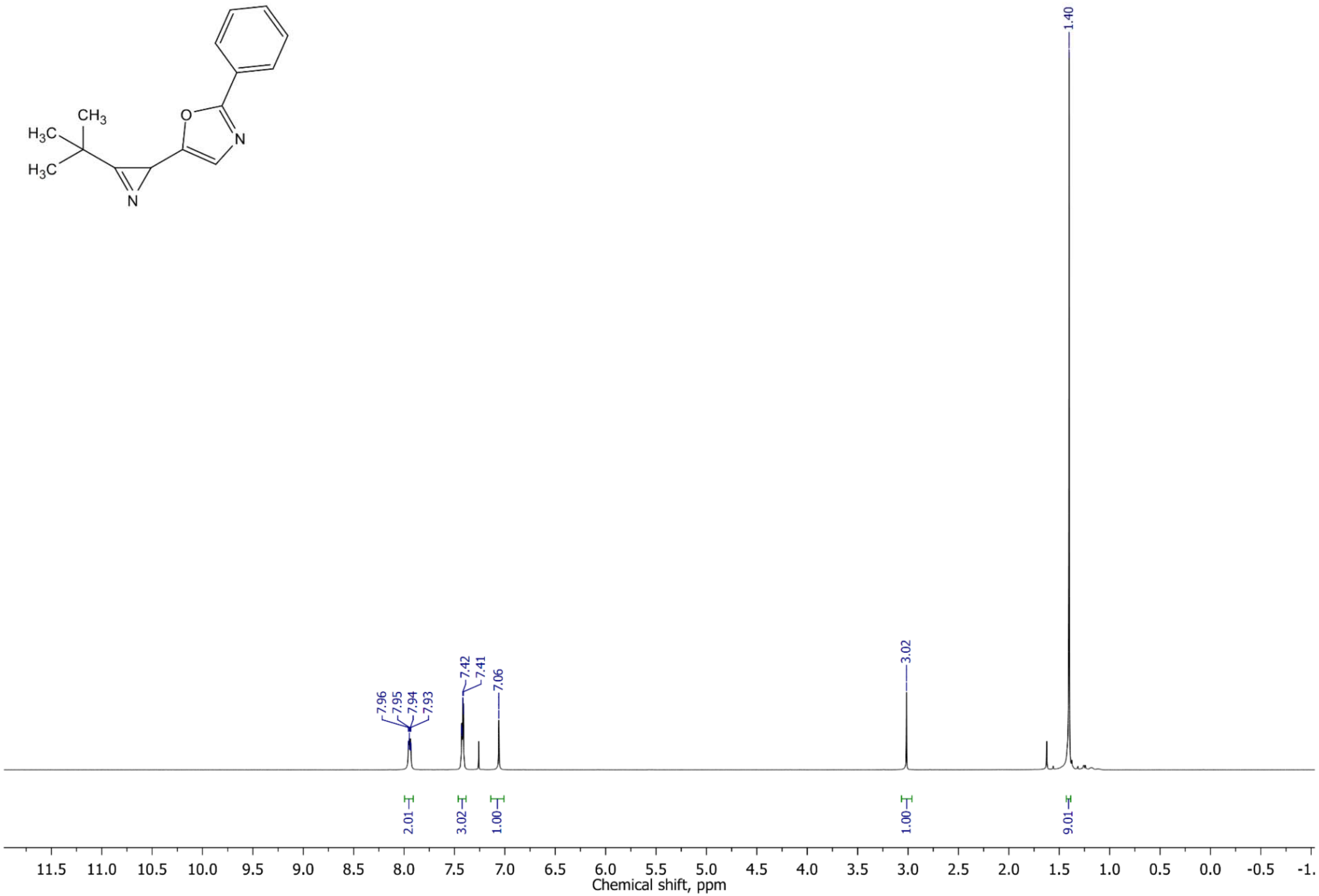

5-(3-(*tert*-Butyl)-2*H*-azirin-2-yl)-2-phenyloxazole 2j, $^{13}C\{^{1}H\}$ NMR, 100 MHz, $CDCl_3$

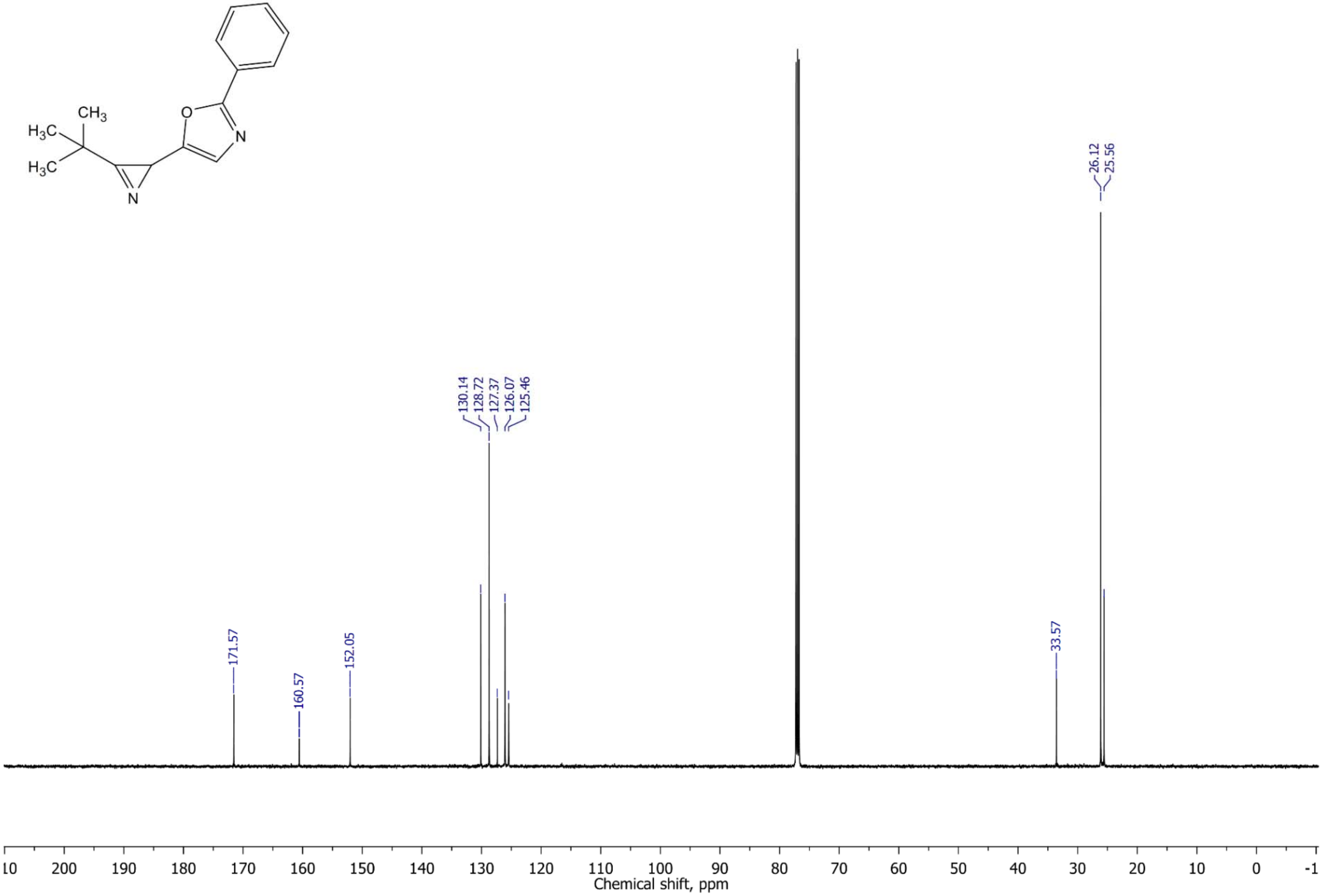

5-(3-(*tert*-Butyl)-2*H*-azirin-2-yl)-2-phenyloxazole 2j, DEPT, 100 MHz, $CDCl_3$

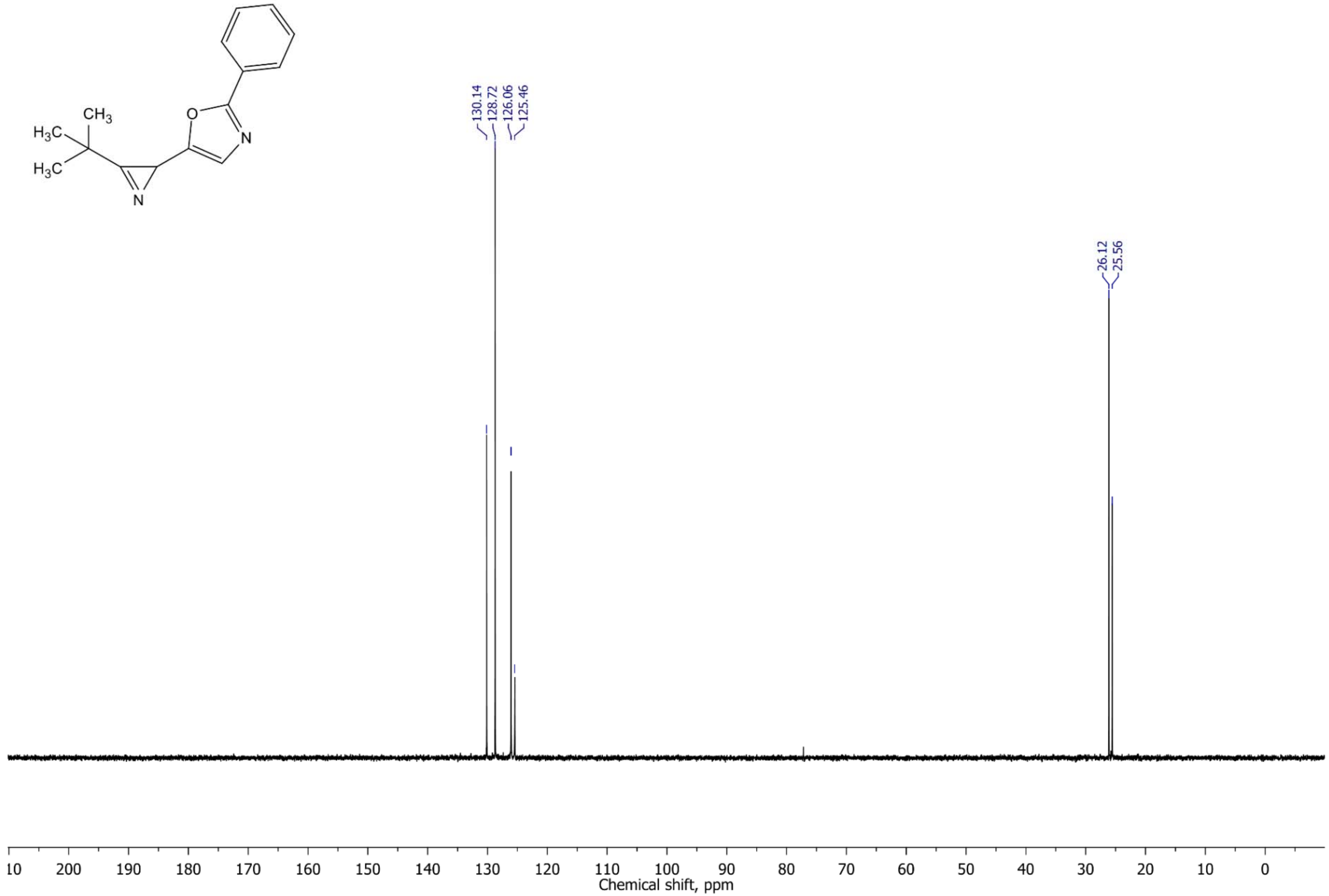

5-(3-(Adamantan-1-yl)-2*H*-azirin-2-yl)-2-phenyloxazole 2k, ^{1}H NMR, 400 MHz, $CDCl_3$

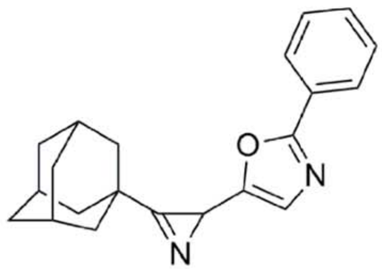

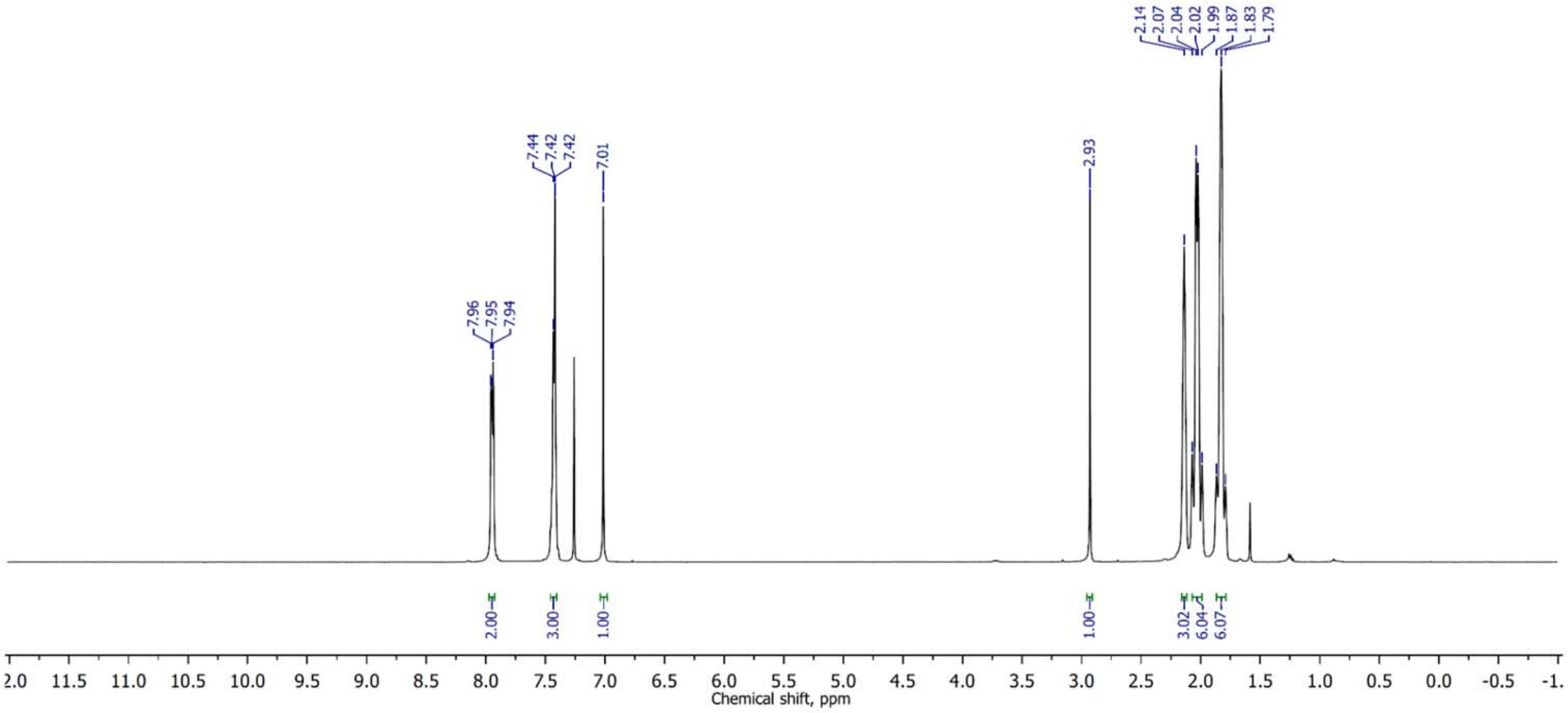

5-(3-(Adamantan-1-yl)-2*H*-azirin-2-yl)-2-phenyloxazole 2k, $^{13}C\{^{1}H\}$ NMR, 100 MHz, $CDCl_3$

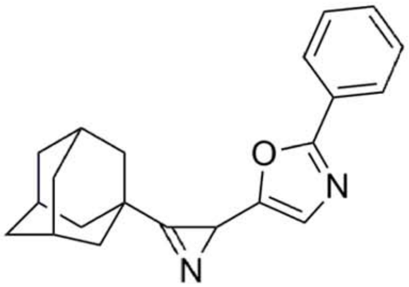

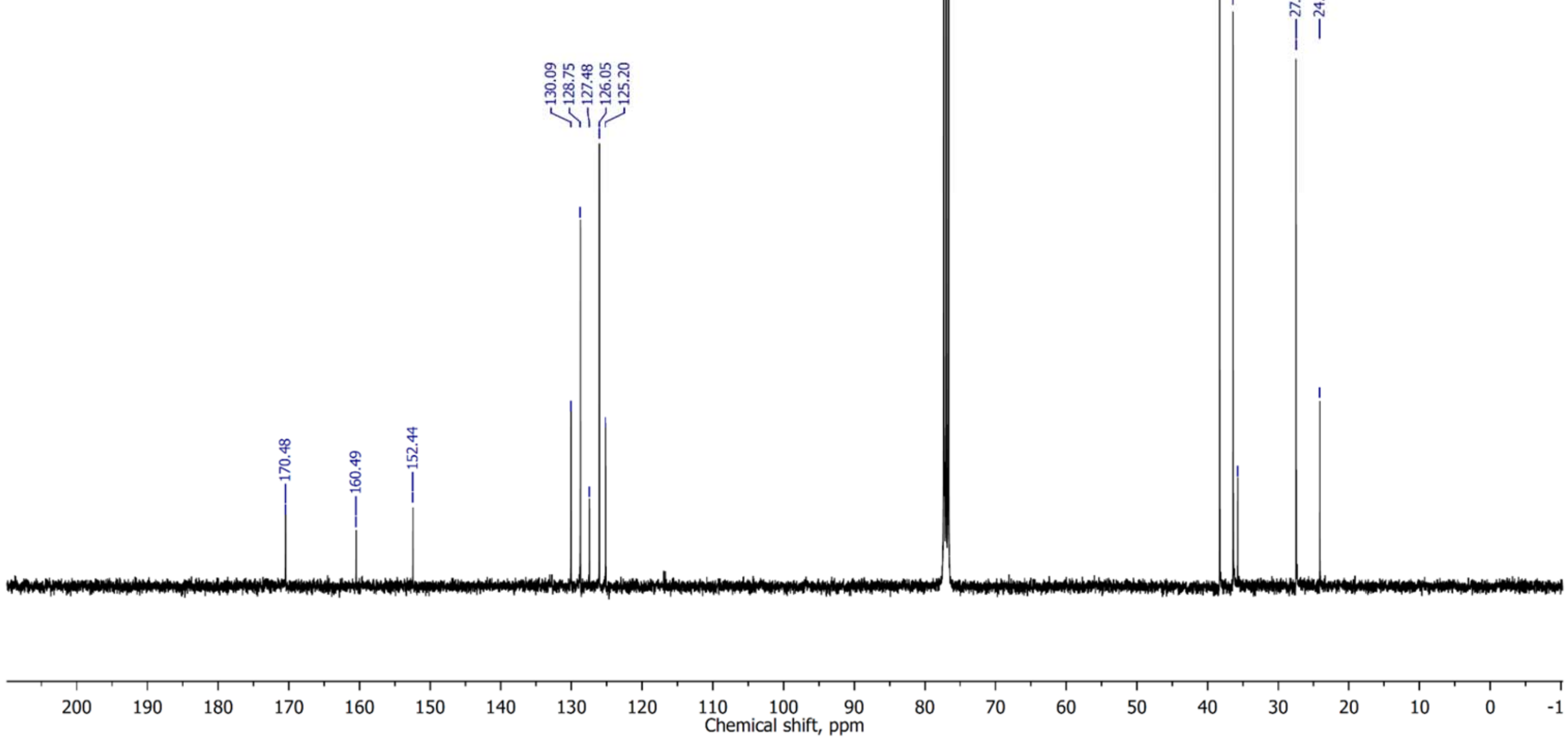

5-(3-(Adamantan-1-yl)-2*H*-azirin-2-yl)-2-phenyloxazole 2k, DEPT, 100 MHz, $CDCl_3$

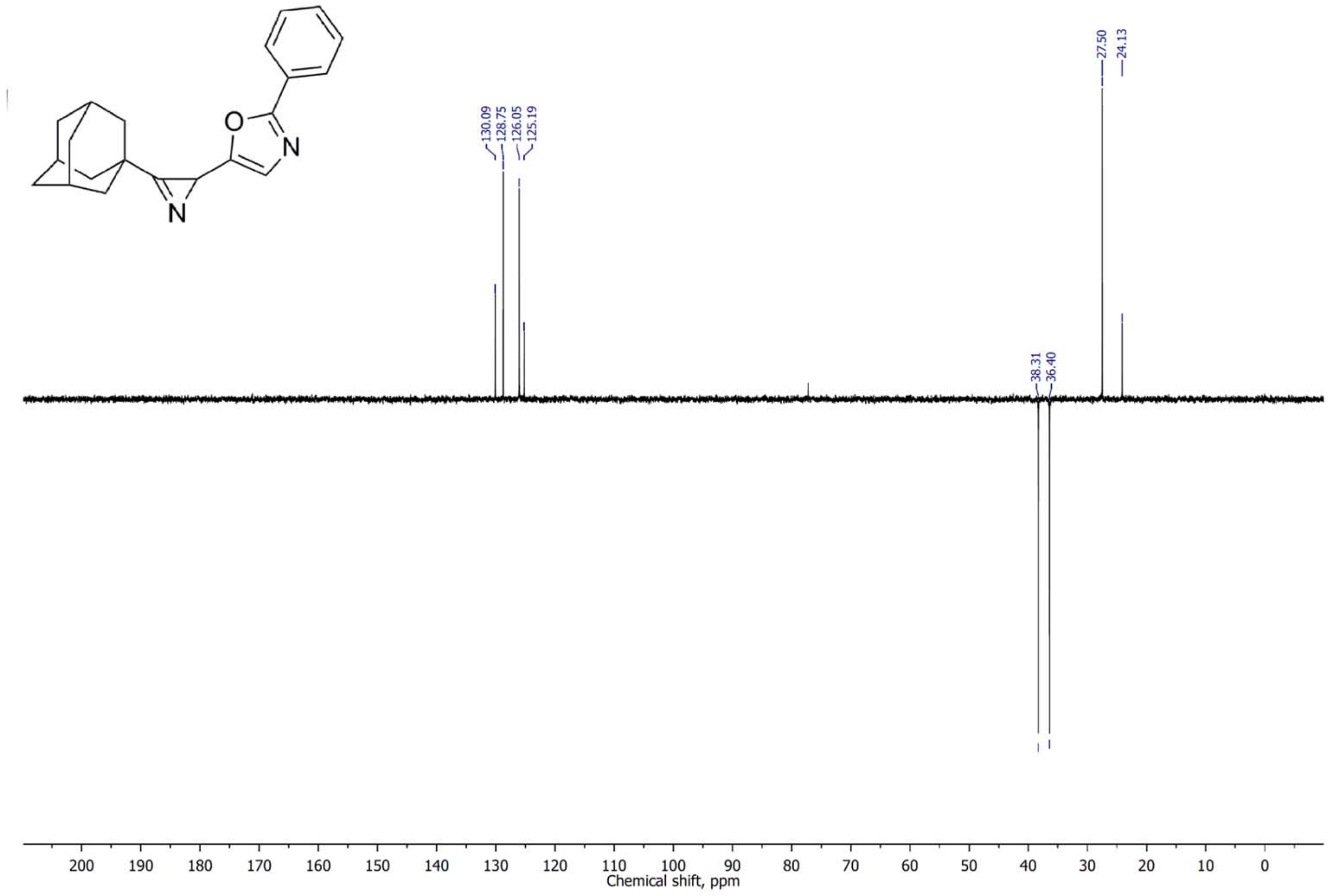

5-(3-(4-Methoxyphenyl)-2*H*-azirin-2-yl)-2-(*p*-tolyl)oxazole 2l, ^{1}H NMR, 400 MHz, $CDCl_3$

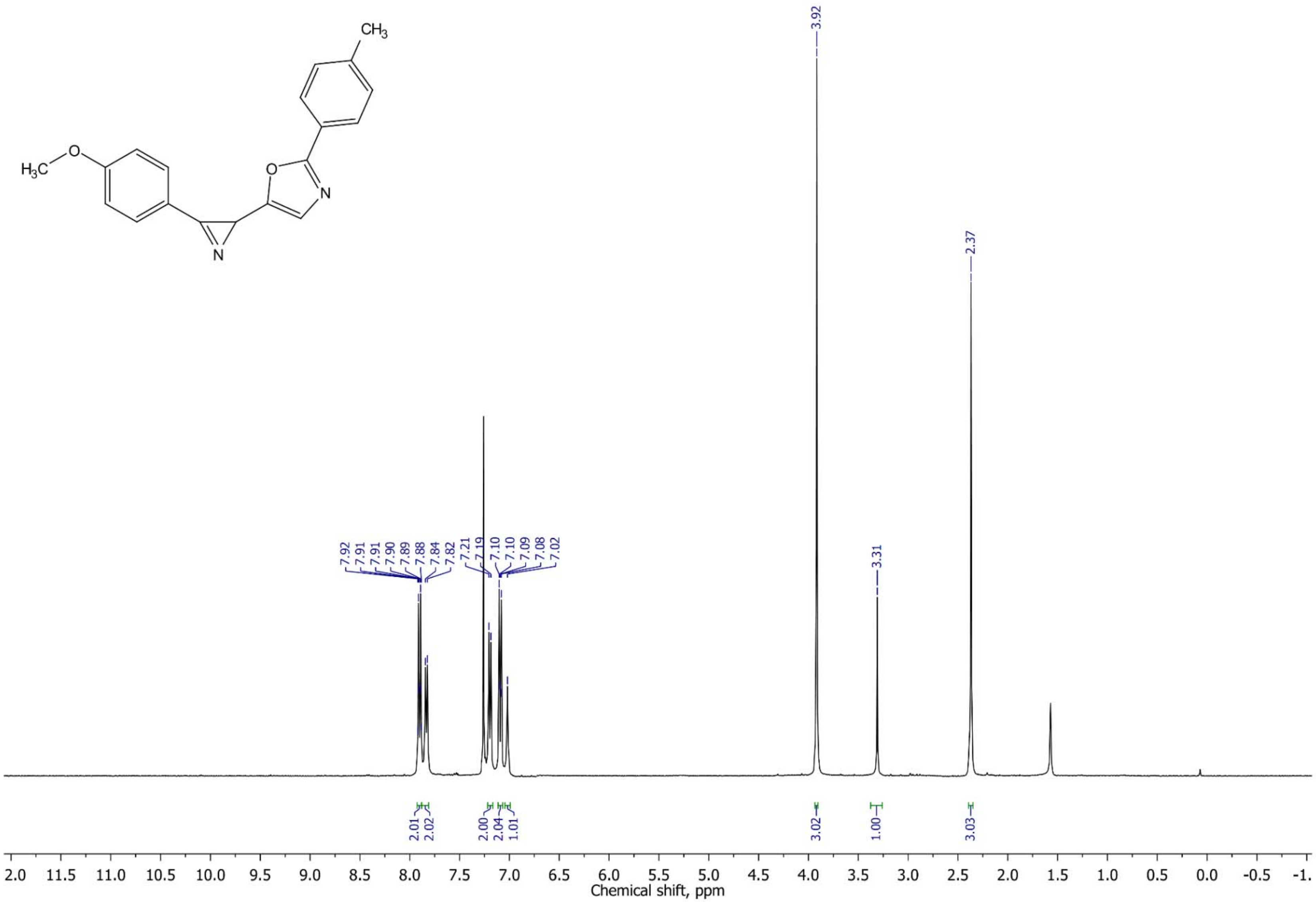

5-(3-(4-Methoxyphenyl)-2*H*-azirin-2-yl)-2-(*p*-tolyl)oxazole 2l, $^{13}C\{^1H\}$ NMR, 100 MHz, $CDCl_3$

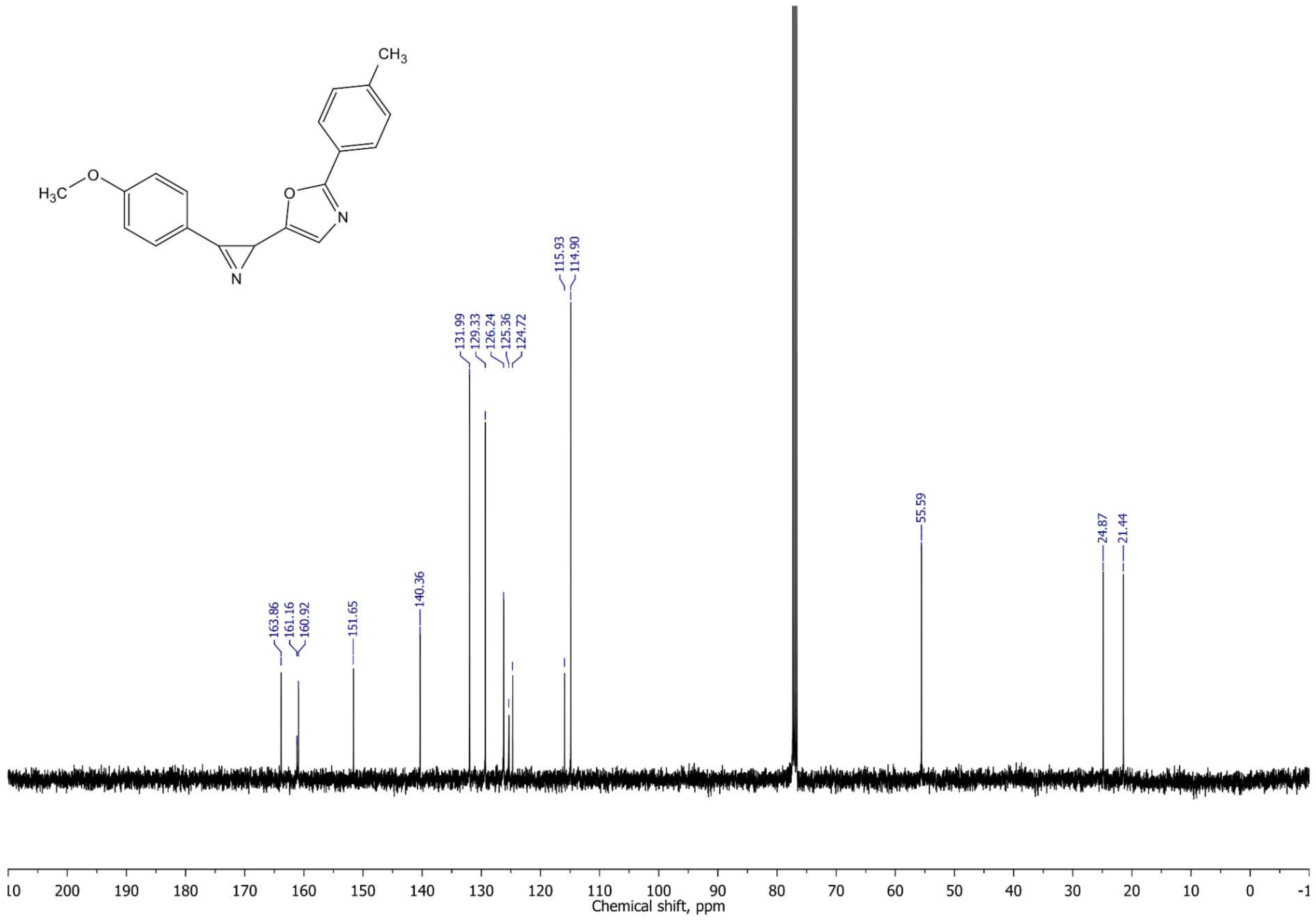

5-(3-(4-Methoxyphenyl)-2*H*-azirin-2-yl)-2-(*p*-tolyl)oxazole 2l, DEPT, 100 MHz, $CDCl_3$

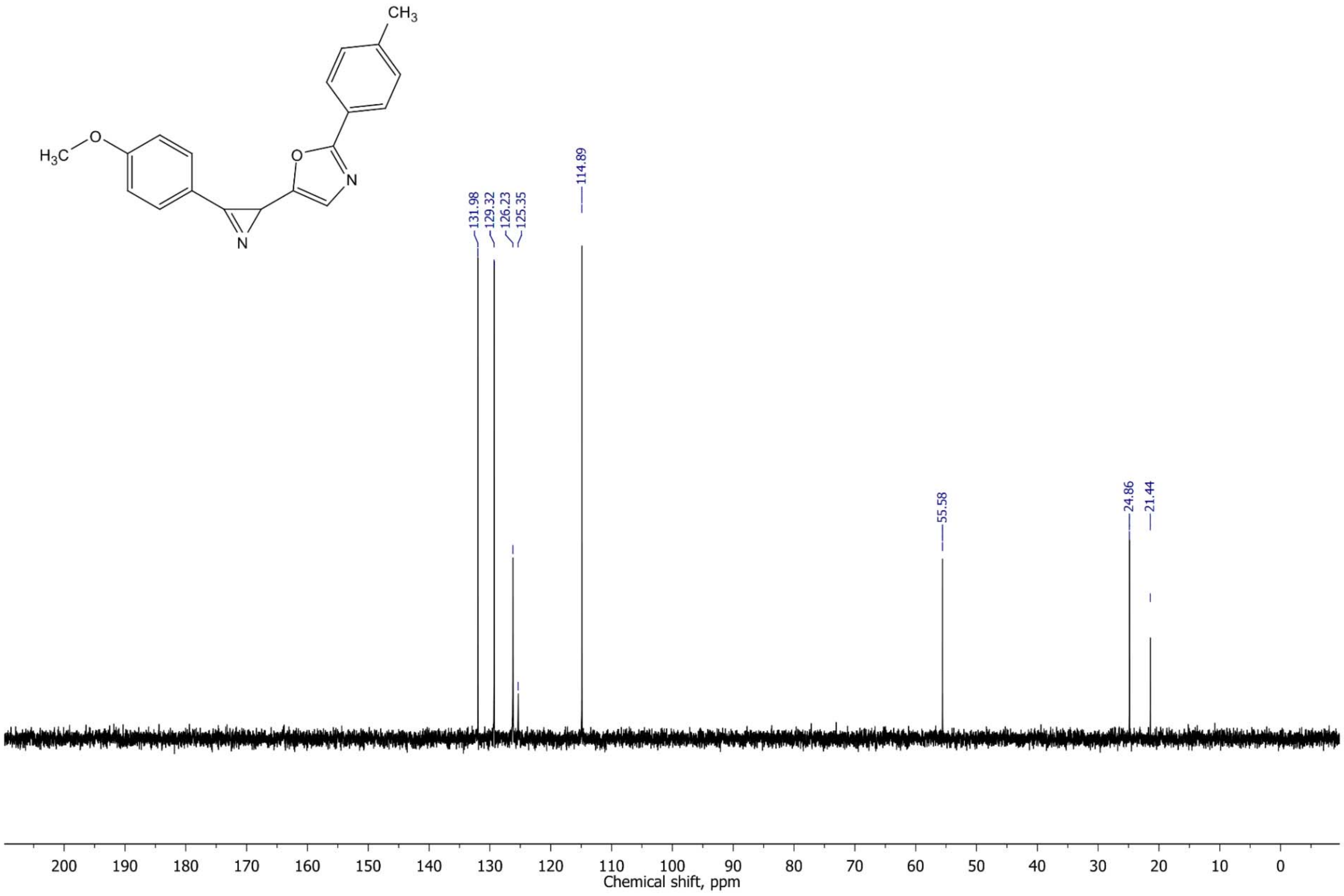

5-(3-(4-Chlorophenyl)-2*H*-azirin-2-yl)-2-(*p*-tolyl)oxazole 2m, ^{1}H NMR, 400 MHz, $CDCl_3$

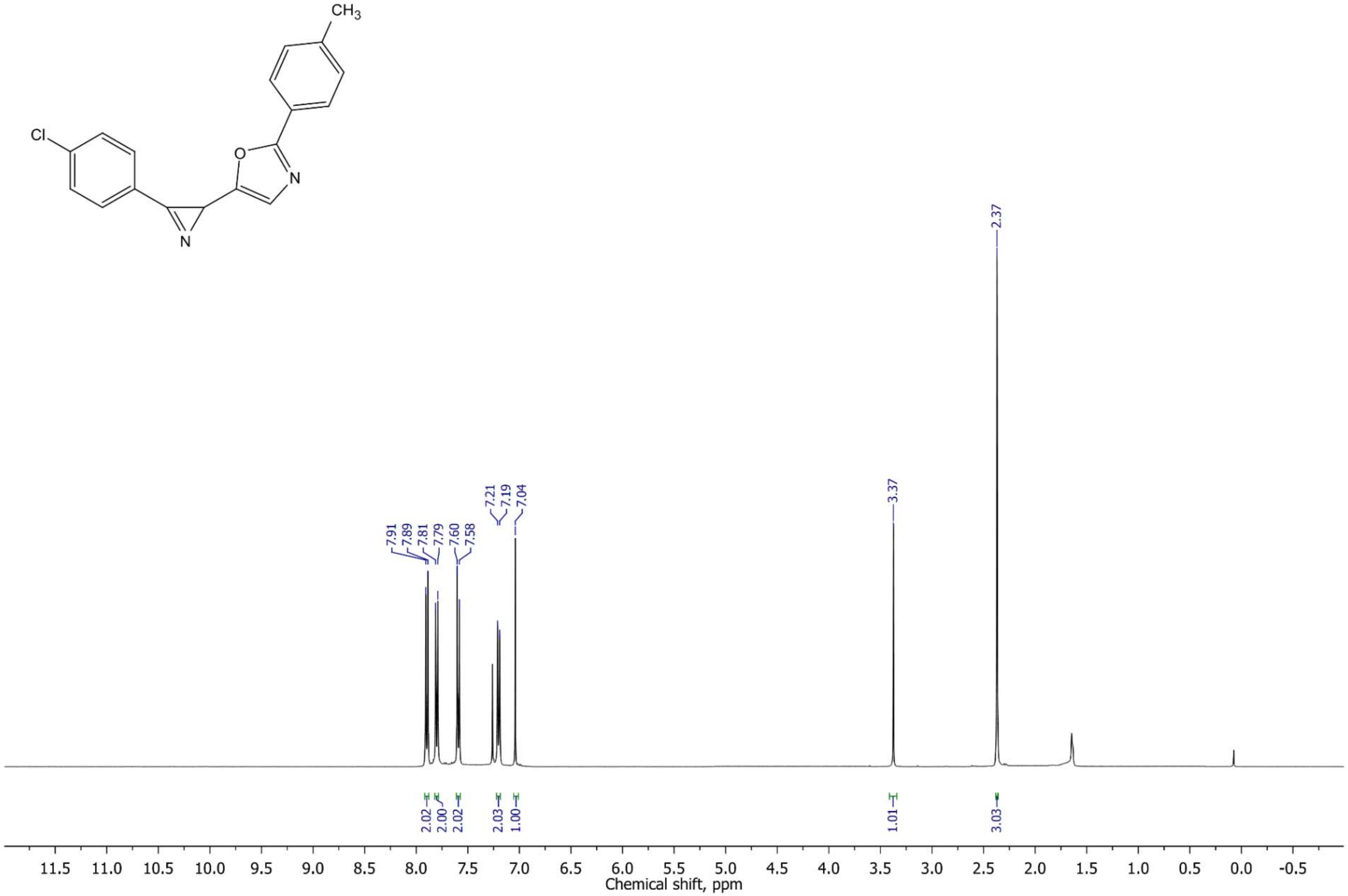

5-(3-(4-Chlorophenyl)-2*H*-azirin-2-yl)-2-(*p*-tolyl)oxazole 2m, $^{13}C\{^{1}H\}$ NMR, 100 MHz, $CDCl_3$

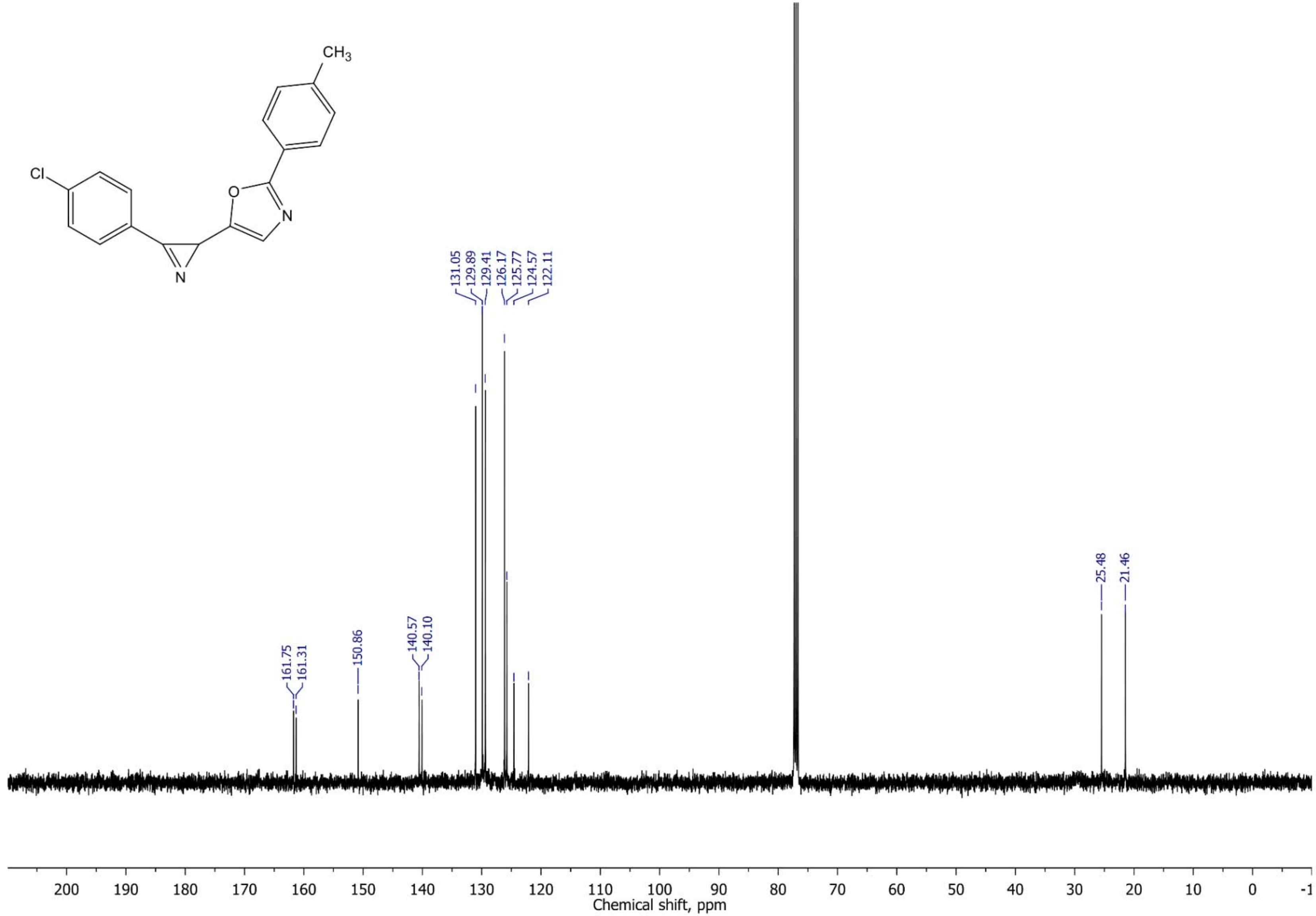

5-(3-(4-Chlorophenyl)-2*H*-azirin-2-yl)-2-(*p*-tolyl)oxazole 2m, DEPT, 100 MHz, $CDCl_3$

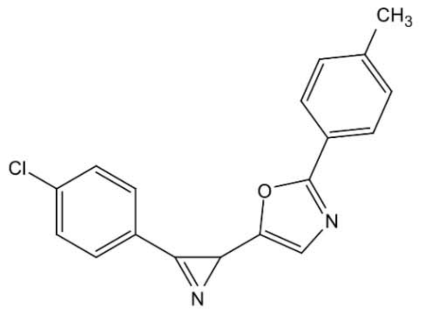

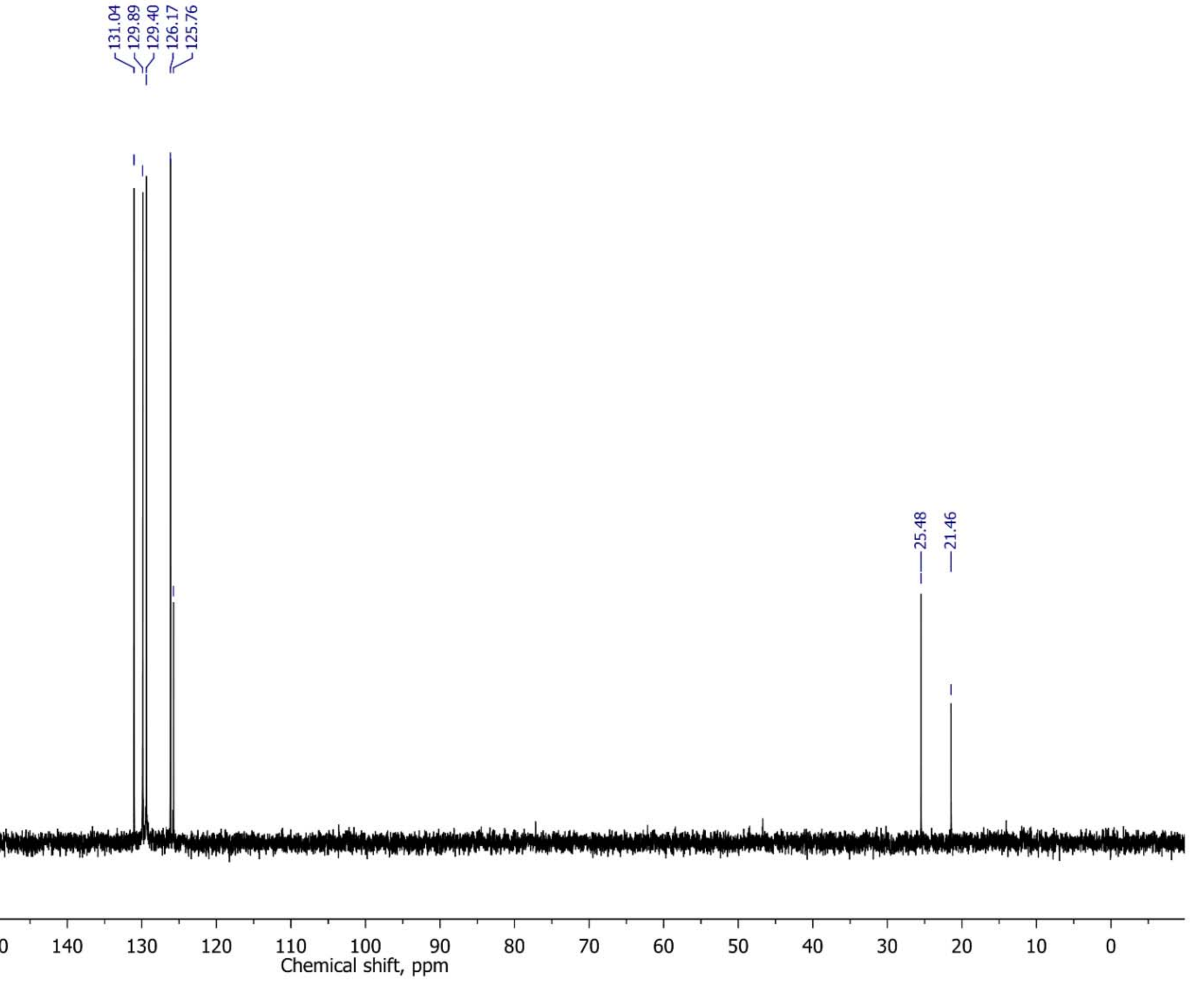

5-(3-(Adamantan-1-yl)-2*H*-azirin-2-yl)-2-(*p*-tolyl)oxazole 2n, ^{1}H NMR, 400 MHz, $CDCl_3$

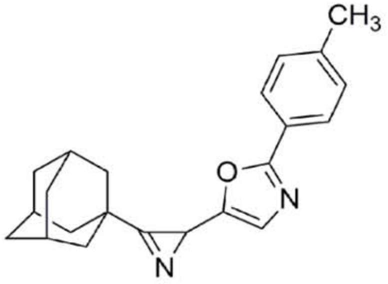

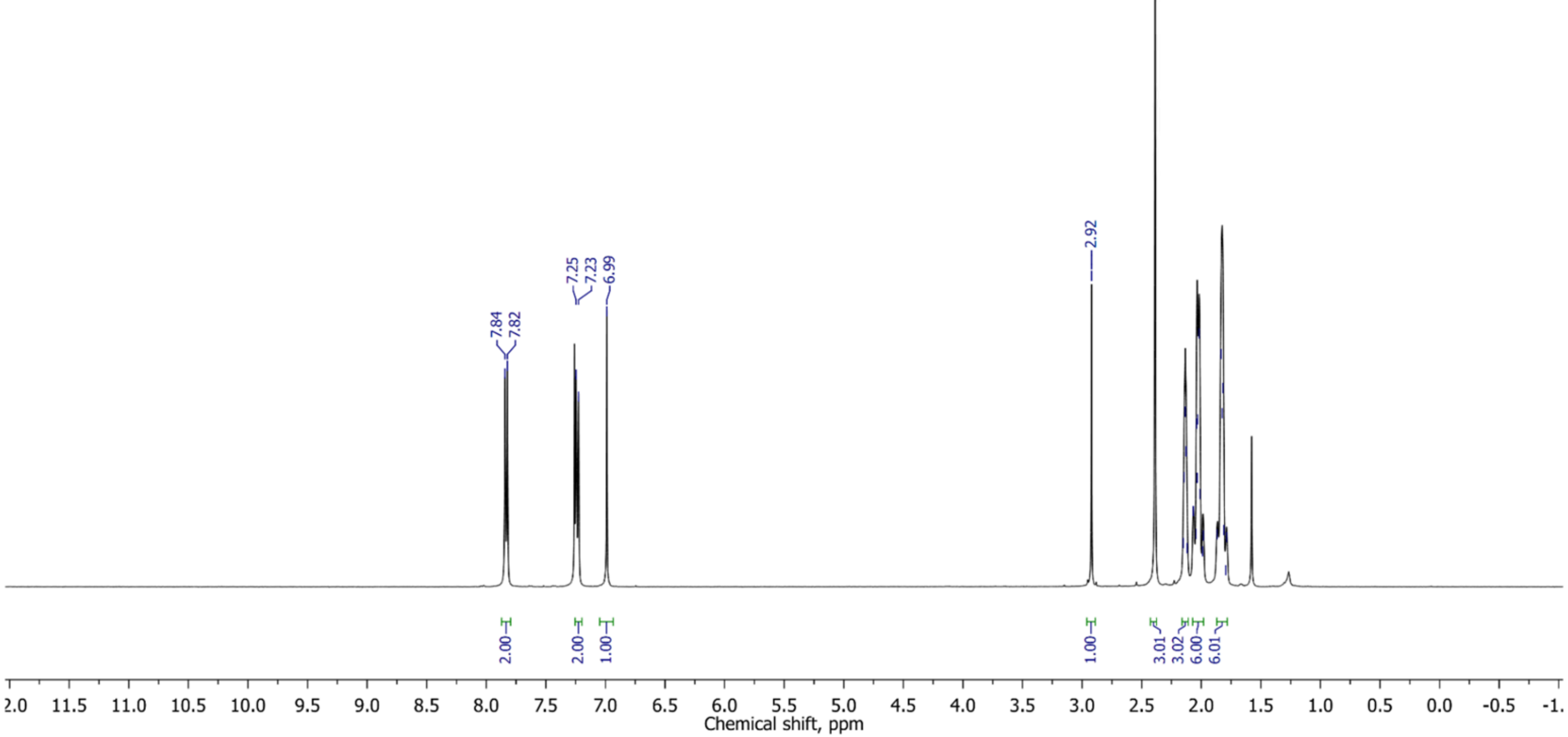

5-(3-(Adamantan-1-yl)-2*H*-azirin-2-yl)-2-(*p*-tolyl)oxazole 2n, $^{13}C\{^{1}H\}$ NMR, 100 MHz, $CDCl_3$

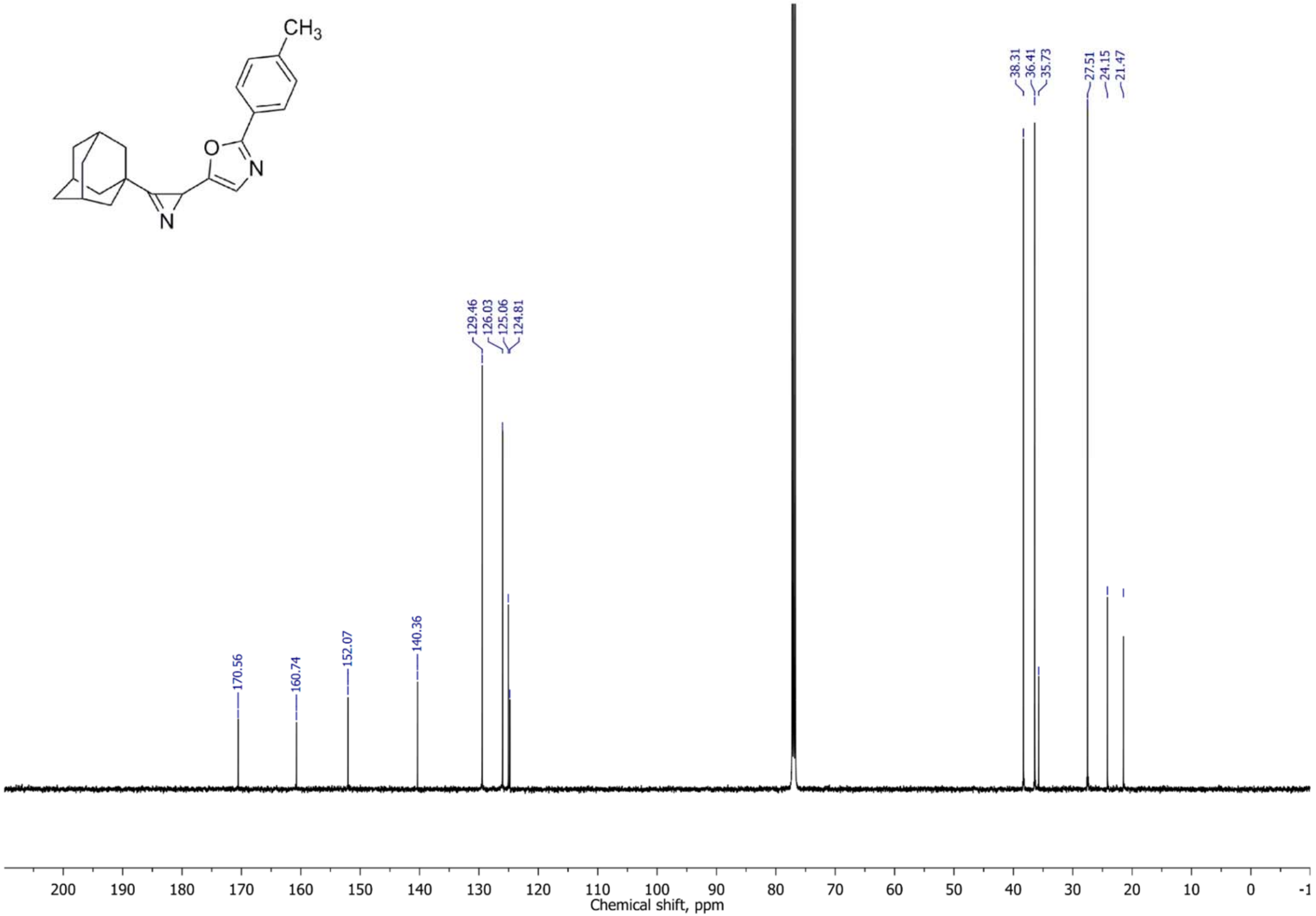

5-(3-(Adamantan-1-yl)-2*H*-azirin-2-yl)-2-(*p*-tolyl)oxazole 2n, DEPT NMR, 100 MHz, $CDCl_3$

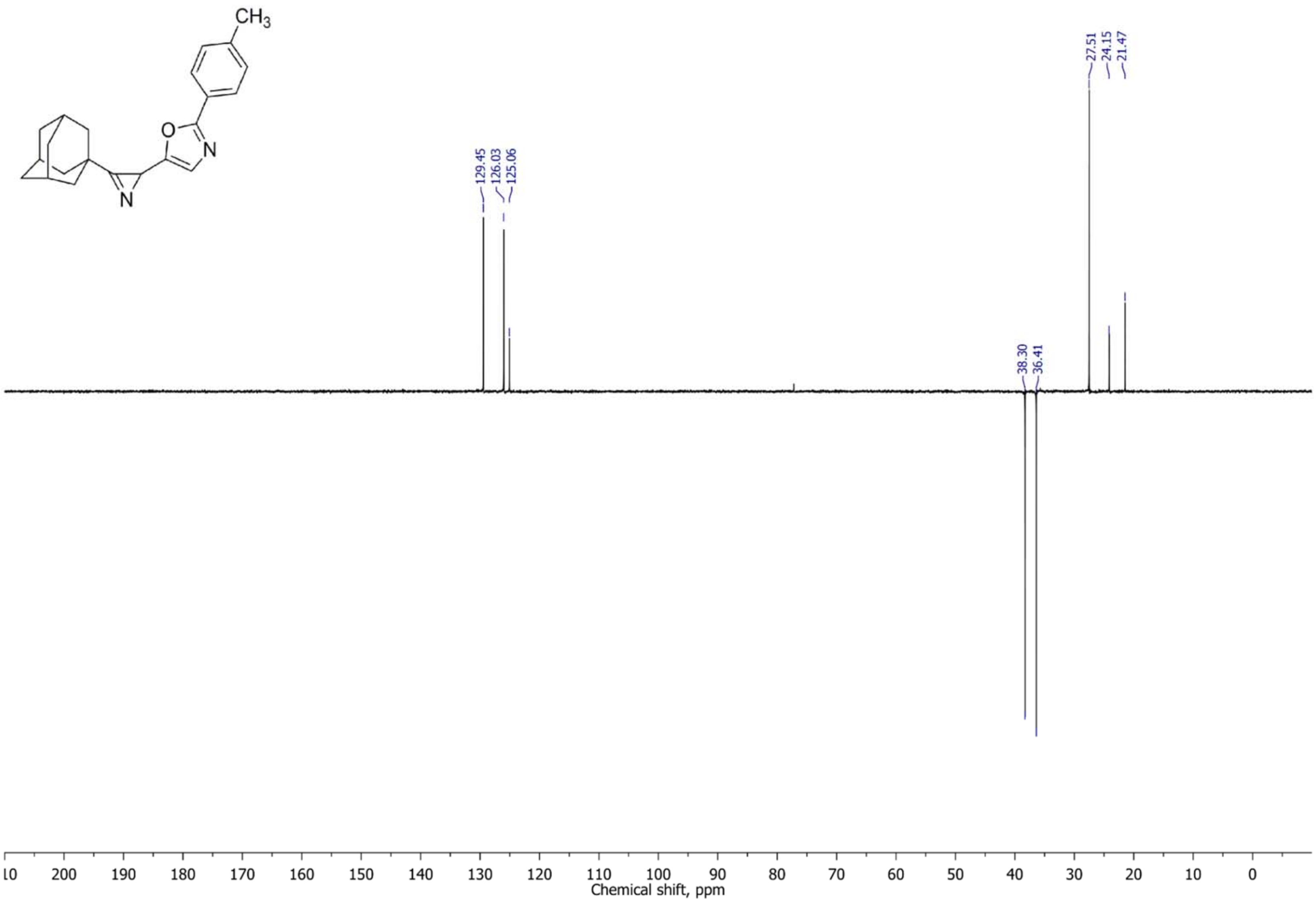

2-(4-Bromophenyl)-5-(3-phenyl-2*H*-azirin-2-yl)oxazole 2o, ^{1}H NMR, 400 MHz, $CDCl_3$

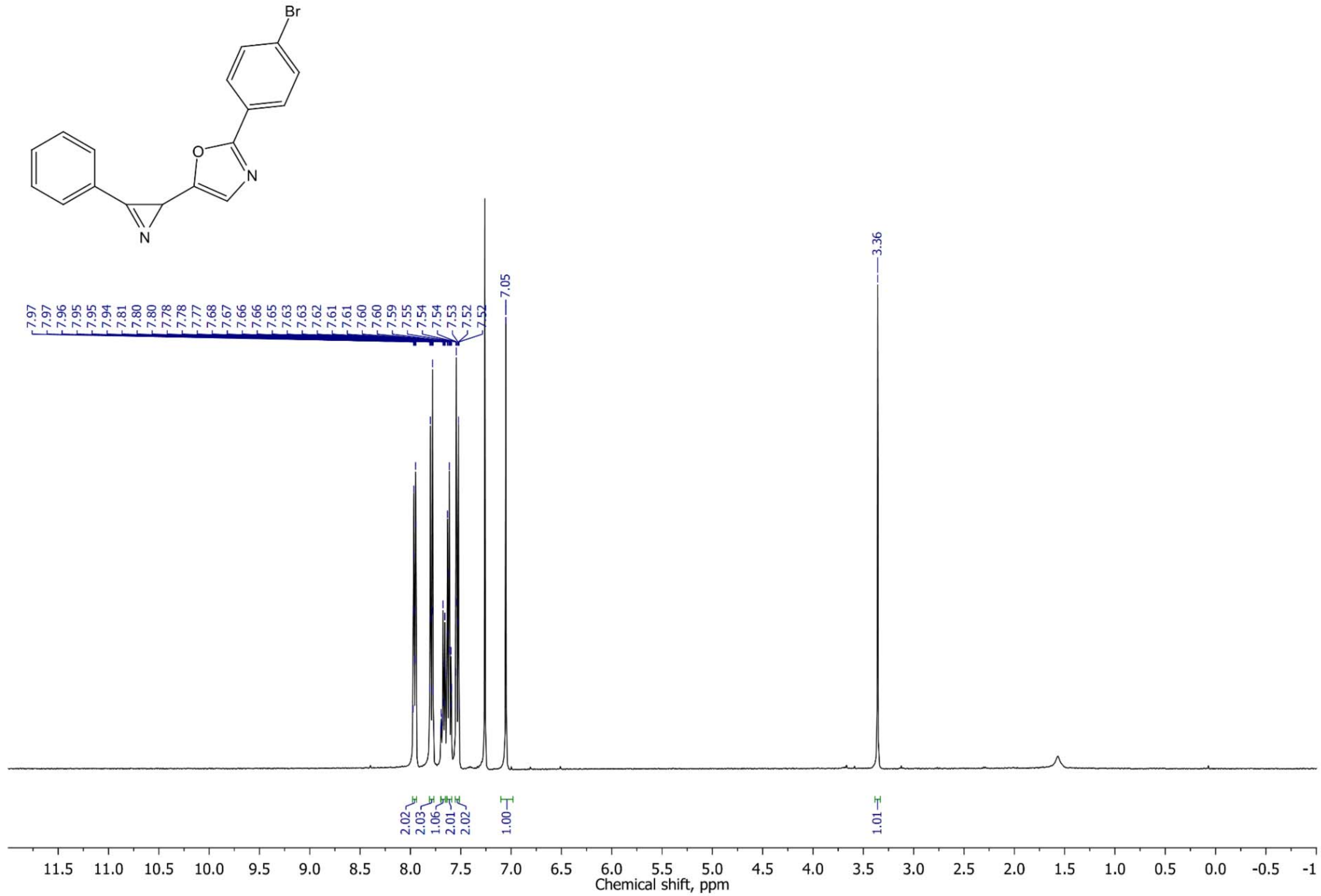

2-(4-Bromophenyl)-5-(3-phenyl-2*H*-azirin-2-yl)oxazole 2o, $^{13}C\{^{1}H\}$ NMR, 100 MHz, $CDCl_3$

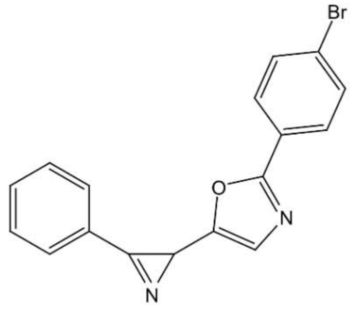

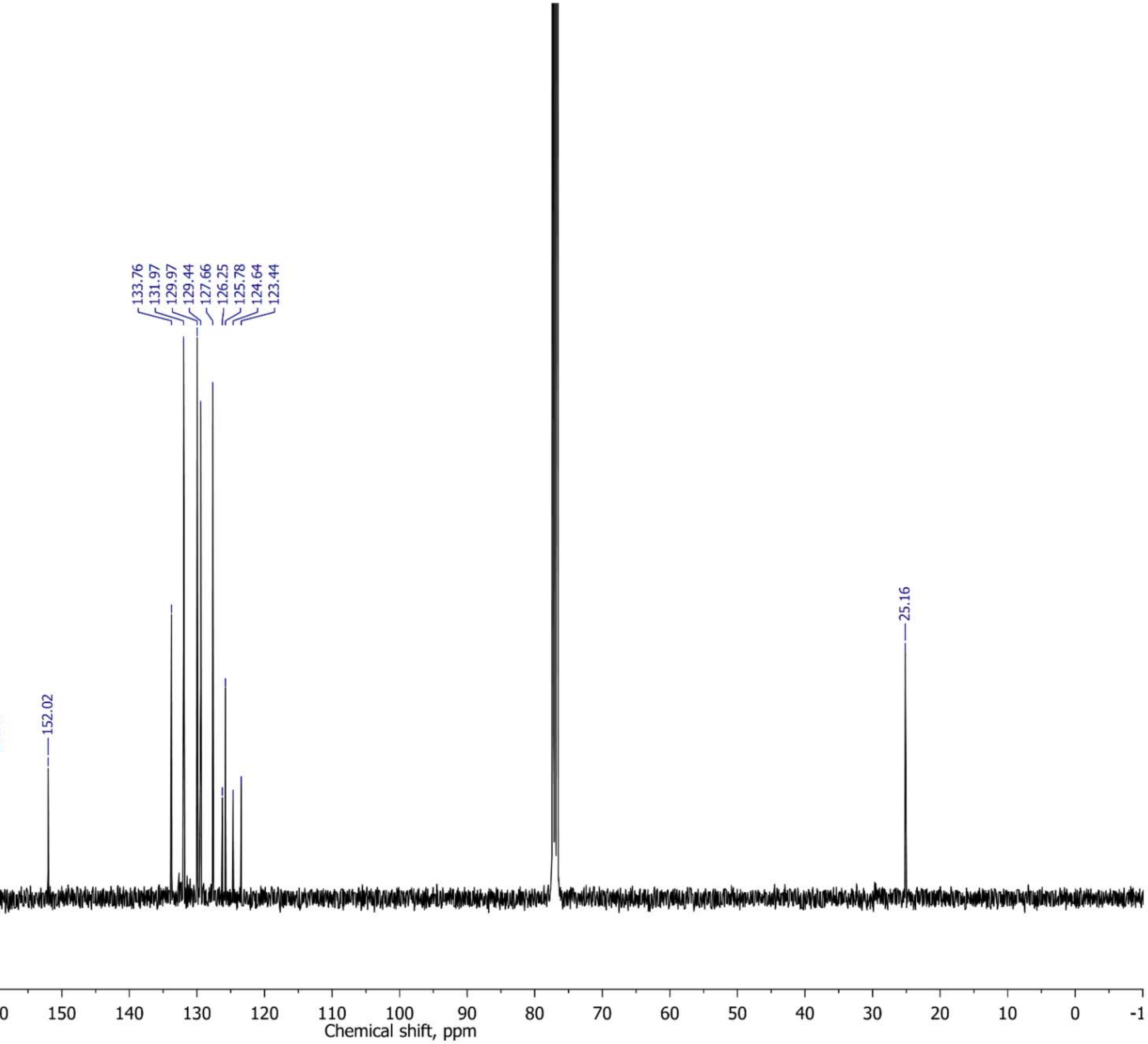

2-(4-Bromophenyl)-5-(3-phenyl-2*H*-azirin-2-yl)oxazole 2o, DEPT, 100 MHz, $CDCl_3$

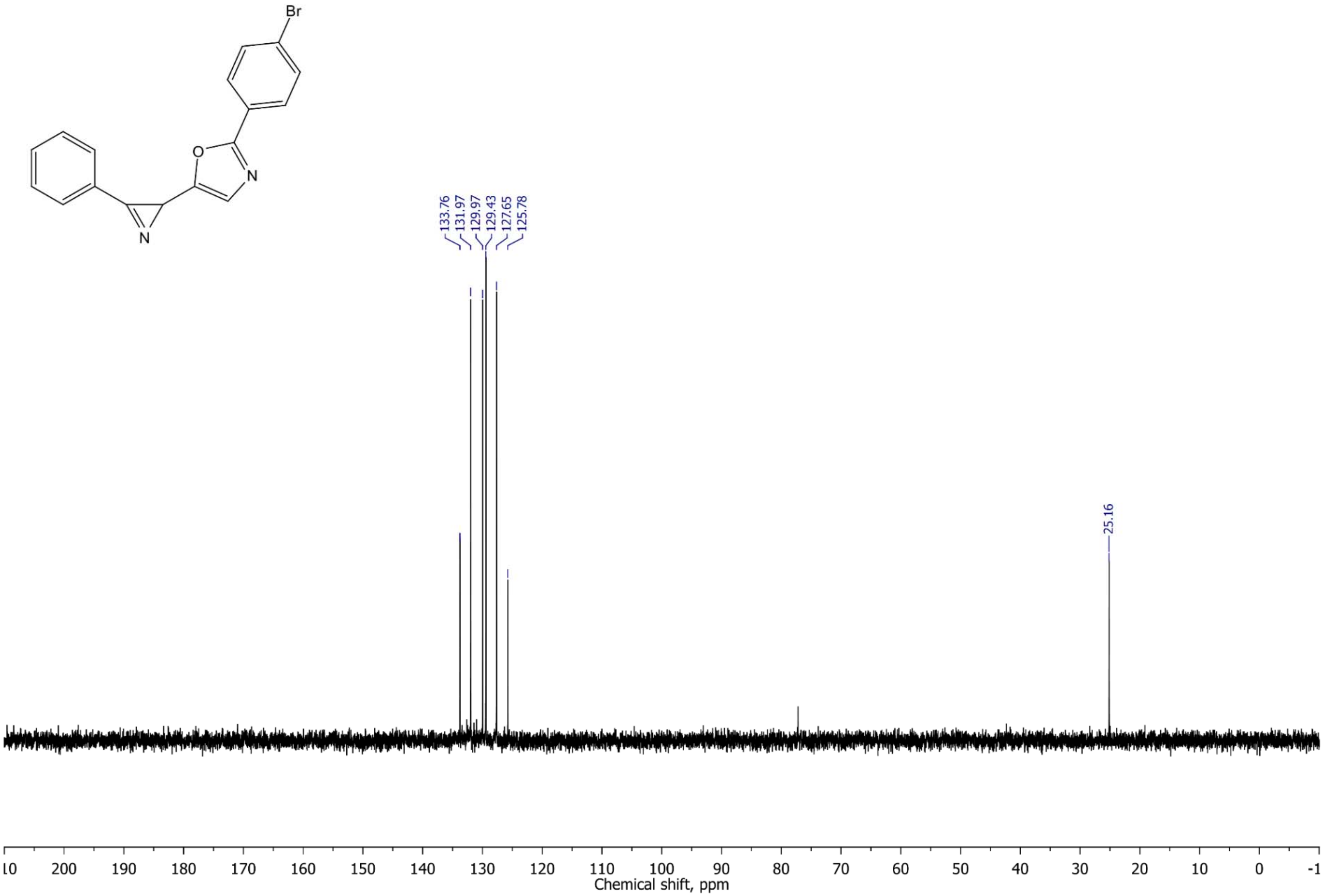

2-(4-Bromophenyl)-5-(3-(*tert*-butyl)-2*H*-azirin-2-yl)oxazole 2p, ^{1}H NMR, 400 MHz, $CDCl_3$

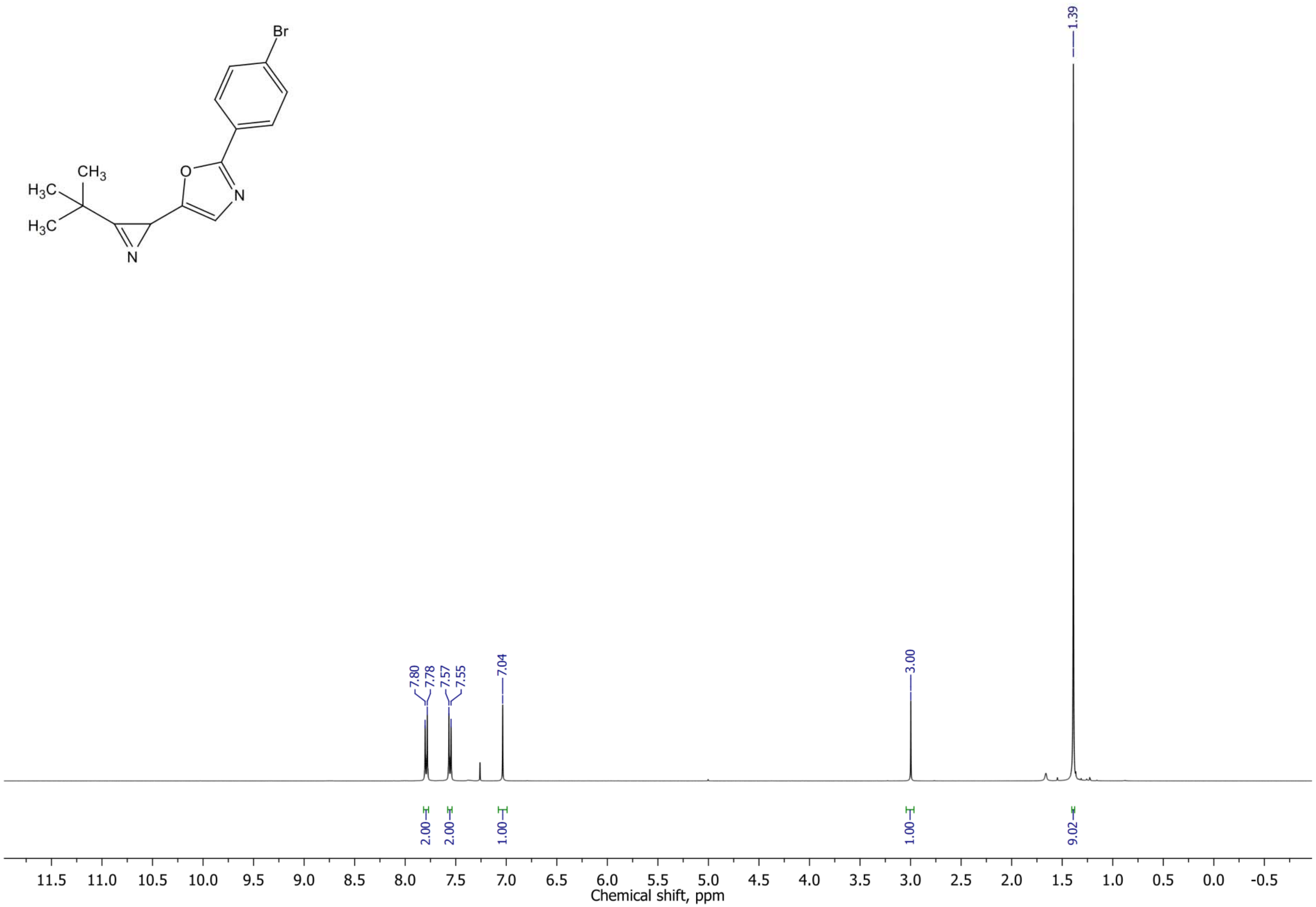

2-(4-Bromophenyl)-5-(3-(*tert*-butyl)-2*H*-azirin-2-yl)oxazole 2p, $^{13}C\{^{1}H\}$ NMR, 100 MHz, $CDCl_3$

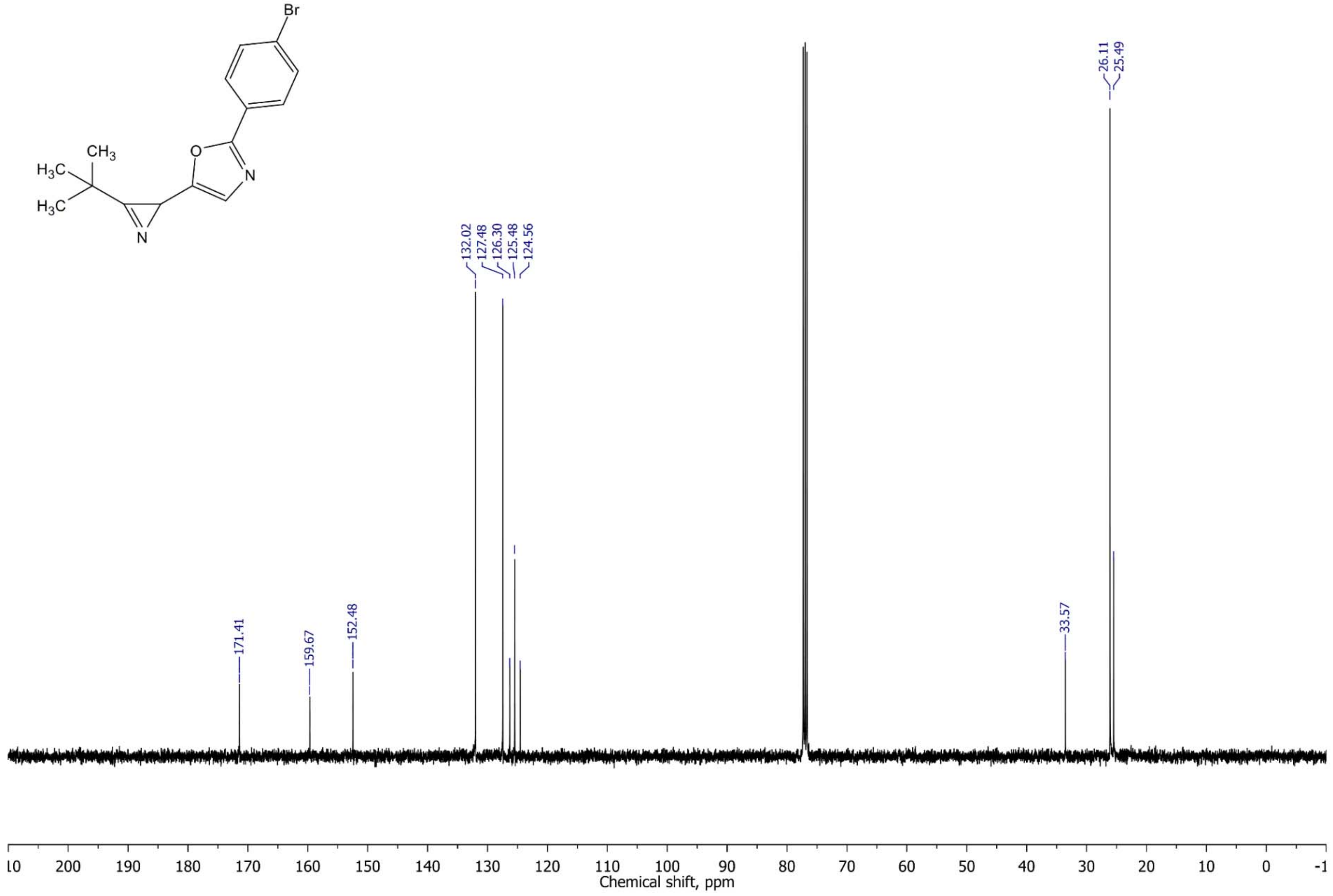

2-(4-Bromophenyl)-5-(3-(*tert*-butyl)-2*H*-azirin-2-yl)oxazole 2p, DEPT, 100 MHz, $CDCl_3$

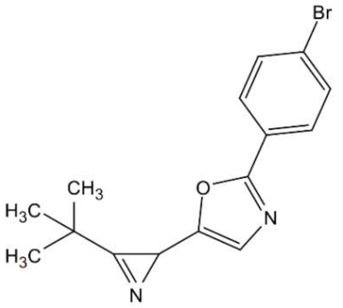

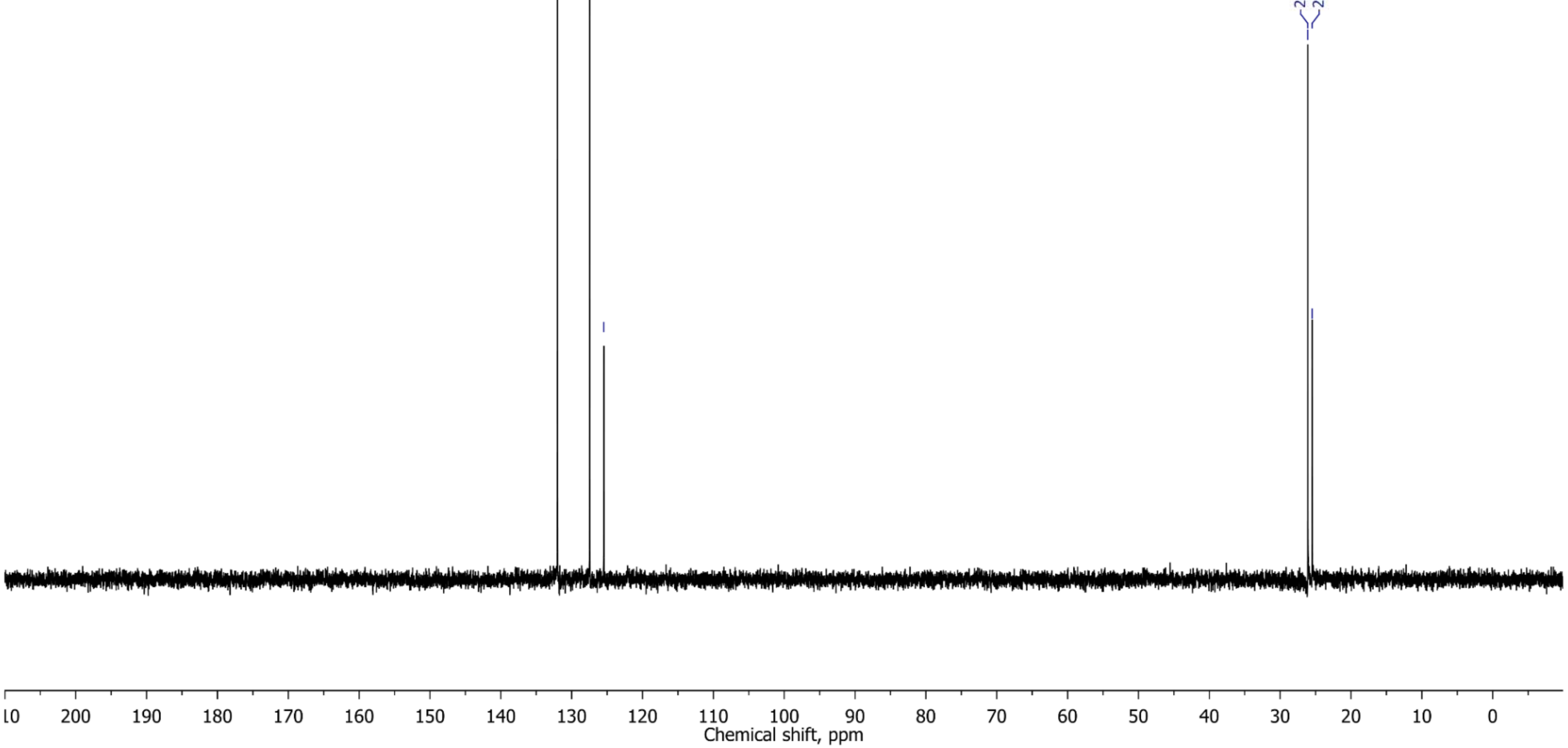

***(E)*-3-(5-(3-phenyl-2*H*-azirin-2-yl)oxazol-2-yl)acrylonitrile 2q, ^{1}H NMR, 400 MHz, CDCl$_3$**

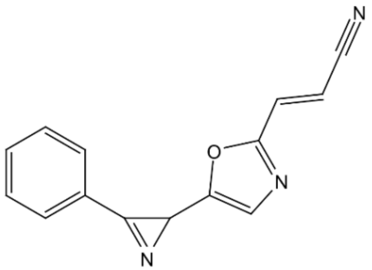

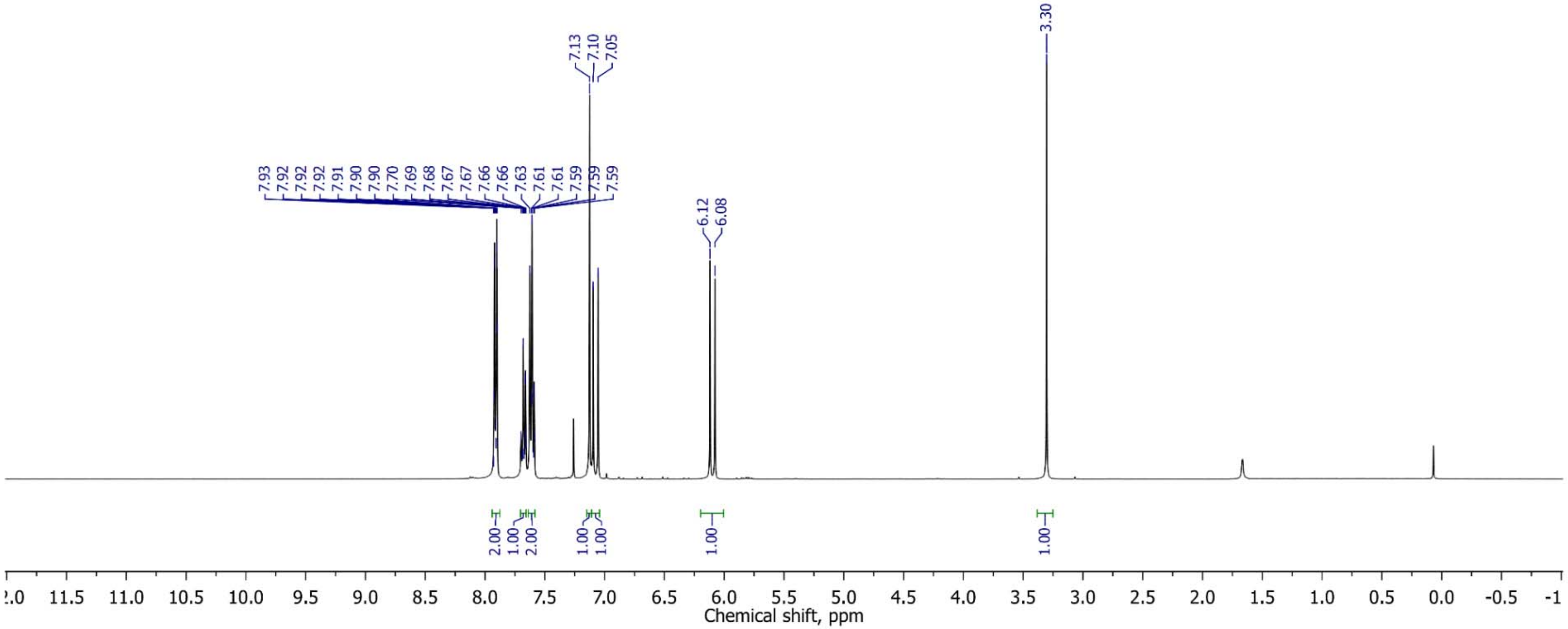

***(E)*-3-(5-(3-phenyl-2*H*-azirin-2-yl)oxazol-2-yl)acrylonitrile 2q, $^{13}C\{^1H\}$ NMR, 100 MHz, $CDCl_3$**

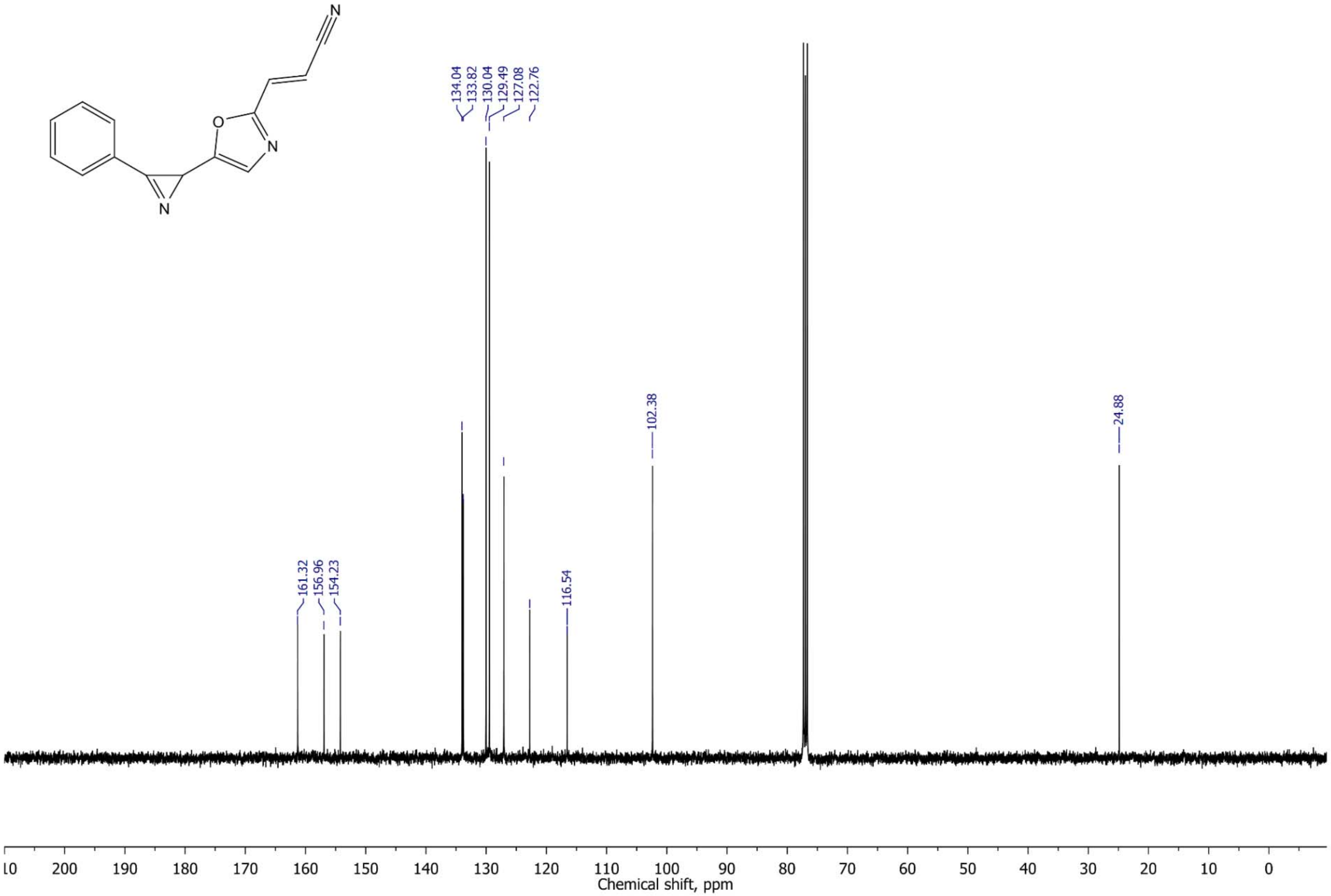

***(E)*-3-(5-(3-phenyl-2*H*-azirin-2-yl)oxazol-2-yl)acrylonitrile 2q, DEPT, 100 MHz, $CDCl_3$**

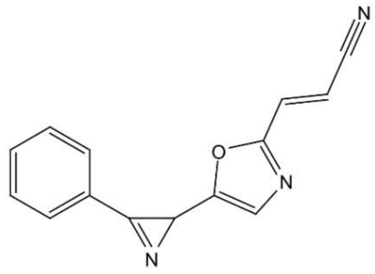

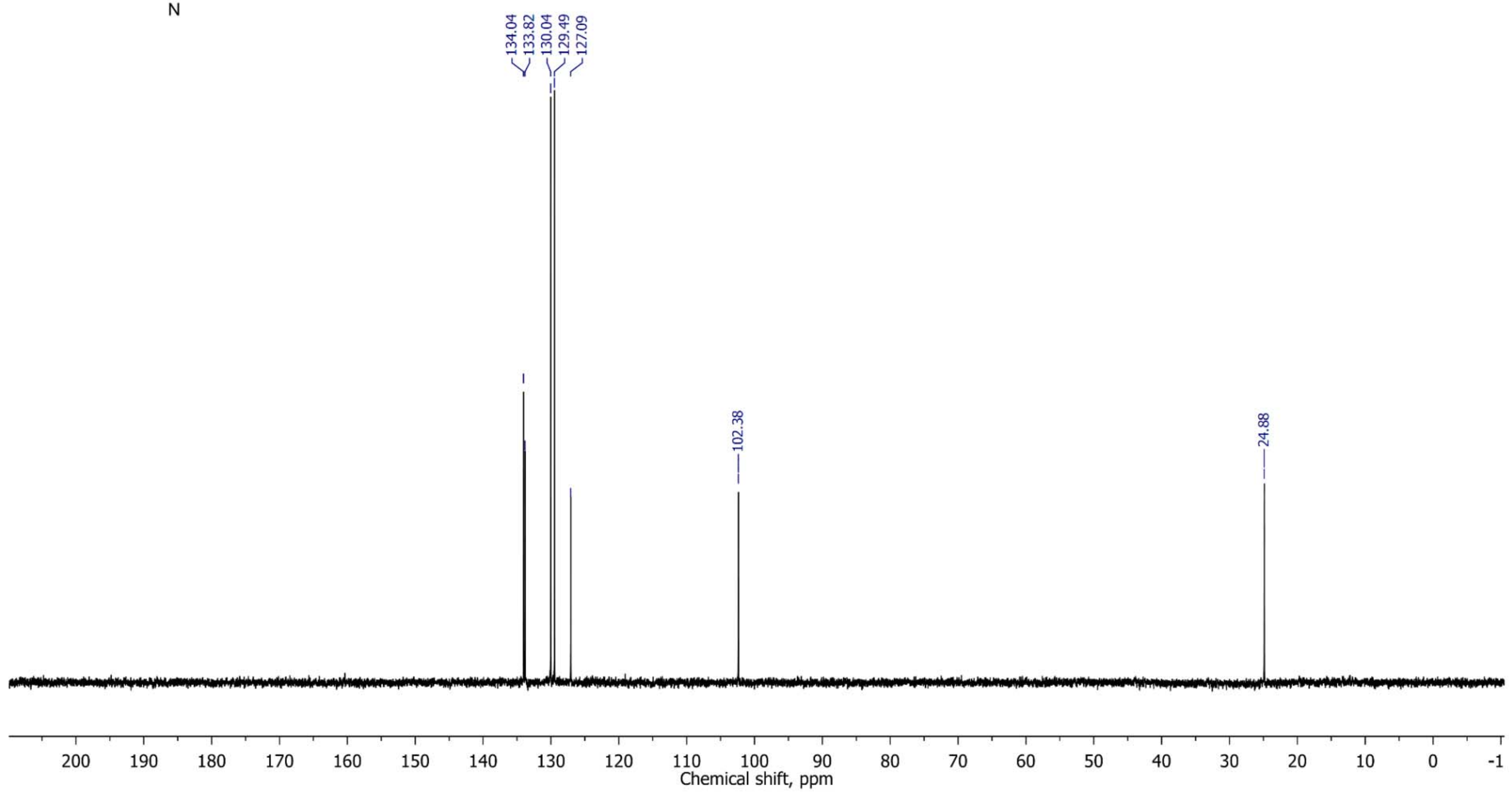

5-(3-Phenyl-2*H*-azirin-2-yl)-2-vinyloxazole 2r, ^{1}H NMR, 400 MHz, $CDCl_3$

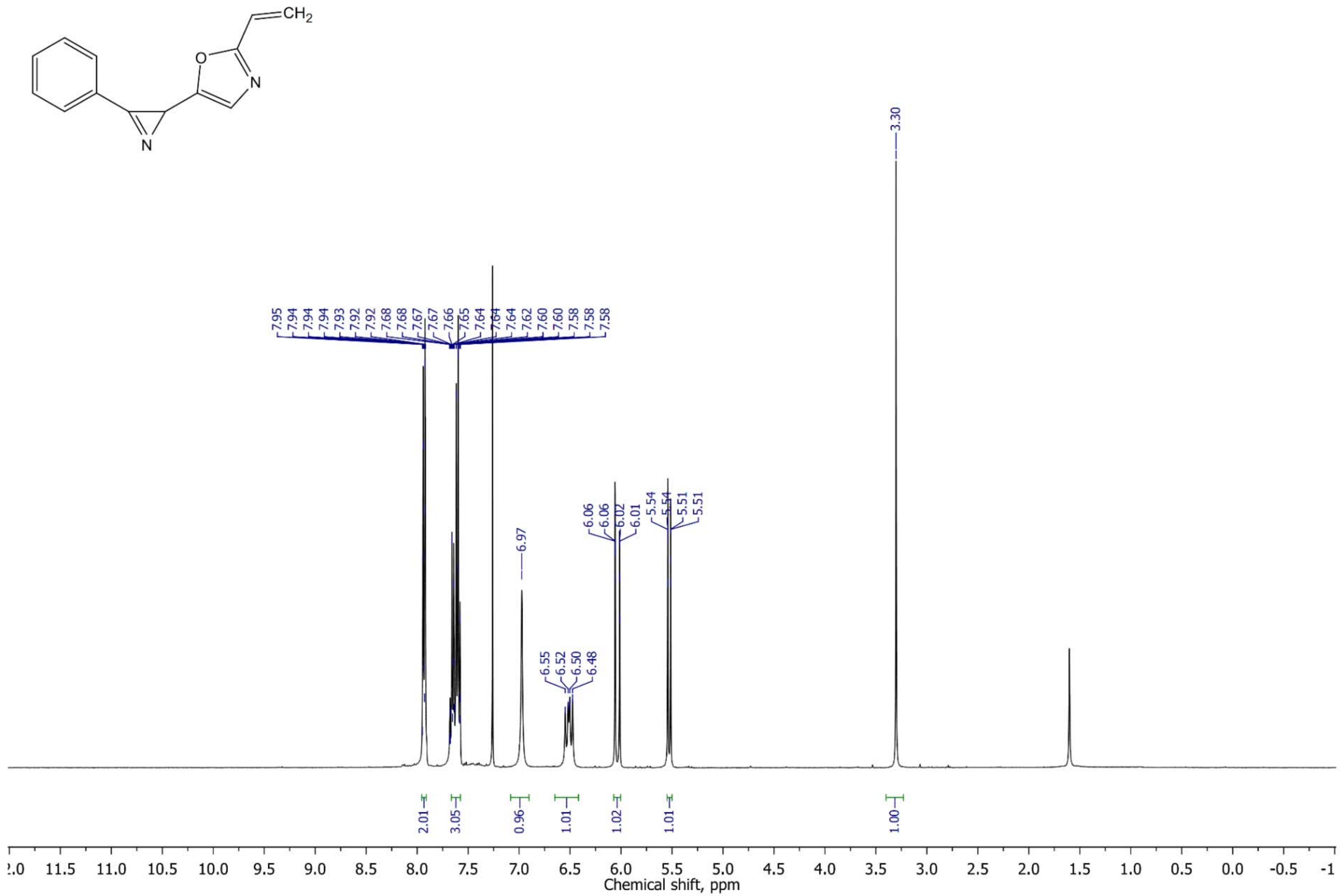

5-(3-Phenyl-2*H*-azirin-2-yl)-2-vinyloxazole 2r, $^{13}C\{^{1}H\}$ NMR, 100 MHz, $CDCl_3$

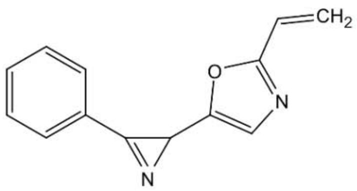

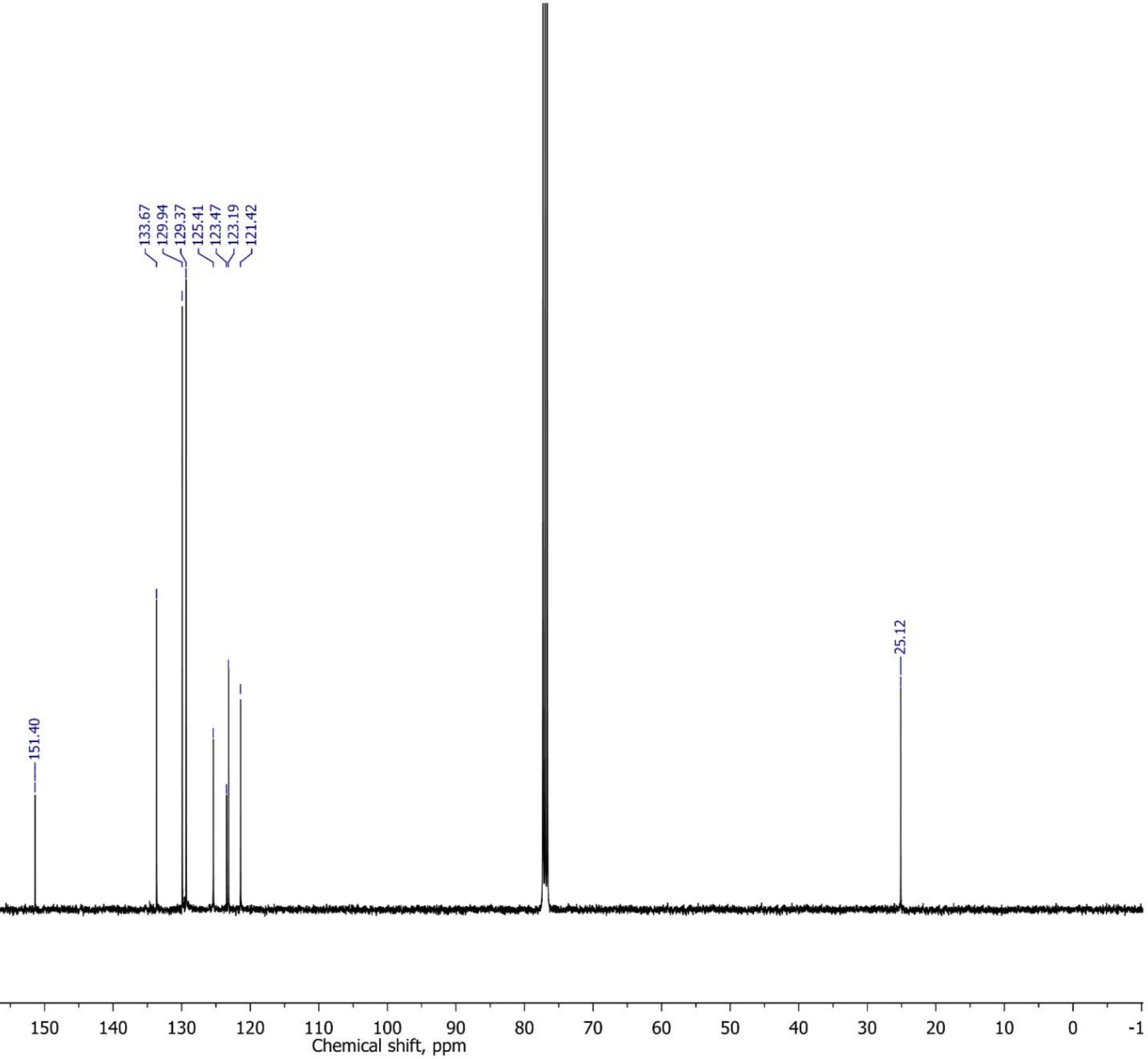

5-(3-Phenyl-2*H*-azirin-2-yl)-2-vinyloxazole 2r, DEPT, 100 MHz, $CDCl_3$

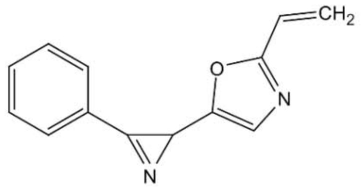

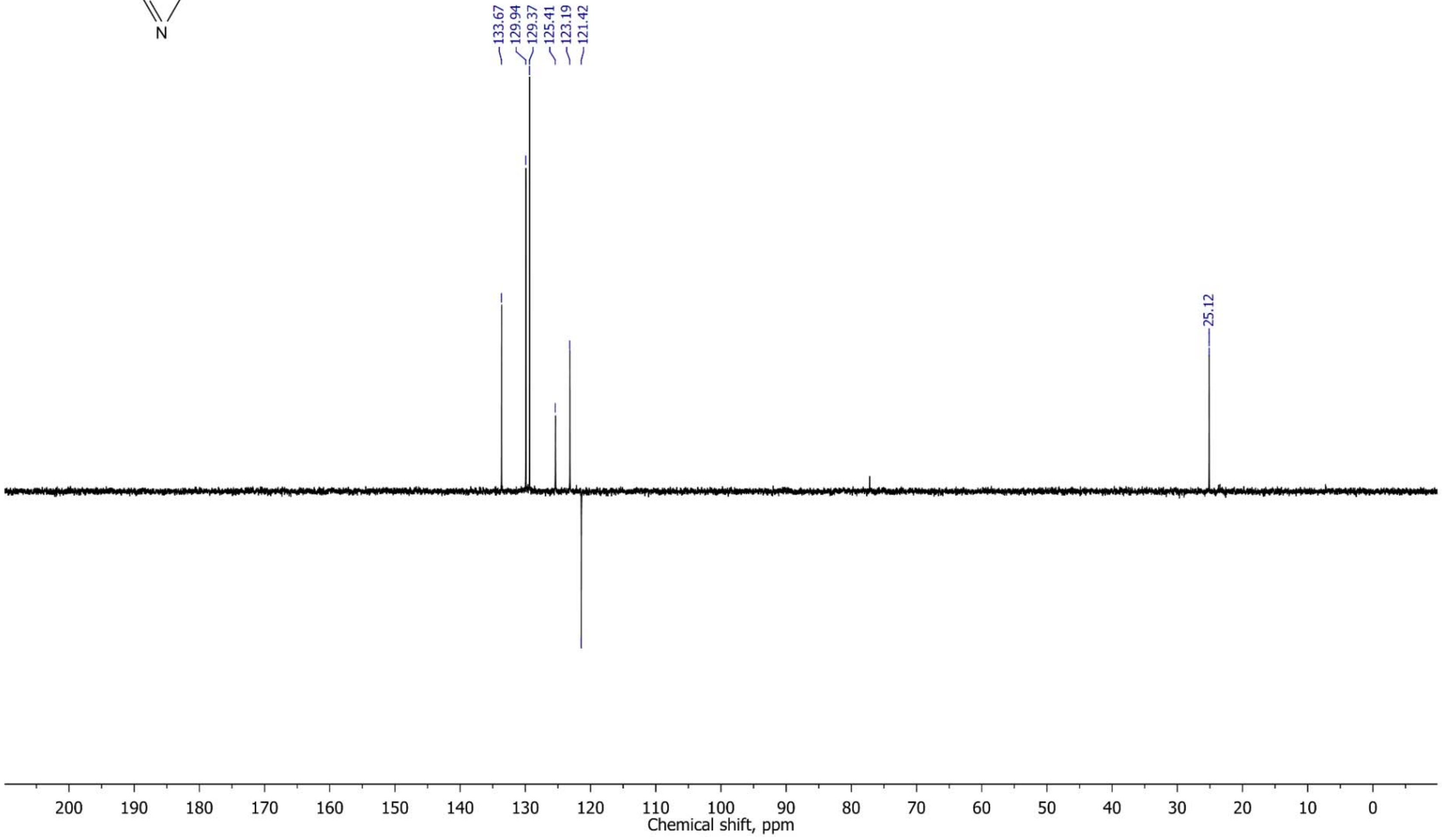

2-(Chloromethyl)-5-(3-phenyl-2*H*-azirin-2-yl)oxazole 2s, ^{1}H NMR, 400 MHz, $CDCl_3$

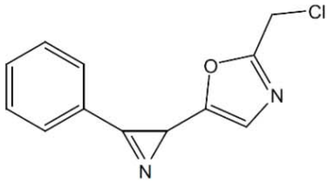

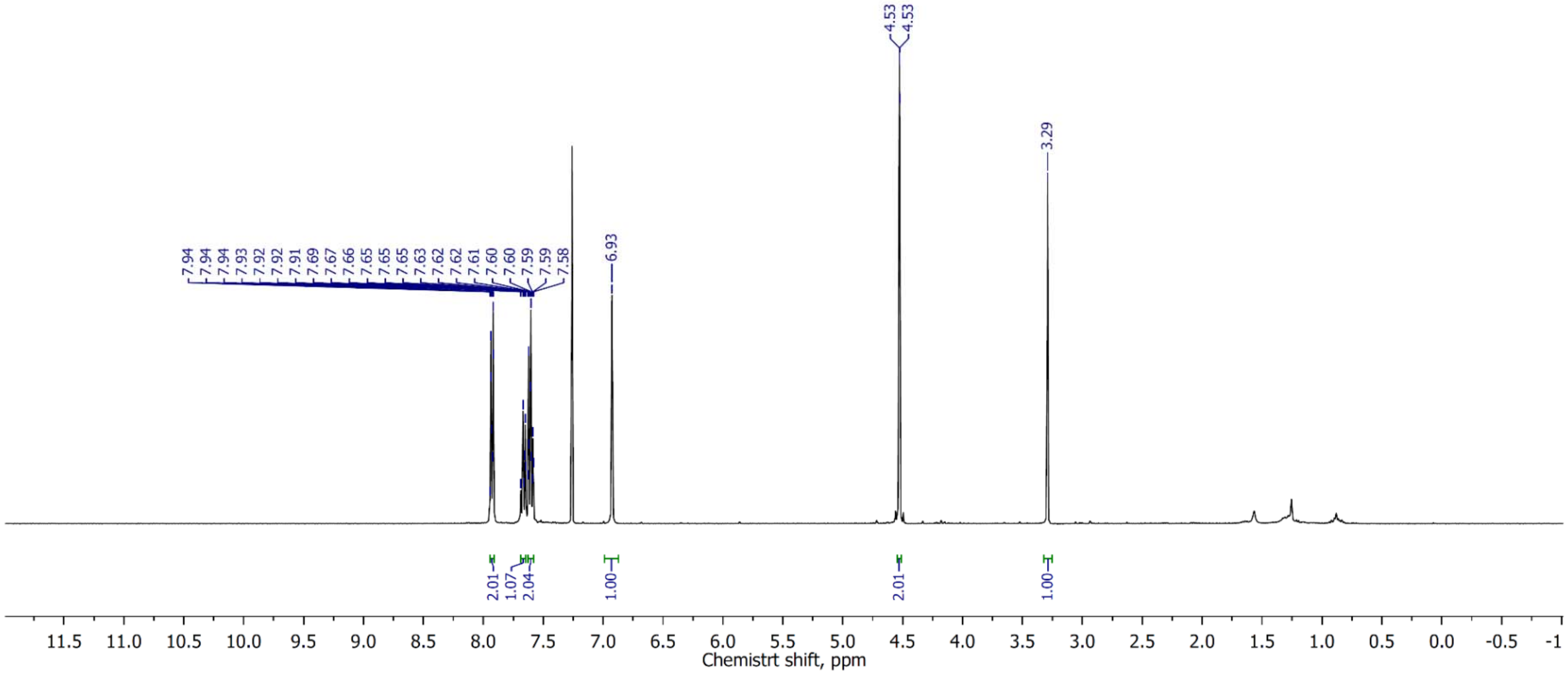

2-(Chloromethyl)-5-(3-phenyl-2*H*-azirin-2-yl)oxazole 2s, $^{13}C\{^{1}H\}$ NMR, 100 MHz, $CDCl_3$

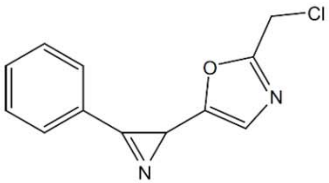

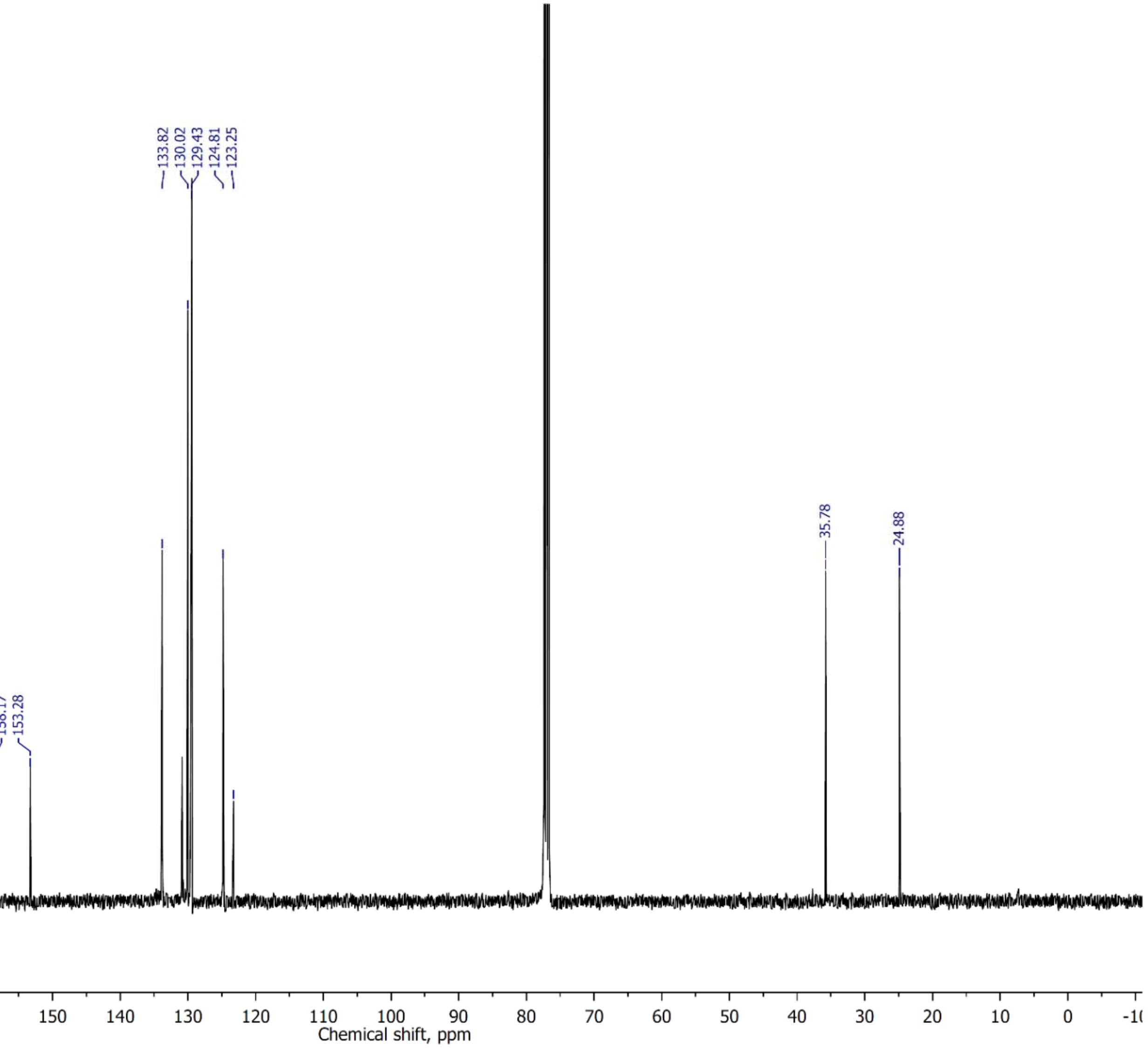

2-(Chloromethyl)-5-(3-phenyl-2*H*-azirin-2-yl)oxazole 2s, DEPT, 100 MHz, $CDCl_3$

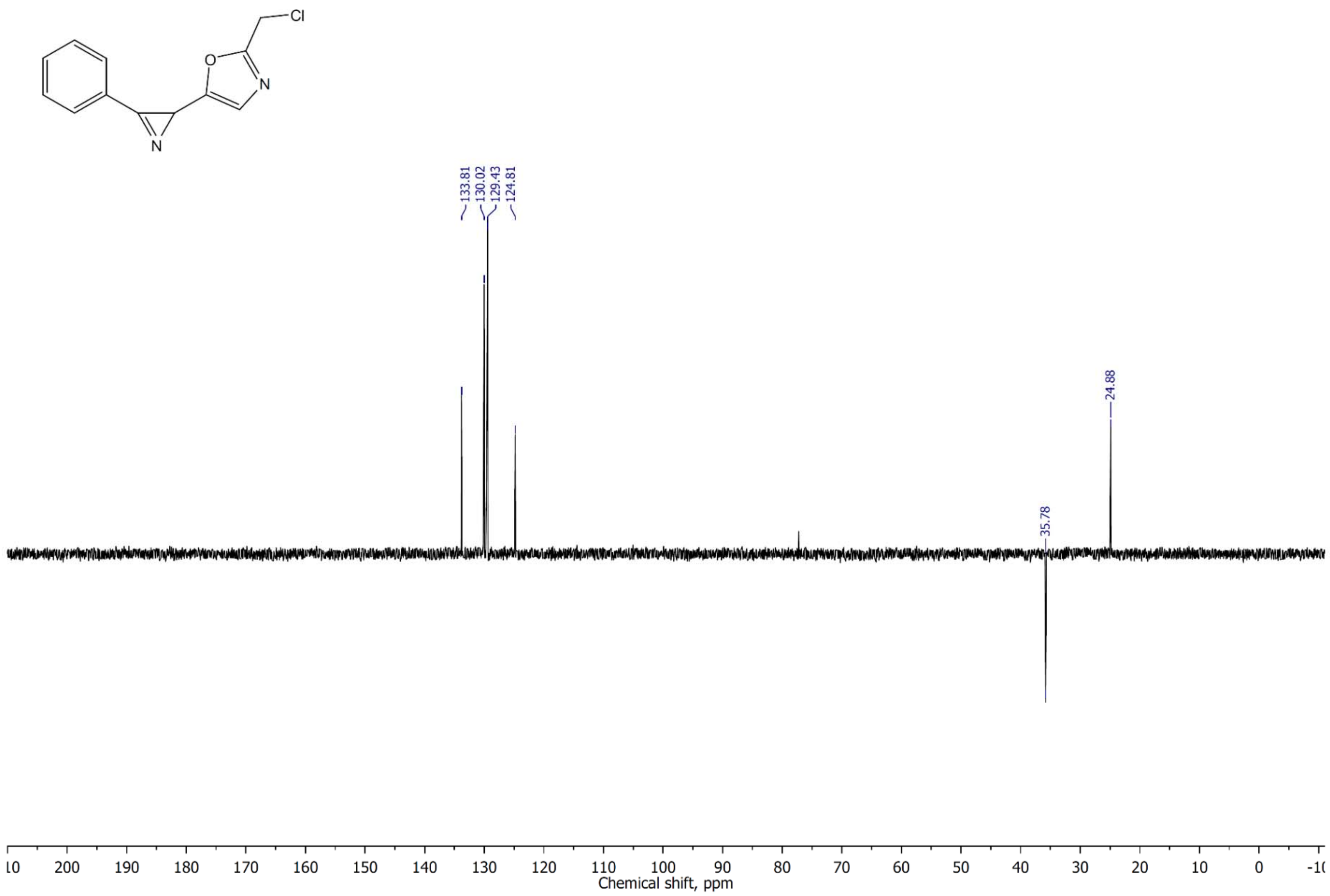

2-Methyl-5-phenyl-4*H*-pyrrolo[2,3-*d*]oxazole 3a, ^{1}H NMR, 400 MHz, DMSO-d_6

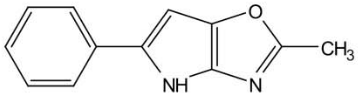

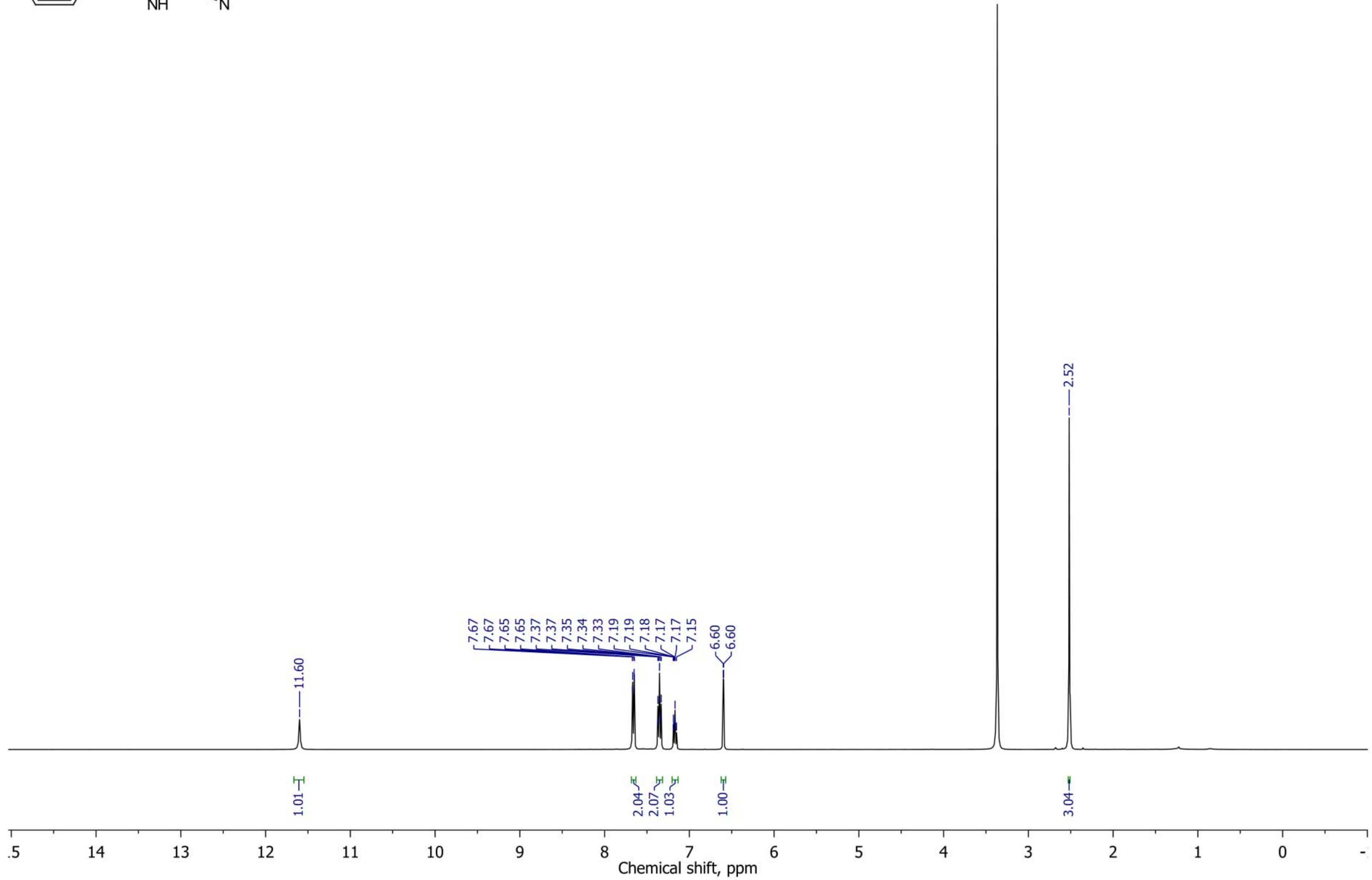

2-Methyl-5-phenyl-4*H*-pyrrolo[2,3-*d*]oxazole 3a, $^{13}C\{^{1}H\}$ NMR, 100 MHz, DMSO-d$_6$

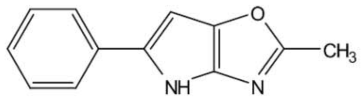

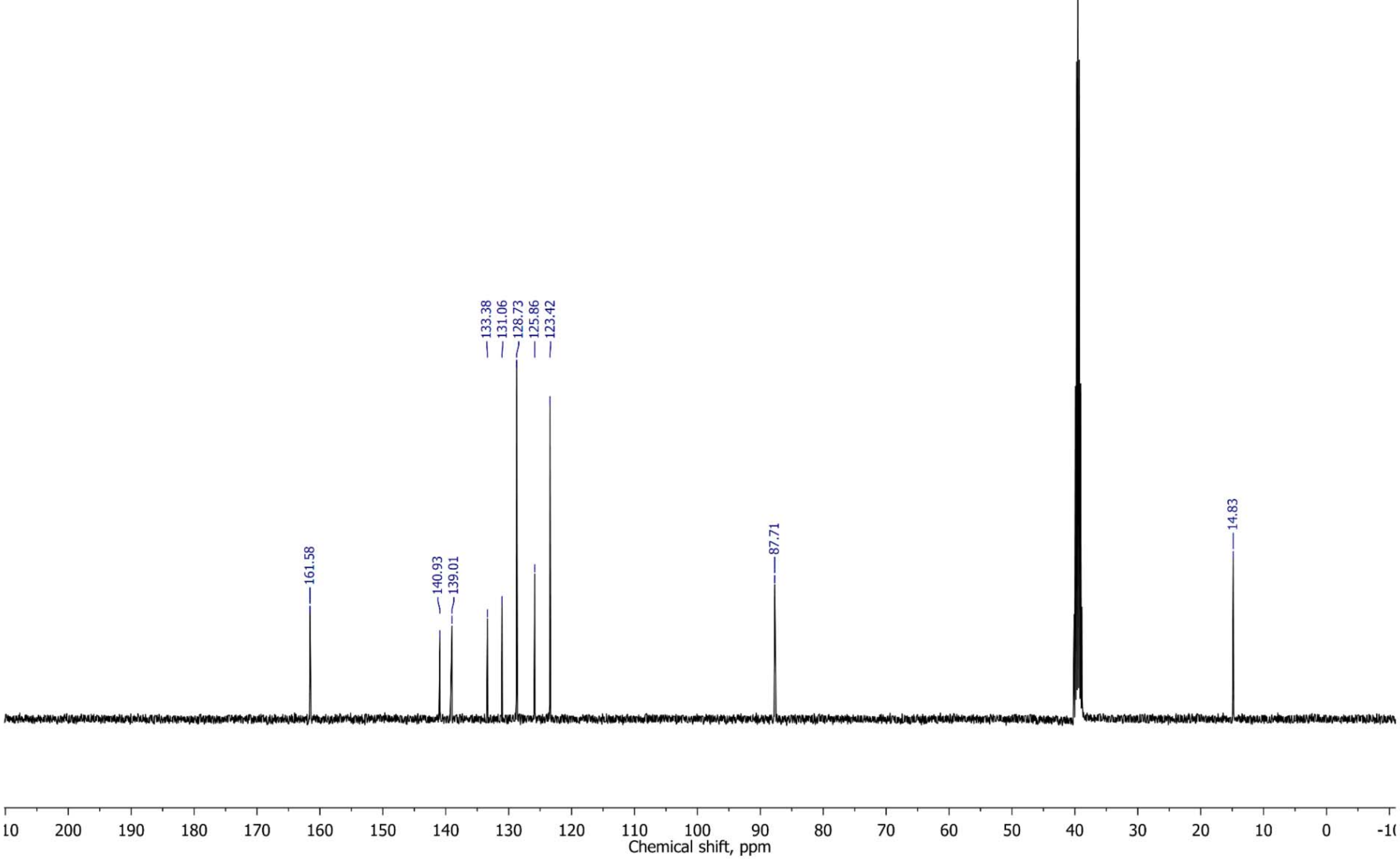

2-Methyl-5-phenyl-4*H*-pyrrolo[2,3-*d*]oxazole 3a, $^{13}C\{^{1}H\}$ NMR, 100 MHz, DMSO-d_6

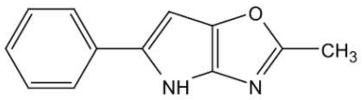

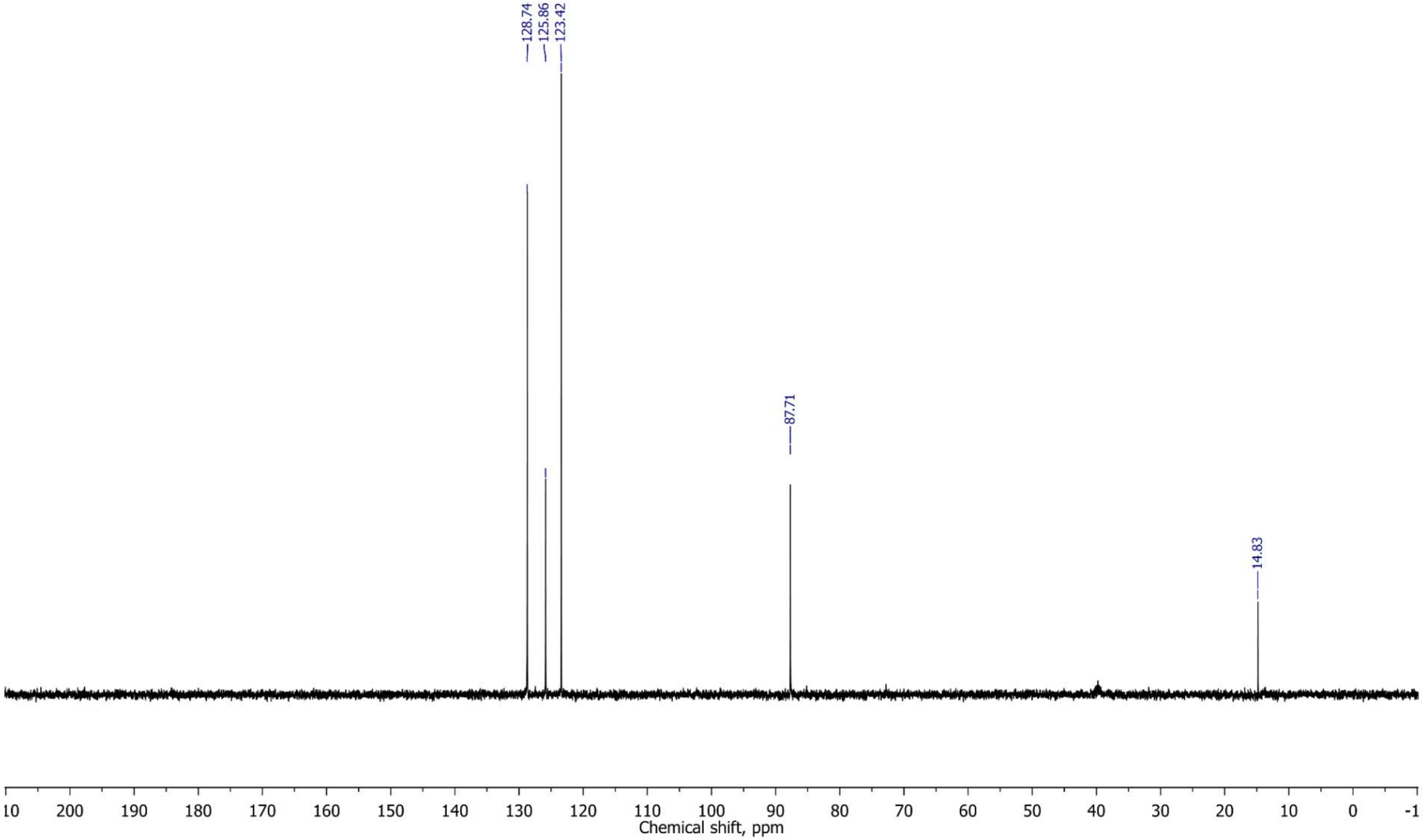

2-Methyl-5-(4-bromophenyl)-4*H*-pyrrolo[2,3-*d*]oxazole 3b, ^{1}H NMR, 400 MHz, DMSO-d$_6$

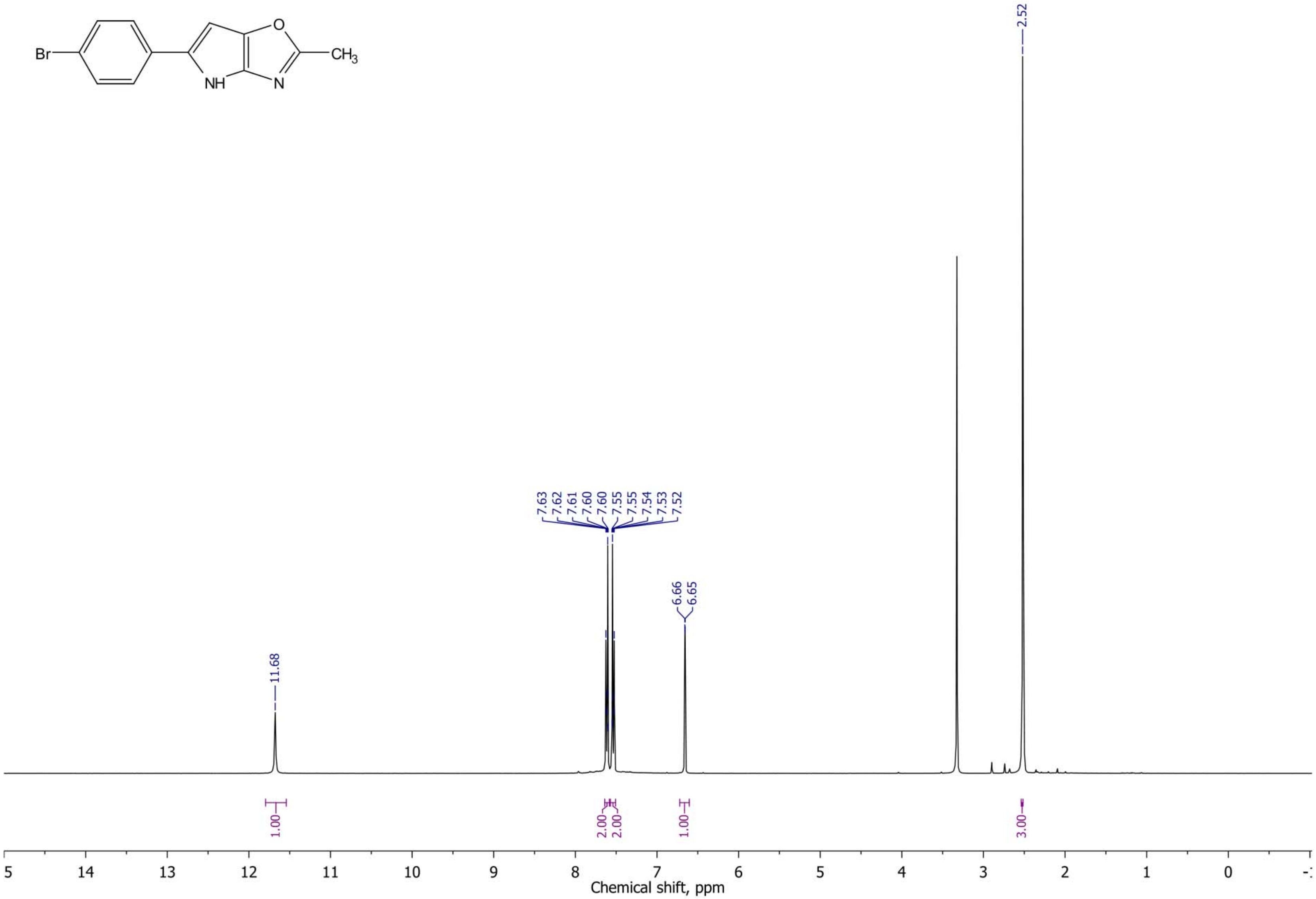

2-Methyl-5-(4-bromophenyl)-4*H*-pyrrolo[2,3-*d*]oxazole 3b, $^{13}C\{^1H\}$ NMR, 100 MHz, DMSO-d$_6$

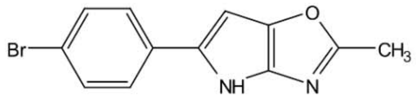

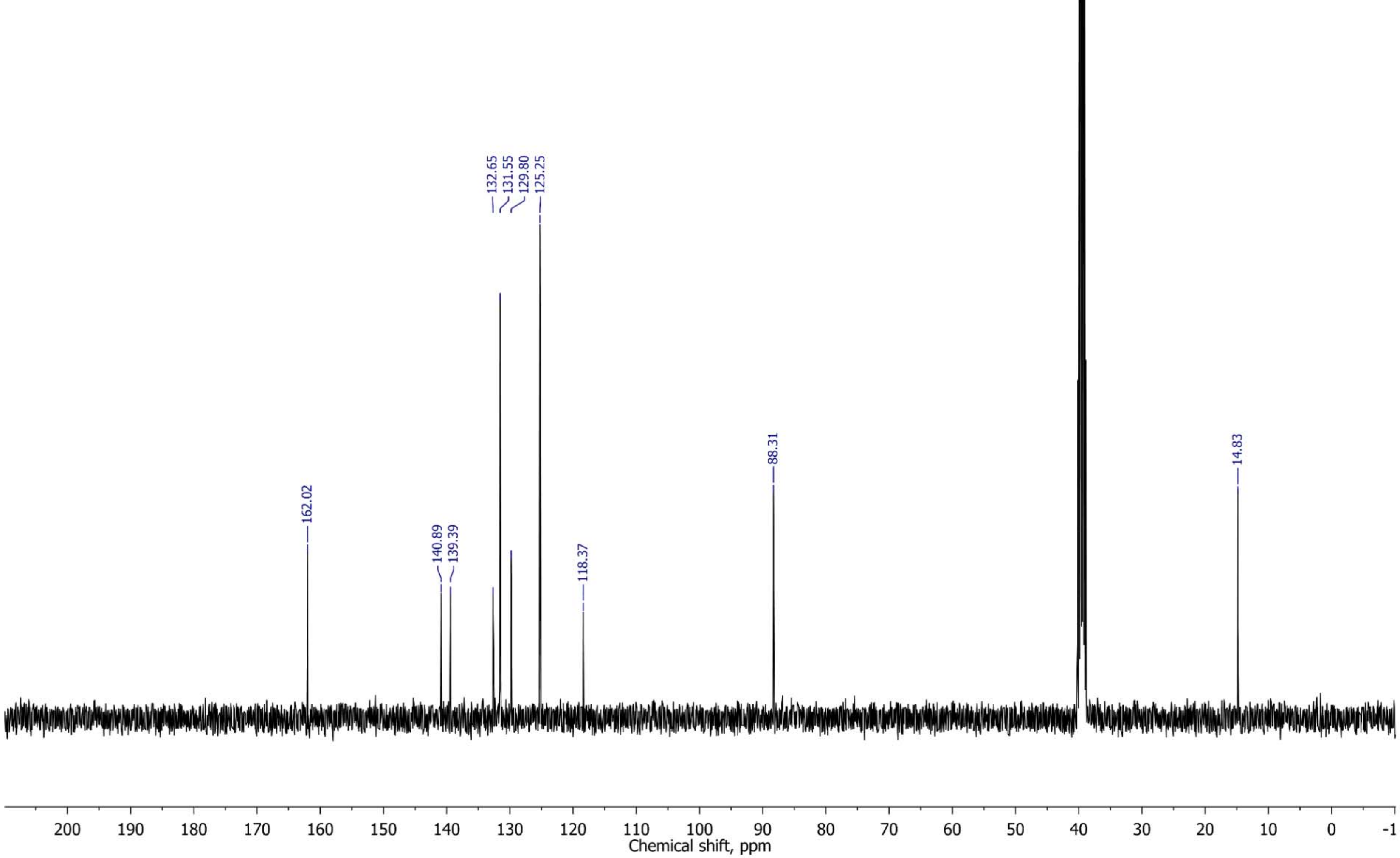

2-Methyl-5-(4-bromophenyl)-4*H*-pyrrolo[2,3-*d*]oxazole 3b, DEPT, 100 MHz, DMSO-d$_6$

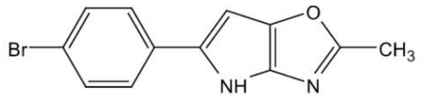

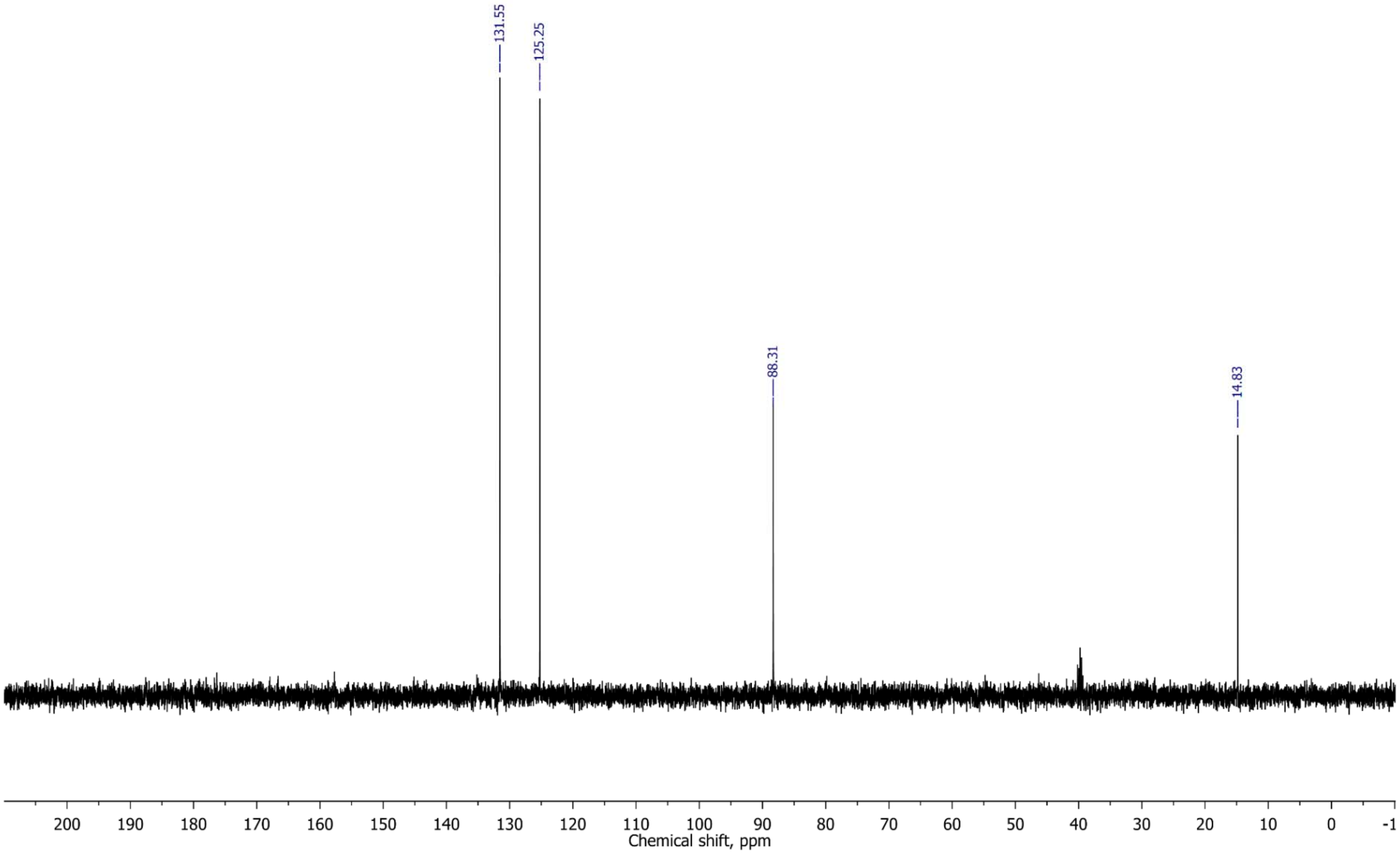

5-(Amantan-1-yl)-2-methyl-4*H*-pyrrolo[2,3-*d*]oxazole 3c, ^{1}H NMR, 400 MHz, $CDCl_3$

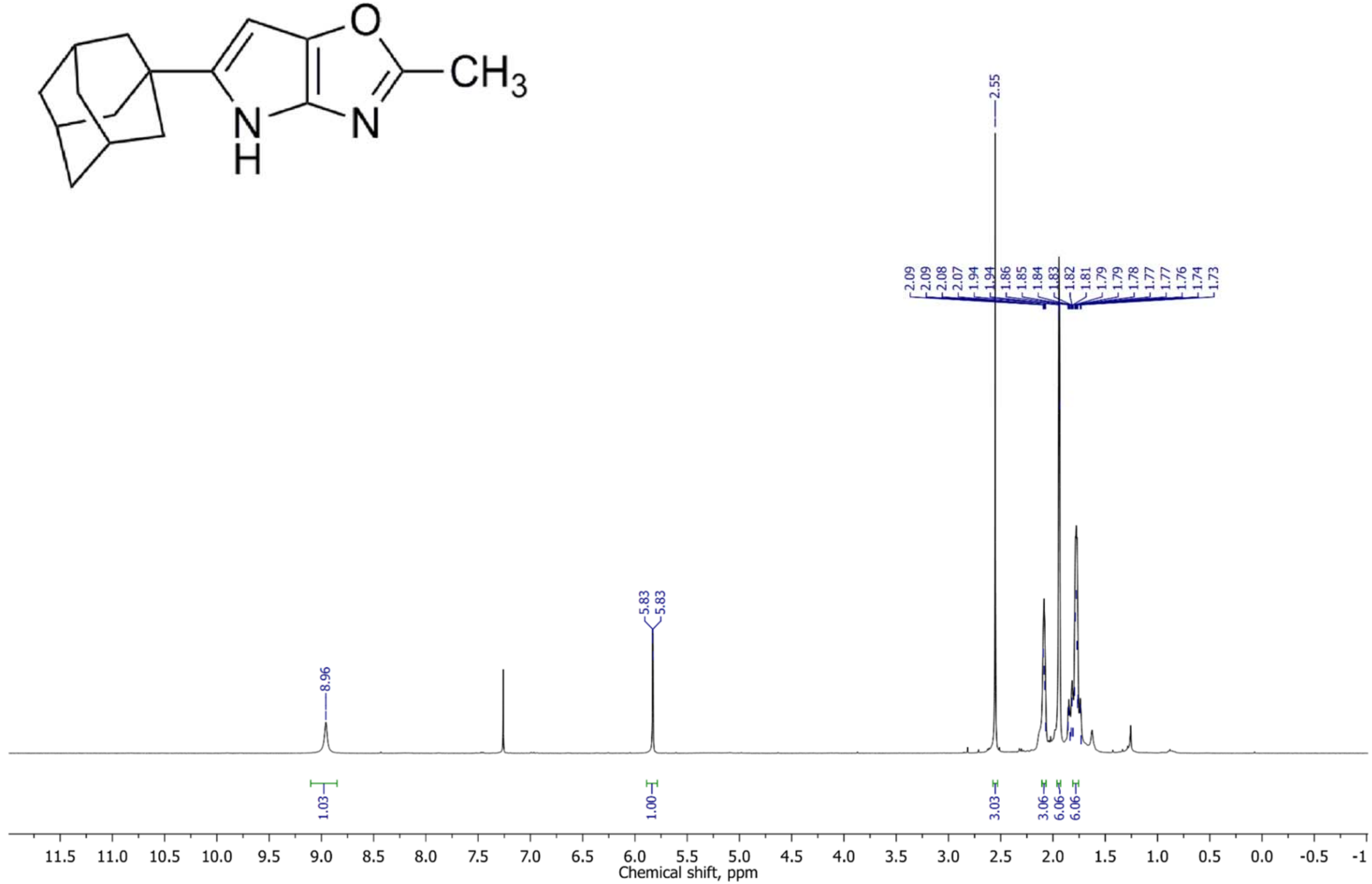

5-(Amantan-1-yl)-2-methyl-4*H*-pyrrolo[2,3-*d*]oxazole 3c, $^{13}C\{^{1}H\}$ NMR, 100 MHz, $CDCl_3$

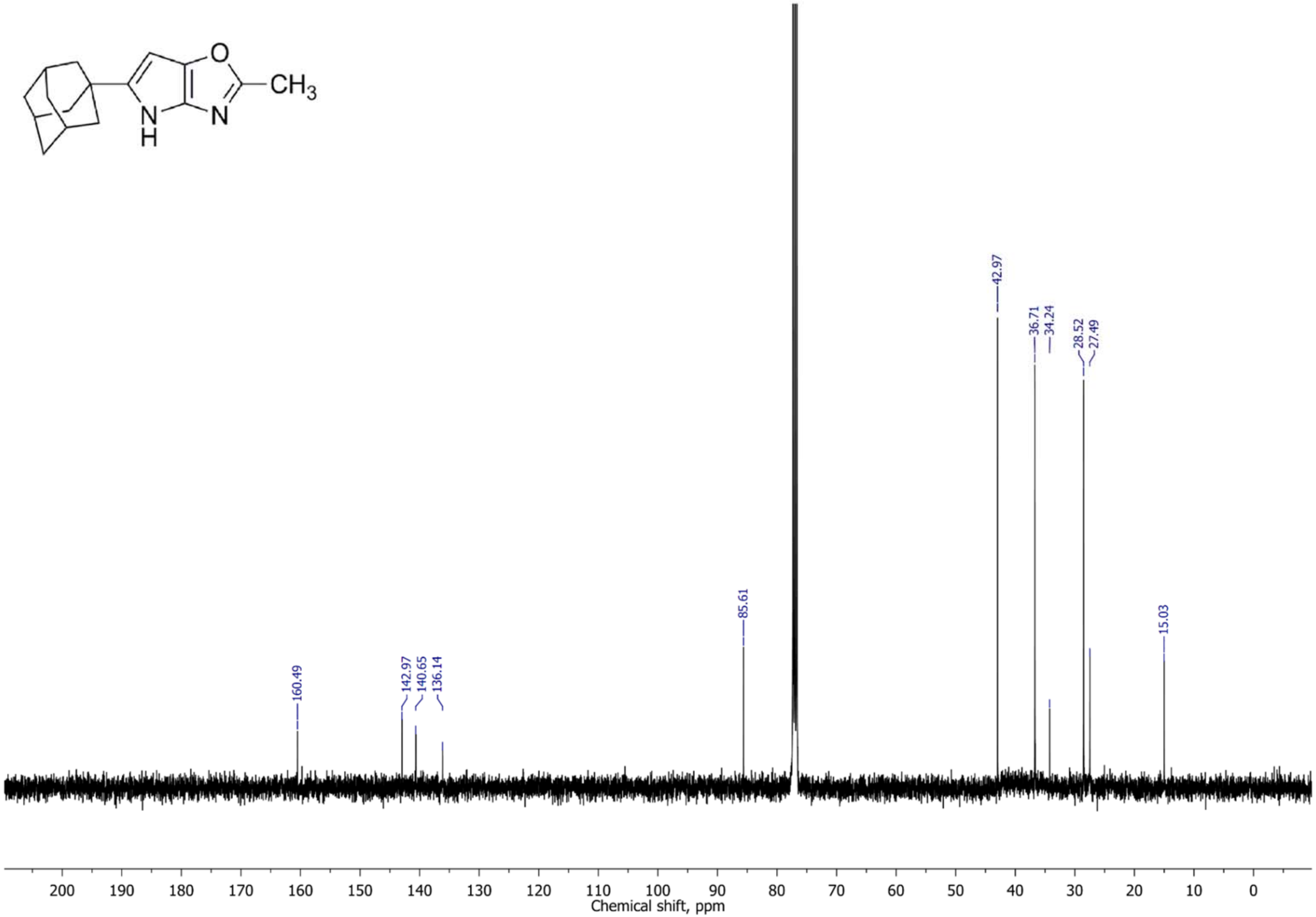

5-(Amantan-1-yl)-2-methyl-4*H*-pyrrolo[2,3-*d*]oxazole 3c, DEPT, 100 MHz, $CDCl_3$

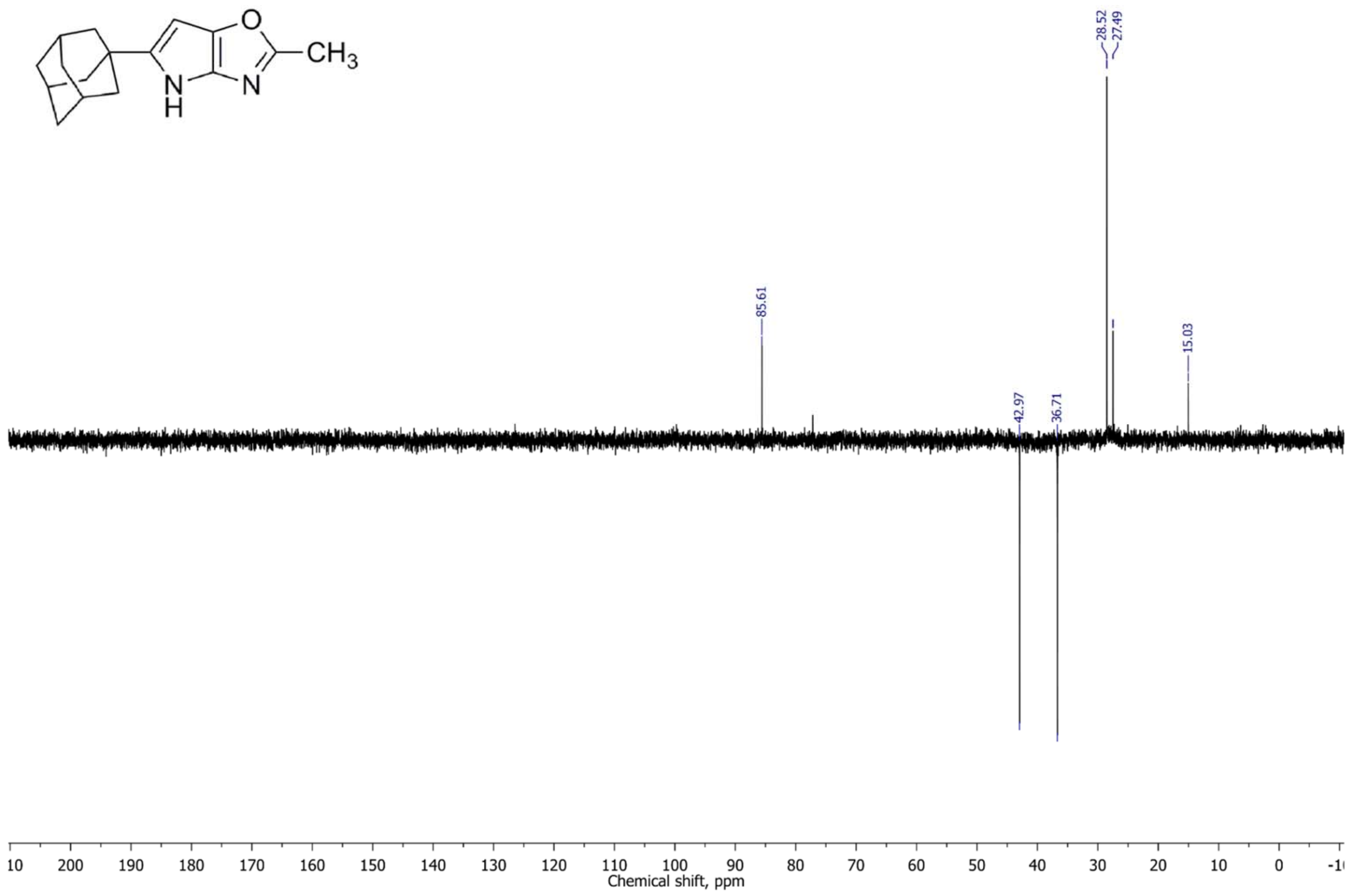

2-Ethyl-5-phenyl-4*H*-pyrrolo[2,3-*d*]oxazole 3d, ^{1}H NMR, 400 MHz, $CDCl_3$

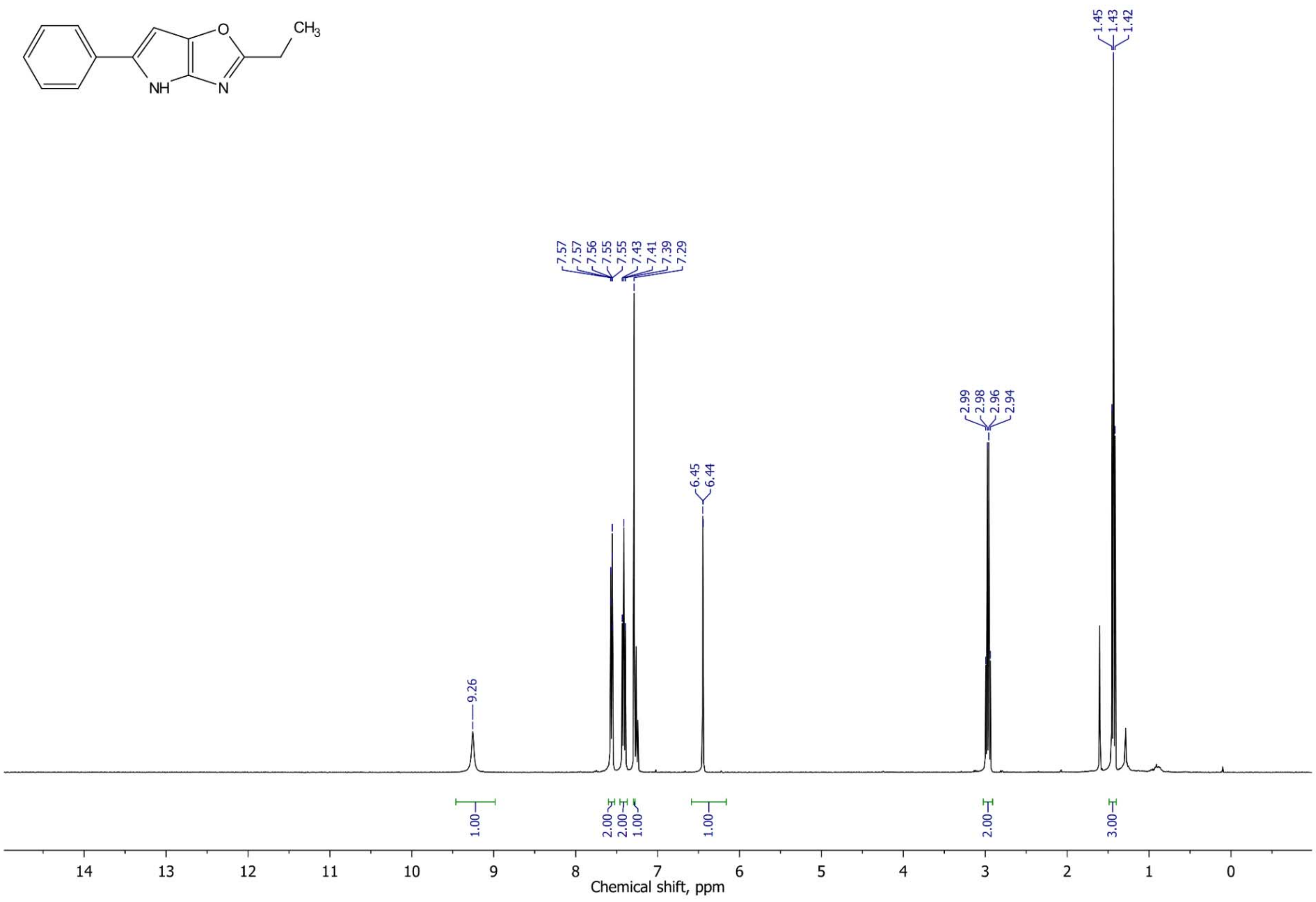

2-Ethyl-5-phenyl-4*H*-pyrrolo[2,3-*d*]oxazole 3d, $^{13}C\{^1H\}$ NMR, 100 MHz, $CDCl_3$

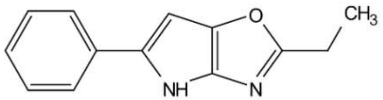

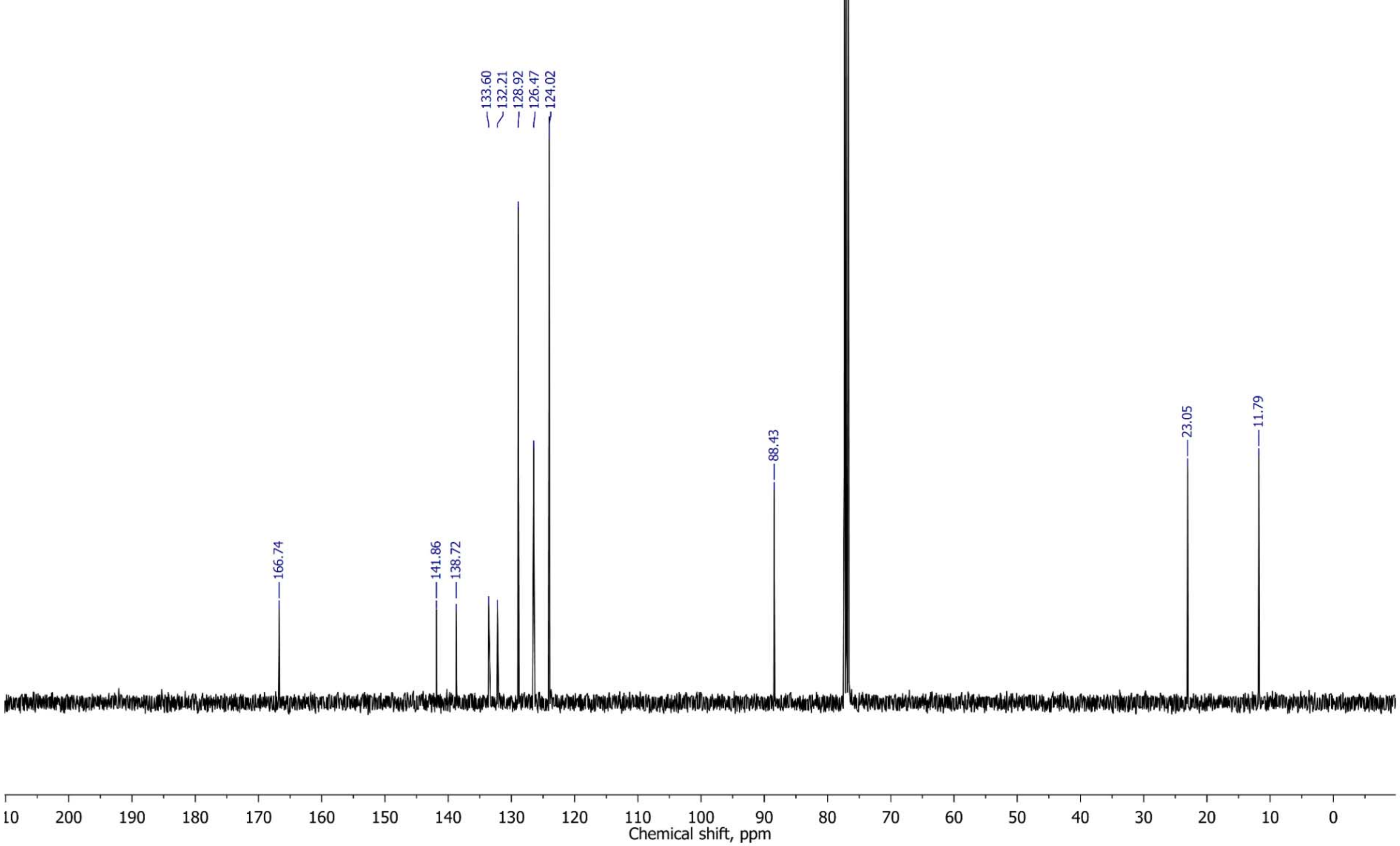

2-Ethyl-5-phenyl-4*H*-pyrrolo[2,3-*d*]oxazole 3d, DEPT, 100 MHz, $CDCl_3$

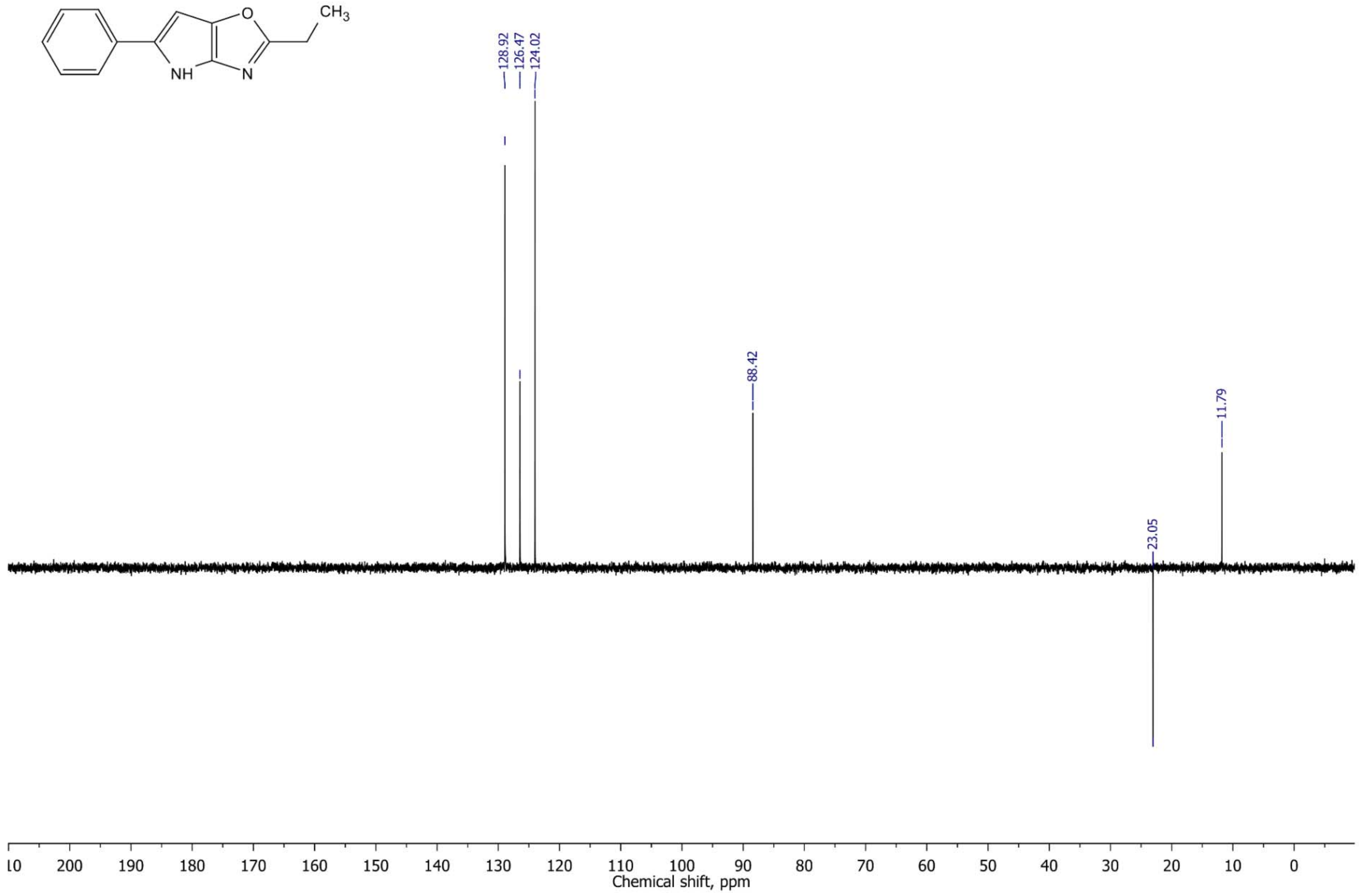

2-Ethyl-5-(4-methoxyphenyl)-4*H*-pyrrolo[2,3-*d*]oxazole 3e, ^{1}H NMR, 400 MHz, DMSO-d$_6$

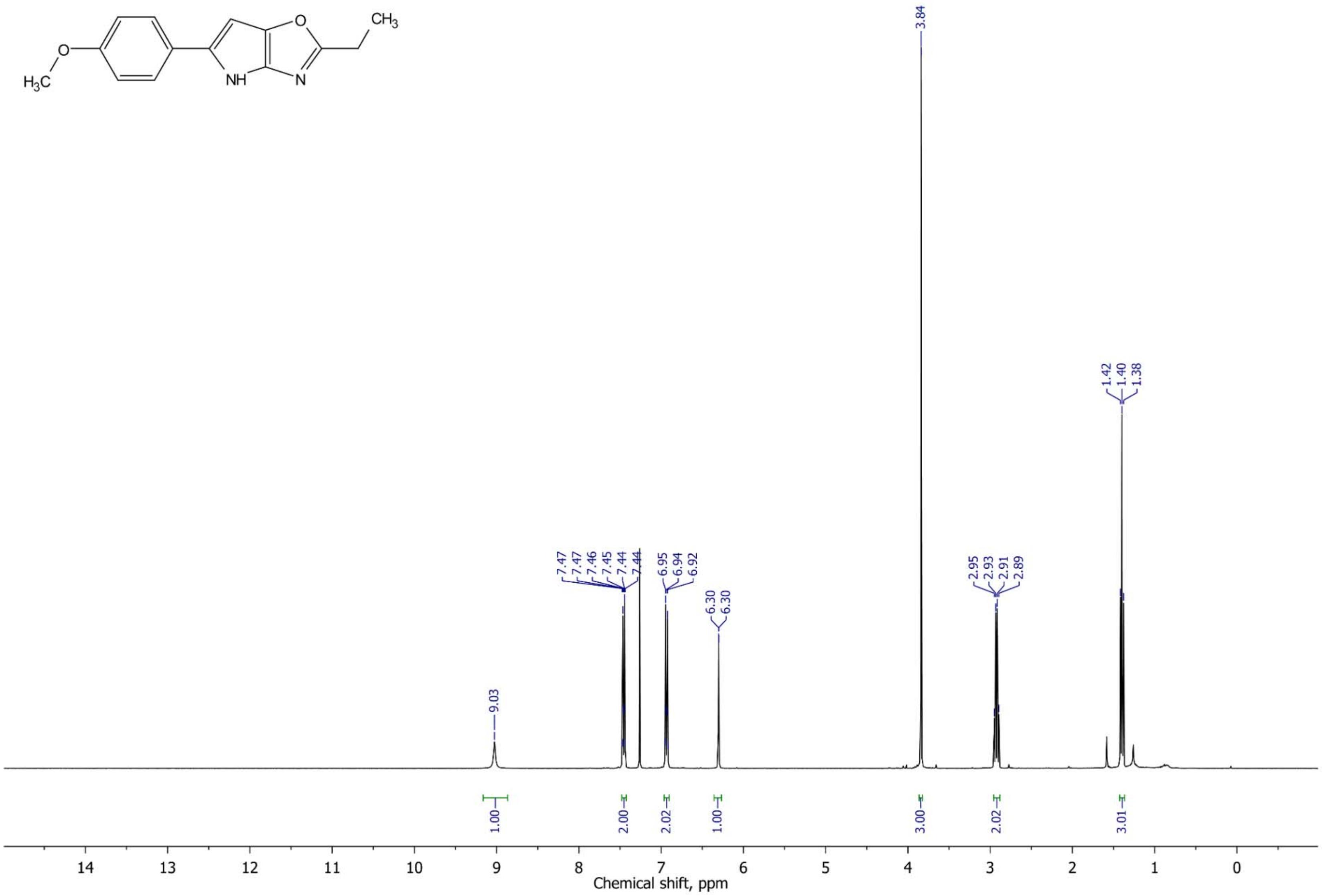

2-Ethyl-5-(4-methoxyphenyl)-4*H*-pyrrolo[2,3-*d*]oxazole 3e, $^{13}C\{^1H\}$ NMR, 100 MHz, DMSO-d_6

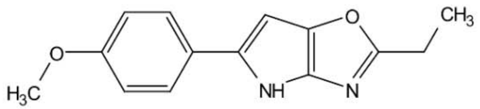

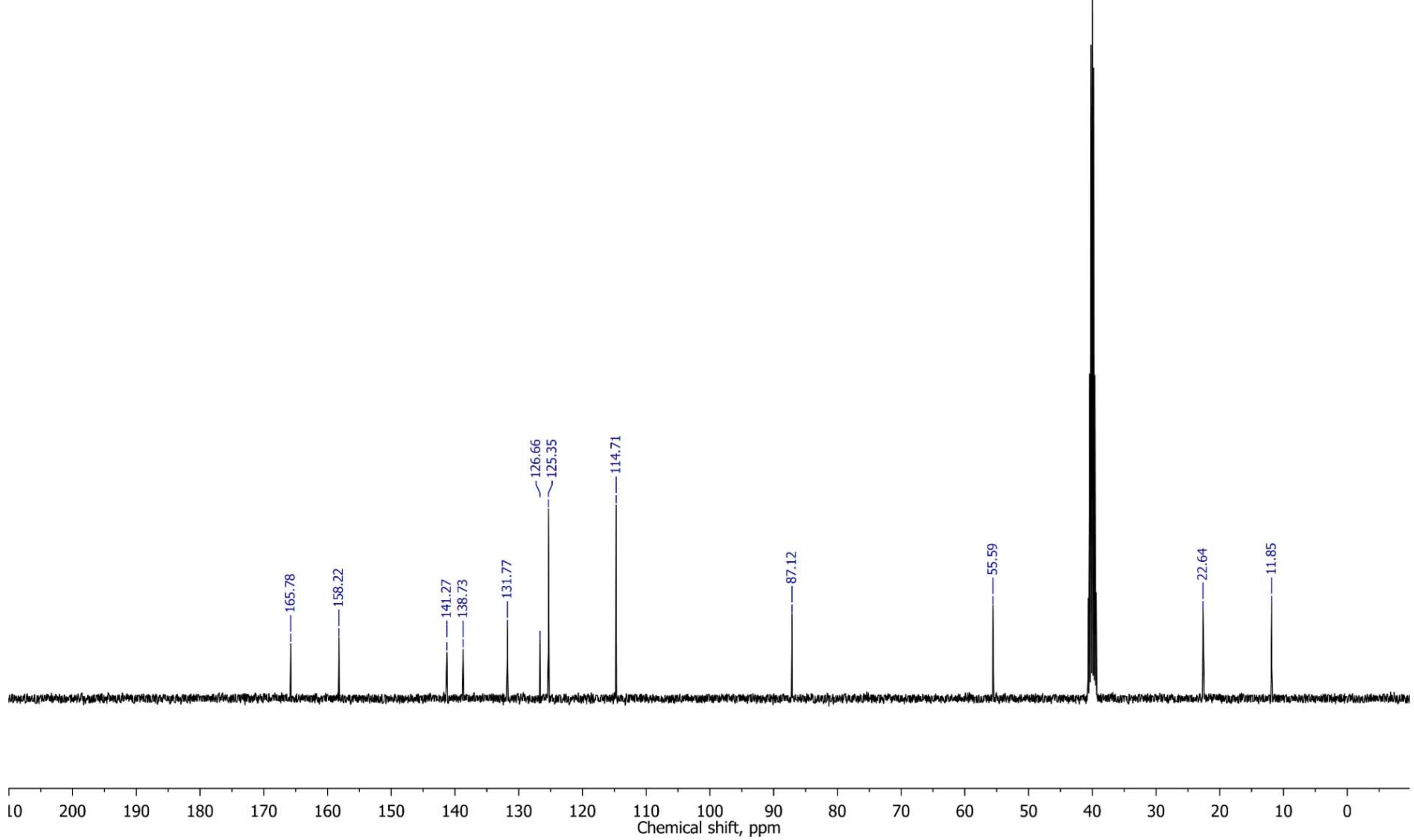

2-Ethyl-5-(4-methoxyphenyl)-4*H*-pyrrolo[2,3-*d*]oxazole 3e, DEPT NMR, 100 MHz, DMSO-d$_6$

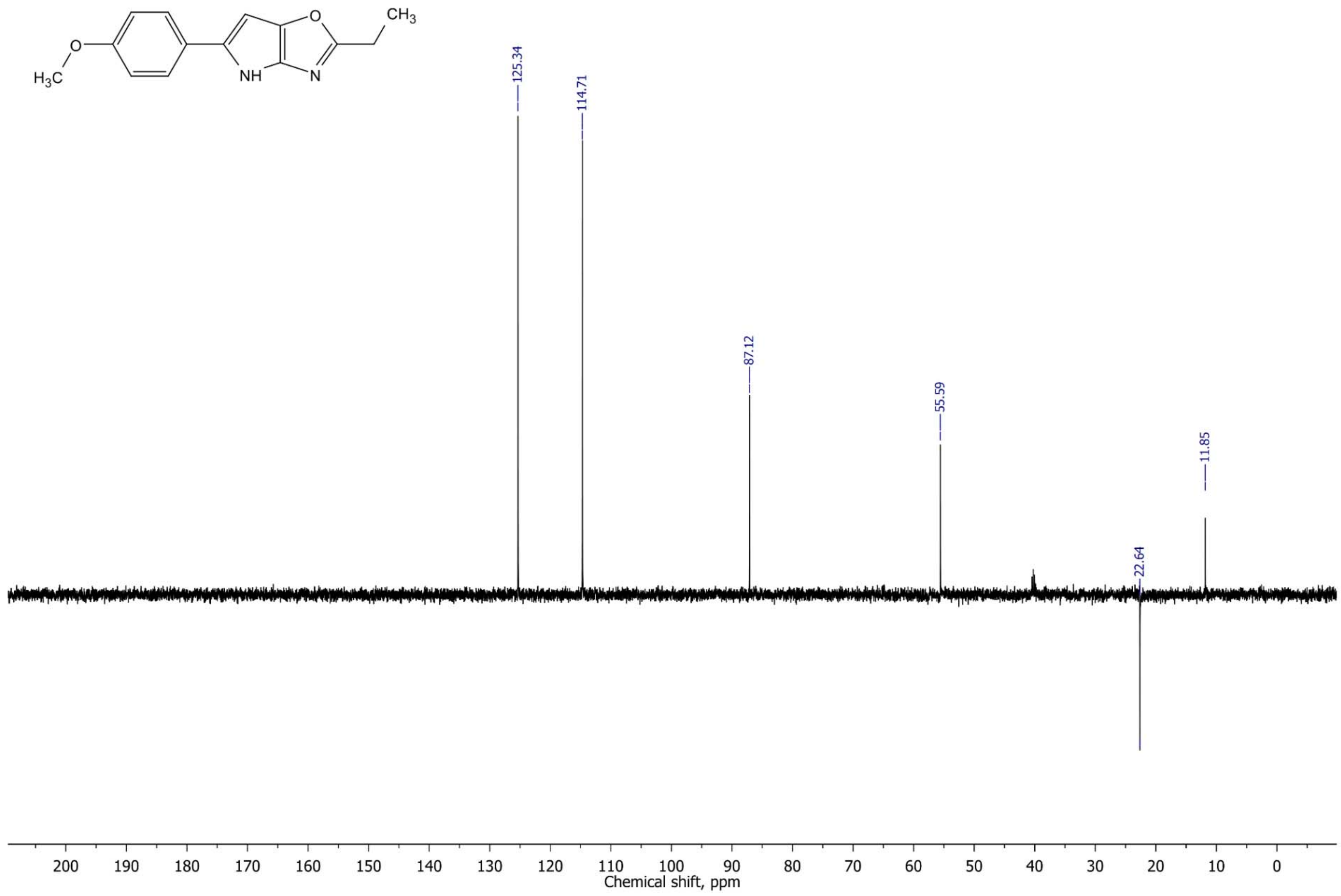

5-(*tert*-Butyl)-2-ethyl-4*H*-pyrrolo[2,3-*d*]oxazole 3f, ^{1}H NMR, 400 MHz, $CDCl_3$

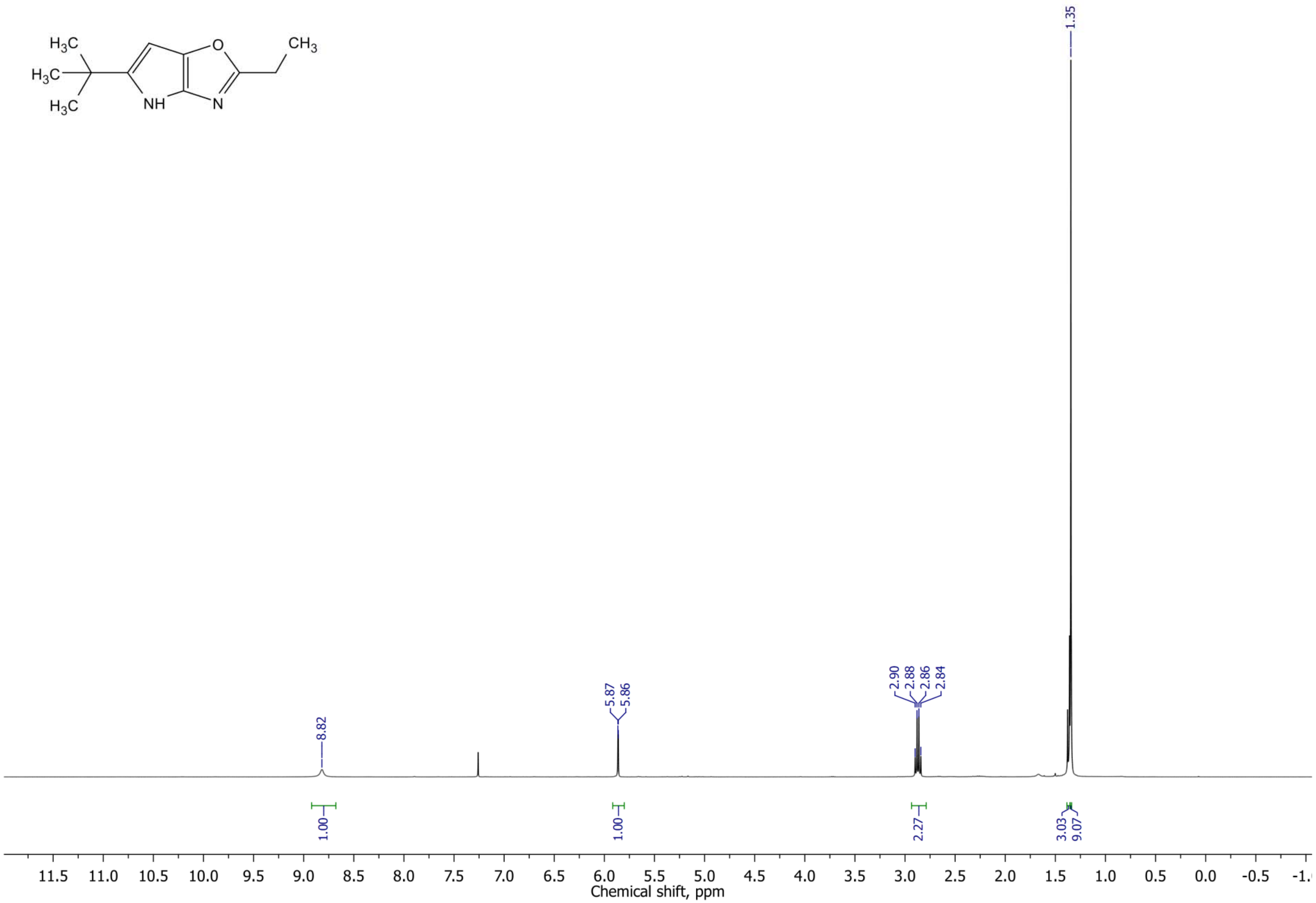

5-(*tert*-Butyl)-2-ethyl-4*H*-pyrrolo[2,3-*d*]oxazole 3f, $^{13}C\{^1H\}$ NMR, 100 MHz, $CDCl_3$

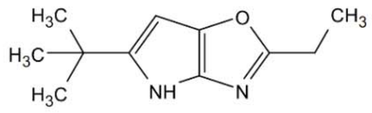

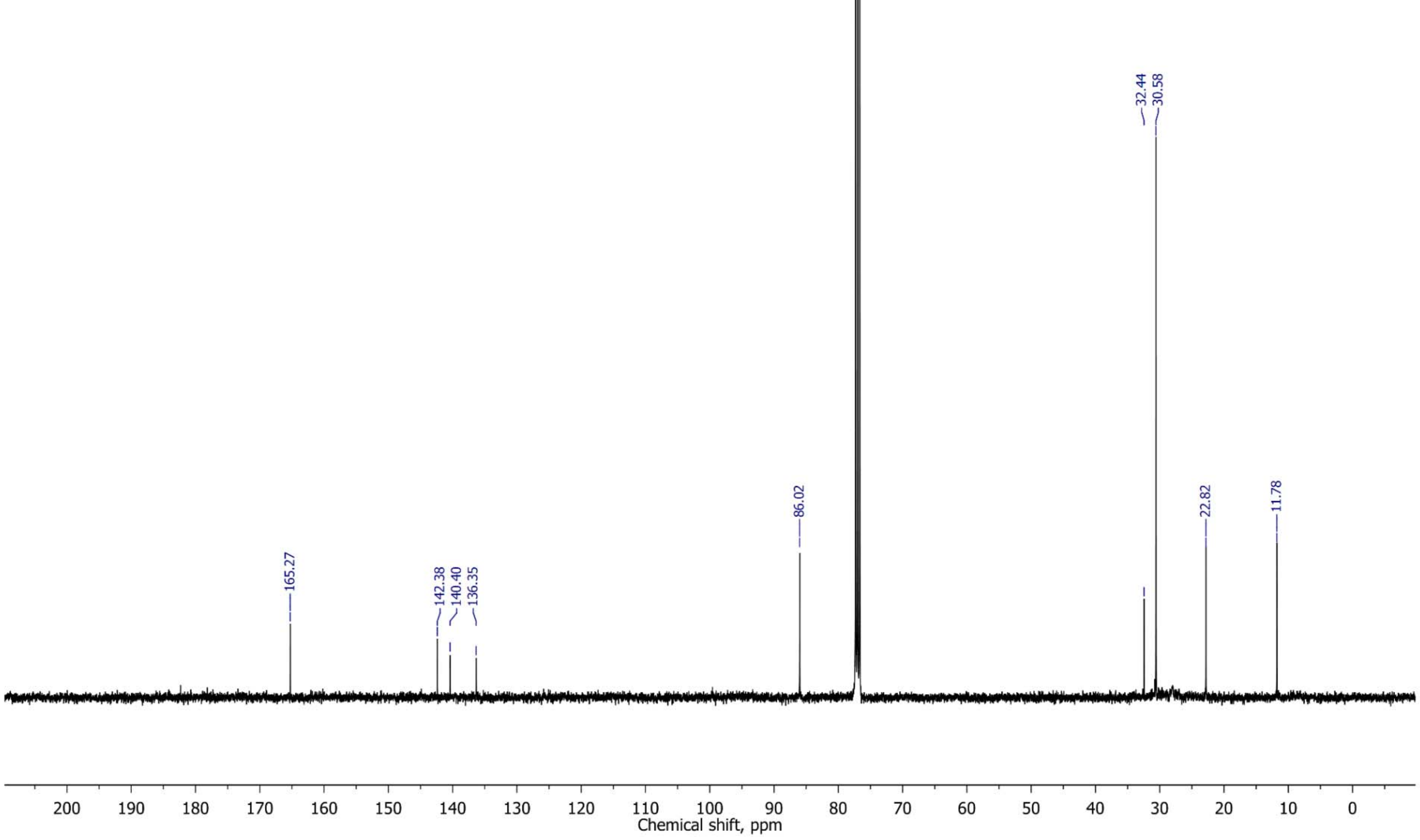

5-(*tert*-Butyl)-2-ethyl-4*H*-pyrrolo[2,3-*d*]oxazole 3f, DEPT, 100 MHz, $CDCl_3$

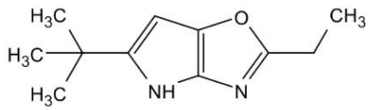

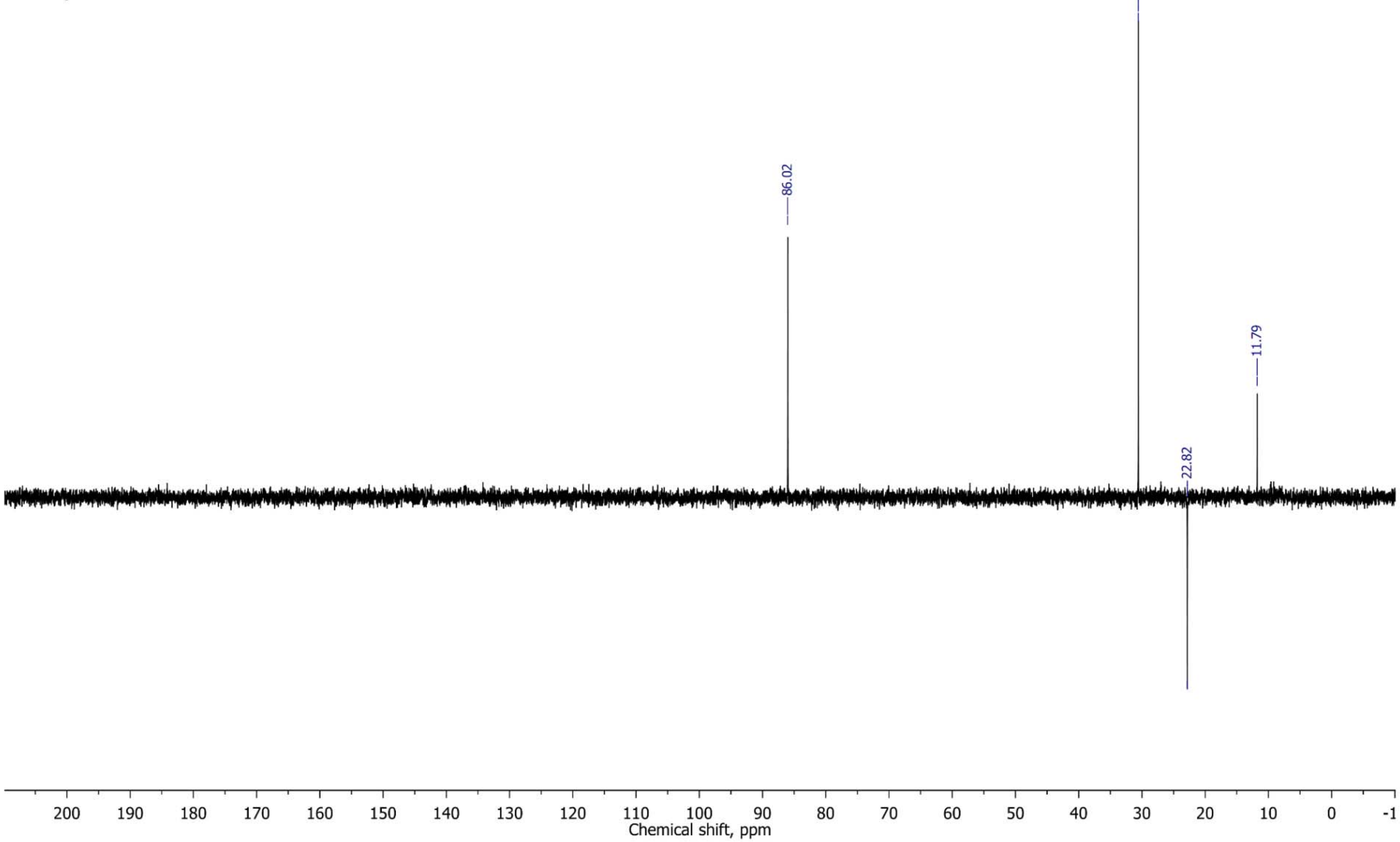

2-Benzyl-5-(4-bromophenyl)-4*H*-pyrrolo[2,3-*d*]oxazole 3g, ^{1}H NMR, 400 MHz, DMSO-d$_6$

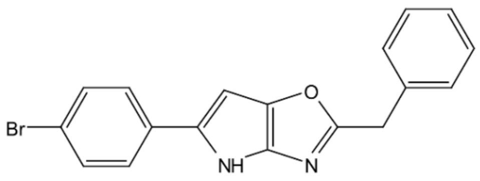

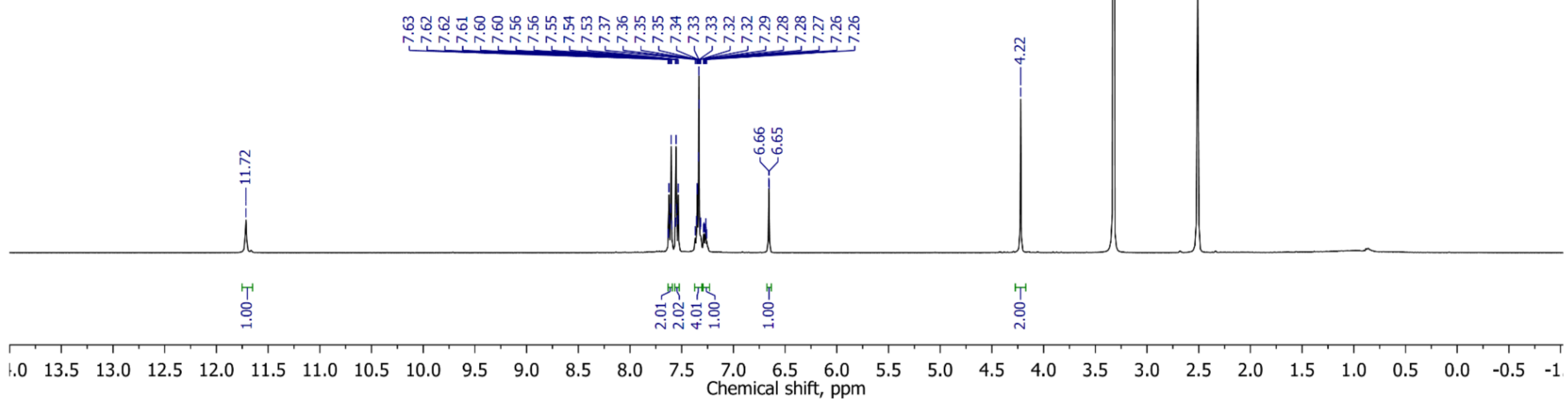

2-Benzyl-5-(4-bromophenyl)-4*H*-pyrrolo[2,3-*d*]oxazole 3g, $^{13}C\{^{1}H\}$ NMR, 100 MHz, DMSO-d_6

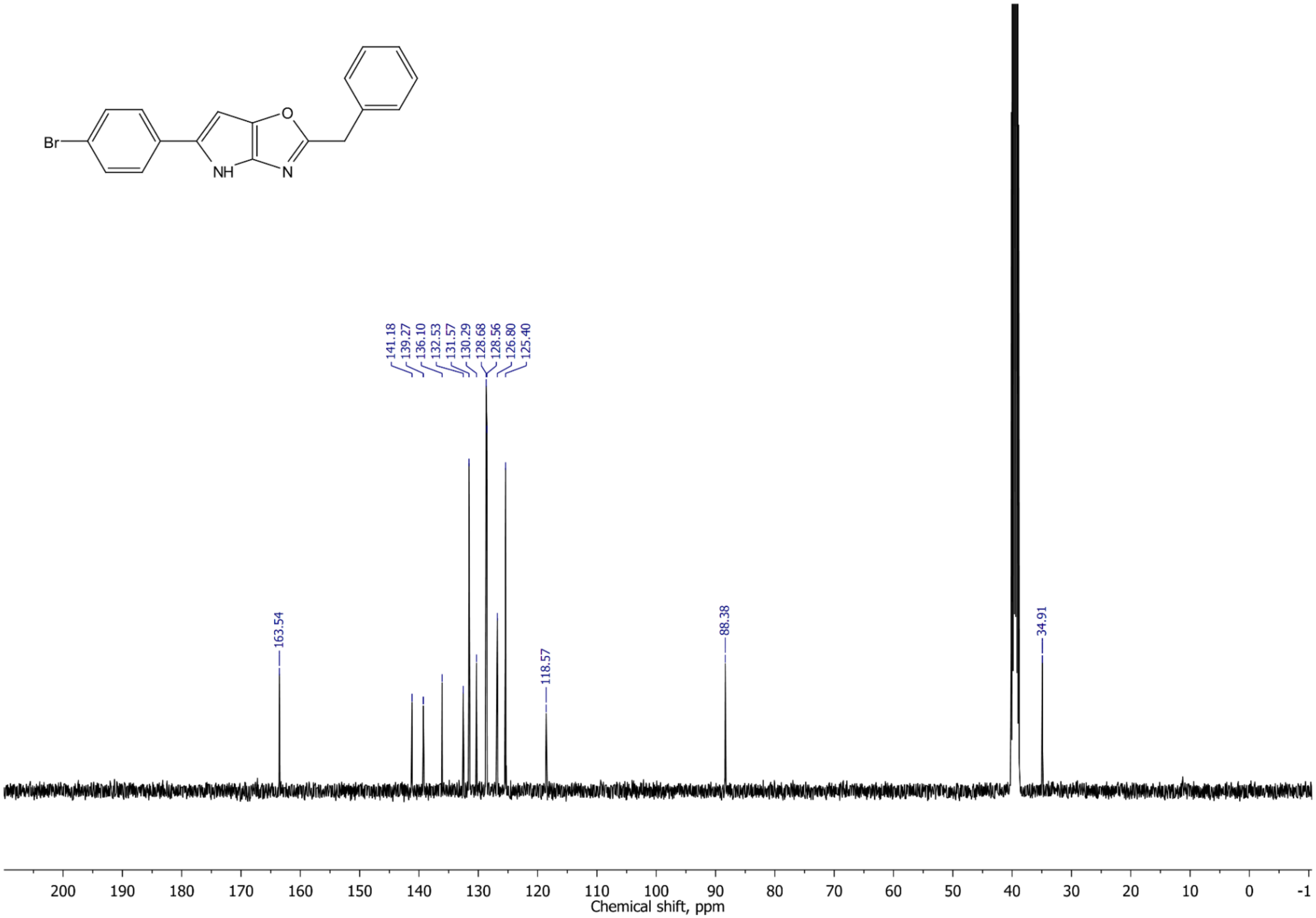

2-Benzyl-5-(4-bromophenyl)-4*H*-pyrrolo[2,3-*d*]oxazole 3g, DEPT, 100 MHz, DMSO-d_6

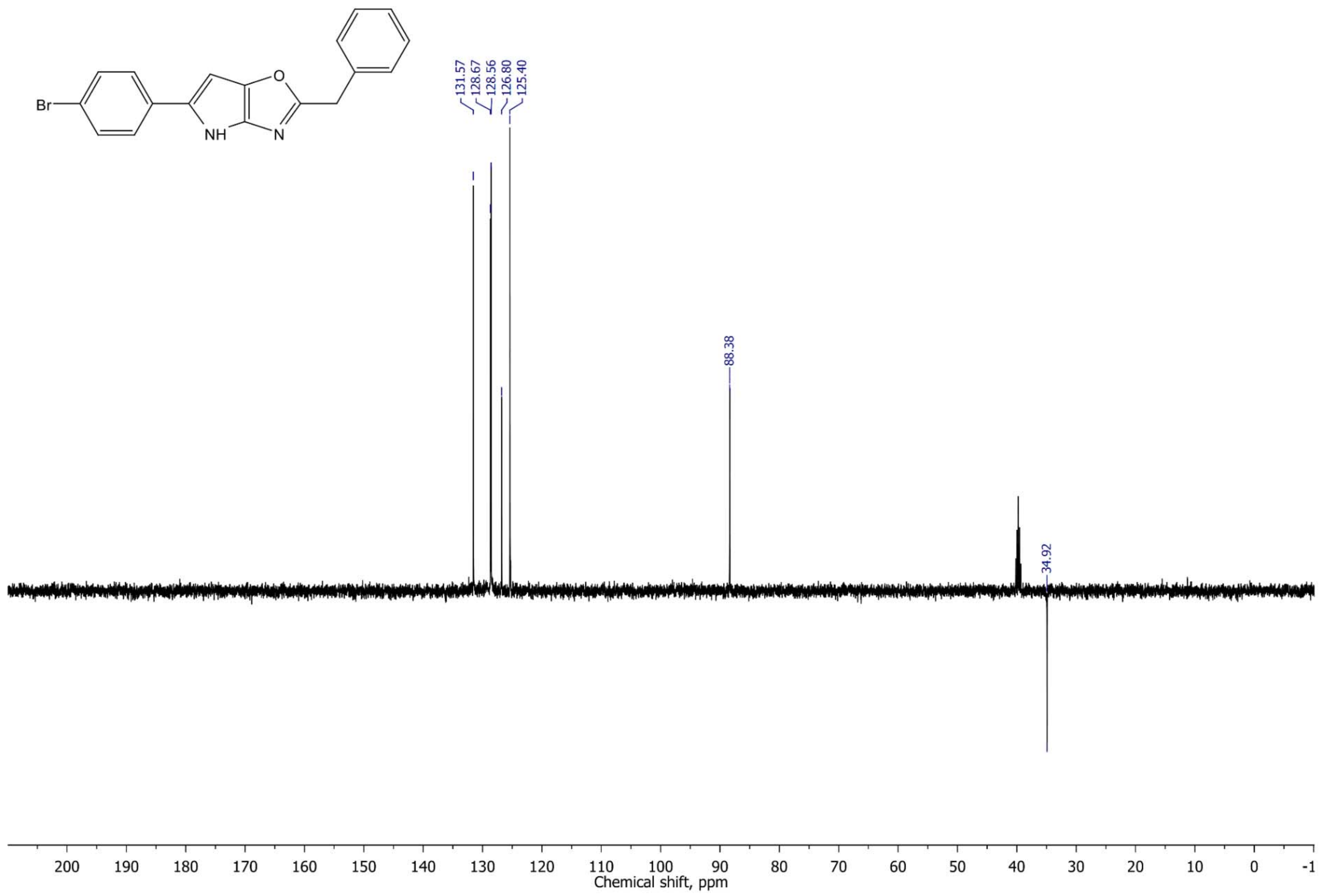

2,5-Diphenyl-4*H*-pyrrolo[2,3-*d*]oxazole 3h, ^{1}H NMR, 400 MHz, DMSO-d_6

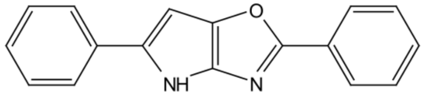

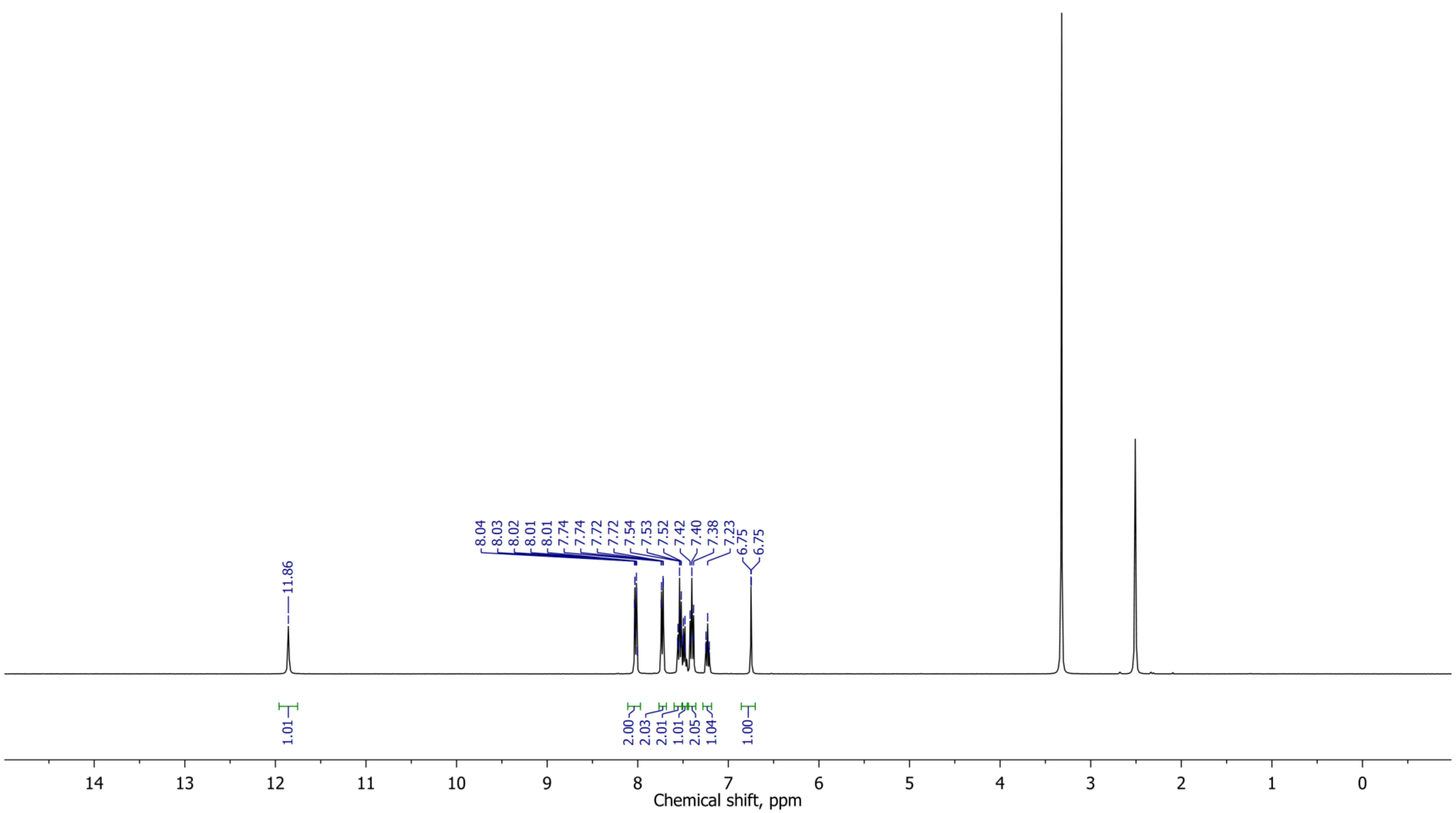

2,5-Diphenyl-4*H*-pyrrolo[2,3-*d*]oxazole 3h, $^{13}C\{^1H\}$ NMR, 100 MHz, DMSO-d$_6$

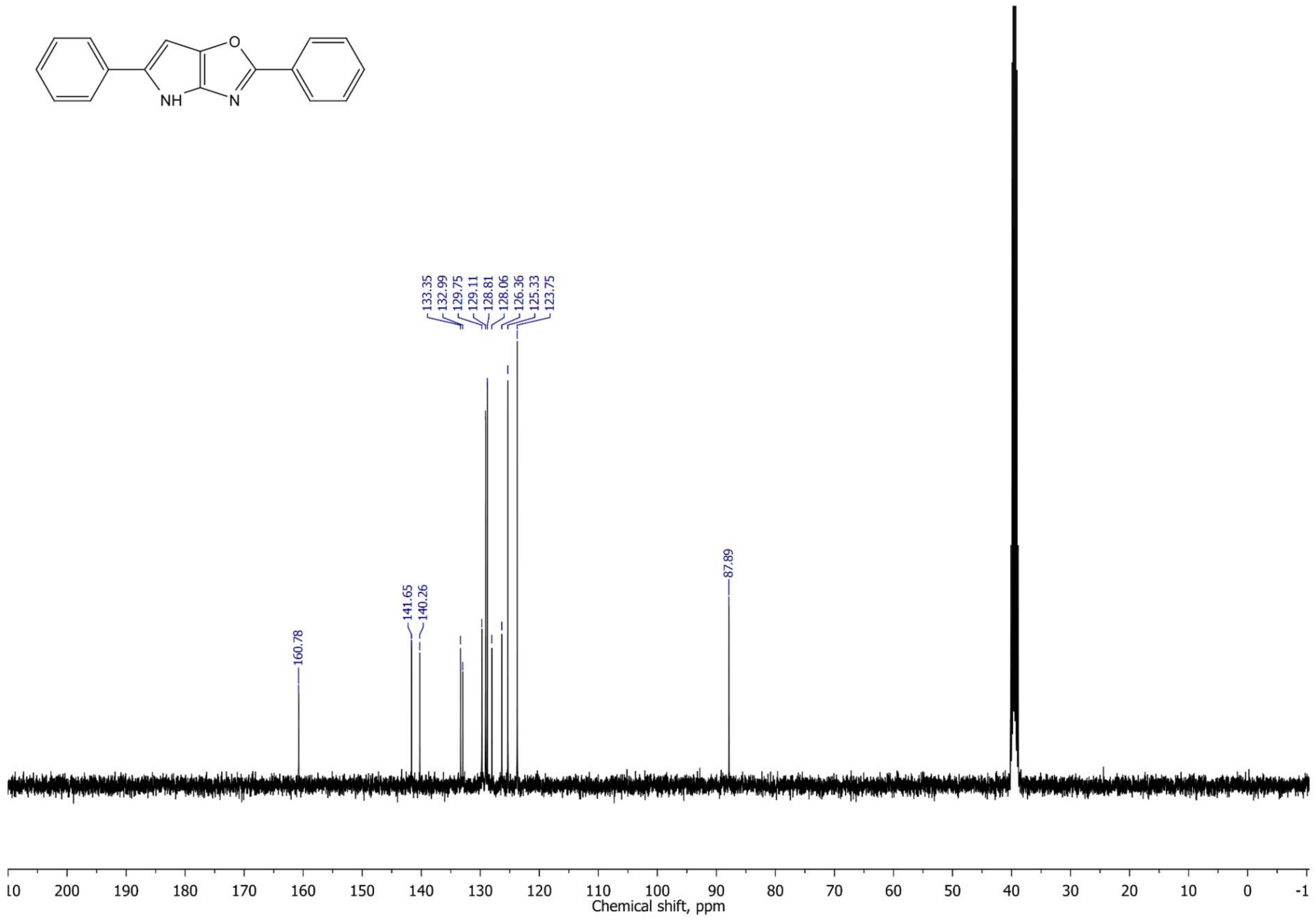

2,5-Diphenyl-4*H*-pyrrolo[2,3-*d*]oxazole 3h, DEPT, 100 MHz, DMSO-d_6

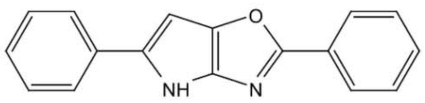

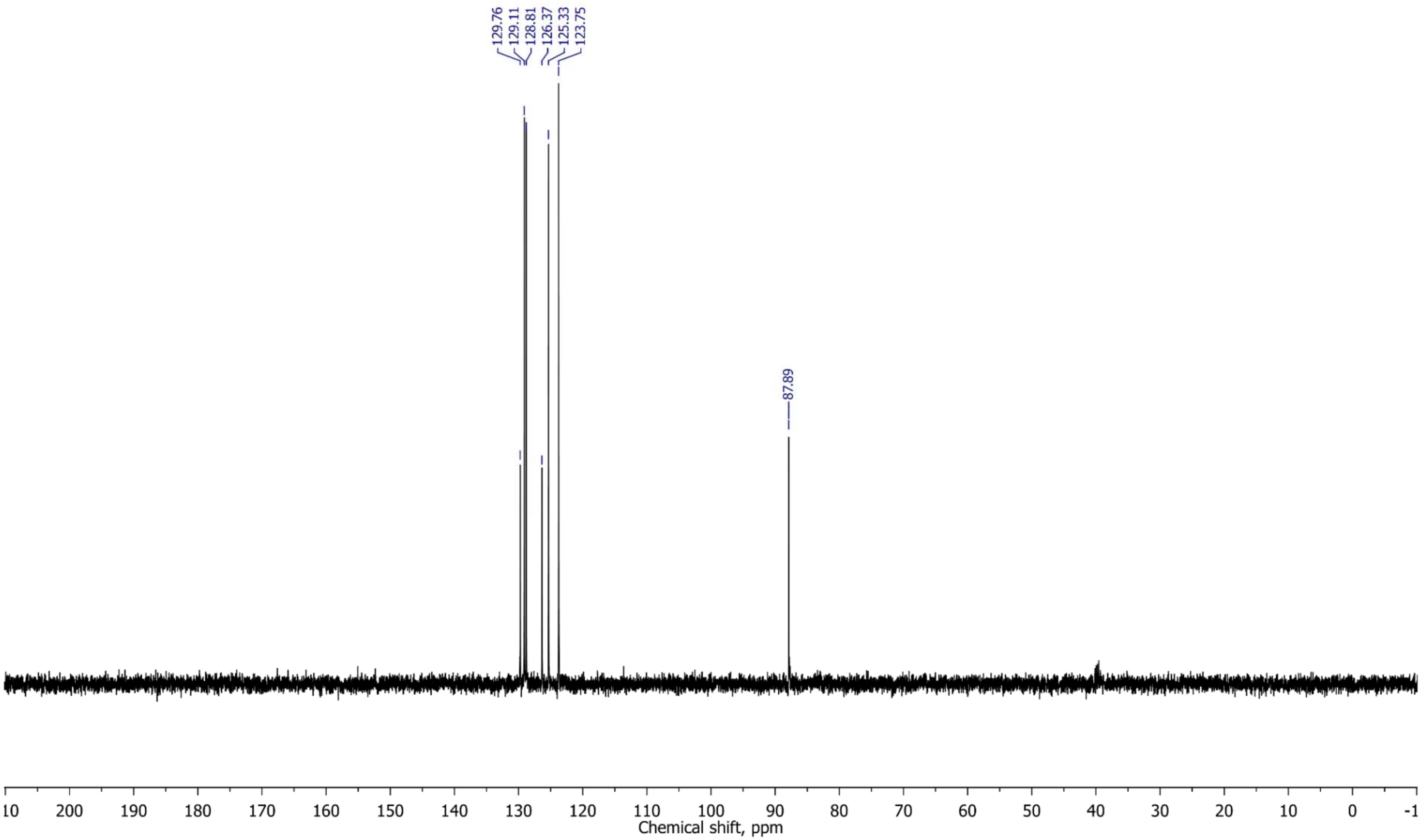

5-(4-Fluorophenyl)-2-phenyl-4*H*-pyrrolo[2,3-*d*]oxazole 3i, ^{1}H NMR, 400 MHz, DMSO-d$_6$

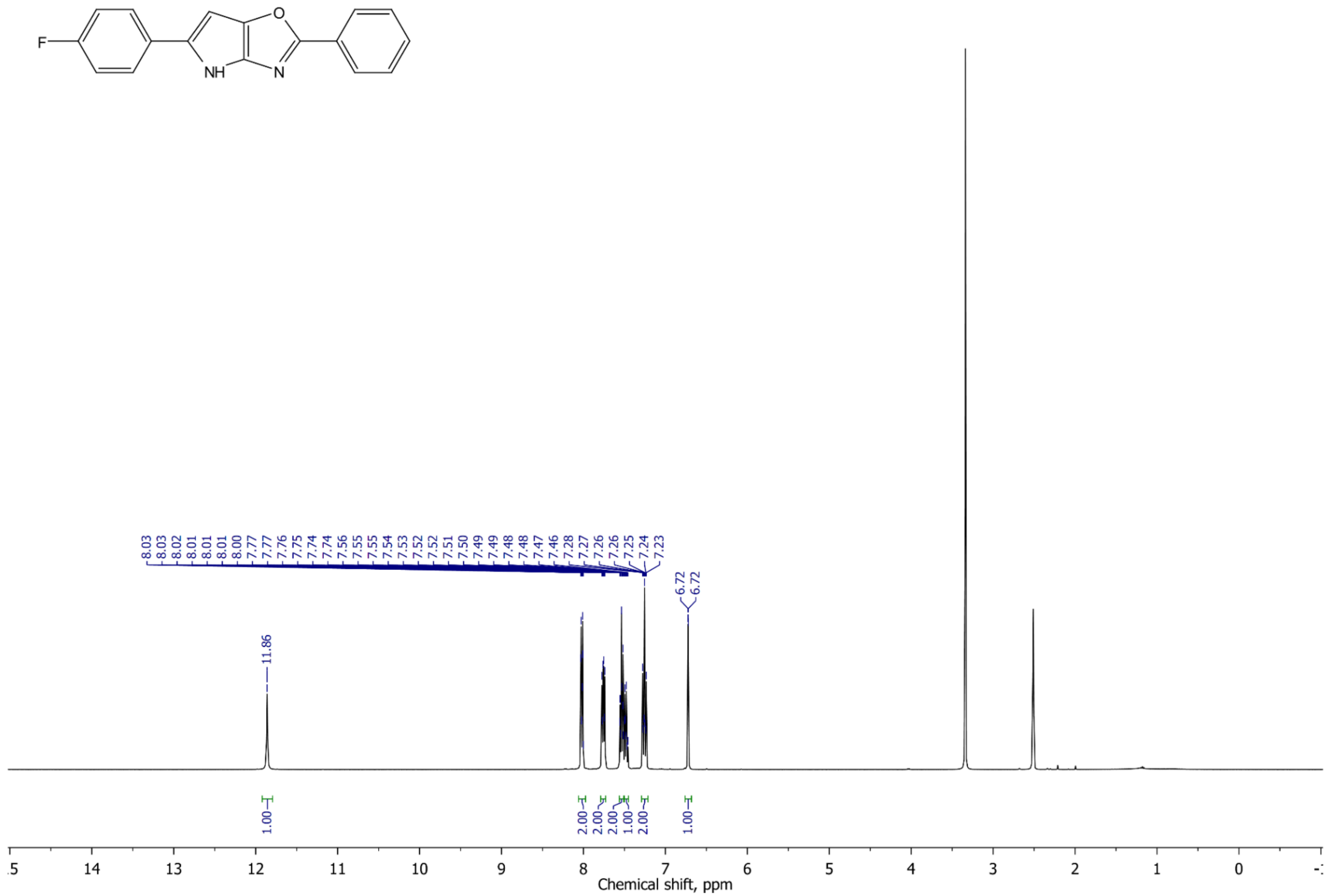

5-(4-Fluorophenyl)-2-phenyl-4*H*-pyrrolo[2,3-*d*]oxazole 3i, $^{13}C\{^{1}H\}$ NMR, 100 MHz, DMSO-d$_6$

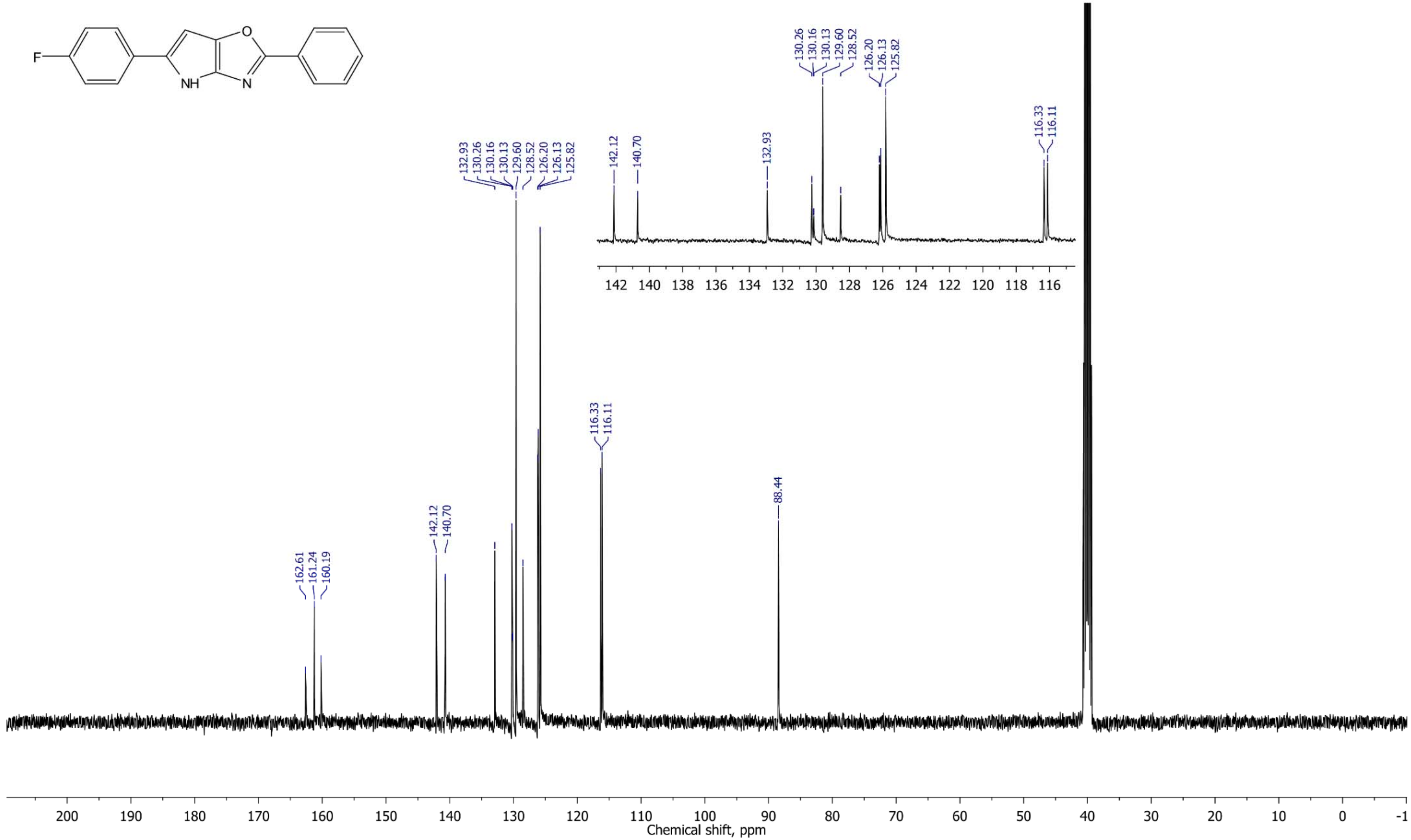

5-(4-Fluorophenyl)-2-phenyl-4*H*-pyrrolo[2,3-*d*]oxazole 3i, DEPT, 100 MHz, DMSO-d_6

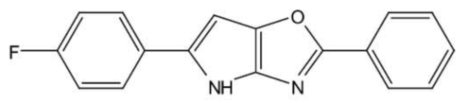

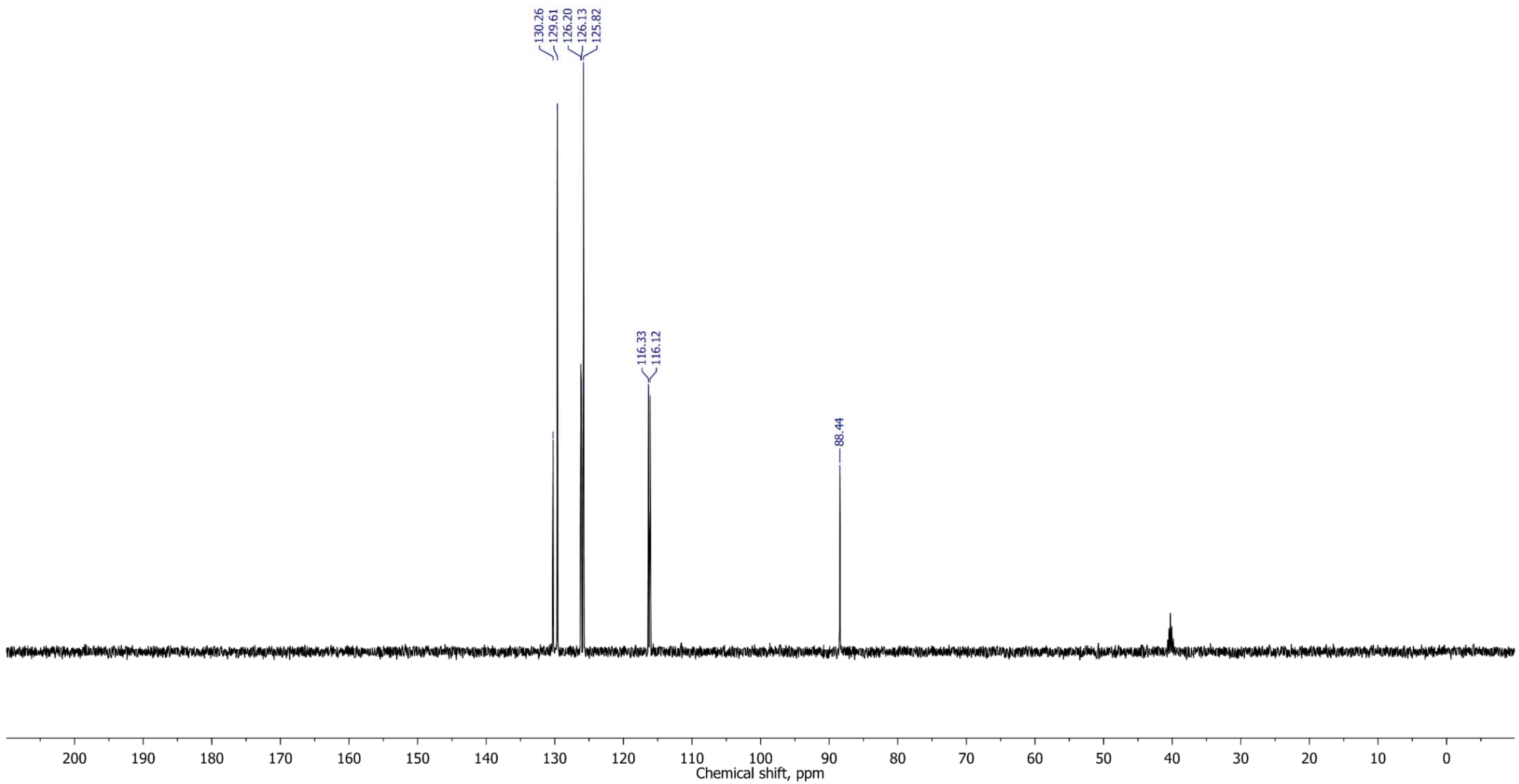

5-(*tert*-Butyl)-2-phenyl-4*H*-pyrrolo[2,3-*d*]oxazole 3j, ^{1}H NMR, 400 MHz, $CDCl_3$

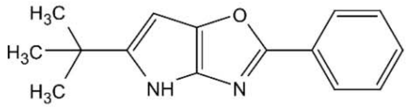

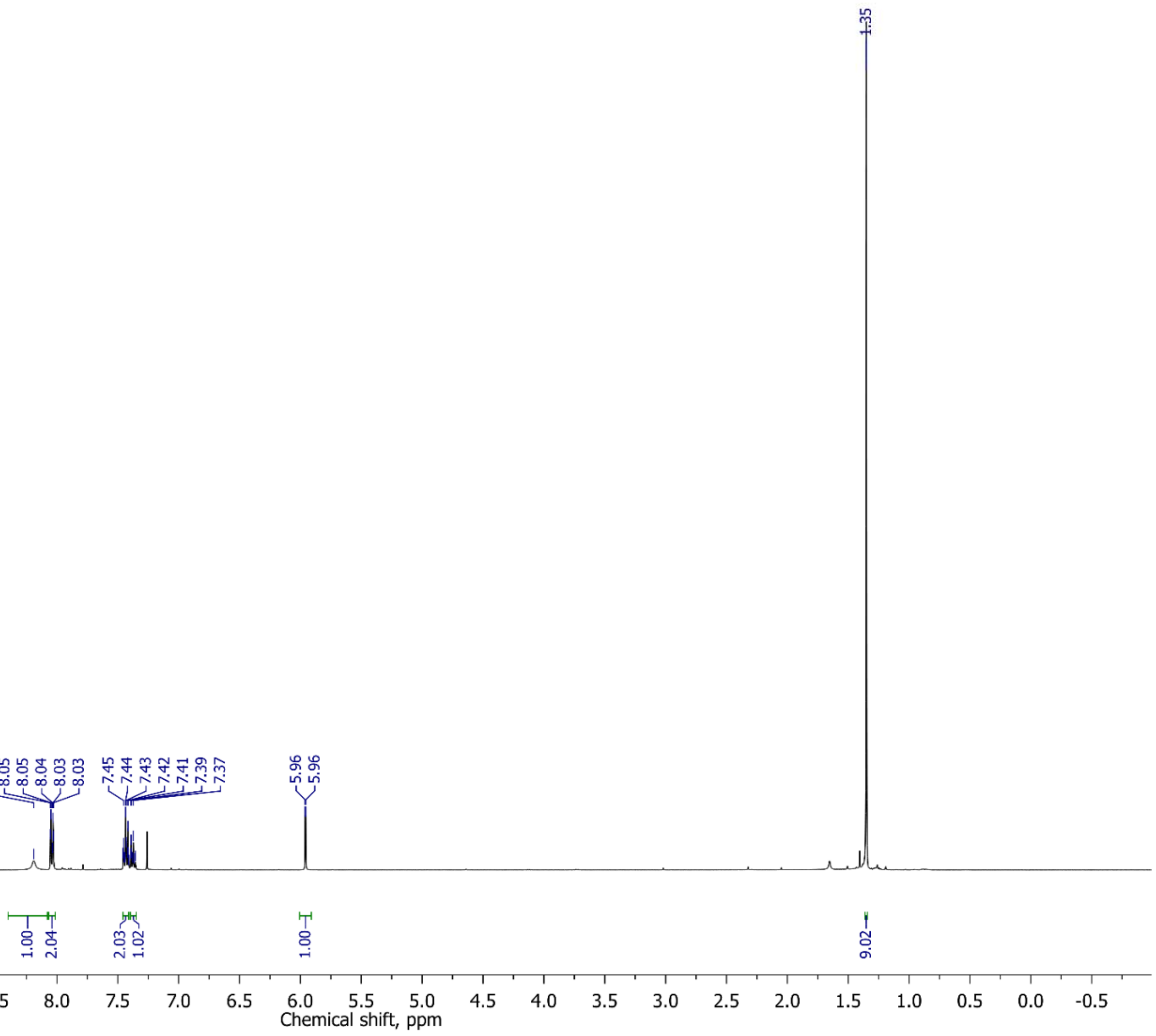

5-(*tert*-Butyl)-2-phenyl-4*H*-pyrrolo[2,3-*d*]oxazole 3j, $^{13}C\{^{1}H\}$ NMR, 100 MHz, $CDCl_3$

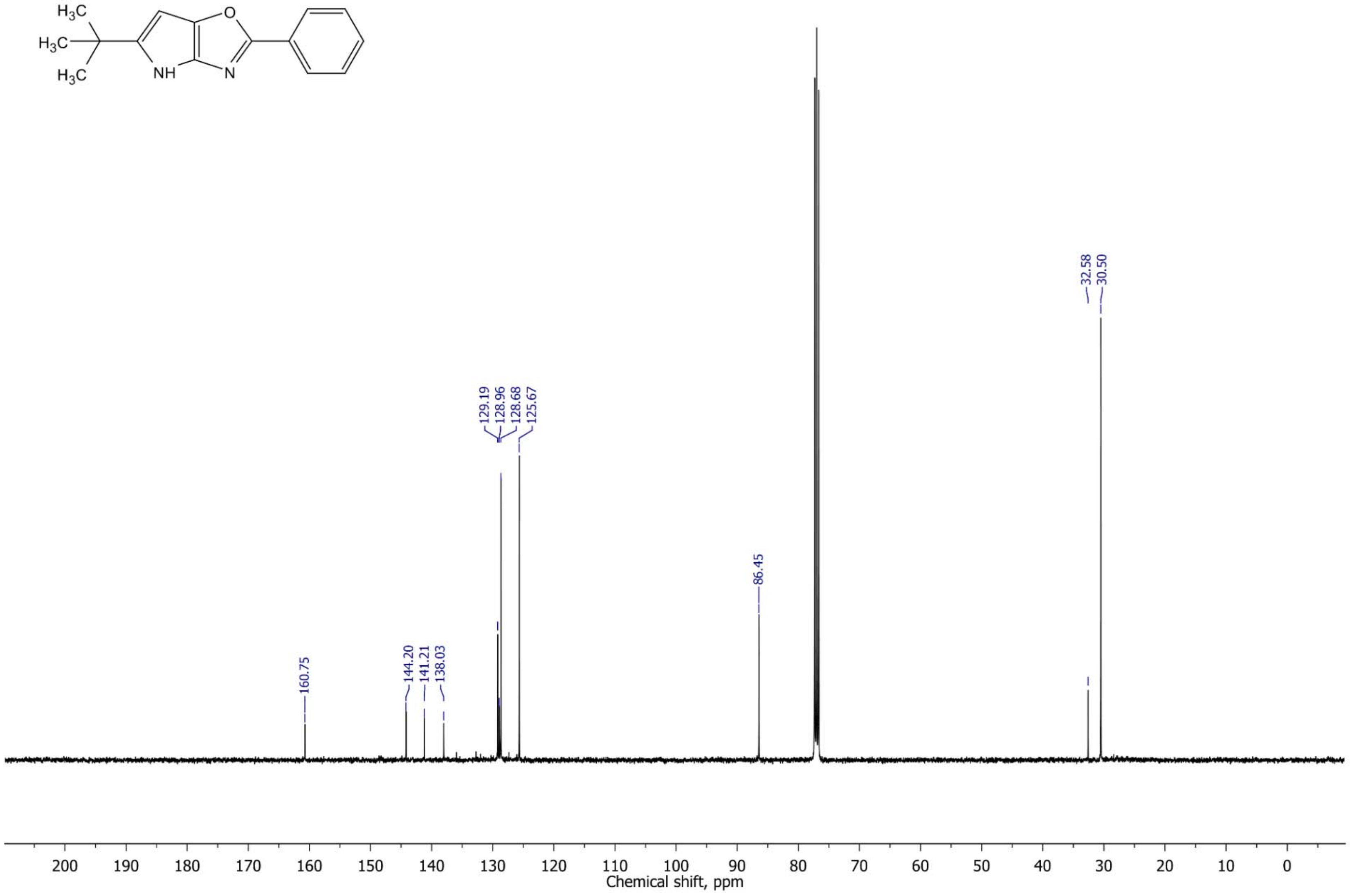

5-(*tert*-Butyl)-2-phenyl-4*H*-pyrrolo[2,3-*d*]oxazole 3j, DEPT, 100 MHz, CDCl$_3$

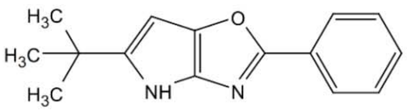

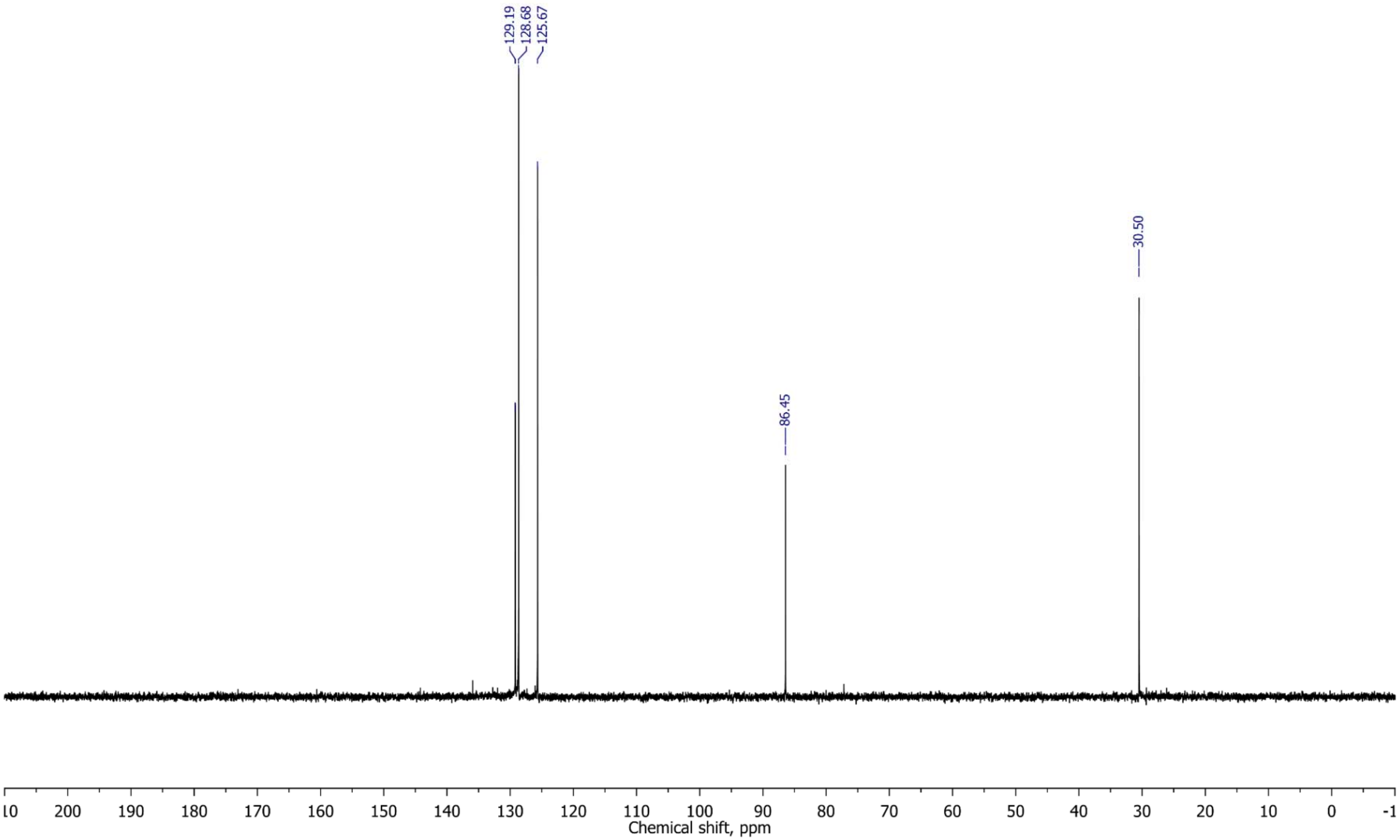

5-(Adamantan-1-yl)-2-phenyl-4*H*-pyrrolo[2,3-*d*]oxazole 3k, ^{1}H NMR, 400 MHz, $CDCl_3$

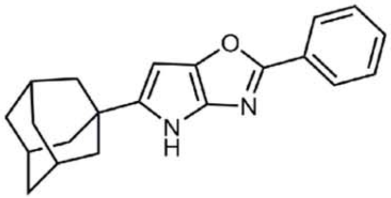

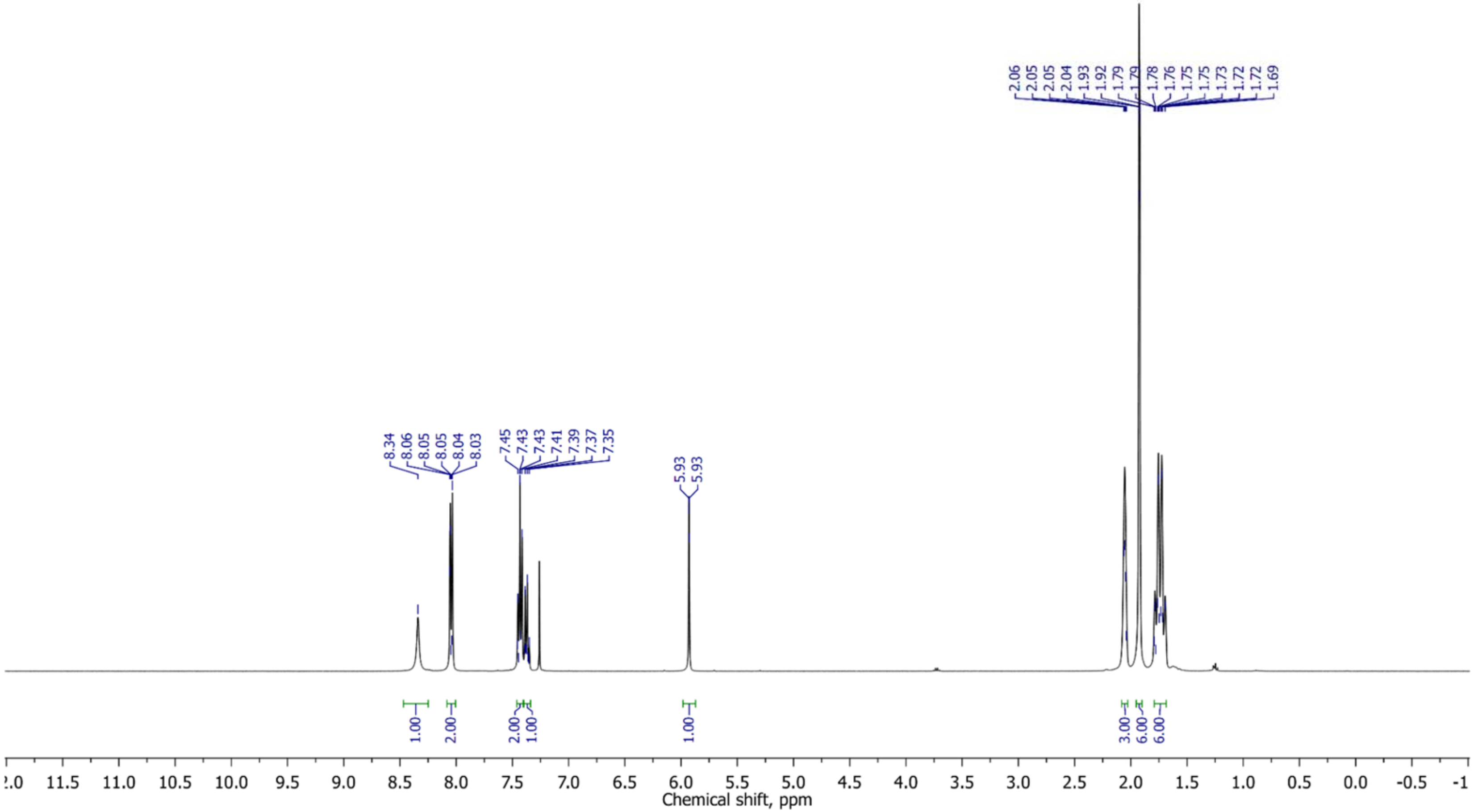

5-(Adamantan-1-yl)-2-phenyl-4*H*-pyrrolo[2,3-*d*]oxazole 3k, $^{13}C\{^1H\}$ NMR, 100 MHz, $CDCl_3$

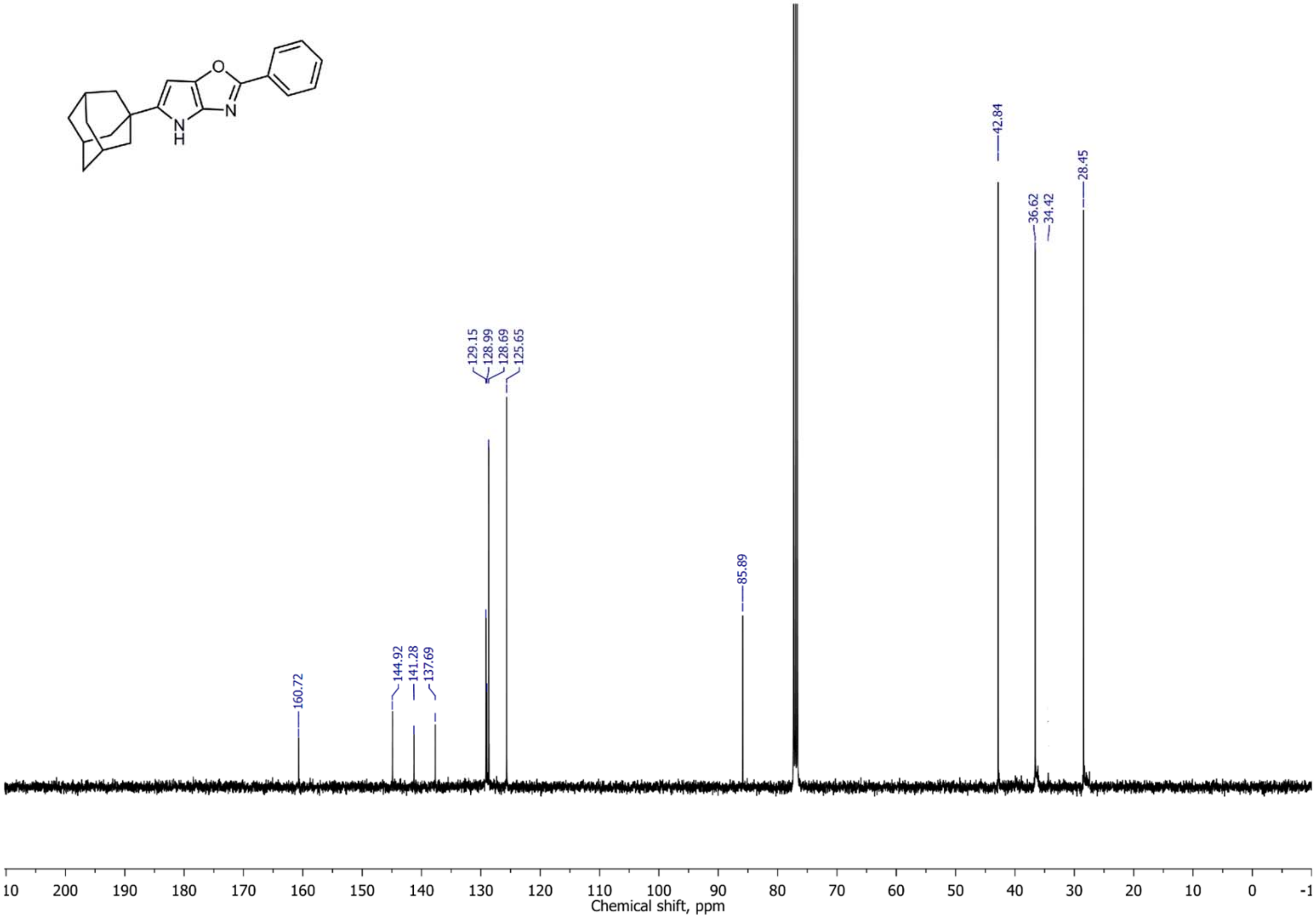

5-(Adamantan-1-yl)-2-phenyl-4*H*-pyrrolo[2,3-*d*]oxazole 3k, DEPT, 100 MHz, $CDCl_3$

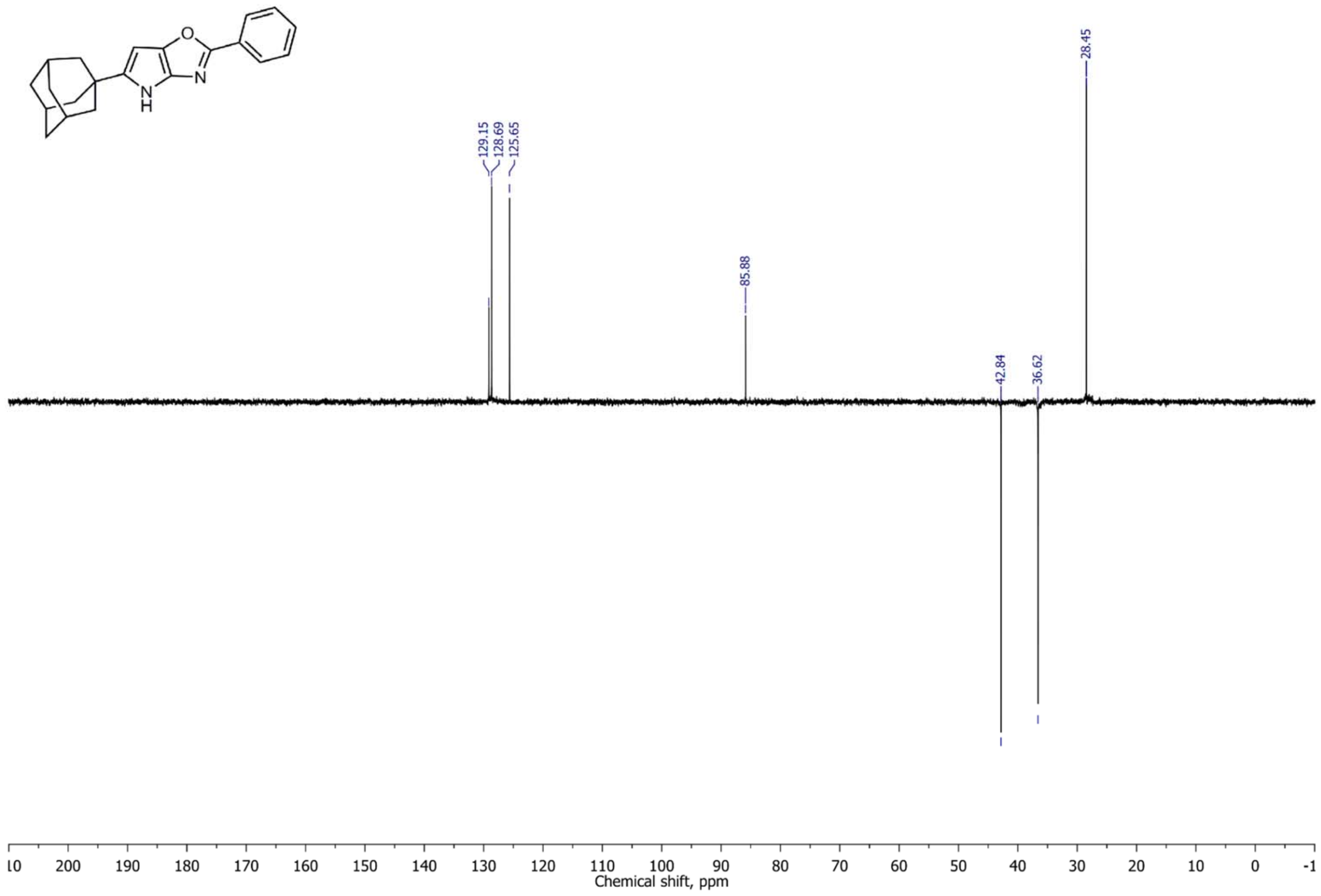

5-(4-Methoxyphenyl)-2-(*p*-tolyl)-4*H*-pyrrolo[2,3-*d*]oxazole 3l, ^{1}H NMR, 400 MHz, DMSO-d$_6$

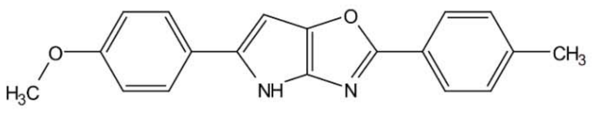

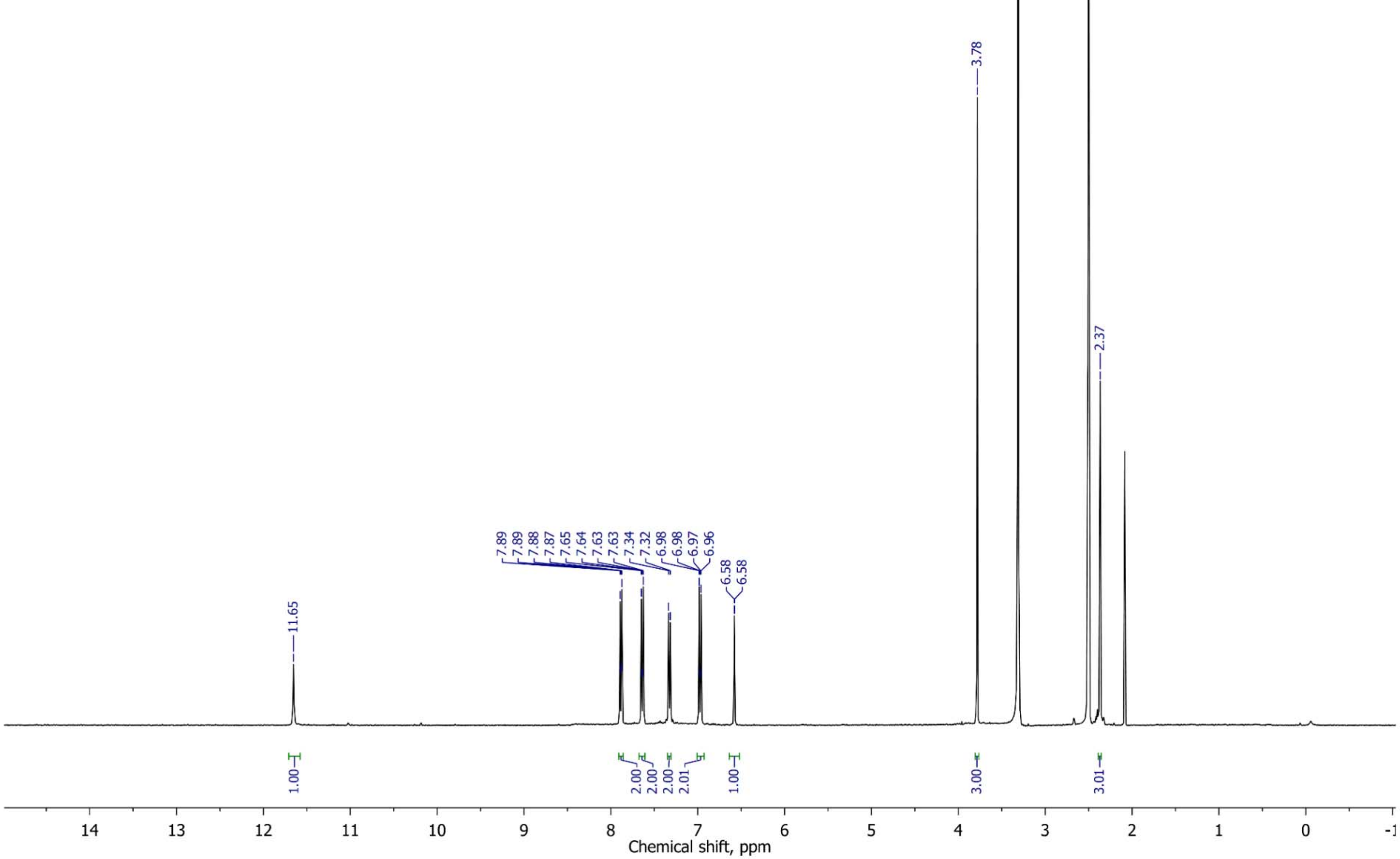

5-(4-Methoxyphenyl)-2-(*p*-tolyl)-4*H*-pyrrolo[2,3-*d*]oxazole 3l, $^{13}C\{^1H\}$ NMR, 100 MHz, DMSO-d_6

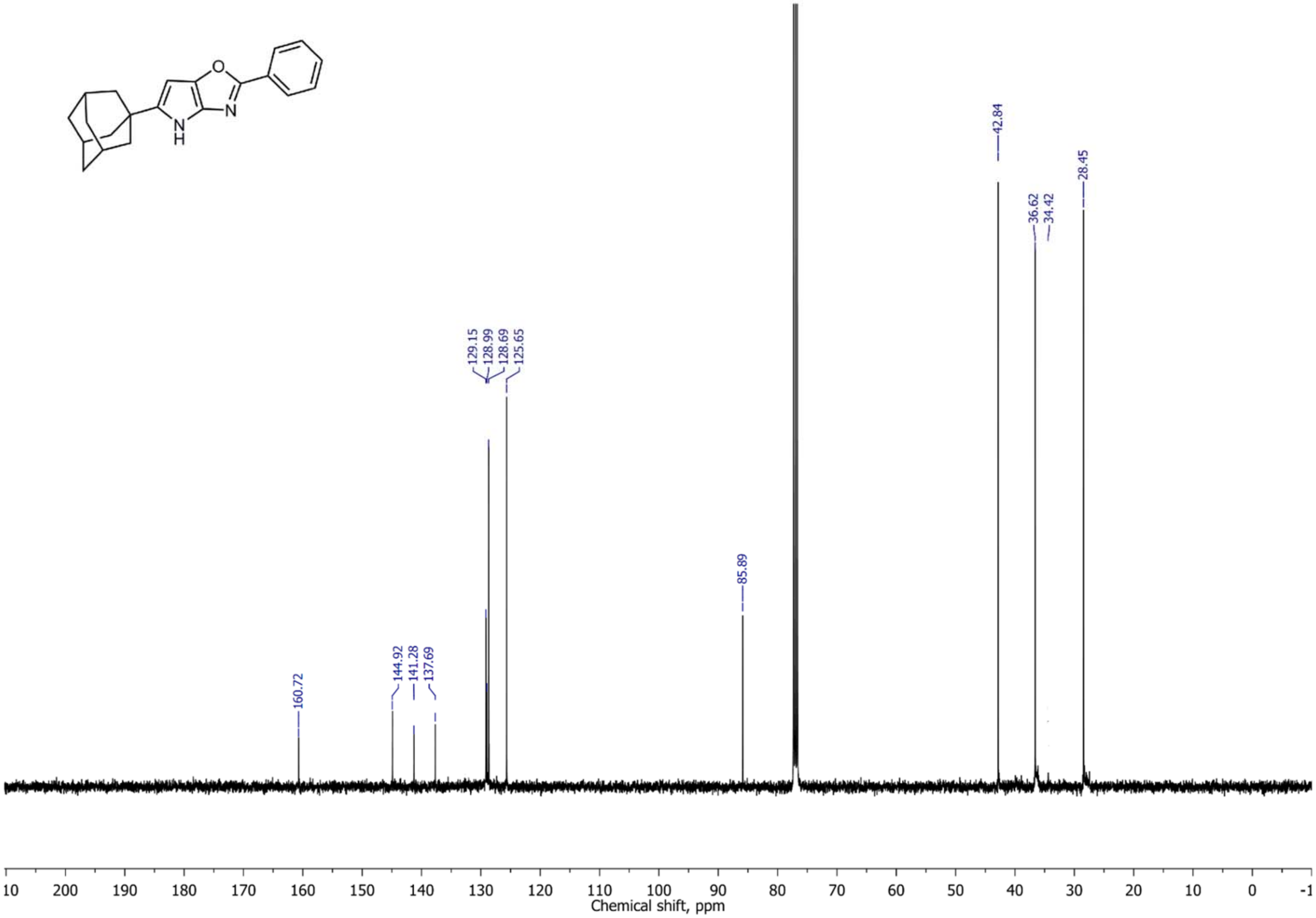

5-(4-Methoxyphenyl)-2-(*p*-tolyl)-4*H*-pyrrolo[2,3-*d*]oxazole 3l, DEPT, 100 MHz, DMSO-d_6

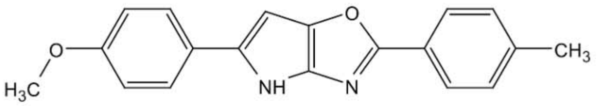

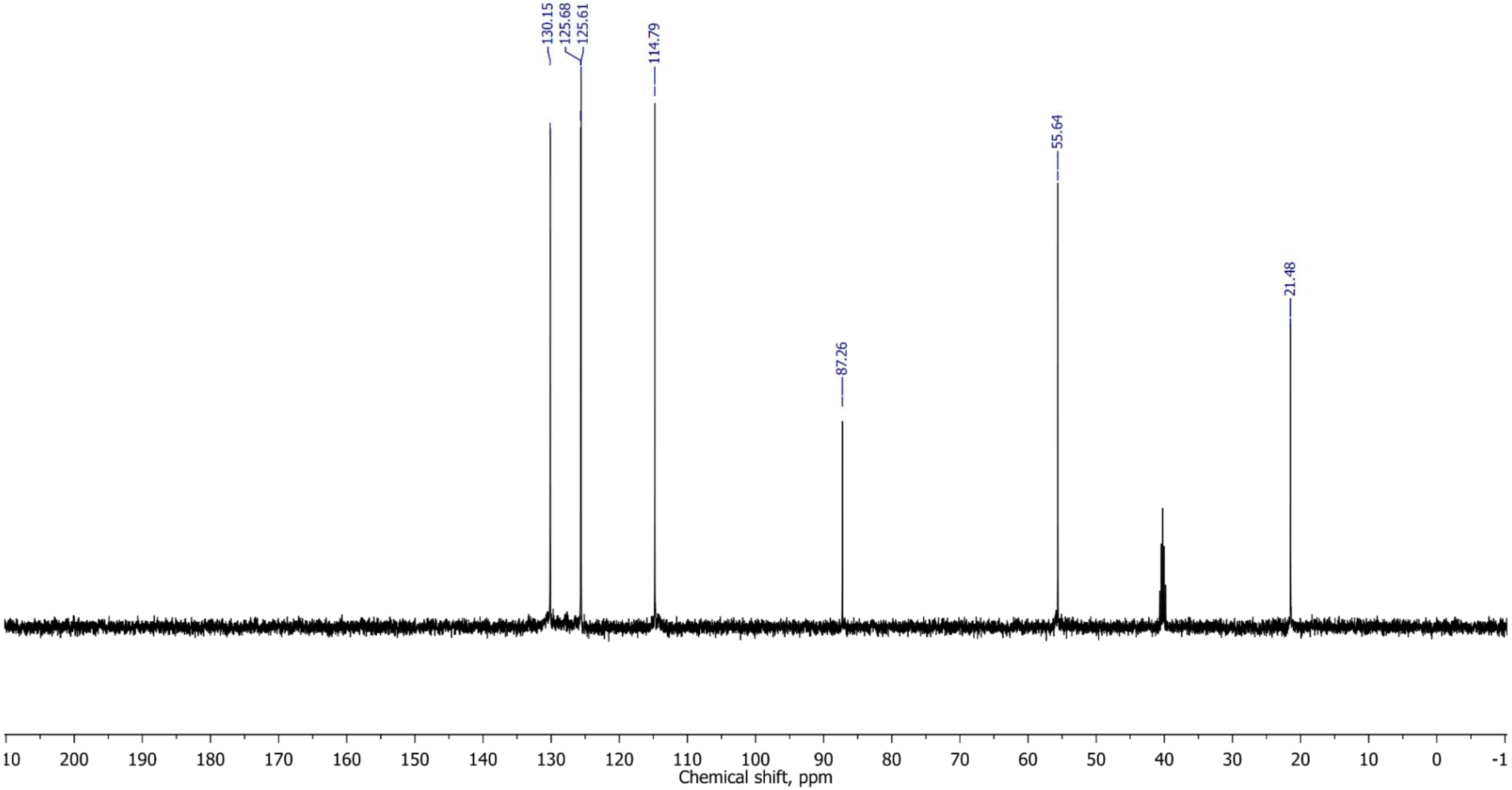

5-(4-Chlorophenyl)-2-(*p*-tolyl)-4*H*-pyrrolo[2,3-*d*]oxazole 3m, ^{1}H NMR, 400 MHz, DMSO-d$_6$

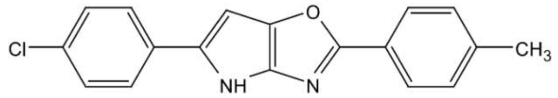

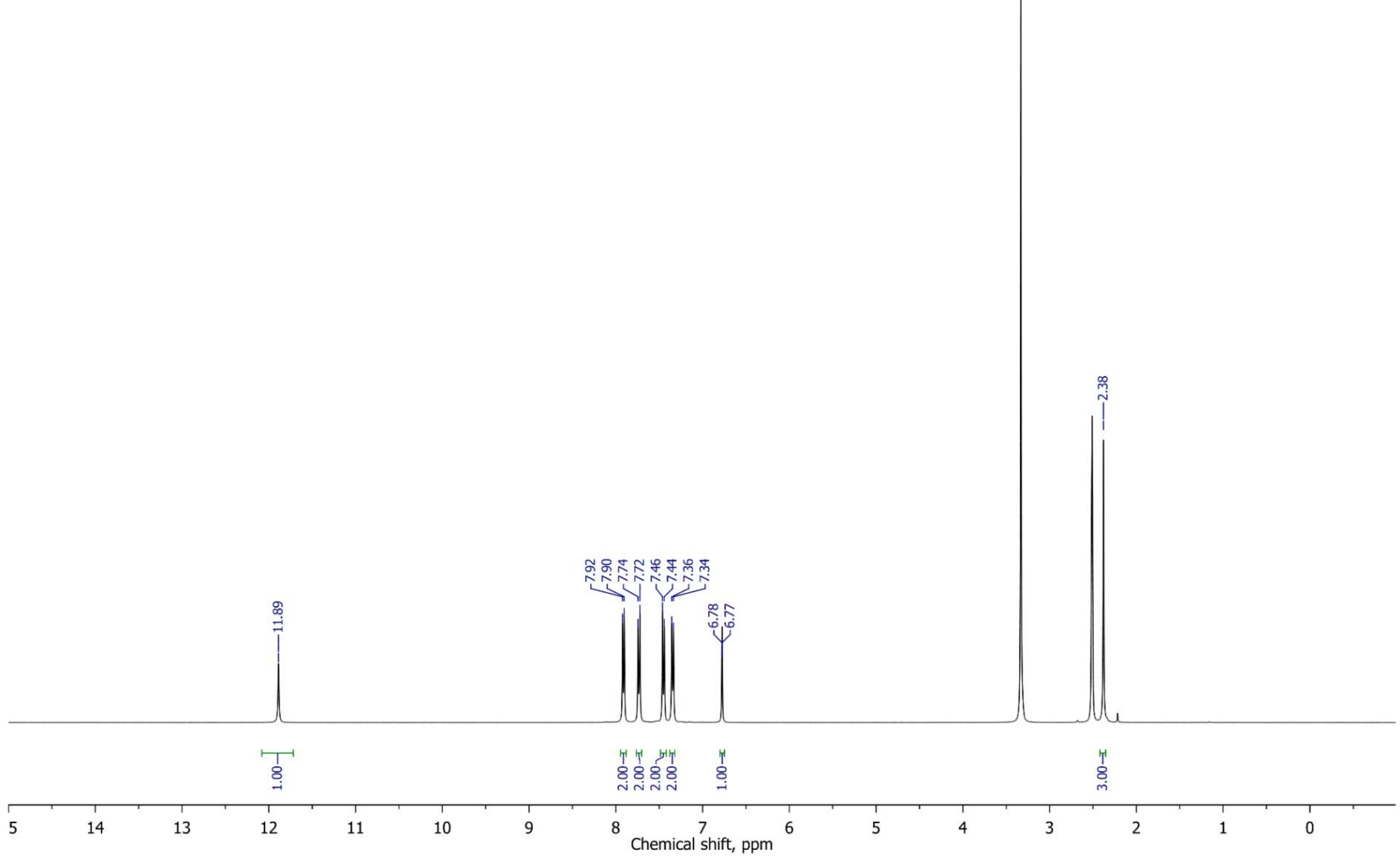

5-(4-Chlorophenyl)-2-(*p*-tolyl)-4*H*-pyrrolo[2,3-*d*]oxazole 3m, $^{13}C\{^1H\}$ NMR, 100 MHz, DMSO-d$_6$

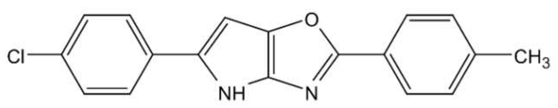

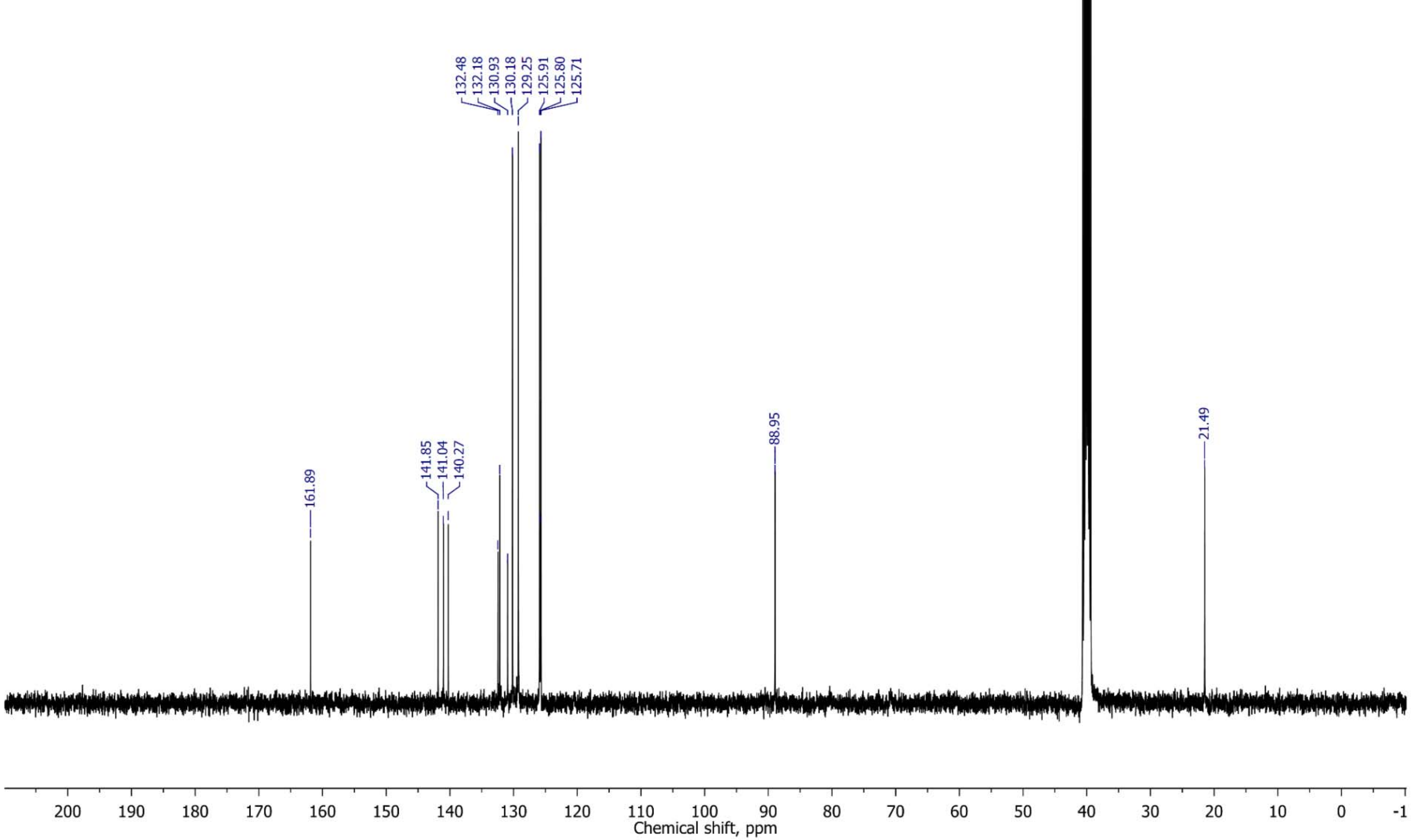

5-(4-Chlorophenyl)-2-(*p*-tolyl)-4*H*-pyrrolo[2,3-*d*]oxazole 3m, DEPT, 100 MHz, DMSO-d_6

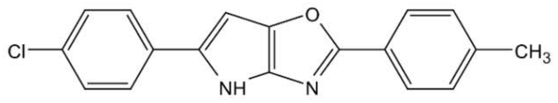

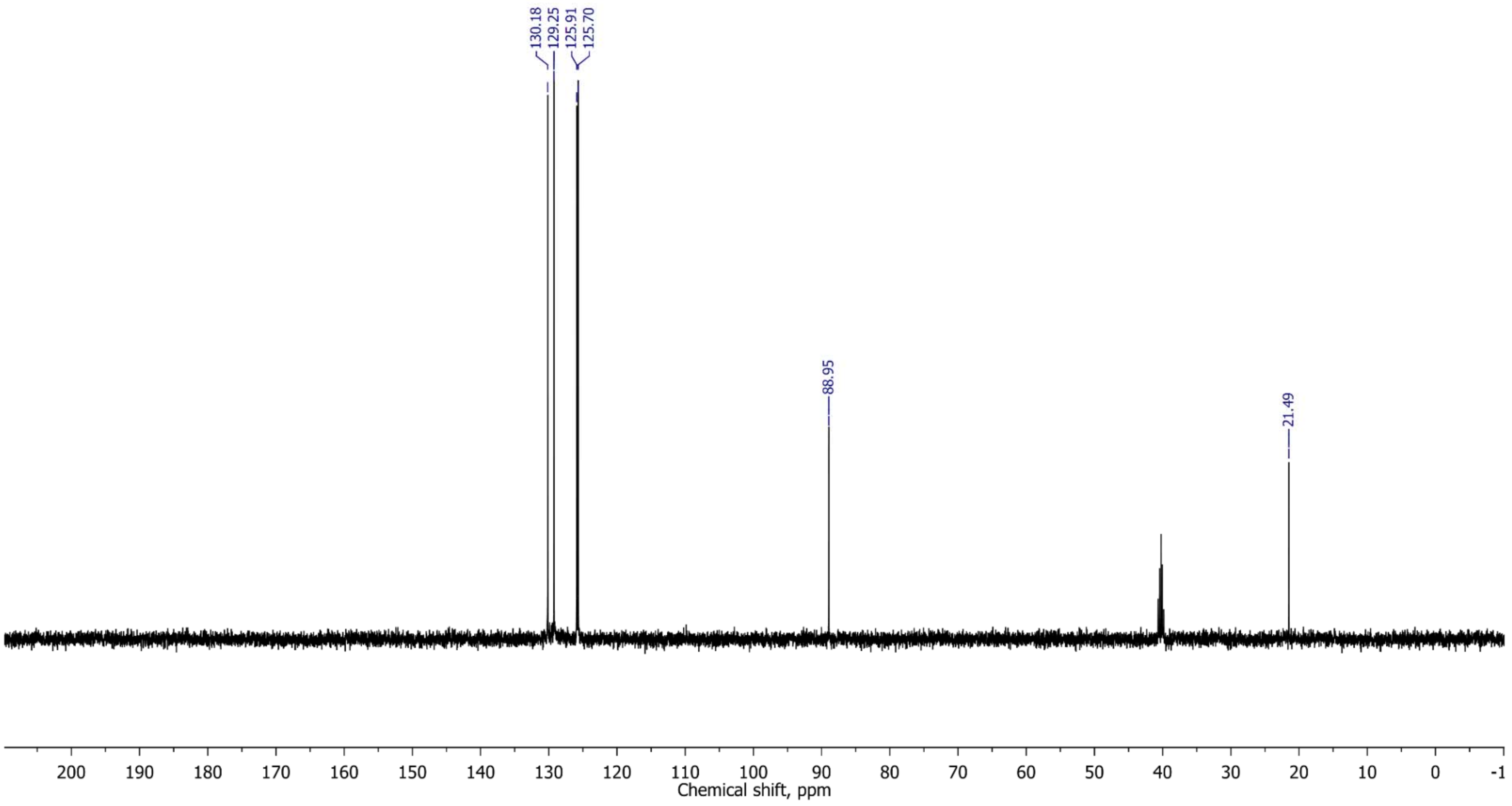

5-(Adamantan-1-yl)-2-(*p*-tolyl)-4*H*-pyrrolo[2,3-*d*]oxazole 3n, ^{1}H NMR, 400 MHz, $CDCl_3$

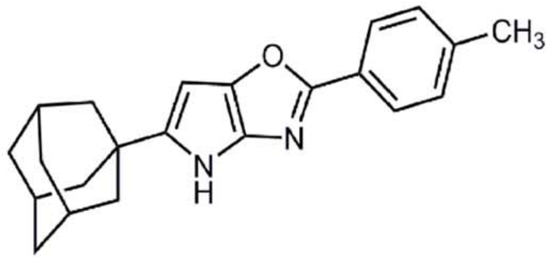

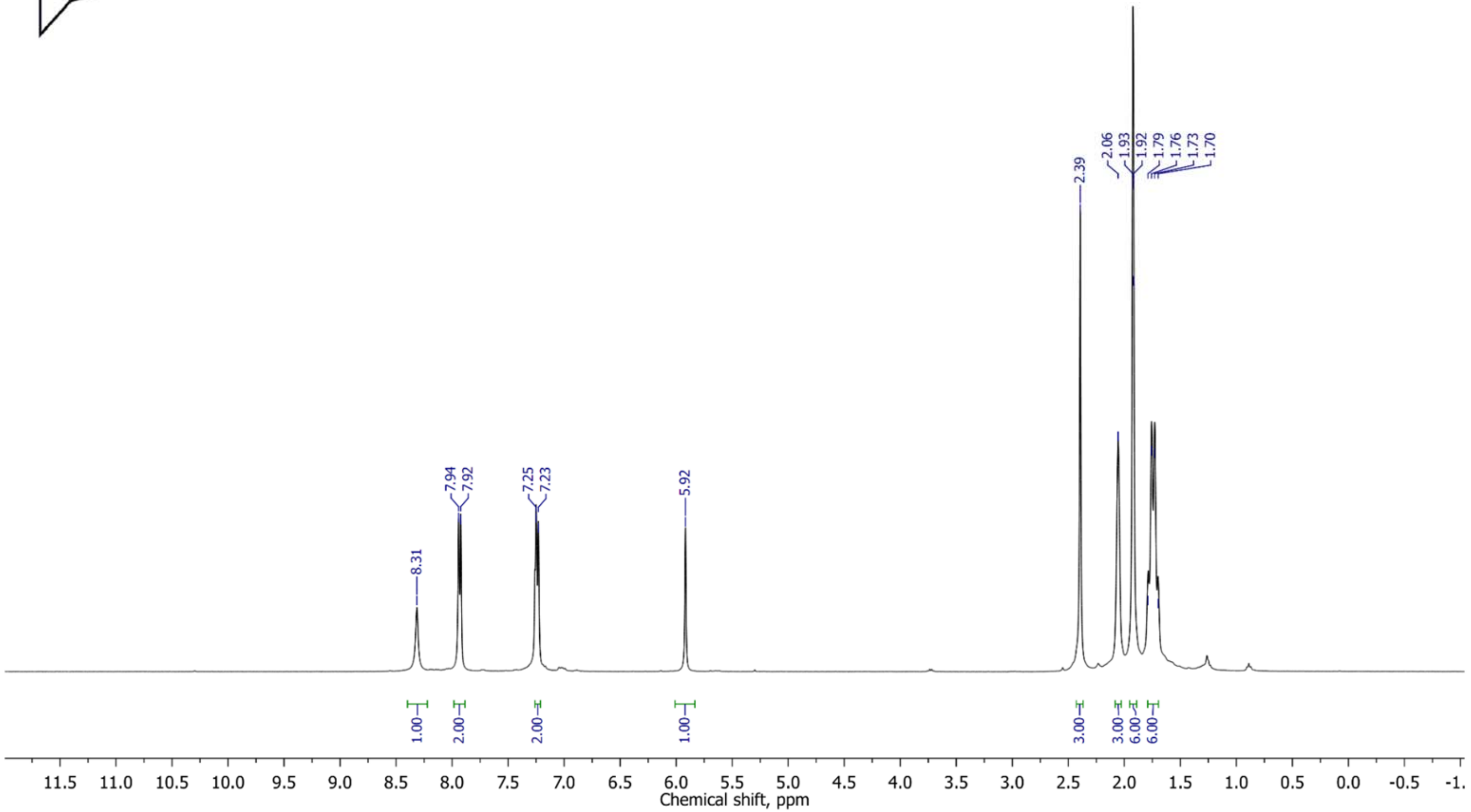

5-(Adamantan-1-yl)-2-(*p*-tolyl)-4*H*-pyrrolo[2,3-*d*]oxazole 3n, $^{13}C\{^{1}H\}$ NMR, 100 MHz, $CDCl_3$

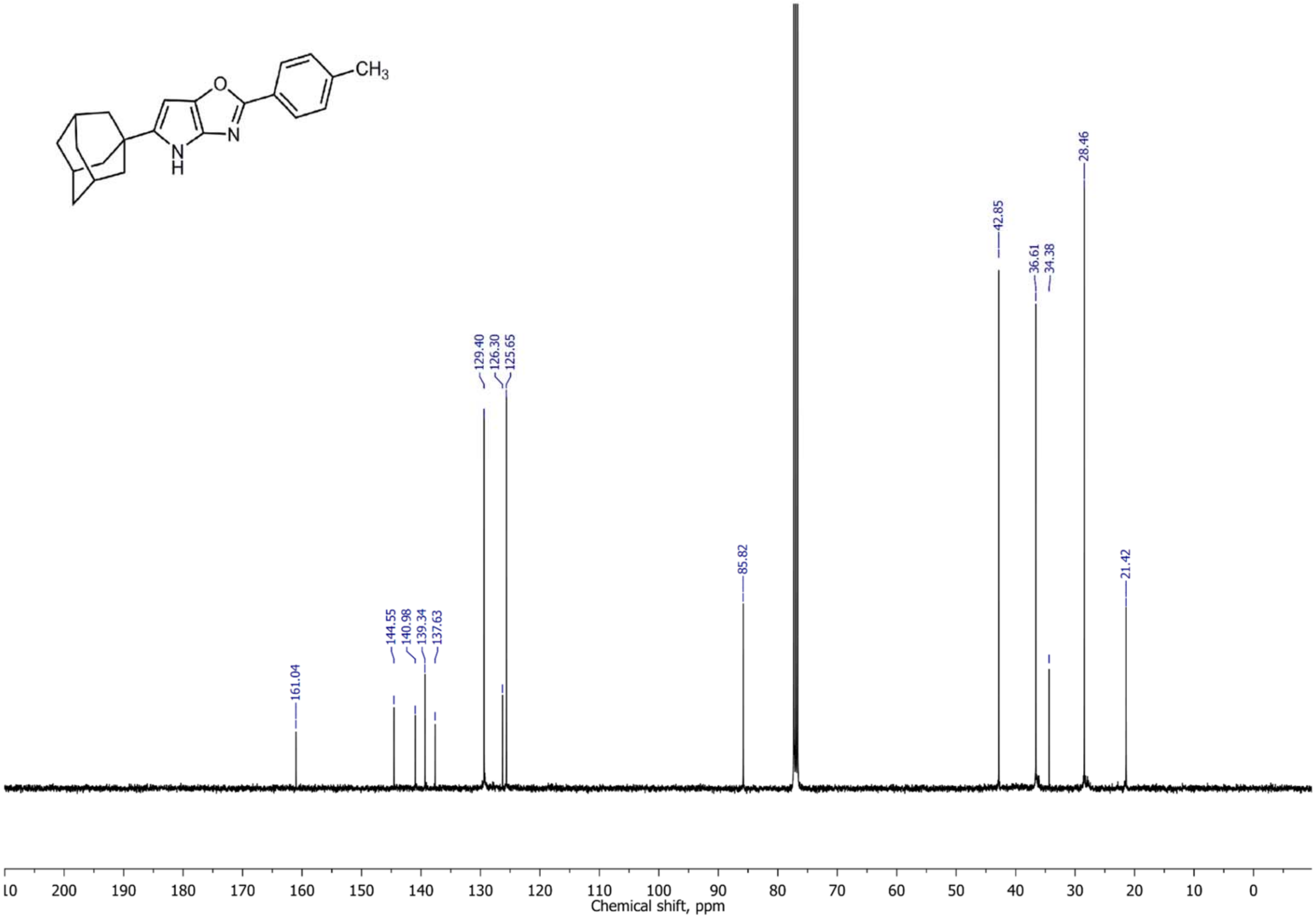

5-(Adamantan-1-yl)-2-(*p*-tolyl)-4*H*-pyrrolo[2,3-*d*]oxazole 3n, DEPT, 100 MHz, $CDCl_3$

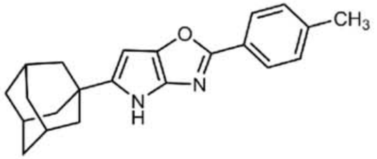

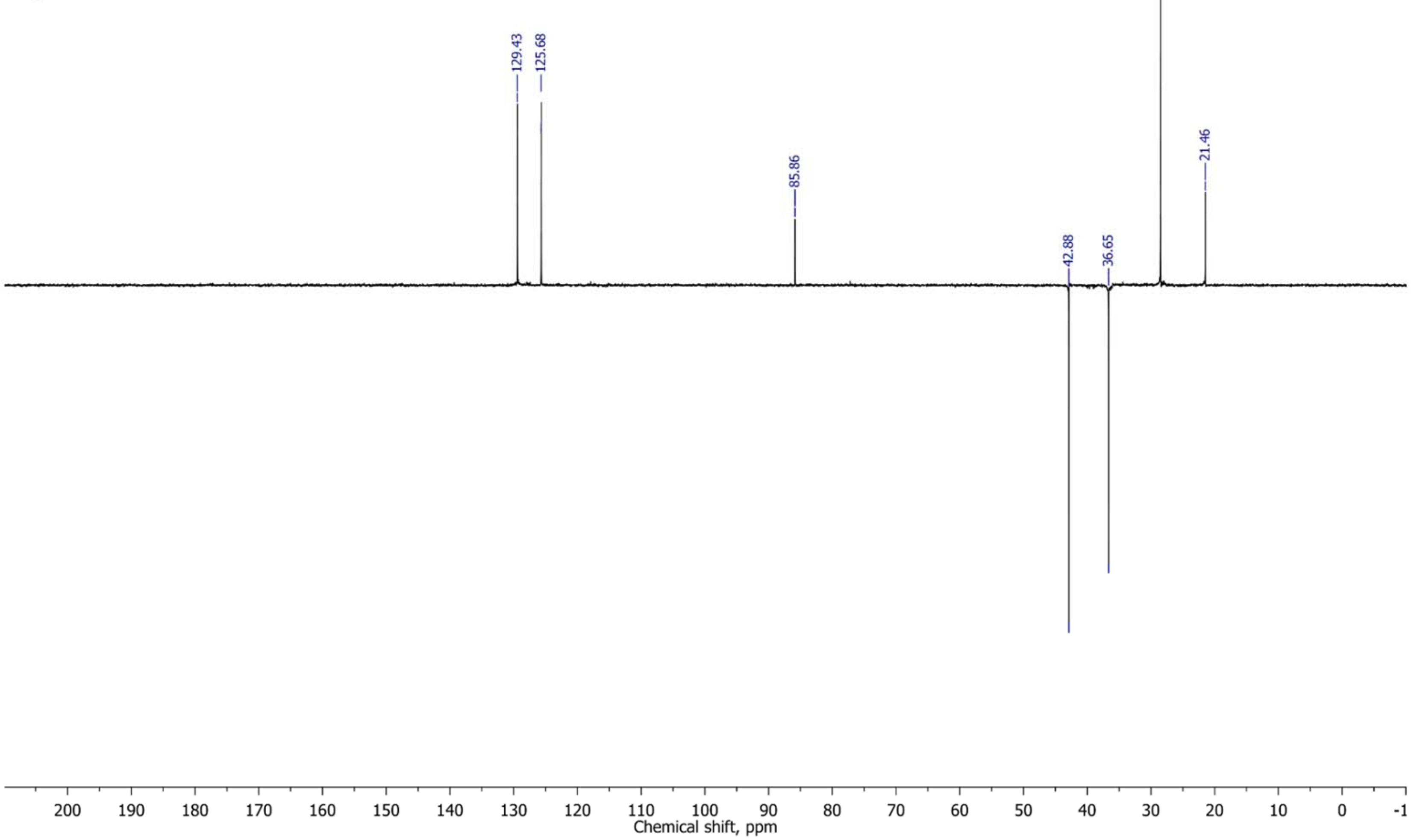

2-(4-Bromophenyl)-5-phenyl-4*H*-pyrrolo[2,3-*d*]oxazole 3o, ^{1}H NMR, 400 MHz, DMSO-d$_6$

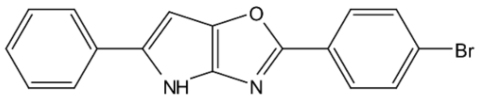

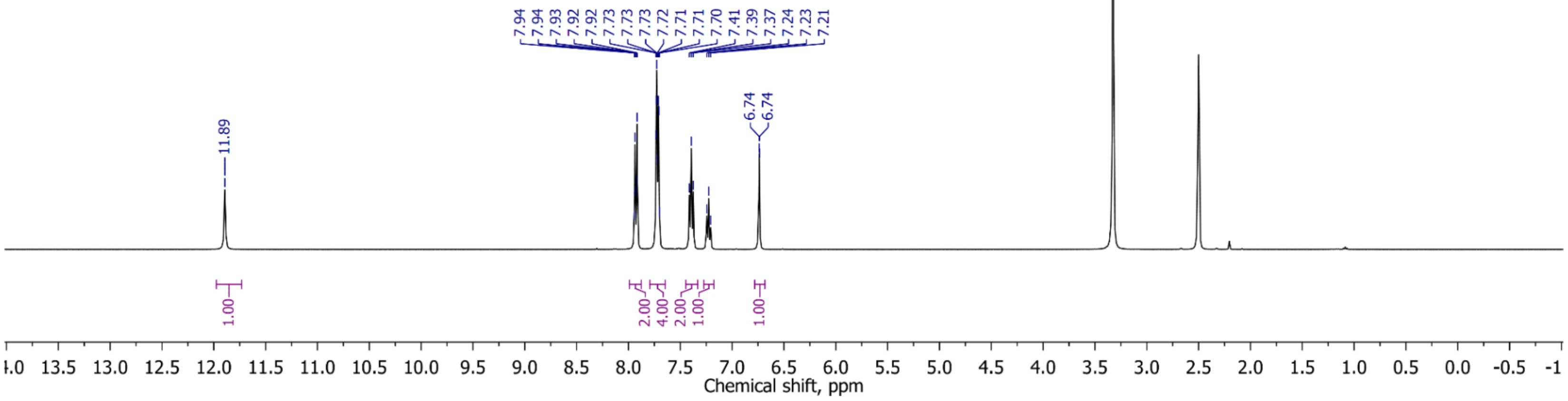

2-(4-Bromophenyl)-5-phenyl-4*H*-pyrrolo[2,3-*d*]oxazole 3o, $^{13}C\{^{1}H\}$ NMR, 100 MHz, DMSO-d_6

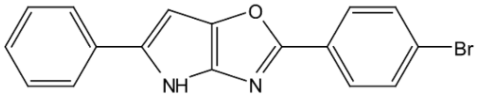

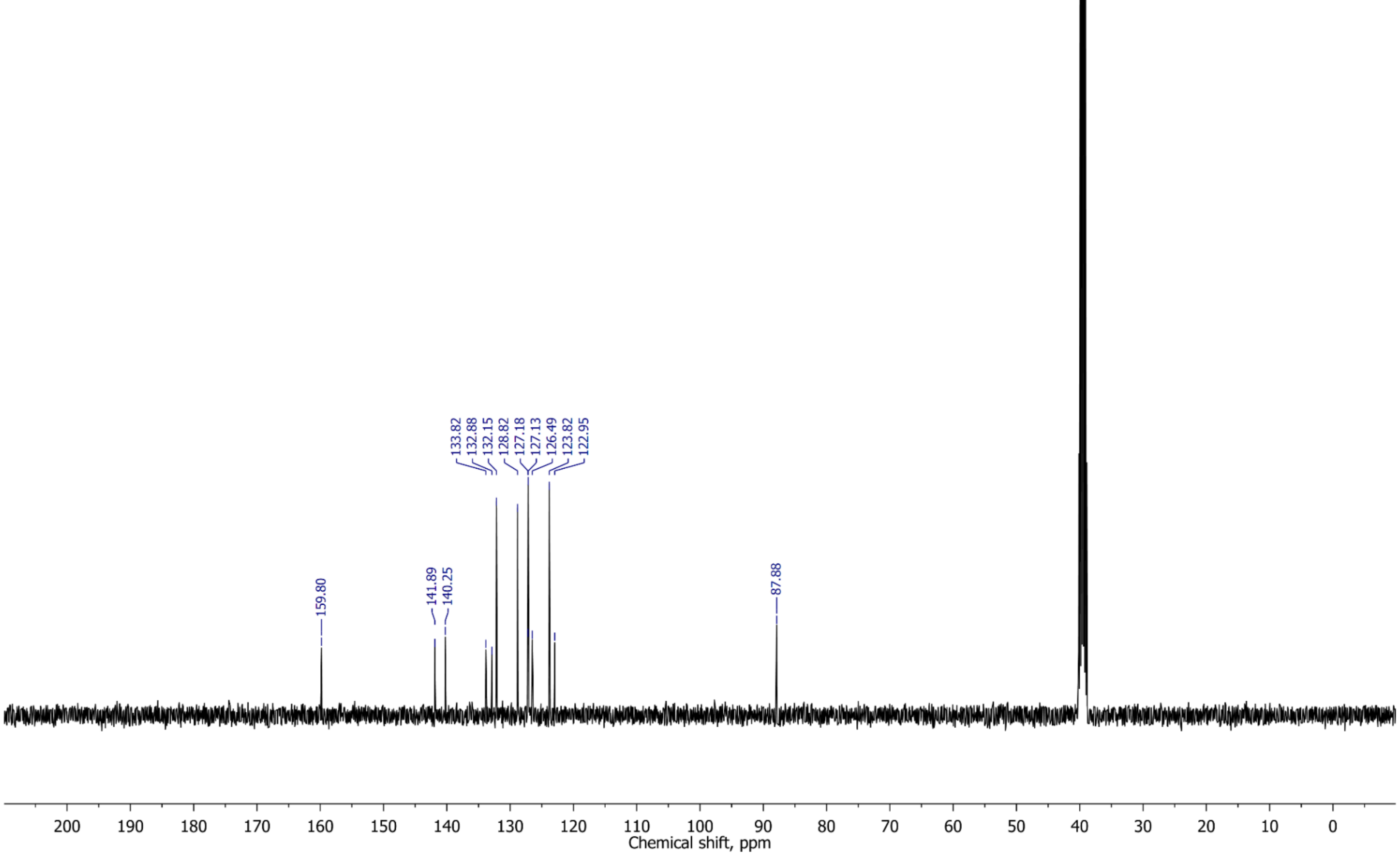

2-(4-Bromophenyl)-5-phenyl-4*H*-pyrrolo[2,3-*d*]oxazole 3o, DEPT, 100 MHz, DMSO-d$_6$

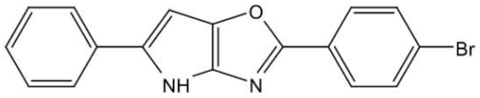

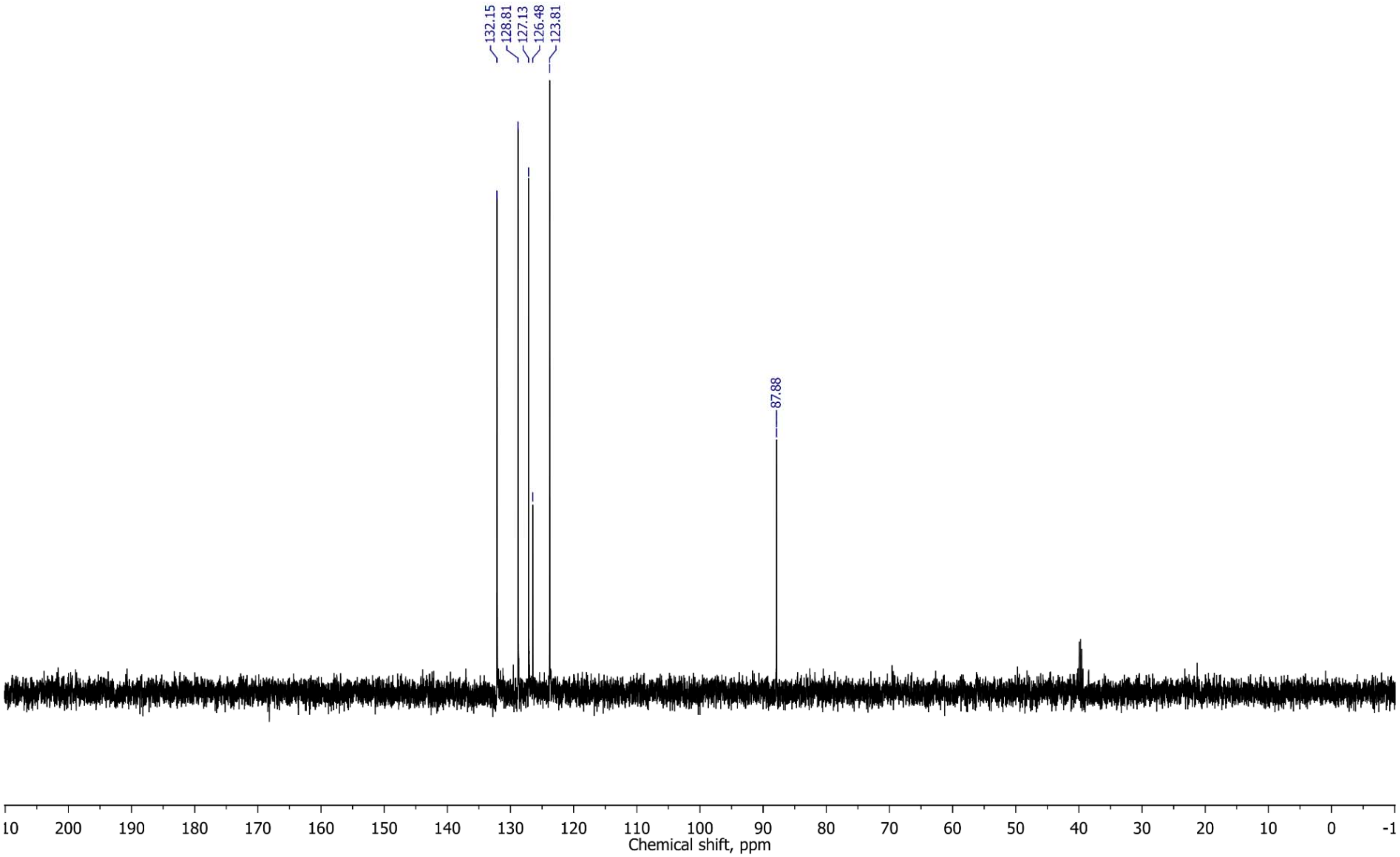

2-(4-Bromophenyl)-5-(*tert*-butyl)-4*H*-pyrrolo[2,3-*d*]oxazole 3p, ^{1}H NMR, 400 MHz, $CDCl_3$

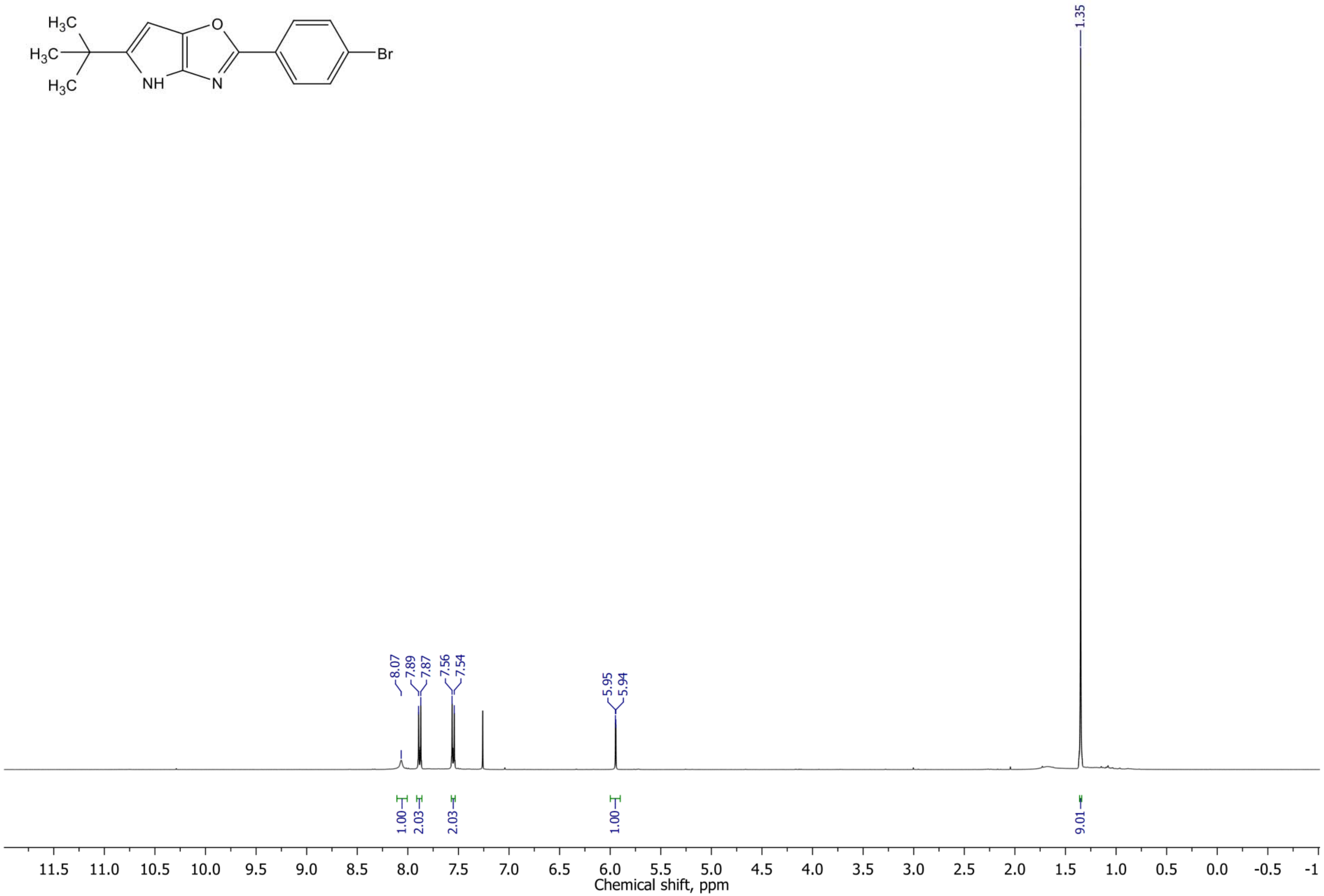

2-(4-Bromophenyl)-5-(*tert*-butyl)-4*H*-pyrrolo[2,3-*d*]oxazole 3p, $^{13}C\{^{1}H\}$ NMR, 100 MHz, $CDCl_3$

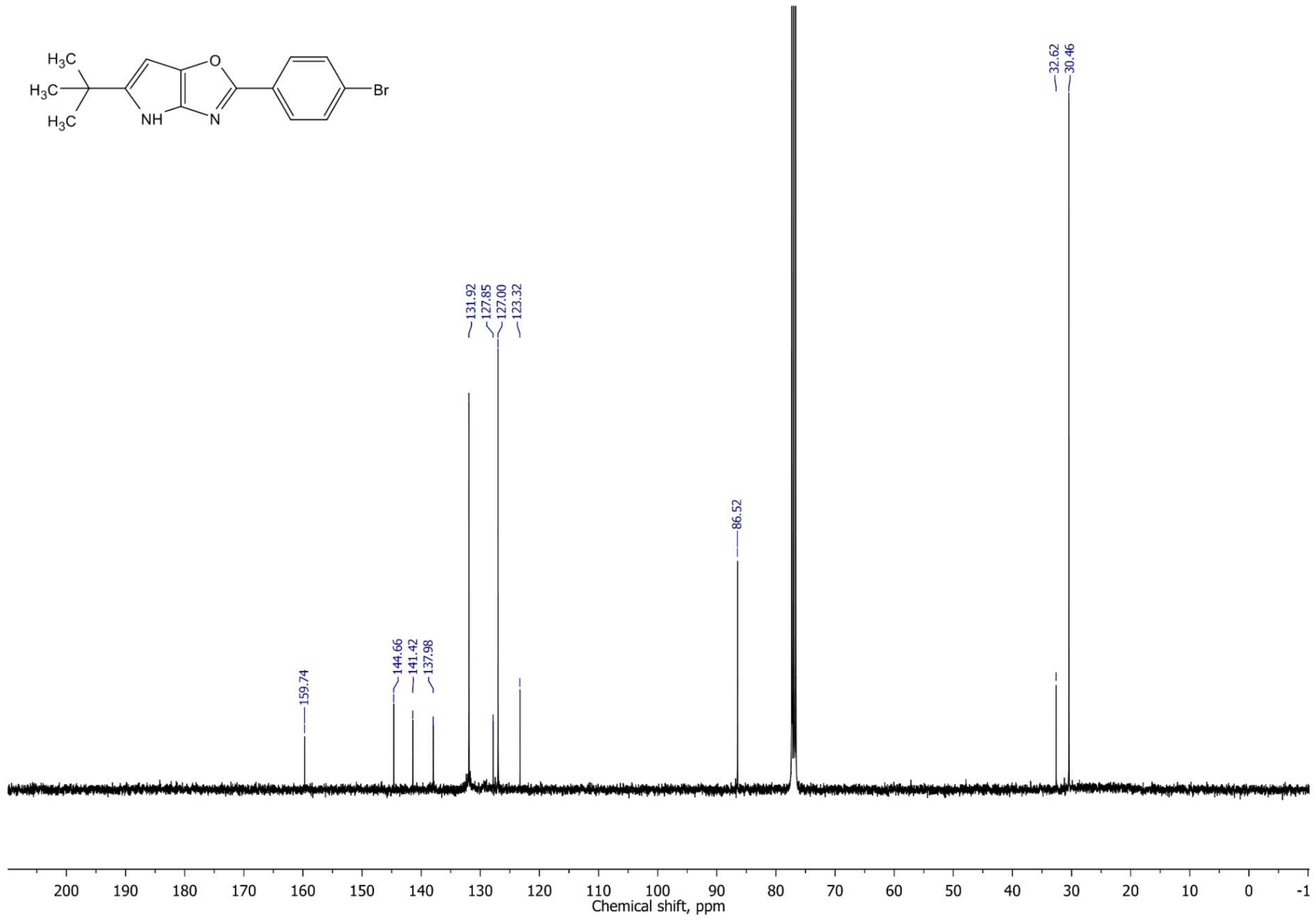

2-(4-Bromophenyl)-5-(*tert*-butyl)-4*H*-pyrrolo[2,3-*d*]oxazole 3p, DEPT, 100 MHz, $CDCl_3$

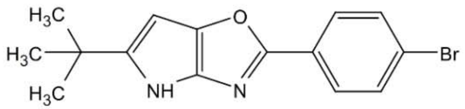

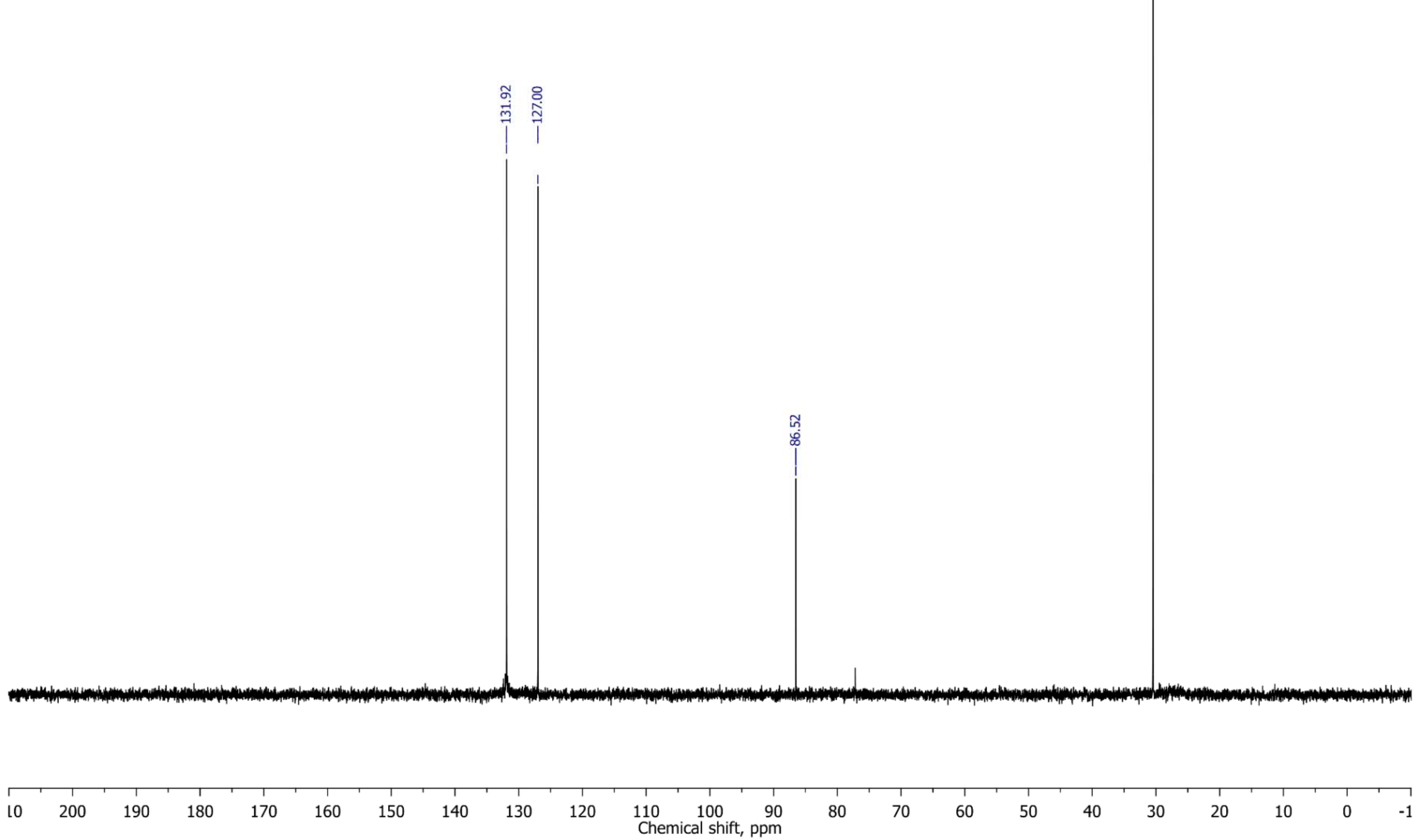

(*E*)-3-(5-phenyl-4*H*-pyrrolo[2,3-*d*]oxazol-2-yl)acrylonitrile 3q, ^{1}H NMR, 400 MHz, DMSO-d_6

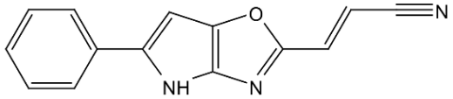

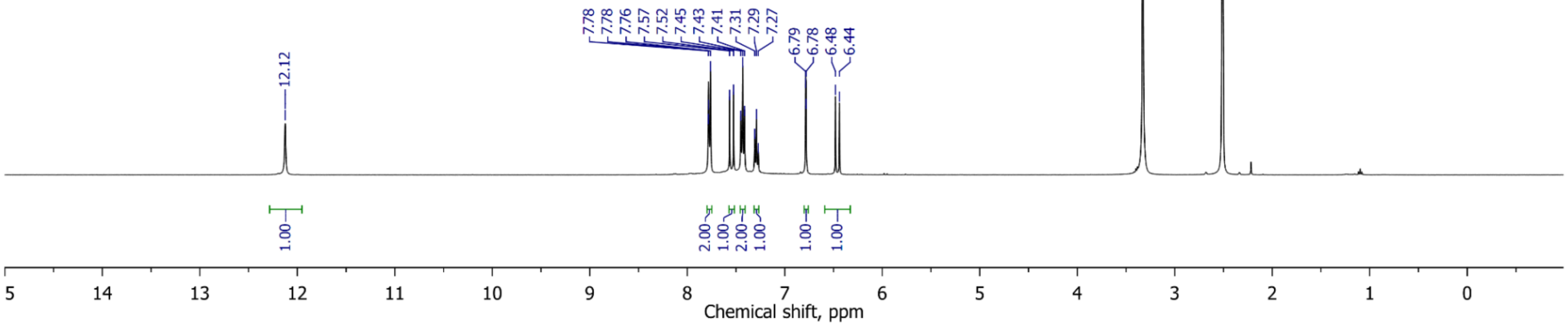

(*E*)-3-(5-phenyl-4*H*-pyrrolo[2,3-*d*]oxazol-2-yl)acrylonitrile 3q, $^{13}C\{^{1}H\}$ NMR, 100 MHz, DMSO-d_6

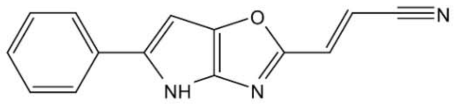

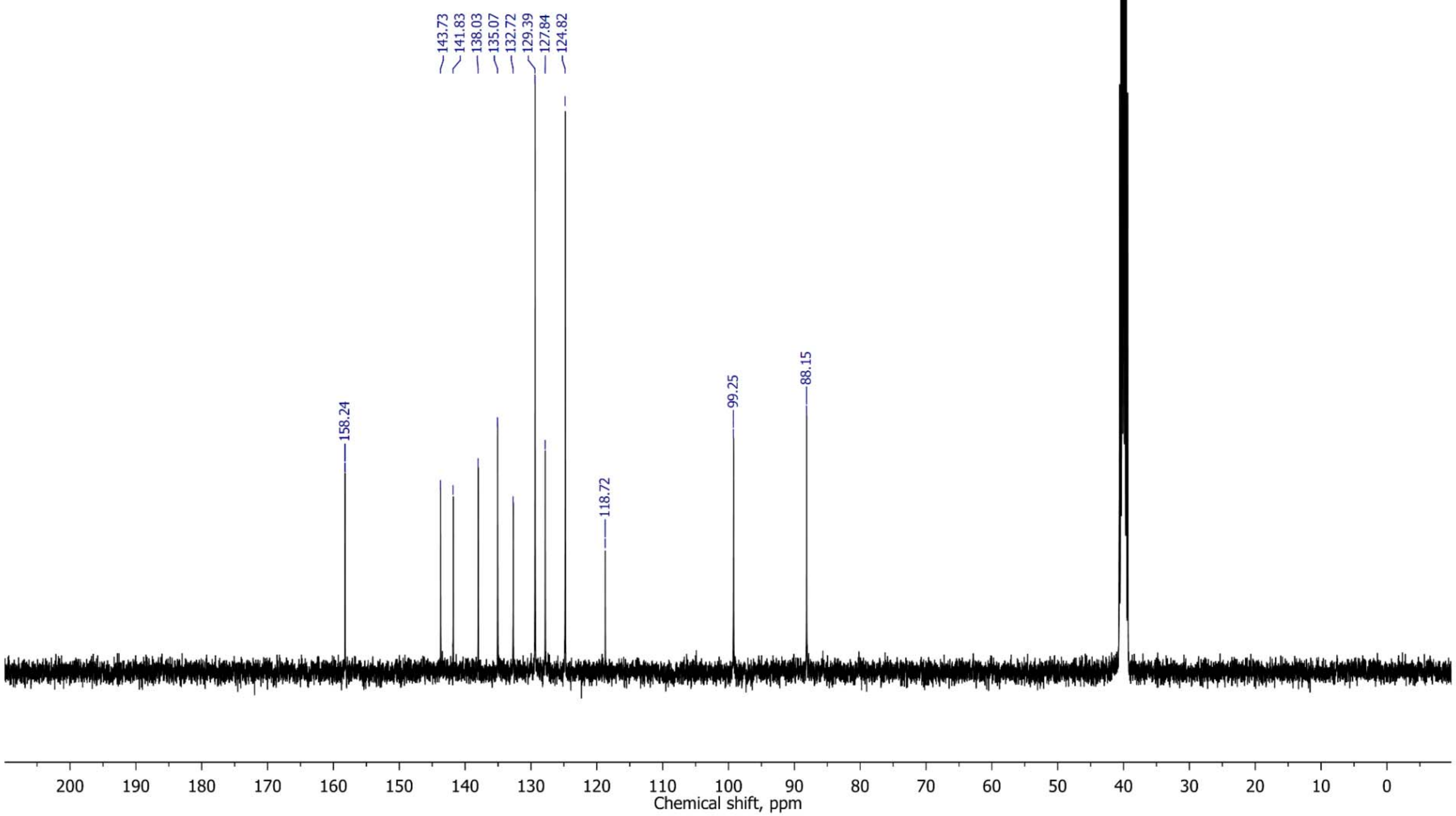

(*E*)-3-(5-phenyl-4*H*-pyrrolo[2,3-*d*]oxazol-2-yl)acrylonitrile 3q, DEPT, 100 MHz, DMSO-d_6

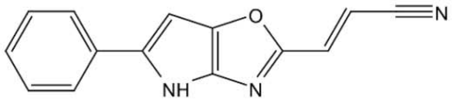

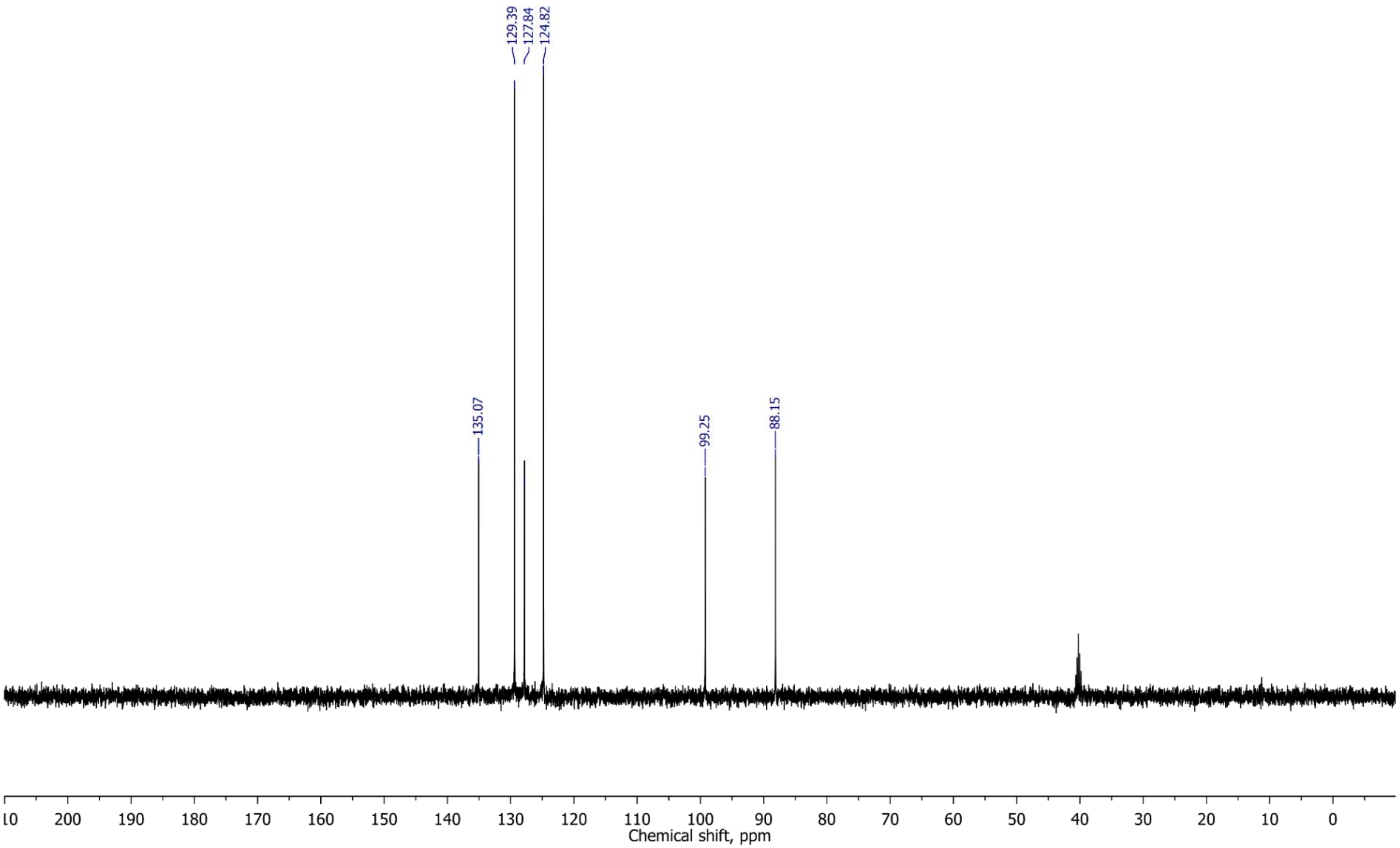

(2-Methyl-5-phenyl-4*H*-pyrrolo[2,3-*d*]oxazol-4-yl)(*p*-tolyl)methanone 4a, ^{1}H NMR, 400 MHz, $CDCl_3$

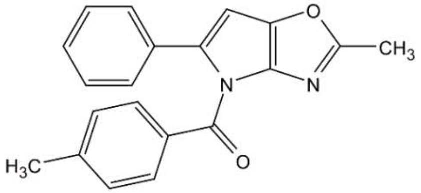

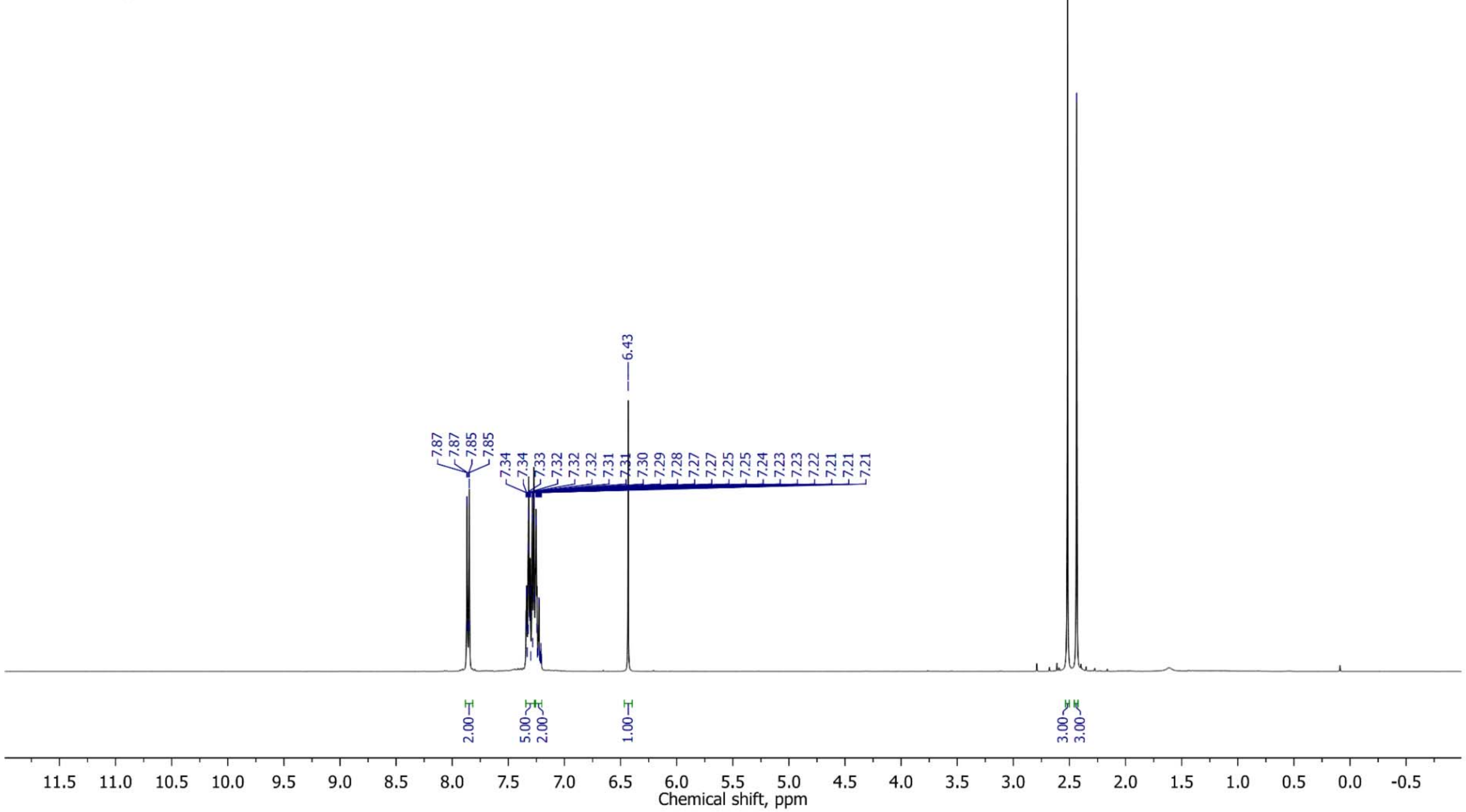

(2-Methyl-5-phenyl-4*H*-pyrrolo[2,3-*d*]oxazol-4-yl)(*p*-tolyl)methanone 4a, $^{13}C\{^1H\}$ NMR, 100 MHz, $CDCl_3$

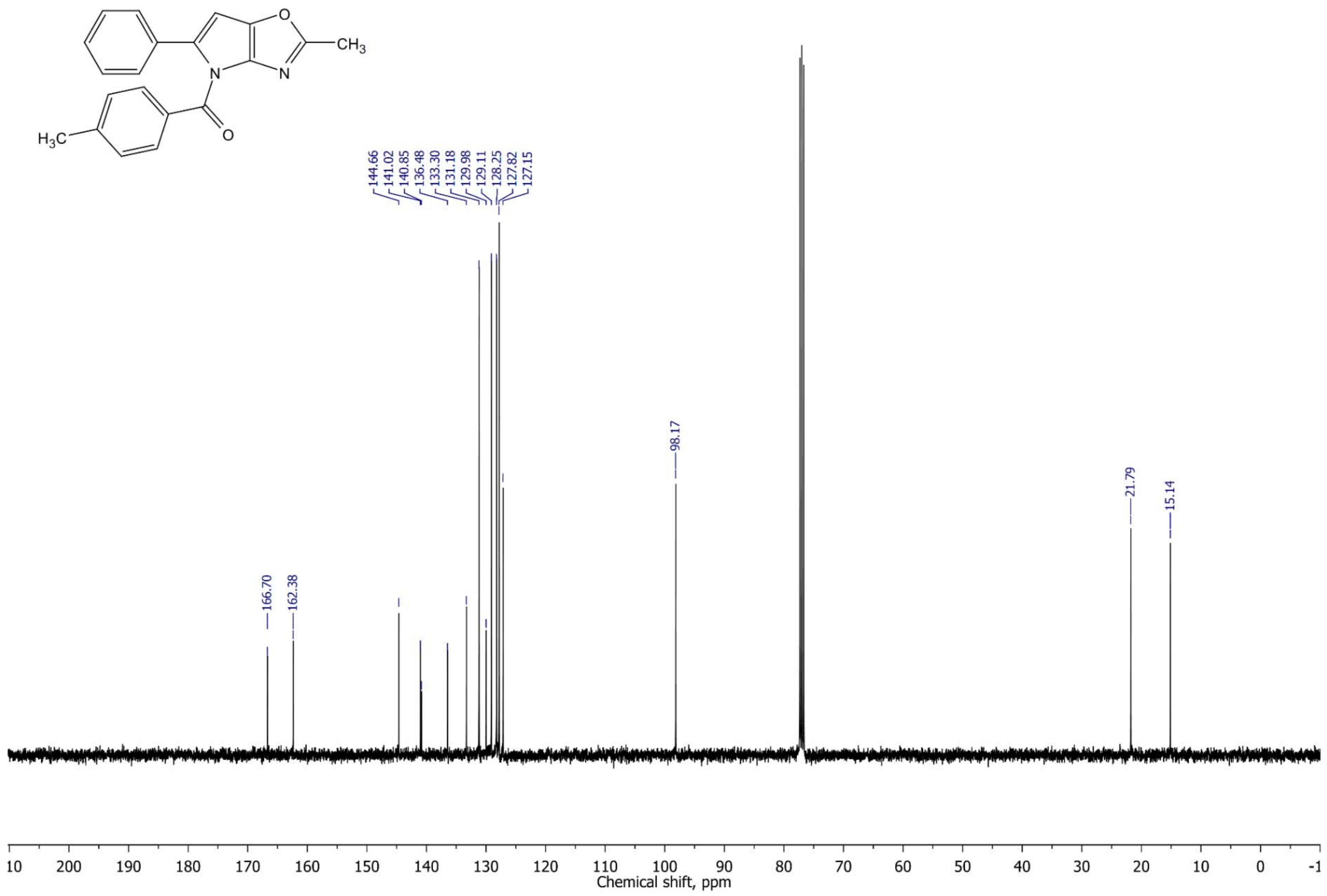

(2-Methyl-5-phenyl-4*H*-pyrrolo[2,3-*d*]oxazol-4-yl)(*p*-tolyl)methanone 4a, DEPT, 100 MHz, $CDCl_3$

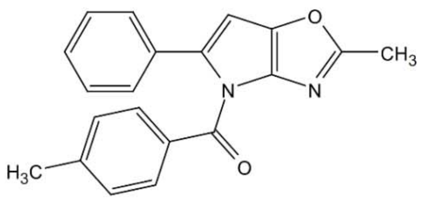

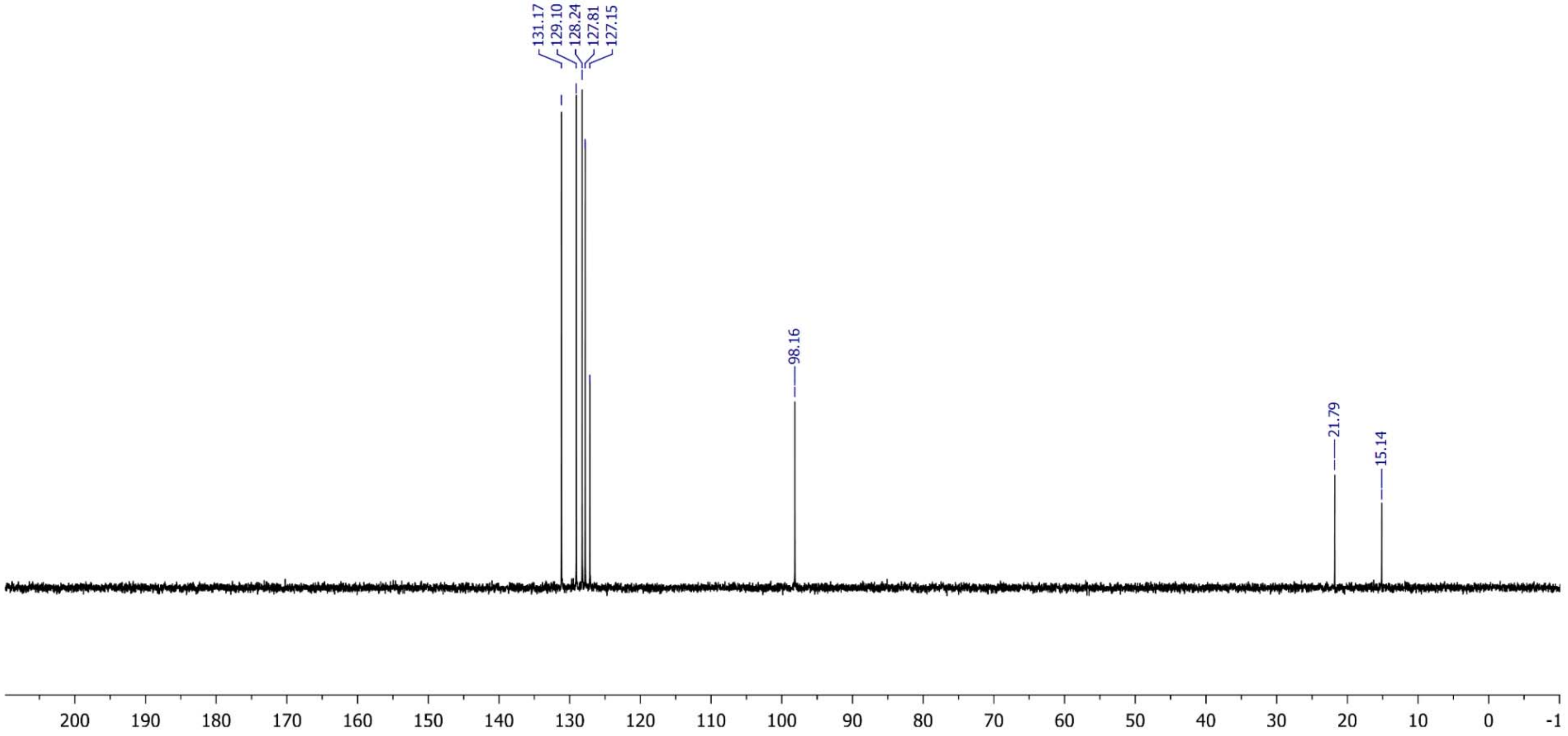

(2,5-Diphenyl-4*H*-pyrrolo[2,3-*d*]oxazol-4-yl)(*p*-tolyl)methanone 4b, ^{1}H NMR, 400 MHz, $CDCl_3$

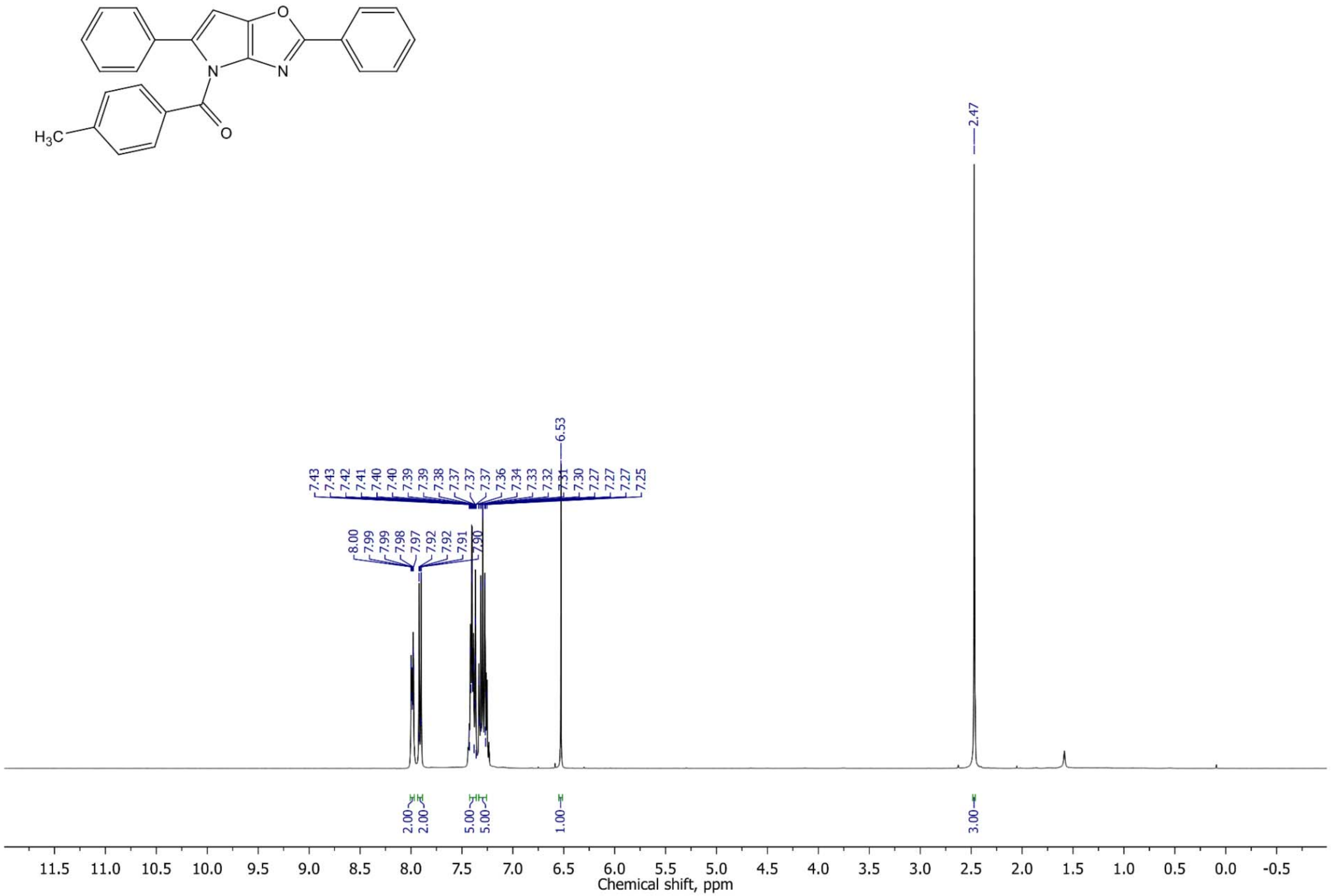

(2,5-Diphenyl-4*H*-pyrrolo[2,3-*d*]oxazol-4-yl)(*p*-tolyl)methanone 4b, $^{13}C\{^1H\}$ NMR, 100 MHz, $CDCl_3$

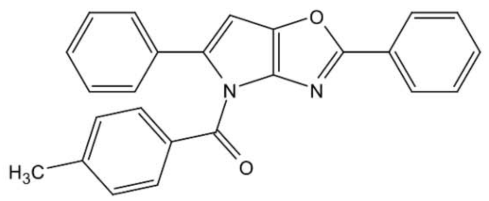

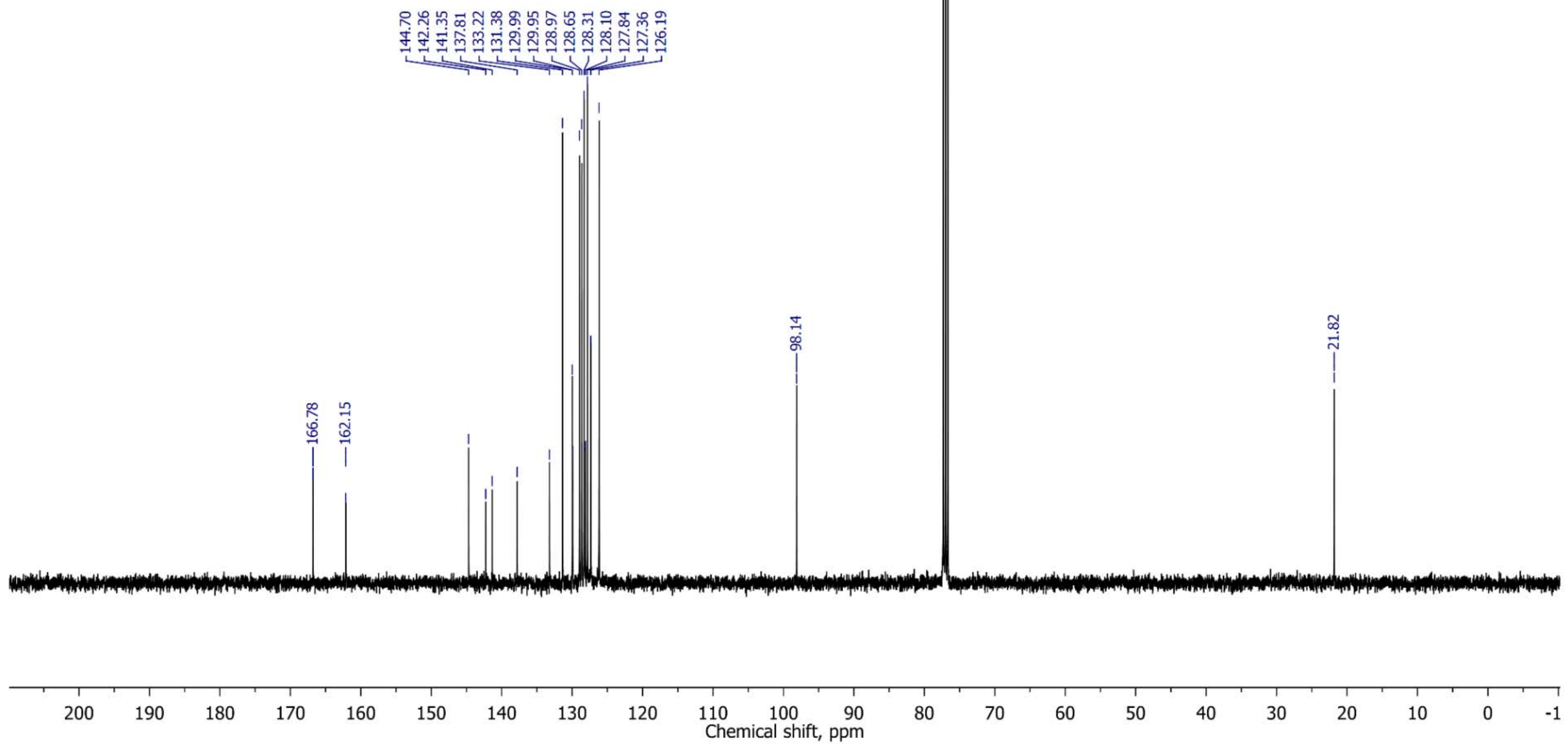

(2,5-Diphenyl-4*H*-pyrrolo[2,3-*d*]oxazol-4-yl)(*p*-tolyl)methanone 4b, DEPT, 100 MHz, $CDCl_3$

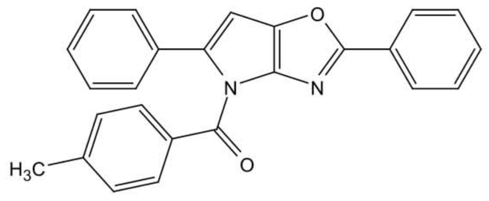

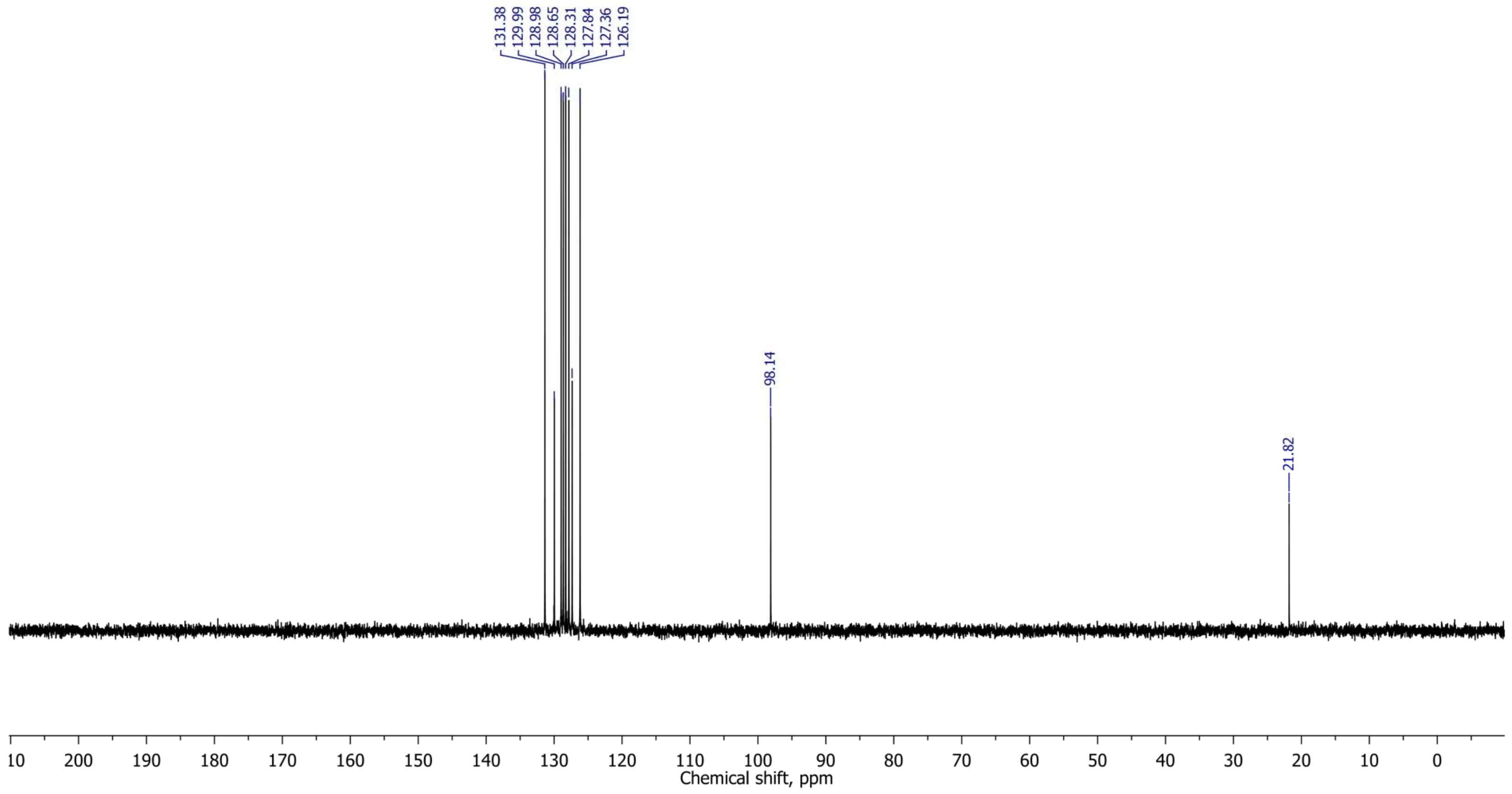

Computational Details

All calculations were performed by using the Gaussian 16 suite of quantum chemical programs[1] at Resource center "Computer center of Saint Petersburg State University". Geometry optimizations of molecules were performed with the B3LYP[2]-D3[3] density functional method and 6-311+G(d,p) basis set using SMD[4] solvent model. Stationary points on the respective potential-energy surfaces were characterized at the same level of theory by evaluating the corresponding Hessian indices. Careful verification of the unique imaginary frequency for the transition state was carried out to check whether the frequency indeed pertains to the desired reaction coordinate.

Table S7. B3LYP-D3/6-311+G(d,p), SMD solvent model for mesitylene.

Absolute Energies (au), Cartesian Coordinates of stationary points

Molecule 2a

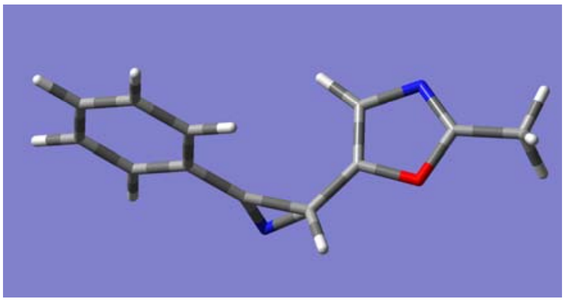

E = -648.157600, H (0K) = -647.964700,
H (298K) = -647.950975,
G (298K) = -648.007342 au.
Imaginary frequency = 0.

N	-0.1844340	-2.1156980	-0.0904160
C	-0.7627840	-1.0587490	0.2753100
C	-2.0143230	-0.3428910	0.1550020
C	-4.2835410	-0.2224190	-0.6614600
H	-5.1126700	-0.6611060	-1.2046020
C	-2.1541080	0.9205340	0.7433660
H	-1.3195260	1.3538860	1.2826410
C	-4.4209750	1.0383240	-0.0738580
C	-3.0844680	-0.9145570	-0.5506480
H	-2.9632780	-1.8926500	-1.0022920
C	-3.3581690	1.6087090	0.6267700
H	-3.4680830	2.5872810	1.0795240
H	-5.3585440	1.5754130	-0.1637450
C	0.6080140	-1.0764520	0.7602730
H	0.8234210	-1.3749460	1.7820450
C	1.6622730	-0.3068050	0.0977450
C	1.7478980	0.4559100	-1.0227330
O	2.9165040	-0.3274170	0.6663130
H	0.9708640	0.7016630	-1.7284700
C	3.6974360	0.4316310	-0.1524690
C	5.1232210	0.6007310	0.2183170
H	5.6155430	1.2247970	-0.5265500
H	5.2160890	1.0747180	1.1999280
H	5.6296190	-0.3676730	0.2650930
N	3.0518180	0.9148640	-1.1678360

TS (2a-3'a)

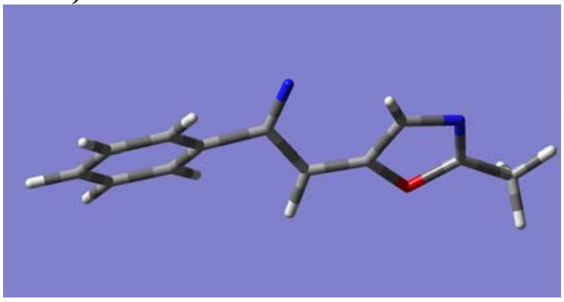

E = -648.098093, H (0K) = -647.907189,
H (298K) = -647.894304,
G (298K) = -647.946297 au.
Imaginary frequency = 1.

N	0.2497300	1.6546120	0.7006630
C	0.5960960	0.5193720	0.2622410
C	2.0527760	0.1369910	0.0920110
C	4.3317010	0.7431380	-0.4359960
H	5.0526080	1.4862030	-0.7587380
C	2.4732320	-1.1614140	0.3900210
H	1.7549710	-1.9006980	0.7293660
C	4.7504770	-0.5559140	-0.1436270
C	2.9897260	1.0923190	-0.3110410
H	2.6556480	2.1011900	-0.5218720
C	3.8197320	-1.5058100	0.2744160
H	4.1398670	-2.5136900	0.5147400
H	5.7970780	-0.8241310	-0.2355580
C	-0.4067780	-0.4493840	-0.1885790
H	-0.1221530	-1.3630500	-0.6993090
C	-1.7210070	-0.0382690	-0.2077040
C	-2.3175580	1.2167410	-0.0229020
O	-2.7626460	-0.9474570	-0.3055850
H	-1.8593820	2.1813770	-0.1552950
C	-3.8841690	-0.2270790	-0.0518360
C	-5.1699460	-0.9535590	-0.0154440
H	-5.9796610	-0.2508830	0.1733810
H	-5.3445270	-1.4690740	-0.9643860
H	-5.1532730	-1.7107710	0.7747020
N	-3.6676860	1.0511570	0.1190920

Molecule 3'a

TS (3'a-3a)

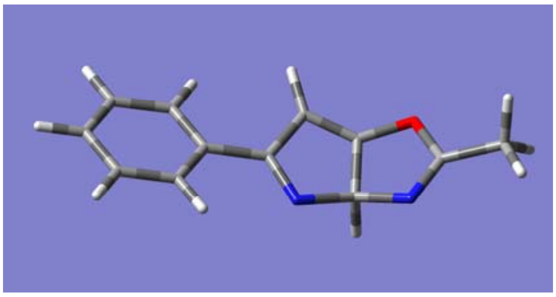

E = -648.169593, H (0K) = -647.975062,
H (298K) = -647.962480,
G (298K) = -648.013872 au.
Imaginary frequency = 0.

N	-0.2914840	-1.0836640	0.4759810
C	0.3791840	0.0078040	0.2309730
C	1.8428700	-0.0028290	0.0454990
C	3.8919270	-1.2374910	-0.3473810
H	4.3981240	-2.1803800	-0.5219870
C	2.5868620	1.1842250	0.0915790
H	2.0925190	2.1308580	0.2743980
C	4.6254890	-0.0494060	-0.2977790
C	2.5135830	-1.2165800	-0.1754530
H	1.9380680	-2.1332230	-0.2113540
C	3.9701770	1.1597560	-0.0757280
H	4.5340860	2.0847190	-0.0313230
H	5.7013100	-0.0683840	-0.4323080
C	-0.4575000	1.2360940	0.0911430
H	-0.1371060	2.1929690	-0.2891870
C	-1.6937770	0.7889880	0.3386580
C	-1.6542970	-0.6429110	0.7357800
O	-2.9646120	1.1198860	-0.0088700
H	-1.7948810	-0.7684980	1.8214340
C	-3.5675900	-0.1599350	-0.2057490
C	-4.9434050	-0.0968410	-0.7468900
H	-5.3178000	-1.1054650	-0.9144820
H	-5.5977270	0.4233940	-0.0413730
H	-4.9527890	0.4636940	-1.6855200
N	-2.8668090	-1.1769100	0.0946960

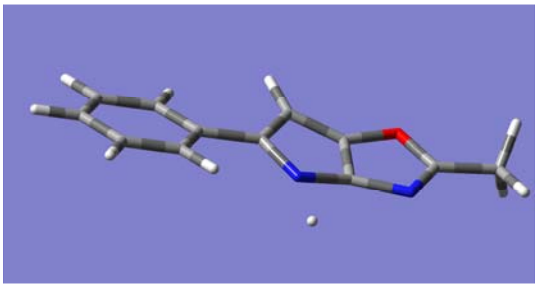

E = -648.123341, H (0K) = -647.933226,
H (298K) = -647.920753,
G (298K) = -647.971817 au.
Imaginary frequency = 1.

N	-0.2952580	-1.1145400	0.1474860
C	0.4099750	0.0496000	-0.0269190
C	1.8807810	0.0201340	-0.0199990
C	3.9581900	-1.2280980	-0.1879130
H	4.4702700	-2.1758150	-0.3136000
C	2.6312110	1.1968530	0.1317590
H	2.1286830	2.1456220	0.2792020
C	4.6937970	-0.0506780	-0.0437520
C	2.5679810	-1.1950710	-0.1790210
H	2.0008820	-2.1097120	-0.3009140
C	4.0231880	1.1611000	0.1154690
H	4.5841060	2.0812970	0.2368030
H	5.7776640	-0.0782460	-0.0534570
C	-0.4188370	1.2008080	-0.1517120
H	-0.0927080	2.2039050	-0.3765950
C	-1.6970970	0.7359090	0.0775680
C	-1.6573140	-0.6473780	0.2906280
O	-3.0023240	1.0914180	-0.0905710
H	-0.9476490	-1.0396630	1.2835430
C	-3.6814810	-0.1311700	-0.0505340
C	-5.1545570	-0.0510450	-0.1698540
H	-5.5691120	-1.0568500	-0.2203790
H	-5.5780500	0.4687120	0.6951730
H	-5.4356340	0.5074990	-1.0664400
N	-2.9411540	-1.1774420	0.1163600

Molecule 3a

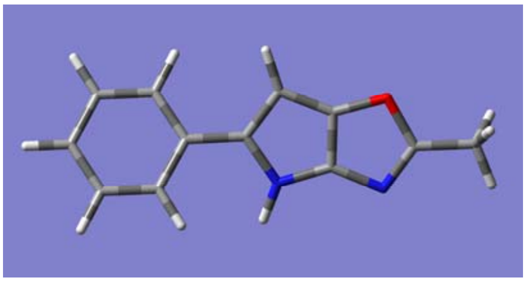

E = -648.208497, H (0K) = -648.013163,
H (298K) = -648.000266,
G (298K) = -648.051998 au.
Imaginary frequency = 0.

C	-0.4244540	0.1155300	0.0108450	
C	-1.8836370	0.0426000	0.0055190	
C	-3.9493230	-1.2226920	0.2450950	
H	-4.4469600	-2.1655800	0.4440120	
C	-2.6545680	1.1911730	-0.2483700	
H	-2.1587660	2.1297110	-0.4671960	
C	-4.7008990	-0.0749660	-0.0030520	
C	-2.5584030	-1.1653680	0.2566790	
H	-1.9964310	-2.0626440	0.4923210	
C	-4.0436920	1.1326000	-0.2446250	
H	-4.6166660	2.0314090	-0.4449040	
H	-5.7839800	-0.1197240	-0.0078290	
C	0.4051540	1.2163530	0.2559180	
H	0.0719740	2.2095380	0.5071160	
C	1.7131450	0.7066290	0.1498410	
C	1.6724820	-0.6310830	-0.1470230	
O	3.0352420	1.0574390	0.2371400	
C	3.7069750	-0.1224070	-0.0268540	
C	5.1870940	-0.0652620	-0.0064980	
H	5.5878930	-1.0523160	-0.2343350	
H	5.5591420	0.6515930	-0.7447390	
H	5.5501610	0.2500740	0.9764790	
N	2.9385320	-1.1522520	-0.2610660	
N	0.3638890	-1.0136320	-0.2212920	
H	0.0155070	-1.9090250	-0.5263950	

References

(1) Gaussian 16, Revision A.03, Frisch, M. J.; Trucks, G. W.; Schlegel, H. B.; Scuseria, G. E.; Robb, M. A.; Cheeseman, J. R.; Scalmani, G.; Barone, V.; Petersson, G. A.; Nakatsuji, H.; Li, X.; Caricato, M.; Marenich, A. V.; Bloino, J.; Janesko, B. G.; Gomperts, R.; Mennucci, B.; Hratchian, H. P.; Ortiz, J. V.; Izmaylov, A. F.; Sonnenberg, J. L.; Williams-Young, D.; Ding, F.; Lipparini, F.; Egidi, F.; Goings, J.; Peng, B.; Petrone, A.; Henderson, T.; Ranasinghe, D.; Zakrzewski, V. G.; Gao, J.; Rega, N.; Zheng, G.; Liang, W.; Hada, M.; Ehara, M.; Toyota, K.; Fukuda, R.; Hasegawa, J.; Ishida, M.; Nakajima, T.; Honda, Y.; Kitao, O.; Nakai, H.; Vreven, T.; Throssell, K.; Montgomery, J. A., Jr.; Peralta, J. E.; Ogliaro, F.; Bearpark, M. J.; Heyd, J. J.; Brothers, E. N.; Kudin, K. N.; Staroverov, V. N.; Keith, T. A.; Kobayashi, R.; Normand, J.; Raghavachari, K.; Rendell, A. P.; Burant, J. C.; Iyengar, S. S.; Tomasi, J.; Cossi, M.; Millam, J. M.; Klene, M.; Adamo, C.; Cammi, R.; Ochterski, J. W.; Martin, R. L.; Morokuma, K.; Farkas, O.; Foresman, J. B.; Fox, D. J. Gaussian, Inc., Wallingford CT, 2016.

(2) (a) Becke, A. D. *J. Chem. Phys.* **1993**, *98*, 5648–5652. (b) Becke, A. D. *Phys. Rev. A* **1988**, *38*, 3098–3100. (c) Lee, C.; Yang, W.; Parr, R. G. *Phys. Rev. B* **1988**, *37*, 785–789.

(3) (a) Grimme, S.; Antony, J.; Ehrlich, S.; Krieg, H. *J. Chem. Phys.* **2010**, *132*, 1054104. (b) Grimme, S.; Ehrlich, S.; Goerigk, L. *J. Comput. Chem.* **2011**, *32*, 1456–1465.

(4) Marenich, A. V.; Cramer, C. J.; Truhlar, D. G. *J. Phys. Chem. B*, **2009**, *11* , 6378-6396.
